# Functional alterations and predictive capacity of gut microbiome in type 2 diabetes

**DOI:** 10.1038/s41598-023-49679-w

**Published:** 2023-12-16

**Authors:** Nihar Ranjan Dash, Mohammad T. Al Bataineh, Rohia Alili, Habiba Al Safar, Noura Alkhayyal, Edi Prifti, Jean-Daniel Zucker, Eugeni Belda, Karine Clément

**Affiliations:** 1 Department of Clinical Sciences, College of Medicine, University of Sharjah, Sharjah, United Arab Emirates; 2 Department of Genetics and Molecular Biology, College of Medicine and Health Sciences, Khalifa University of Science and Technology, PO Box: 127788, Abu Dhabi, United Arab Emirates; 3 Center for Biotechnology, Khalifa University of Science and Technology, Abu Dhabi, UAE; 4 INSERM, Nutrition and obesities: systemics approaches (NutriOmics), Sorbonne University, Paris, France; 5 Nutrition Department, Pitié-Salpêtrière Hospital, Assistance Publique Hôpitaux de Paris, Paris, France; 6 University Hospital Sharjah, Sharjah, United Arab Emirates; 7 Unité de Modélisation Mathématique et Informatique des Systèmes Complexes, UMMISCO, IRD, Sorbonne Université, 93143 Bondy, France

**Keywords:** Microbiome, Bioinformatics, Endocrinology

## Abstract

The gut microbiome plays a significant role in the development of Type 2 Diabetes Mellitus (T2DM), but the functional mechanisms behind this association merit deeper investigation. Here, we used the nanopore sequencing technology for metagenomic analyses to compare the gut microbiome of individuals with T2DM from the United Arab Emirates (n = 40) with that of control (n = 44). DMM enterotyping of the cohort resulted concordantly with previous results, in three dominant groups *Bacteroides* (K1), *Firmicutes* (K2), and *Prevotella* (K3) lineages. The diversity analysis revealed a high level of diversity in the Firmicutes group (K2) both in terms of species richness and evenness (Wilcoxon rank-sum test, p value < 0.05 vs. K1 and K3 groups), consistent with the Ruminococcus enterotype described in Western populations. Additionally, functional enrichment analyses of KEGG modules showed significant differences in abundance between individuals with T2DM and controls (FDR < 0.05). These differences include modules associated with the degradation of amino acids, such as arginine, the degradation of urea as well as those associated with homoacetogenesis. Prediction analysis with the Predomics approach suggested potential biomarkers for T2DM, including a balance between a depletion of *Enterococcus faecium* and *Blautia* lineages with an enrichment of *Absiella* spp or *Eubacterium limosum* in T2DM individuals, highlighting the potential of metagenomic analysis in predicting predisposition to diabetic cardiomyopathy in T2DM patients.

## Introduction

The incidence of Type 2 Diabetes Mellitus (T2DM) is exponentially rising in both developing and developed countries involving around 537 million adults and causing 6.7 million deaths in 2021^1^. From the Middle East and North Africa perspective, the region has the second-highest rate of worldwide diabetes growth, with a projected 95 million cases by 2030 from the current 73 million cases in 2021 (77% increase)^2,3^. T2DM mellitus is spiraling out of control to become a worldwide public health priority. Traditionally, several risk factors both uncontrollable and controllable have been associated with T2DM and including ethnicity, family history, age, obesity, lifestyle, uncontrol diet, and physical inactivity among others. Interestingly, in the recent decade, a new factor, called gut microbiome dysbiosis, is emerging as a significant contributor to the cause, progress, and outcome of diabetes in humans^4,5^.

The term "dysbiosis" of gut microbiome refers to a persistent and repeated alteration of the microbiome composition with functional alterations in the human gut, both of which have been associated with health issues including several chronic metabolic disorders, including obesity and diabetes^6^. Although there is no universal consensus on what constitutes a control gut microbiome, some hallmarks have been suggested. The gut microbiome constitutes around 100 trillion or more microorganisms (bacteria, fungi, phages) residing in the gastrointestinal tract principally belonging to six phyla: *Firmicutes, Bacteroidetes, Actinobacteria, Proteobacteria, Fusobacteria,* and *Verrucomicrobia*^7^. Of these, *Firmicutes* and *Bacteroidetes* are the most predominant and makeup 90% of the gut microbiome composition. There are more than 4600 diverse species of bacteria distributed differently along the gastrointestinal tract according to recent microbial gene catalogs^8^. The composition of the gut microbial community includes for one-third of the core microbiome, which is relatively stable within the variations due to age, diet, genetics, geography, and lifestyle^9^.

The bulk of recent research has found evidence of an altered gut microbiome in T2DM when compared with controls^10,11^. Currently, data from European, North American, and Asian countries suggest that the gut microbiome of individuals with T2DM exhibits a depletion of butyrate-producing bacteria, sulfate-reducing bacteria, and a reduction in the genera of *Bifidobacterium*, *Bacteroides*, *Faecalibacterium*, *Akkermansia*, and *Roseburia*. On the other hand, these T2DM patients' microbiome profiles were found to contain bacterial species that deplete probiotics, species that degrade mucin, and show an increase in the *Ruminococcus*, *Fusobacteria*, and *Blautia* genera^12,13^. Importantly, antidiabetic medications, notably Metformin have been shown to exert an important effect on the metagenomic profiles of individuals with T2DM^14–16^.

Despite a demonstrable difference in gut microbiome composition between T2DM and controls, reported results to remain inconsistent regarding the microbial diversity and involvement of a specific taxonomic group in the disease. Similarly, the specific molecular mechanisms by which these alterations contribute to the disease pathogenesis remain to be deciphered and it is unclear whether the microbial alteration is the cause or is a consequence of T2DM. Therefore, it is yet unknown if the quantity, quality, and functionality of the gut microbiome matter in T2DM in human hosts.

We believe there is a lack of reporting of gut microbial composition and its alterations among patients from the Middle Eastern regions where obesity and diabetes are highly prevalent with reported rates of 16.3% in the United Arab Emirates, 22% in Saudi Arabia, and 21.1% in Oman^17^. We previously reported that there was a distinct difference between the microbiome compositions of T2DM and non-T2DM controls in a study comprising 50 native Emirati people. *Bacteroides*-2 and *Ruminococcus* enterotypes were more prevalent in patients with T2DM compared with controls based on 16S amplicon sequencing. The latter were enriched with the *Prevotella* enterotype, even though we were unable to detect any significant difference in microbial diversity between the disease and control^18^.

Here, we evaluated the abundance (quantity) and the diversity of microbiome (quality) in stool samples of a new cohort composed of 84 individuals from the United Arab Emirates with or without T2DM using nanopore metagenomic sequencing. The first objective was to study the functional pathways for the abundances of genes annotated with the KEGG ortholog groups (KOs) to examine not only composition but also functional potential in T2DM cases and controls. The second objective was to build interpretable predictive models of T2DM status based on the abundance of bacterial species in order to uncover the most influential species affecting the disease's state.

## Material and methods

### Sample collection and bacterial DNA extraction

A total of 84 stool samples were collected from patients prospectively recruited to the endocrinology clinic at University Hospital Sharjah (Sharjah, UAE), 40 patients with T2DM mellitus, and 44 controls. All participants gave written informed consent and were Basic demographic data such age, gender, marital status, level of education, diet, height, and weight are recorded. The volunteers were excluded if they had experienced liquid (diarrheal) stools, had taken antibiotics, or had been prescribed probiotics during the previous three months. Samples were stored immediately at − 80 °C. Bacterial DNA extraction was performed using Auto-Pure 96 Nucleic Acid Purification System, with “NucleoMag DNA Microbiome Kit” (Macherey–Nagel, Paris, France). The bacterial wall lysis was performed according to the optimized protocol detailed in^19^. DNA yield was evaluated by a fluorometer, Qubit (Life Technologies Alfortville, France), and DNA quality was evaluated by Nanodrop (Thermo Scientific, Alfortville, France).

### Library preparation and sequencing

A total of 1.5 µg of DNA was used to perform PCR-free library construction. DNA end repair was performed using the NEBNext FFPE Repair Mix (New England Biolabs (NEB), Evry, France). We used NEBNext Ultra II End Repair/dA-Tailing Module (NEB) for the “end prep” step, 1D Native barcoding genomic DNA kit (Oxford Nanopore Technologies (ONT)), and “NEB Blunt/TA Ligase Master Mix kit (NEB) for DNA multiplexing and adapters ligation. Agentcourt AMPure XP (Beckman Coulter, Villepinte, France) beads were used for DNA purification. Whole genome metagenomic sequencing was performed with a MinION sequencer (ONT) using 72h runs and 12 samples per run.

### Bioinformatics analyses

Samples were sequenced on nine Nanopore runs (maximum 12 samples per run), generating 16,292,504 reads (1,810,278 reads per run on average) of an average length of 1.1 kb. 20 samples were resequenced > 1 time due to insufficient sequencing depth in the first try. These samples were individually processed until raw species abundance tables reconstruction and collapsed by summing raw abundances before downsizing and normalization steps. Reads were base-called, quality filtered, and demultiplexed with Guppy (Version 2.1.3) with default parameters. After demultiplexing and quality filtering, 12,241,582 of the reads (75.13% of the total) were assigned to biological samples (115,487 reads per sample on average). Reads were processed as described previously^19^, with a two-step taxonomic binning procedure of Nanopore reads. In the first step, Centrifuge tool version 4.0 with default parameters^20^ was used to classify nanopore reads against a comprehensive database of 4644 species-level non-redundant prokaryotic genomes of the Unified Human Genome catalog 1.0^8^ plus the human genome, which allows removing host contaminants (Supplemental Table 1). In a second step, individual read bins were aligned against the corresponding reference genomes with Minimap2 version 2.2^21^, retaining read-taxonomic assignments with a minimum alignment quality (MapQ score) of 5 against the corresponding reference genome, a threshold fixed based on simulation experiments^19^. No significant differences were observed in terms of microbiome diversity (species richness, Shannon diversity) nor microbiome composition across sequencing runs (Supplemental Fig. 1A–C), but we observed significant differences in microbiome diversity between the 20 samples that were re-sequenced and the rest of samples (Supplemental Fig. 1D,E) and a higher impact even if non-significant on microbiome composition (Supplemental Fig. 1F; PERMANOVA p-value = 0.073). As consequence, all analyses presented in the manuscript were adjusted by the resequencing status of the samples.

**Figure 1:**
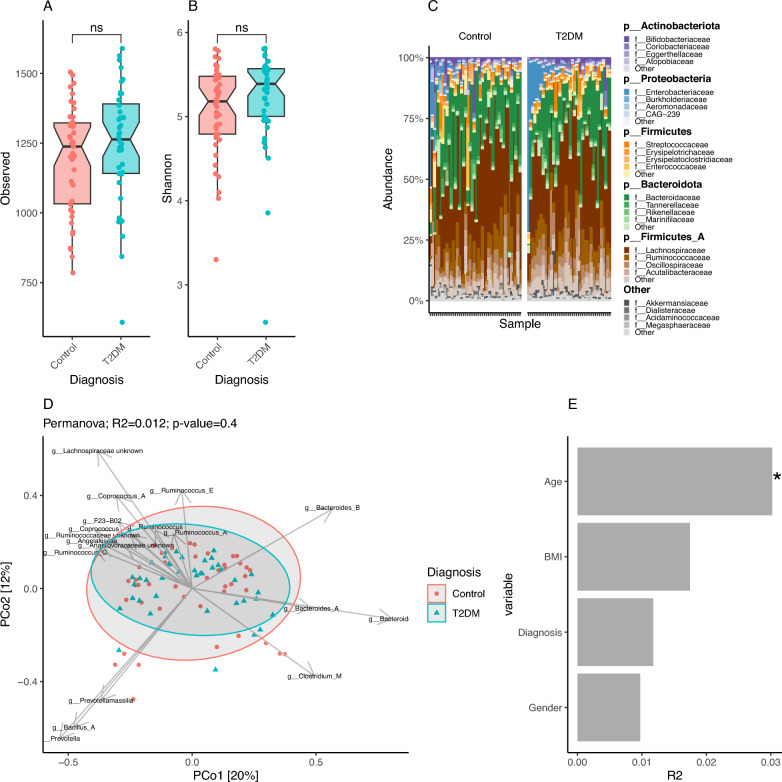
Microbial diversity, taxonomic and compositional profiling of the study cohort. Differences in species richness (**A**) and evenness (**B**, Shannon index) between Control (n = 43- and T2DM (n = 40) individuals (ns = p-value < 0.05; Wilcoxon rank-sum test). (**C**) Taxonomic profile of the study cohort based on phylum-level annotations derived from UHGC1.0 database. For the 4 most abundance phylums, the top 4 family features are visualized. Others bin groups phylum with relative abundances < 2%. Samples are ordered by the relative abudance of Enterobacteriaceae family. (**D**) PCoA ordination based on Bray–Curtis beta-diversity matrix computed from genus-level abundance data. Samples are colored based on the clinical study group. Abundance vectors of 18 bacterial genera with the most significant associations with the k = 3 enterotypes (FDR < 0.05; Kruskal–Wallis test; Supplemental Fig. 2) are fitted on the ordination plot with envfit function of the vegan R package. (**E**) Impact of different covariates over microbiome composition of the study cohort. Barplot represents the effect sizes (R2, x-axis) product of Permanova tests assessing the marginal effects of each covariate in the x-axis over Bray–Curtis beta-diversity matrix computed from genus-level abundance data (each covariate analyzed in a model with all other covariates plus the resequencing status of the sample; adonis2 function of vegan package; *p-value < 0.05).

Species-level abundance tables were built from taxonomic binning results and were integrated with taxonomic information and sample metadata in phyloseq R objects^22^ for subsequent ecological analyses in R (version 3.6.2). Species abundance tables were downsized to 20K reads per sample to control for variations in raw sequencing depth across samples (Supplemental Table 1) followed by normalization by genome length and transformation to relative abundances (RPKM). Alpha diversity (Observed species, Shannon) was computed from the rarified species abundance table at 10 different thresholds (from 10 to 100K counts per sample at steps of 10K counts) with the *estimate richness* function of phyloseq R package^22^. An upsizing procedure was subsequently applied with the *momr* R package^23^ to fit distributions of correlations between different downsized levels and “predict” values for the samples not reaching a given rarefaction threshold. Diversity values in the manuscript were reported at the 20K rarefaction threshold.

**Table 1:** Clinical differences between study groups.

	Control	T2DM	p.overall
	*N* = *44*	*N* = *40*	
Age	44.5 [34.0;54.0]	68.5 [61.8;77.2]	< 0.001
Gender			0.044
Woman	19 (43.2%)	27 (67.5%)	
Men	25 (56.8%)	13 (32.5%)	
BMI	24.9 [22.9;28.6]	29.5 [26.3;34.6]	0.001

Differences in age and BMI were evaluated with Wilcoxon rank-sum tests, whereas differences in gender were evaluated with Chi-square test.

The vegan R package^24^ vegan: Community Ecology package version 2.6-2) was used to compute Bray–Curtis beta-diversity matrices from the same rarefied species abundance table collapsed at the genus level (*vegdist* function), and to visualize microbiome similarities with Principal Coordinate analysis (PCoA) (*cmdscale* function). Environmental fitting of genus abundance vectors over PCoA ordination from Bray–Curtis inter-sample dissimilarity matrix was computed with the *envfit* function of the *vegan* R package. Enterotype classifications were performed from the genus abundance matrix using the Dirichlet Multinomial Mixture (DMM) method as described^25^ and implemented in the Dirichlet Multinomial R package. (DirichletMultinomial: Dirichlet-Multinomial Mixture Model Machine Learning for Microbiome Data. R package version 1.28.0). The abundance of KEGG ortholog groups (KO groups) was quantified from the functional annotations of UHGC1.0 genomes, from which the abundance of KO was computed as the sum of the abundances of the species containing these KO groups (20K downsized RKPM abundance tables)^26^.

### Prediction of T2DM status from species abundances

*Predomics* R package version 1.01^27^ was used to build interpretable predictive models of T2DM status based on the abundance of bacterial species (20K downsized RKPM abundance tables). Only features (species) with 20% prevalence across control and T2DM groups were used for prediction tasks (1787 features). Models were trained on 26 different algorithms including GLMNET, Random Forest, and Support Vector Machine (SVM) as state-of-the-art (SOTA) methods, and BTR (Bin/Ter/Ratio) native *predomics* models describing simple ecological relationships in microbial ecosystems that were learned with five different heuristics (Terga1/Terga2/Terbeam/Terda/Metal). Details of the different heuristics and BTR models can be found in^27^. Models were evaluated for accuracy and AUC on a 10 times tenfold cross-validation schema, and the results of the best algorithm were further explored to extract a family of best models (FBM), described as models whose accuracy is within a given window of the best model’s accuracy. This window is defined by computing a significance threshold assuming that accuracy follows a binomial distribution (p < 0.05). No hyperparameter optimization was performed. Features included in the FBM were further explored in terms of prevalence across models and feature importance, described as the mean decrease accuracy (MDA) of the model after feature removal.

### Statistical analyses

Linear regression analyses were used to evaluate the impact of different clinical variables (age, gender, BMI), sequencing covariates (resequencing status of the samples) and disease state over alpha diversity distributions (Observed Species, Shannon index). The significance of alpha diversity changes between study groups was tested with non-parametric Wilcoxon rank-sum tests over the original diversity values and over the residuals of linear regression analyses of log-transformed alpha diversity (dependent variable) vs. age and age + gender + BMI + resequencing status (independent variables). Dissimilarity in community structure by disease state and other covariates (age, gender, and BMI controlling for resequencing status) was assessed with permutational multivariate analyses of variance (PERMANOVA) assessing the marginal effects of each variable over the Bray–Curtis beta-diversity with *adonis2* function of *vegan* package. vegan: Community Ecology Package. R package version 2.6-2). Differences in enterotype composition across study groups were evaluated with Chi-Square tests. To identify metagenomic features (species and KO groups) associated with disease state while accounting for sequencing depth and the confounding effect of age and resequencing status linear regression models were fitted with log-transformed feature abundance (20K downsized RKPM abundance tables) as the dependent variable and disease state, age and resequencing status as dependent variables with *lm* function of base R. Based on the results of the linear regression analyses on KO abundance tables, functional enrichment analyses of KEGG modules and Gut Metabolic Modules (GMMs)^28^ were carried out to identify high-order functional features associated with T2DM transition using the KO-associated P-values (p-value < 0.05) and the corresponding beta coefficients of the linear regression analyses between controls and T2DM as effect sizes using the Reporter Feature algorithm as implemented in the Piano R package v.2.2.0^29^. The null distribution was used as the significate method and P-values were adjusted for multiple comparisons with the Benjamini–Hochberg method. All analyses were conducted in the R environment (version 3.6.2).

### Ethics approval and consent to participate

The study was performed after receiving ethical approval from University Hospital Sharjah Ethics Research Committee (UHS-HERC-021-0702). All the experimental protocols for involving human data followed the Declaration of Helsinki and were approved by the competent Research Ethics Committee of the University of Sharjah.

### Consent for publication

Informed written consent was obtained from all subjects.

## Results

### Diversity and compositional analyses

Regarding clinical differences (Table 1), T2DM individuals (mean 69.6 (± 12.3 sd) years) were significantly (*p* value < 0.01; Wilcoxon rank-sum test) older than the controls (46.9 (± 17.3 sd) years), showed higher BMI (mean 30.9 (± 7.01 sd) vs. mean 26.3 (± 6.12 sd) than the control group; *p* value = 0.001; Wilcoxon rank-sum test) and were enriched in women (67.5% vs. 43.2% in the control group; *p* value = 0.044, Chi-square test). Non-significant differences in microbial diversity were observed between groups in terms of species richness (Fig. 1A) and evenness (Fig. 1B). Permanova, and linear regression analyses of individual covariates (age, BMI, gender) showed no significant association of any of them with microbial diversity (*p* value > 0.05) (Fig. 1D,E). The differences in diversity between study groups stayed consistent when correcting for additional confounders (age, gender, BMI and resequencing status) (Supplementary Fig. 1).

The taxonomic profile showed as expected the dominance of lineages from the *Firmicutes* and *Bacteroidota* phyla, with some individuals enriched in *Proteobacterial* lineages (Fig. 1C). Stratification of the cohort around 3 discrete microbiome compositions by the DMM approach^25^, from genus-level abundance table, showed two main groups of individuals dominated by *Bacteroides* (K1 group; n = 46 individuals) and *Firmicutes* lineages (K2 group; n = 34 individuals), followed by a small group of 4 individuals (K3) enriched in *Prevotella* lineages (Supplemental Fig. 1A–C). Diversity distributions across the three enterotypes show a high diversity profile of the K2 group both in terms of species richness and evenness (Supplemental Fig. 1D,E; *p* value < 0.05 vs. K1 and K3 groups; Wilcoxon rank-sum test) that is in line with the classic *Ruminococcus* enterotype^30,31^. No significant differences in species richness were observed between the K1 (*Bacteroides*-enriched) and K3 (*Prevotella*-enriched) groups (Supplemental Fig. 1D, p value > 0.05), with the *Prevotella* group showing significantly lower levels of evenness (Shannon index; Supplemental Fig. 1E; *p* value < 0.05).

A decomposition of the human gut microbiome into 4 community types based on the same DMM approach has been observed in several cohorts^32–34^, which includes a dysbiotic composition named *Bacteroides2* associated with systemic inflammation and characterized by low levels of microbial diversity and microbial cell loads. The decomposition of the cohort at K = 4 groups, even if less supported in terms of the Laplace metric derived from DMM enterotyping (Supplemental Fig. 1A) shows a potential *Bacteroides2* group (K4) composed of 4 individuals derived from the *Bacteroides*-enriched composition (Supplemental Fig. 1B,F). This shows a dysbiotic microbiome profile in terms of low species richness (Supplemental Fig. 1G) and evenness (Supplemental Fig. 1H). Importantly, in this population, no significant differences in enterotype composition between T2DM and control groups were observed in neither enterotyping approach, with three (Supplementary Fig. 1I; *p* value = 0.087, Chi-square test) or four groups (Supplementary Fig. 1J; *p* value = 0.28, Chi-square test). Only a tendency towards the enrichment of the high-diversity composition (K2 enterotype) is observed in T2DM, in line with a trend toward high diversity profile.

Permutational Analyses of Variance (PERMANOVA) over microbiome composition (Bray–Curtis beta-diversity matrix derived from genus-level abundance data) controlling for other covariates (BMI, Age, gender and resequencing status) showed no impact of disease state (R2 = 0.01, *p* value = 0.52), with only age showing a significant association with microbiome composition (R2 = 0.028, *p* value = 0.005) (Supplementary Fig. 3B).

### Age-adjusted univariate associations of taxonomic and functional features with T2DM

**Figure 2:**
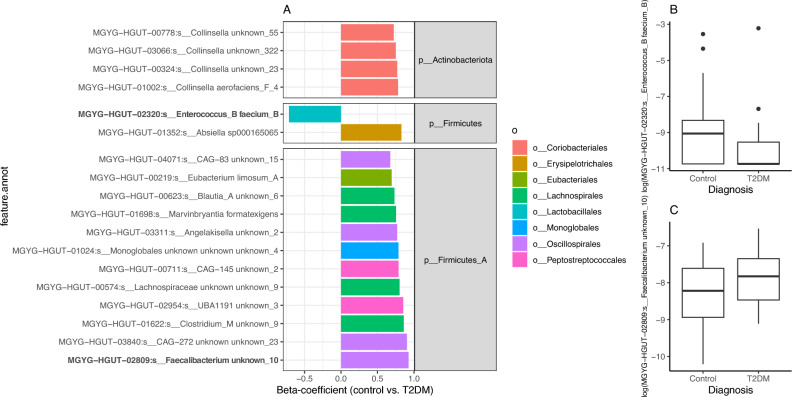
Species features with significant differences in abundance between T2DM and Control groups in the study cohort. (**A**) Barplot of beta coefficients in the abundance of 18 bacterial species with strongest significant differences in abundances between the Control and T2DM group (p-value < 0.01; linear regression model with log-transformed species abundances by disease state adjusted by age and resequencing status of the samples; full results in Supplemental Table 2). (**B**,**C**) Boxplots of log-transformed abundances of 2 bacterial species in bold y-axis of barplot of panel A illustrating the direction of abundance changes between study groups.

We next searched for taxonomic features significantly different between non-T2DM and T2DM groups while accounting for the confounding effect of age (*e.g.* significant impact on microbiome composition in PERMANOVA analyses) by fitting linear regression models of log-transformed species abundance (20K RPKM abundances) by disease, age and resequencing status. Significant differences in abundance were observed for 105 bacterial species between both groups, of which 10 were significantly increased in the control group and 95 were significantly increased in the T2DM group (p-value < 0.05; Supplemental Table 2). None of these features resist adjustment for multiple comparisons (FDR > 0.05). Focusing on the species with the strongest differences at p-value level (p-value < 0.01) 17 species showed significantly higher abundance in T2DM (avg. ± std. error of beta coefficients in linear regression analyses vs. study group (control as reference level) adjusted by age and resequencing status = 0.78 ± 0.068), interestingly characterized by the dominance of Firmicutes lineages of the group A phylum including MGYG-HGUT-02809:s_*Faecalibacterium,* MGYG-HGUT-01698:*s*_*Marvinbryantia formatexigens,* MGYG-HGUT-00219:s_Eubacterium limosum_A or MGYG-HGUT-01352:*s*_*Absiella sp000165065* (Fig. 2A,C). On the other hand, MGYG-HGUT-02320:*s*_*Enterococcus_B faecium_B* was the species with the most significant increases in the control group (Fig. 2A,B). Exploring the other species with significant differences at p-value < 0.05 level, Bacteroides lineages including *MGYG-HGUT-02300: s*_*Bacteroides cutis* were also enriched in the control group (Supplemental Table 2).

**Figure 3:**
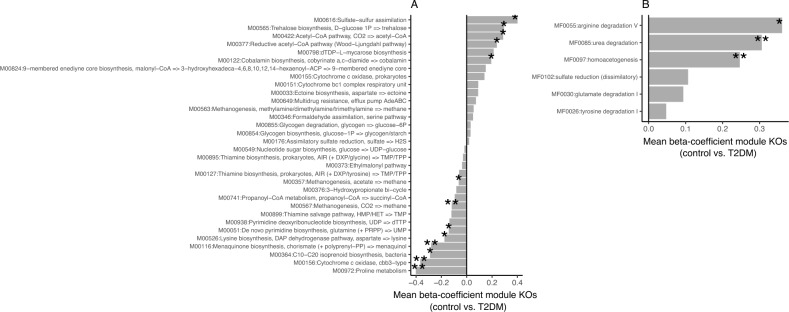
Functional features with significant differences between Control and T2DM groups in the study cohort. (**A**) 32 KEGG modules significantly enriched in differentially abundant KO groups between Control and T2DM groups (** FDR < 0.05, *P value < 0.05; Gene Set Enrichment Analyses). The mean beta coefficients of module KOs abundances between control and T2DM individuals are represented as an indicator of enrichment direction (linear regression of log-transformed KO abundances by disease state adjusted by age and resequencing status; modules enriched in the T2DM group = mean beta coefficients of KO groups in Controls vs. T2DM > 0; modules enriched in the Control group = mean beta coefficients of KO groups in Controls vs. T2DM < 0). (**B**) Same as (**A**) for 6 Gut Metabolic Modules (GMMs) significantly enriched in differentially abundant KO groups between Control and T2DM groups (**FDR < 0.05; Gene Set Enrichment Analyses).

Similar analyses at the functional level based on the abundance of KEGG orthology groups (KO groups) identified 174 KOs with significant differences in abundance between T2DM and control groups (p-value < 0.05). Of which, 65 were increased in controls (avg. ± std. error of beta coefficients in linear regression analyses of KO abundances vs. study group (control as reference level) adjusted by age and resequencing status = − 0.605 ± 0.07) whereas 109 were increased in the T2DM group (avg. ± std. error of beta coefficients in linear regression analyses of KO abundances vs. study group (control as reference level) adjusted by age and resequencing status = − 0.607 ± 0.07). None of these KO groups resist adjustment for multiple comparisons (FDR > 0.05). We then used gene set enrichment analyses^29^ of KEGG functional modules with p-values from linear regression analyses of KO abundances vs. study group (control as reference level) adjusted by age and resequencing status and the corresponding beta-coefficients as indicators of effect size. We identified 32 KEGG modules significantly enriched in KO groups differentially abundant in both study groups (*p* value < 0.05), of which only 8 showed significant direct enrichment in KO groups with higher abundance in the control group (p-value < 0.05 in linear regression models of log-transformed KO abundance by disease state, age and resequencing status + beta-coefficient < 0 in control vs. T2DM group; Fig. 3A) and 5 showed significant enrichment in KO groups with direct higher abundance in the T2DM group (p-value < 0.05 in linear regression models of log-transformed KO abundance by disease state, age and re-sequencing status + beta coefficients > 0 control vs. T2DM group; Fig. 3A).

Interestingly, among the modules enriched in T2DM, we observed modules associated with sulphate reduction (M00616), which has also been previously associated with T2DM^16^, as well as modules associated to acetate production like M00422:Acetyl-CoA pathway, CO_2_ ⟹ acetyl-CoA and M00377:Reductive acetyl-CoA pathway (Wood–Ljungdahl pathway) (PMID: 37432351, PMID: 32126162) and modules for cobalamin production (Fig. 3A). In contrast, the control group was significantly enriched in modules involved in the metabolism of different aminoacids (M00972: Proline metabolism, M00526: Lysine biosynthesis, DAP dehydrogenase pathway, aspartate ⟹ lysine), modules involved in methanogenesis (M00567: Methanogenesis, CO_2_ ⟹ methane and M00357: Methanogenesis, acetate ⟹ methane) as well as modules involved in propionate production (M00741: Propanoyl-CoA metabolism, propanoyl-CoA ⟹ succinyl-CoA). Similar gene set enrichment analyses over Gut Metabolic Modules (GMMs) showed an enrichment of arginine, and urea degradation as well as an increased acetogenic potential of the microbiome in the T2DM group, in line with results observed in the KEGG module space (Fig. 3B).

### Prediction of T2DM state from taxonomic abundances and prevalence

We next explored the predictive capacity of the metagenomic data to predict the T2DM status using a suite of machine learning (ML) algorithms implemented in the Predomics R package^27^, developed in-house. This includes both state-of-the-art-methods (SOTA), including random forest, GLMNET, and support vector machines (SVM) as well as the ecosystem-inspired BTR models (Bin/Ter/Ratio) that are learned using different heuristics (metal, terbeam, terda, terga1, terga2).

**Figure 4:**
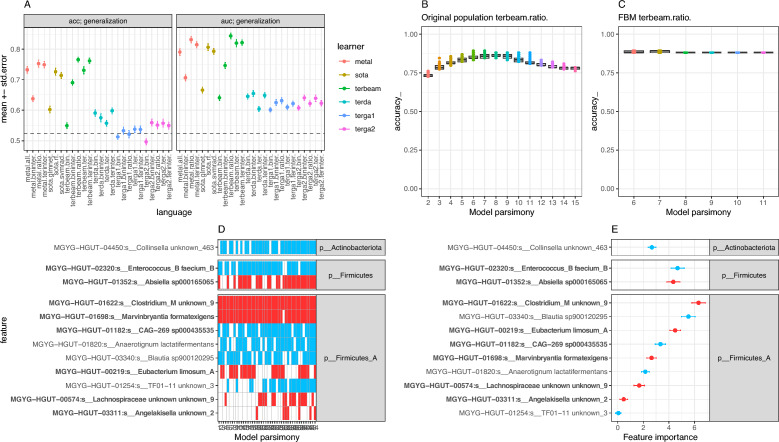
Prediction of T2DM state from taxonomic abundances based on Predomics models. (**A**) mean ± standard error of accuracy (acc) and AUC of T2DM predictions of best models based on 26 different learners integrated into the Predomics package from 10 times tenfold cross-validation schema. The dashed line represents the majority class (i.e., the accuracy obtained when simply predicting the T2DM status through chance alone; 0.52). Predictions from terbeam learner and ratio language show the best performance in comparison with other BTR models. (**B**) Boxplots of the accuracy (y-axis) of terbeam-ratio models (n = 1316) at different model sparsities (number of features per model; x-axis). (**C**) Same as the B panel for the Family of Best Models (FBM; n = 44 terbeam-ratio models whose accuracy is within a given window of the best model’s accuracy). (**D**) Heatmap representing the prevalence of the 12 bacterial species (y-axis) included in the 44 models in the FBM (red = presence; white = absence). (**E**) Mean ± standard error of feature importance variable (decrease accuracy when the feature is removed in cross-validation process) for the 12 bacterial species included in the terbeam-ratio FBM (red = high mean abundance in the T2DM group; blue = High mean abundance in the control group). Species overlapping with the ones showing significant changes in differential abundance analyses (p-value < 0.05; linear regression model with log-transformed species abundances by disease state adjusted by age and resequencing status of the samples; Fig. 3 and Supplemental Table 2) are highlighted in bold.

Twenty-six different prediction methods were explored with a 10 times tenfold cross-validation schema based on species abundance data, which showed ratio models generated with the terbeam heuristic as the ones with the best performance in terms of cross-validation accuracy (mean 0.76 ± 8.4e−03 se) and AUC (mean 0.84 ± 9.54e−03 se) (Fig. 4A). In these types of models, the ratio between the sum of two groups of species has been selected that, if above a given threshold learned by the model, predicts the individual as the T2DM class. A total of 1316 models were retained by the terbeam heuristic with a degree of sparsity in terms of the number of species ranging from 2 to 15 species (Fig. 4B). Among them, a Family of Best Models (FBM) consisting of 44 models with statistically non different accuracy levels (see methods) were identified (Fig. 4C). These models included 12 different bacterial species, among which we observed *MGYG-HGUT-01352:s__Absiella sp000165065*, *MGYG*-*HGUT*-*01622:s__Clostridium_M unknown_9*, *MGYG*-*HGUT-01698:s__Marvinbryantia formatexigens* and *MGYG-HGUT-00219:s__Eubacterium limosum_A,* all enriched in the T2DM group and species like *MGYG*-*HGUT*-*02320:s__Enterococcus_B faecium_B*, enriched in the control group, being all these species highly prevalent across these 44 models (Fig. 4D), suggesting that they are key species in terms of the predictive capability of these models. This was also confirmed by the feature importance values of these species defined as the mean decrease accuracy (Fig. 4E). Importantly, despite the fact that Predomics algorithm don’t allow adjustment of the predictive models by cofounding factors, 8/12 species retained in the FBM (bold in Fig. 4D,E) were also included among the significant ones resulting from the differential abundance analyses adjusted by age and resequencing status (p-value < 0.05, Supplemental Table 2).

**Figure 5:**
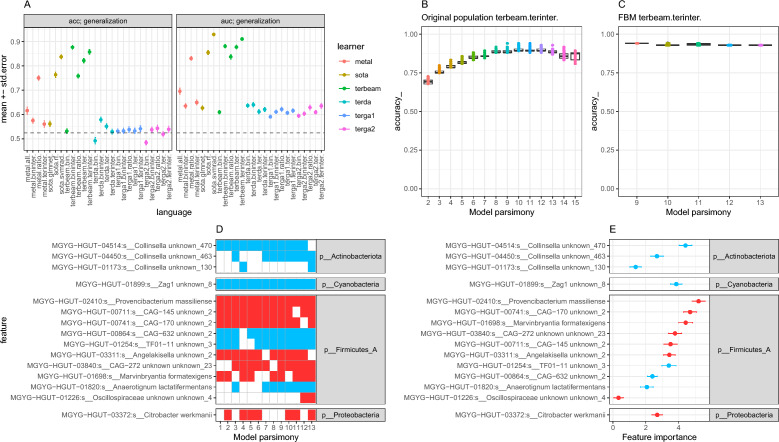
Prediction of T2DM state from taxonomic prevalence based on Predomics models. (**A**) mean ± standard error of accuracy (acc) and AUC of T2DM predictions of best models based on 26 different learners integrated into the Predomics package from 10 times tenfold cross-validation schema from species presence/absence data. The dashed line represents the majority class (i.e., the accuracy obtained when simply predicting the T2DM status through chance alone; 0.52). Predictions from terbeam learner and terinter language show the best performance in comparison with other BTR models considering AUC and accuracy (acc). (**B**) Boxplots of the accuracy (y-axis) of terbeam-terinter models (n = 1316) at different model sparsities (number of features per model; x-axis). (**C**) Same as the B panel for the Family of Best Models (FBM; n = 13 terbeam-terinter models whose accuracy is within a given window of the best model’s accuracy). (**D**) Heatmap representing the prevalence of the 14 bacterial species (y-axis) included in the 13 models in the FBM (red = presence and more prevalent in T2DM group; blue = presence and more prevalent in control group; white = absence). (**E**) Mean ± standard error of feature importance variable (decrease accuracy when the feature is removed in cross-validation process) for the 14 bacterial species included in the terbeam-terinter FBM (red = high mean prevalence in the T2DM group; blue = High mean prevalence in the control group).

Similar analyses with presence/absence data showed that ternary models learned with the terbeam learner were the ones showing the best performance among Predomics BTR models (Fig. 5A; mean AUC = 0.91 ± 6.96e−03 se; mean accuracy = 0.85 ± 9.7e−03). In these models is the difference between two combinations of species that defines the classification of a sample as control or T2DM. A total of 1316 models of this type were retained by the terbeam heuristic (Fig. 5B), 13 of them were included in the FBM (Fig. 5C). Among the species retained in these models, MGYG-HGUT-01698: s__Marvinbryantia formatexigens (enriched in T2DM group) and MGYG-HGUT-01820: s__Anaerotignum lactatifermentans (enriched in control group) overlaps with the results of the models trained on species abundances, and emerged a proteobacterial lineage enriched in the T2DM group (MGYG-HGUT-03372: s__*Citrobacter werkmanii* ) important in terms of feature prevalence (Fig. 5D) and feature importance (Fig. 5E). A significant positive correlation was observed between the feature importances of species retained in the FBM of Predomics based on presence/absence data vs. mean decrease Gini of the best Random Forest model (Supplemental Fig. 4; Spearman rho = 0.6, pvalue = 0.02), supporting the important features retained by Predomics algorithms by SOTA method (Random Forest).

## Discussion

We expanded upon a previous pilot study by utilizing the novel long-read nanopore sequencing technology to characterize the taxonomic and functional profiles of gut microbiomes in both individuals with T2DM and controls from the University Hospital Sharjah^18^. We observed a non-significant difference in microbial diversity between T2DM and control groups in terms of species richness and evenness after controlling for age, gender, and BMI using the Observed Species and Shannon estimator indexes. The high-throughput sequencing technology has indicated a significant decrease in the species richness (the number of organisms of one type of microbe) and diversity (of different microbe types) in the majority of T2DM cases estimated using indices such as Chao1, Shannon, and Simpson^35,36^. Concurrently, when compared to controls, T2DM patients have been also shown to have opposite findings of an increase^37,38^ or a non-significant correlation^39,40^ in microbial diversity using similar nonparametric estimating indices.

When we examine the compositional difference (quality) in the gut microbiome between T2DM and controls, we observed that phyla *Firmicutes* and *Bacteroidetes* were predominant in both the T2DM and control groups. The gut microbiome of T2DM patients had more representations of the phylum *Proteobacteria*–genus *Klebsiella*, phylum *Bacteroidetes*–genus *Prevotella* and phylum *Firmicutes*–genus *Clostridium*. In contrast, the controls had predominance lineages of genus *Bacteroides*–phylum *Bacteroidetes* and genus *Blautia*–phylum *Firmicutes*. Even if we observed non-significant differences in enterotype composition between controls and T2DM individuals, there are common compositional patterns with our previous study^41^. These include T2DM individuals having a higher proportion of *Ruminococcus*-enriched enterotype and the control group having a higher proportion of *Prevotella*-enriched enterotype.

The involvement of specific taxonomic groups in T2DM also varies across studies. Different taxa are indeed reported to be associated both positively and negatively with T2DM in different studies^7,10^. For example, the phyla *Firmicutes, Bacteroidetes,* and the ratio (*Bacteroidetes*/*Firmicutes*) have opposite representations in T2DM patients^36,42–44^. Even within the same phylum *Firmicutes* and class *Clostridia* have an opposite representation in T2DM patients when compared to the controls in two different studies^40,42^. These inconsistencies became more variable as we moved in the lineage from phylum to species level. Some species from the *Clostridium* and *Lactobacillus* genera displayed different abundance profiles in the disease state and controls^10,42^. Here, three bacterial species: *Enterococcus faecium*, *Absiella spp*., and *Eubacterium limosum* were identified as strong predictors of T2DM by the Predomics approach. There were prevalent in the family of 44 best models and also among the most important features in the out-of-bag perturbations^45^. Noteworthy, while *Eubacterium limosum* produces short-chain fatty acids^46^, *Absiella innocuum.* is opportunistic pathogen that are found in the altered gut microbiome and attenuate chronic inflammatory processes^47^, both of them enriched in the T2DM group. In contrast, *E. faecium*, enriched in the control group, is a common human gut commensal (PMID: 27165538), with different strains that has shown lipid-lowering effect on rats with hypercholesterolemia (10.1007/s00217-008-0932-9), anti-obesity effect in HFD-fed mice (PMID: 25089585) and, in combination with *Bacillus subtilis*, prevent obesity-associated hyperlipidemia and modulates gut microbiota in mice (PMID: 33144552). Next, we studied the potential activity (functionality) of the gut microbiome to derive any significant difference between the disease state and controls. There was a distinction in the functional profile of the gut microbiome between T2DM and non-T2DM groups observed in the KEGG module space. An enrichment of the potential for arginine and urea degradation, sulphate reduction and acetate production through homoacetogenic bacteria was observed in individuals with T2DM. These enhanced functional contributions have suggested pro-inflammatory activities that may result in chronic low-grade inflammation the hallmark of T2DM^7,48^. Notably, the increase of the homoacetogenic potential of the microbiome in T2DM individuals could be linked to the increase in the abundances of *Marvibryantia formatexigens*, which has been described as fermenting glucose to acetate in the presence of high formate concentrations (PMID: 14532100). This increase in the acetogenic potential of the microbiome in the T2DM group is noteworthy given their important regulatory role in body weight control and insulin sensitivity through effects on lipid metabolism and glucose homeostasis^49^ and references therein. Microbial-derived acetate production is consequence of the fermentation of indigestible foods especially foods of acetogenic fibers like galacto-ligosaccharides or inulin^50^, so nutritional differences could explain this enrichment in the T2DM group.

Our study expands upon a previous pilot study, which investigated the gut microbiome of a population in the United Arab Emirates. This cohort shared several commonalities, such as geography, climate, lifestyle, food, race, and culture, that are known to influence gut microbiome composition, making it an important group to study^51,52^. Additionally, the design of the study which we tried to maintain the methods as close as possible to the earlier study protocol in terms of sampling of stool specimens, DNA extraction protocol, sequencing, bioinformatics interpretations, depth of analysis, and prediction models. The study has limitations such as the confounding factor of age differences between study groups, which were accounted for in univariate statistical analyses, medication use such as Metformin, and a small sample size that may explain some discrepancies with findings from larger cohorts. Moreover, using KEGG module calculation, we have an estimate of the functional potential based on metagenomic sequencing. We also acknowledge prediction analysis limitation, a more functional approach such as metatranscriptomics and metaproteomics could be envisaged in the future.

In conclusion, our study aimed to address the functional potential of the gut microbiome in individuals with T2DM in the UAE. First, we found that the gut microbiome of T2DM patients exhibited changes in composition compared to controls, with an increase in opportunistic pathogens such as *Prevotella,* proteobacterial lineages*,* and *Clostridium*, and a decrease in beneficial bacteria like *Desulfovibrio* and *Bacteroides*. These results align with previous studies that have identified dysbiotic gut microbiome in T2DM. Second, we discovered that T2DM patients had distinct gut microbiome functionality potential, characterized by enriched modules associated with the degradation of amino acids, and urea and notably an increased acetogenic potential of the microbiome potentially explained by the increase of acetogenic lineages like *Marvibryantia formatexigens*.

### Supplementary Information

Supplementary Information.

## Data Availability

Gene sequencing data used for this study were submitted to the European Nucleotide Archive (ENA) and are available under accession number ERA20716515 (private access until paper acceptance). Additional data supporting this study's findings are available on request from the corresponding author.
