# Supplementary material for: Functional alterations and predictive capacity of gut microbiome in type 2 diabetes
Source: Sci Rep. 2023 Dec 16;13:22386. doi: 10.1038/s41598-023-49679-w (PMC10725451; doi:10.1038/s41598-023-49679-w)
Supplement: Supplementary file 1 — Supplementary Information. [file 41598_2023_49679_MOESM1_ESM.pdf]

Functional Alterations and Predictive Capacity of Gut Microbiome in Type 2 Diabetes

Nihar Ranjan Dash[1†], Mohammad T. Al Bataineh [2,3†*], Rohia Alili[4,5], Habiba Al Safar[3], Noura Alkhayyal[6], Edi Prifti[4,7], Jean-Daniel Zucker[4,7], Eugeni Belda[4,7], Karine Clément[4,5*]

Supplementary information

Supplemental Table 1. Summary reads for each sample with additional metadata

Code	quality filtered reads	human Reads	quality filtered + human decontaminated reads	Age	Gender	BMI	Diagnosis	run	resequenced
1240	253107	1511	251596	65	M	24.540394 67	Control	run_202,run_204	Yes
5017	130858	909	129949	42	F	27.5	Control	run_202	No
5230	342590	1288	341302	36	M	40.258	Control	run_206	No
7701	87134	561	86573	72	F	31.2	T2DM	run_205	No
8425	263836	1532	262304	70	M	16.806134 24	Control	run_206	No
9559	82911	453	82458	65	M	28.003	Control	run_208	No
11911	89414	352	89062	51	M	22.503	Control	run_203,run_207	Yes
12657	99524	859	98665	61	F	35.3704142	T2DM	run_203	No
19166	74590	301	74289	38	F	24.2	Control	run_208	No
20514	111857	443	111414	70	M	26.5625	T2DM	run_203	No
25449	164377	725	163652	51	M	27.940220 92	Control	run_200,run_204	Yes
29879	66603	9063	57540	79	M	39.556	T2DM	run_201,run_207	Yes
30424	229783	669	229114	65	F	28.696329 64	T2DM	run_204	No
30885	58710	149	58561	76	F	28.040378 14	T2DM	run_202,run_204	Yes
34669	202098	1101	200997	55	F	26.787	Control	run_206	No
36909	71834	230	71604	91	F	49.822485 21	T2DM	run_203	No

36920	122446	513	121933	85	F	27.303438 38	T2DM	run_206	No
37732	274825	2337	272488	73	F	36.066471 88	T2DM	run_201	No
37828	107639	945	106694	58	M	28.621	T2DM	run_205	No
40437	225074	891	224183	49	F	19.7710718	Control	run_207,run_208	Yes
42380	162652	533	162119	87	F	30.6640625	T2DM	run_203,run_208	Yes
42734	201768	1584	200184	78	F	29.475842 62	T2DM	run_202,run_204	Yes
43608	28706	2329	26377	76	F	36.840489 45	T2DM	run_200	No
47590	116387	1023	115364	86	F	34.159722 58	T2DM	run_201	No
48050	171766	1859	169907	52	M	28.905075 73	Control	run_201	No
49781	110892	399	110493	74	M	24.151662 05	T2DM	run_200	No
49926	102122	478	101644	32	M	16.64	Control	run_203	No
51007	69934	279	69655	36	F	24.956	Control	run_200,run_207	Yes
55459	296327	1815	294512	64	F	19.093433 84	T2DM	run_202,run_204	Yes
57061	71893	281	71612	49	F	23.242728 53	Control	run_203	No
58720	213366	2508	210858	22	M	24.913494 81	Control	run_201	No
62351	71157	319	70838	61	F	48.321	T2DM	run_203	No
63573	129020	87114	41906	82	F	32.255	T2DM	run_205	No

63712	297155	1420	295735	66	F	29.81328579	T2DM	run_200,run_205,run_208	Yes
67621	67275	603	66672	87	F	20.92838722	Control	run_205	No
74317	121737	2111	119626	76	M	30.85960738	T2DM	run_201	No
74872	76646	620	76026	83	M	41.11842105	T2DM	run_208	No
78451	38631	526	38105	34	F	32.46618106	Control	run_207	No
82640	58945	15386	43559	78	M	23.98375407	Control	run_200	No
84277	70022	257	69765	50	F	42.38	T2DM	run_203	No
85791	65547	354	65193	48	M	26.644	Control	run_205	No
86455	153093	519	152574	67	F	25.682	T2DM	run_206	No
87182	92487	677	91810	38	M	25.02	Control	run_205	No
89572	119654	392	119262	23	M	22.985	Control	run_208	No
91183	93994	677	93317	49	M	23.24598478	Control	run_205	No
93865	65486	1323	64163	73	M	23.739	T2DM	run_205	No
94595	6969	50	6919	77	M	21.17732971	Control	run_203,run_207	Yes
95492	114469	2218	112251	68	M	28.48	T2DM	run_201	No
100612	109120	401	108719	67	M	23.85	T2DM	run_208	No
108175	169251	512	168739	35	F	38.408	Control	run_200,run_204	Yes

108945	166642	707	165935	86	F	27.77226741	T2DM	run_202,run_204	Yes
111473	326669	1333	325336	37	M	33.252	Control	run_206	No
113950	162042	4088	157954	37	M	23.64758344	Control	run_201	No
117130	98611	732	97879	31	F	17.926	Control	run_205	No
117402	219986	692	219294	66	M	31.39172336	T2DM	run_202,run_204	Yes
118721	264525	995	263530	62	F	25.39	T2DM	run_206	No
121860	88961	3653	85308	42	M	17.75114478	Control	run_201	No
122185	183541	613	182928	33	M	23.124	Control	run_206	No
124387	95015	445	94570	60	F	35.61128024	T2DM	run_200	No
124812	155684	686	154998	83	F	27.11111111	T2DM	run_207	No
137654	43722	87	43635	77	M	19.39646202	T2DM	run_202	No
139215	219590	663	218927	28	M	22.196	Control	run_206	No
140738	117900	468	117432	54	F	43.91658891	Control	run_203,run_207	Yes
144829	287339	1030	286309	66	F	34.33922997	T2DM	run_202,run_204	Yes
145108	228389	2309	226080	47	F	21.39543264	Control	run_201	No
145501	53614	183	53431	61	F	29.4	T2DM	run_200	No

153133	69141	288	68853	71	F	38.44675125	Control	run_200	No
160518	200411	742	199669	67	M	23.588	T2DM	run_206	No
167490	32563	102	32461	94	M	27.27633406	Control	run_202	No
167941	43018	206	42812	24	M	22.72043837	T2DM	run_200	No
174307	103437	420	103017	69	F	40.44444444	T2DM	run_207,run_208	Yes
177949	46857	12277	34580	59	F	24.4	T2DM	run_203	No
179104	306186	1341	304845	66	M	30.73941662	Control	run_200,run_207,run_208	Yes
179257	239788	891	238897	34	F	29.565	Control	run_206	No
181640	28879	183	28696	23	F	28.516	Control	run_207	No
189102	170913	96860	74053	36	F	24.722	Control	run_205	No
189935	89265	253	89012	56	F	29.6	T2DM	run_208	No
191943	843472	714929	128543	32	F	32.343	Control	run_201	No
192695	133856	1094	132762	59	F	31.01143151	T2DM	run_201	No
194044	31390	2589	28801	54	F	25.6	Control	run_207	No
194086	97774	1945	95829	30	M	23.665	Control	run_205	No
195873	122820	2014	120806	52	M	32	Control	run_208	No
196004	234748	700	234048	50	F	28.3	Control	run_202,run_204	Yes
1251400	209153	777	208376	31	M	19.446	Control	run_206	No

Supplemental Table 2. Full results of linear regression analyses of UHGC species abundances (log-transformed) vs. Study group adjusted by age and re-sequencing status

feature	pvalue	estimate	fdr	d	p	c	o	f	g	s
MGYG-HGUT-00002:s__Blautia_A sp900066165	0.714473939	0.101900576	0.979458475	d__Bacteria	p__Firmicutes_A	c__Clostridia	o__Lachnospirales	f__Lachnospiraceae	g__Blautia_A	s__Blautia_A sp900066165
MGYG-HGUT-00003:s__Alistipes shahii	0.240933643	0.322310795	0.902181367	d__Bacteria	p__Bacteroidota	c__Bacteroidia	o__Bacteroidales	f__Rikenellaceae	g__Alistipes	s__Alistipes shahii
MGYG-HGUT-00004:s__Anaerotruncus colihominis	0.436296255	-0.208838718	0.950806594	d__Bacteria	p__Firmicutes_A	c__Clostridia	o__Oscillospirales	f__Ruminococcaceae	g__Anaerotruncus	s__Anaerotruncus colihominis
MGYG-HGUT-00013:s__Bacteroides unknown_1	0.582585942	-0.153567794	0.975671757	d__Bacteria	p__Bacteroidota	c__Bacteroidia	o__Bacteroidales	f__Bacteroidaceae	g__Bacteroides	s__Bacteroides unknown_1
MGYG-HGUT-00016:s__Anaerotignum sp001304995	0.557501878	0.163486103	0.968532073	d__Bacteria	p__Firmicutes_A	c__Clostridia	o__Lachnospirales	f__Anaerotignaceae	g__Anaerotignum	s__Anaerotignum sp001304995
MGYG-HGUT-00017:s__Collinsella unknown_1	0.267955564	0.307798866	0.920494192	d__Bacteria	p__Actinobacteriota	c__Coriobacteriia	o__Coriobacteriales	f__Coriobacteriaceae	g__Collinsella	s__Collinsella unknown_1
MGYG-HGUT-00018:s__Coprococcus eutactus	0.188256958	0.360449989	0.891825559	d__Bacteria	p__Firmicutes_A	c__Clostridia	o__Lachnospirales	f__Lachnospiraceae	g__Coprococcus	s__Coprococcus eutactus
MGYG-HGUT-00022:s__Faecalibacterium prausnitzii_C	0.49394657	0.188745691	0.968532073	d__Bacteria	p__Firmicutes_A	c__Clostridia	o__Oscillospirales	f__Ruminococcaceae	g__Faecalibacterium	s__Faecalibacterium prausnitzii_C

MGYG-HGUT-00023:s__Streptococcus parasanguinis	0.034939848	0.57792525	0.768225834	d__Bacteria	p__Firmicutes	c__Bacilli	o__Lactobacillales	f__Streptococcaceae	g__Streptococcus	s__Streptococcus parasanguinis
MGYG-HGUT-00026:s__Catenibacterium mitsuokai	0.856526523	0.050272328	0.992878088	d__Bacteria	p__Firmicutes	c__Bacilli	o__Erysipelotrichales	f__Erysipelatoclostridiaceae	g__Catenibacterium	s__Catenibacterium mitsuokai
MGYG-HGUT-00027:s__Mitsuokella jalaludinii	0.994919891	0.001789221	0.998803346	d__Bacteria	p__Firmicutes_C	c__Negativicutes	o__Selenomonadales	f__Selenomonadaceae	g__Mitsuokella	s__Mitsuokella jalaludinii
MGYG-HGUT-00028:s__Anaerostipes hadrus_A	0.418456396	-0.226332463	0.93970925	d__Bacteria	p__Firmicutes_A	c__Clostridia	o__Lachnospirales	f__Lachnospiraceae	g__Anaerostipes	s__Anaerostipes hadrus_A
MGYG-HGUT-00029:s__Bacteroides finegoldii	0.373225749	-0.249185749	0.93970925	d__Bacteria	p__Bacteroidota	c__Bacteroidia	o__Bacteroidales	f__Bacteroidaceae	g__Bacteroides	s__Bacteroides finegoldii
MGYG-HGUT-00030:s__CAG-603 sp900066105	0.015566442	0.636457489	0.768225834	d__Bacteria	p__Firmicutes_A	c__Clostridia	o__Lachnospirales	f__Lachnospiraceae	g__CAG-603	s__CAG-603 sp900066105
MGYG-HGUT-00031:s__Blautia_A sp900066205	0.192405752	0.361236	0.891825559	d__Bacteria	p__Firmicutes_A	c__Clostridia	o__Lachnospirales	f__Lachnospiraceae	g__Blautia_A	s__Blautia_A sp900066205
MGYG-HGUT-00032:s__Hungatella effluvii	0.09175932	-0.463856413	0.883307256	d__Bacteria	p__Firmicutes_A	c__Clostridia	o__Lachnospirales	f__Lachnospiraceae	g__Hungatella	s__Hungatella effluvii
MGYG-HGUT-00033:s__CAG-41 sp900066215	0.326519888	-0.27425	0.930624692	d__Bacteria	p__Firmicutes_A	c__Clostridia	o__Monoglobales	f__UBA1381	g__CAG-41	s__CAG-41 sp900066215

		2136								
MGYG-HGUT-00034:s__Massilimaliae timonensis	0.826521599	-0.059178842	0.991936936	d__Bacteria	p__Firmicutes_A	c__Clostridia	o__Oscillospirales	f__Ruminococcaceae	g__Massilimaliae	s__Massilimaliae timonensis
MGYG-HGUT-00035:s__Blautia_A hydrogenotrophica	0.850459609	0.053007375	0.992878088	d__Bacteria	p__Firmicutes_A	c__Clostridia	o__Lachnospirales	f__Lachnospiraceae	g__Blautia_A	s__Blautia_A hydrogenotrophica
MGYG-HGUT-00036:s__GCA-900066995 sp900291955	0.015527253	0.641317123	0.768225834	d__Bacteria	p__Firmicutes_A	c__Clostridia	o__Oscillospirales	f__Acutalibacteraceae	g__GCA-900066995	s__GCA-900066995 sp900291955
MGYG-HGUT-00037:s__Turicibacter sp001543345	0.80932571	0.066405396	0.987142302	d__Bacteria	p__Firmicutes	c__Bacilli	o__Haloplasmatales	f__Turicibacteraceae	g__Turicibacter	s__Turicibacter sp001543345
MGYG-HGUT-00038:s__Dorea longicatena	0.527255592	0.171469713	0.968532073	d__Bacteria	p__Firmicutes_A	c__Clostridia	o__Lachnospirales	f__Lachnospiraceae	g__Dorea	s__Dorea longicatena
MGYG-HGUT-00039:s__Faecalibacterium prausnitzii_H	0.053037262	0.537716758	0.85721132	d__Bacteria	p__Firmicutes_A	c__Clostridia	o__Oscillospirales	f__Ruminococcaceae	g__Faecalibacterium	s__Faecalibacterium prausnitzii_H
MGYG-HGUT-00040:s__UBA1191 sp900066305	0.841341057	-0.055607517	0.992878088	d__Bacteria	p__Firmicutes_A	c__Clostridia	o__Peptostreptococcales	f__Anaerovoracaceae	g__UBA1191	s__UBA1191 sp900066305
MGYG-HGUT-00041:s__Acetatifactor sp900066365	0.088419796	0.463476933	0.882716064	d__Bacteria	p__Firmicutes_A	c__Clostridia	o__Lachnospirales	f__Lachnospiraceae	g__Acetatifactor	s__Acetatifactor sp900066365
MGYG-HGUT-00042:s__Bacteroides_A sp900066445	0.22736	0.33603	0.90089	d__Bact	p__Bacteroidota	c__Bacteroidia	o__Bacteroidales	f__Bacteroidaceae	g__Bacteroides_A	s__Bacteroides_A sp900066445

	538	0699	1204	eria						
MGYG-HGUT-00043:s__Bacteroides_A sp900066455	0.366796923	0.249623861	0.939709925	d__Bacteria	p__Bacteroidota	c__Bacteroidia	o__Bacteroidales	f__Bacteroidaceae	g__Bacteroides_A	s__Bacteroides_A sp900066455
MGYG-HGUT-00044:s__Parabacteroides merdae	0.171115571	-0.381011765	0.891825559	d__Bacteria	p__Bacteroidota	c__Bacteroidia	o__Bacteroidales	f__Tannerellaceae	g__Parabacteroides	s__Parabacteroides merdae
MGYG-HGUT-00045:s__Erysipelatoclostridium sp000752095	0.444029365	0.213829023	0.960630116	d__Bacteria	p__Firmicutes	c__Bacilli	o__Erysipelotrichales	f__Erysipelatoclostridiaceae	g__Erysipelatoclostridium	s__Erysipelatoclostridium sp000752095
MGYG-HGUT-00050:s__Blautia_A sp900066505	0.986636478	0.004634953	0.998803346	d__Bacteria	p__Firmicutes_A	c__Clostridia	o__Lachnospirales	f__Lachnospiraceae	g__Blautia_A	s__Blautia_A sp900066505
MGYG-HGUT-00051:s__GCA-900066575 sp900066385	0.131332346	0.414867925	0.891825559	d__Bacteria	p__Firmicutes_A	c__Clostridia	o__Lachnospirales	f__Lachnospiraceae	g__GCA-900066575	s__GCA-900066575 sp900066385
MGYG-HGUT-00052:s__Anaerostipes sp000508985	0.401072307	-0.231195621	0.939709925	d__Bacteria	p__Firmicutes_A	c__Clostridia	o__Lachnospirales	f__Lachnospiraceae	g__Anaerostipes	s__Anaerostipes sp000508985
MGYG-HGUT-00054:s__Bacteroides acidifaciens	0.192894334	-0.358017349	0.891825559	d__Bacteria	p__Bacteroidota	c__Bacteroidia	o__Bacteroidales	f__Bacteroidaceae	g__Bacteroides	s__Bacteroides acidifaciens
MGYG-HGUT-00056:s__Muribaculum sp002358615	0.080891682	-0.482564632	0.877826247	d__Bacteria	p__Bacteroidota	c__Bacteroidia	o__Bacteroidales	f__Muribaculaceae	g__Muribaculum	s__Muribaculum sp002358615

MGYG-HGUT-00057:s__Bacteroides sp002491635	0.5870811 16	-0.152371 11	0.9756717 57	d__Bacteria	p__Bacteroidota	c__Bacteroidia	o__Bacteroidales	f__Bacteroidaceae	g__Bacteroides	s__Bacteroides sp002491635
MGYG-HGUT-00058:s__Eubacterium_I sp900066595	0.7864211 84	0.0758032 86	0.9820647 49	d__Bacteria	p__Firmicutes_A	c__Clostridia	o__Lachnospirales	f__Lachnospiraceae	g__Eubacterium_I	s__Eubacterium_I sp900066595
MGYG-HGUT-00059:s__Lawsonibacter sp900066645	0.3260692 42	0.2746805 24	0.9306246 92	d__Bacteria	p__Firmicutes_A	c__Clostridia	o__Oscillospirales	f__Oscillospiraceae	g__Lawsonibacter	s__Lawsonibacter sp900066645
MGYG-HGUT-00060:s__Lachnospira eligens_B	0.4058531 81	0.2295544 83	0.9397092 5	d__Bacteria	p__Firmicutes_A	c__Clostridia	o__Lachnospirales	f__Lachnospiraceae	g__Lachnospira	s__Lachnospira eligens_B
MGYG-HGUT-00061:s__Collinsella aerofaciens_E	0.1032476 26	0.4464328 59	0.8833072 56	d__Bacteria	p__Actinobacteriota	c__Coriobacteriia	o__Coriobacteriales	f__Coriobacteriaceae	g__Collinsella	s__Collinsella aerofaciens_E
MGYG-HGUT-00062:s__Intestinibacter bartlettii	0.5408789 6	-0.171768 797	0.9685320 73	d__Bacteria	p__Firmicutes_A	c__Clostridia	o__Peptostreptococcales	f__Peptostreptococcaceae	g__Intestinibacter	s__Intestinibacter bartlettii
MGYG-HGUT-00063:s__Emergencia sp900066695	0.0354420 39	0.5831418 63	0.7682258 34	d__Bacteria	p__Firmicutes_A	c__Clostridia	o__Peptostreptococcales	f__Anaerovoraceae	g__Emergencia	s__Emergencia sp900066695
MGYG-HGUT-00064:s__Clostridium baratii	0.0146050 95	-0.669295 483	0.7682258 34	d__Bacteria	p__Firmicutes_A	c__Clostridia	o__Clostridiales	f__Clostridiaceae	g__Clostridium	s__Clostridium baratii
MGYG-HGUT-00065:s__Anaerostipes sp900066705	0.7972836 61	-0.07149	0.9846205 27	d__Bacteria	p__Firmicutes_A	c__Clostridia	o__Lachnospirales	f__Lachnospiraceae	g__Anaerostipes	s__Anaerostipes sp900066705

		3946								
MGYG-HGUT-00066:s__GCA-900066755 sp900066755	0.788680034	0.075290956	0.982825119	d__Bacteria	p__Firmicutes_A	c__Clostridia	o__Lachnospirales	f__Lachnospiraceae	g__GCA-900066755	s__GCA-900066755 sp900066755
MGYG-HGUT-00067:s__Dorea sp900066765	0.051610074	0.531357679	0.85721132	d__Bacteria	p__Firmicutes_A	c__Clostridia	o__Lachnospirales	f__Lachnospiraceae	g__Dorea	s__Dorea sp900066765
MGYG-HGUT-00068:s__Agathobaculum sp900291975	0.074789449	0.479913478	0.877826247	d__Bacteria	p__Firmicutes_A	c__Clostridia	o__Oscillospirales	f__Butyricicoccaceae	g__Agathobaculum	s__Agathobaculum sp900291975
MGYG-HGUT-00069:s__Clostridium_A leptum	0.097114067	0.450541923	0.883307256	d__Bacteria	p__Firmicutes_A	c__Clostridia	o__Oscillospirales	f__Acutalibacteraceae	g__Clostridium_A	s__Clostridium_A leptum
MGYG-HGUT-00070:s__Lawsonibacter sp900066825	0.927595047	-0.023879095	0.994479474	d__Bacteria	p__Firmicutes_A	c__Clostridia	o__Oscillospirales	f__Oscillospiraceae	g__Lawsonibacter	s__Lawsonibacter sp900066825
MGYG-HGUT-00071:s__Pseudoflavonifractor capillosus	0.788412675	0.072090343	0.982825119	d__Bacteria	p__Firmicutes_A	c__Clostridia	o__Oscillospirales	f__Oscillospiraceae	g__Pseudoflavonifractor	s__Pseudoflavonifractor capillosus
MGYG-HGUT-00073:s__GCA-900066905 sp900066905	0.435957863	0.214414138	0.950806594	d__Bacteria	p__Firmicutes_A	c__Clostridia	o__Christensenellales	f__GCA-900066905	g__GCA-900066905	s__GCA-900066905 sp900066905
MGYG-HGUT-00074:s__Alistipes onderdonkii	0.877424154	-0.042085724	0.994479474	d__Bacteria	p__Bacteroidota	c__Bacteroidia	o__Bacteroidales	f__Rikenellaceae	g__Alistipes	s__Alistipes onderdonkii
MGYG-HGUT-00076:s__Roseburia intestinalis	0.34850	0.25724	0.93970	d__Bact	p__Firmicutes_A	c__Clostridia	o__Lachnospirales	f__Lachnospiraceae	g__Roseburia	s__Roseburia intestinalis

	7906	6585	925	eria						
MGYG-HGUT-00077:s__Eubacterium_E hallii_A	0.511891221	0.182646489	0.968532073	d__Bacteria	p__Firmicutes_A	c__Clostridia	o__Lachnospirales	f__Lachnospiraceae	g__Eubacterium_E	s__Eubacterium_E hallii_A
MGYG-HGUT-00078:s__Lachnospira rogosae	0.087701733	0.450941889	0.882716064	d__Bacteria	p__Firmicutes_A	c__Clostridia	o__Lachnospirales	f__Lachnospiraceae	g__Lachnospira	s__Lachnospira rogosae
MGYG-HGUT-00080:s__Anaerostipes caccae	0.241663119	-0.319856795	0.902181367	d__Bacteria	p__Firmicutes_A	c__Clostridia	o__Lachnospirales	f__Lachnospiraceae	g__Anaerostipes	s__Anaerostipes caccae
MGYG-HGUT-00084:s__Gemmiger formicilis	0.024617955	0.61452149	0.768225834	d__Bacteria	p__Firmicutes_A	c__Clostridia	o__Oscillospirales	f__Ruminococcaceae	g__Gemmiger	s__Gemmiger formicilis
MGYG-HGUT-00086:s__Lawsonibacter unknown_1	0.968911763	0.010914589	0.994479474	d__Bacteria	p__Firmicutes_A	c__Clostridia	o__Oscillospirales	f__Oscillospiraceae	g__Lawsonibacter	s__Lawsonibacter unknown_1
MGYG-HGUT-00087:s__CAG-81 sp000435795	0.205635088	0.351966462	0.891825559	d__Bacteria	p__Firmicutes_A	c__Clostridia	o__Lachnospirales	f__Lachnospiraceae	g__CAG-81	s__CAG-81 sp000435795
MGYG-HGUT-00088:s__Streptococcus lutetiensis	0.725167259	-0.092948475	0.979458475	d__Bacteria	p__Firmicutes	c__Bacilli	o__Lactobacillales	f__Streptococcaceae	g__Streptococcus	s__Streptococcus lutetiensis
MGYG-HGUT-00089:s__CAG-353 sp900066885	0.652270469	0.121814011	0.975671757	d__Bacteria	p__Firmicutes_A	c__Clostridia	o__Oscillospirales	f__Ruminococcaceae	g__CAG-353	s__CAG-353 sp900066885
MGYG-HGUT-00090:s__Lawsonibacter unknown_2	0.81225	0.06346	0.98714	d__Bact	p__Firmicutes_A	c__Clostridia	o__Oscillospirales	f__Oscillospiraceae	g__Lawsonibacter	s__Lawsonibacter unknown_2

	7084	6191	2302	eria						
MGYG-HGUT-00092:s__UBA7160 unknown_1	0.717549196	0.09931074	0.979458475	d__Bacteria	p__Firmicutes_A	c__Clostridia	o__Lachnospirales	f__Lachnospiraceae	g__UBA7160	s__UBA7160 unknown_1
MGYG-HGUT-00093:s__Klebsiella_A grimontii	0.853797452	-0.049889908	0.992878088	d__Bacteria	p__Proteobacteria	c__Gammaproteobacteria	o__Enterobacterales	f__Enterobacteriaceae	g__Klebsiella_A	s__Klebsiella_A grimontii
MGYG-HGUT-00094:s__Collinsella unknown_2	0.403475353	0.227745056	0.93970925	d__Bacteria	p__Actinobacteriota	c__Coriobacteriia	o__Coriobacteriales	f__Coriobacteriaceae	g__Collinsella	s__Collinsella unknown_2
MGYG-HGUT-00095:s__GCA-900066755 unknown	0.757131359	0.080612232	0.979458475	d__Bacteria	p__Firmicutes_A	c__Clostridia	o__Lachnospirales	f__Lachnospiraceae	g__GCA-900066755	s__GCA-900066755 unknown
MGYG-HGUT-00098:s__Bacteroides bouchesdurhonensis	0.87947238	0.042275306	0.994479474	d__Bacteria	p__Bacteroidota	c__Bacteroidia	o__Bacteroidales	f__Bacteroidaceae	g__Bacteroides	s__Bacteroides bouchesdurhonensis
MGYG-HGUT-00099:s__Flavonifractor plautii	0.906028119	-0.032818829	0.994479474	d__Bacteria	p__Firmicutes_A	c__Clostridia	o__Oscillospirales	f__Oscillospiraceae	g__Flavonifractor	s__Flavonifractor plautii
MGYG-HGUT-00102:s__Terrisporobacter unknown_1	0.359674336	-0.243803998	0.93970925	d__Bacteria	p__Firmicutes_A	c__Clostridia	o__Peptostreptococcales	f__Peptostreptococcaceae	g__Terrisporobacter	s__Terrisporobacter unknown_1
MGYG-HGUT-00104:s__Clostridium unknown_1	0.386343486	-0.242546685	0.93970925	d__Bacteria	p__Firmicutes_A	c__Clostridia	o__Clostridiales	f__Clostridiaceae	g__Clostridium	s__Clostridium unknown_1

MGYG-HGUT-00105:s__Bacteroides clarus	0.111354859	-0.440360997	0.883307256	d__Bacteria	p__Bacteroidota	c__Bacteroidia	o__Bacteroidales	f__Bacteroidaceae	g__Bacteroides	s__Bacteroides clarus
MGYG-HGUT-00106:s__Enterococcus_D gallinarum	0.147467298	-0.397817021	0.891825559	d__Bacteria	p__Firmicutes	c__Bacilli	o__Lactobacillales	f__Enterococcaceae	g__Enterococcus_D	s__Enterococcus_D gallinarum
MGYG-HGUT-00108:s__Citrobacter freundii	0.75344932	0.087874568	0.979458475	d__Bacteria	p__Proteobacteria	c__Gammaproteobacteria	o__Enterobacterales	f__Enterobacteriaceae	g__Citrobacter	s__Citrobacter freundii
MGYG-HGUT-00109:s__Streptococcus unknown_1	0.376922957	0.247816768	0.939709925	d__Bacteria	p__Firmicutes	c__Bacilli	o__Lactobacillales	f__Streptococcaceae	g__Streptococcus	s__Streptococcus unknown_1
MGYG-HGUT-00112:s__Actinomyces sp900323545	0.554017714	0.162312692	0.968532073	d__Bacteria	p__Actinobacteriota	c__Actinobacteria	o__Actinomycetales	f__Actinomycetaceae	g__Actinomyces	s__Actinomyces sp900323545
MGYG-HGUT-00113:s__Streptococcus salivarius	0.385195871	0.242918218	0.939709925	d__Bacteria	p__Firmicutes	c__Bacilli	o__Lactobacillales	f__Streptococcaceae	g__Streptococcus	s__Streptococcus salivarius
MGYG-HGUT-00114:s__Streptococcus unknown_2	0.488613314	-0.194045635	0.968532073	d__Bacteria	p__Firmicutes	c__Bacilli	o__Lactobacillales	f__Streptococcaceae	g__Streptococcus	s__Streptococcus unknown_2
MGYG-HGUT-00117:s__Barnesiella sp003150885	0.14317724	-0.406117261	0.891825559	d__Bacteria	p__Bacteroidota	c__Bacteroidia	o__Bacteroidales	f__Barnesiellaceae	g__Barnesiella	s__Barnesiella sp003150885
MGYG-HGUT-00118:s__Lachnospiraceae unknown unknown_1	0.96356	0.01274	0.99447	d__Bact	p__Firmicutes_A	c__Clostridia	o__Lachnospirales	f__Lachnospiraceae	g__Lachnospiraceae unknown	s__Lachnospiraceae unknown unknown_1

	7186	581	9474	eria						
MGYG-HGUT-00119:s__Blautia sp000432195	0.031843242	0.587530296	0.768225834	d__Bacteria	p__Firmicutes_A	c__Clostridia	o__Lachnospirales	f__Lachnospiraceae	g__Blautia	s__Blautia sp000432195
MGYG-HGUT-00121:s__Eubacterium callanderi	0.417088975	0.219715995	0.93970925	d__Bacteria	p__Firmicutes_A	c__Clostridia	o__Eubacteriales	f__Eubacteriaceae	g__Eubacterium	s__Eubacterium callanderi
MGYG-HGUT-00123:s__Blautia sp001304935	0.707563904	-0.103549338	0.979458475	d__Bacteria	p__Firmicutes_A	c__Clostridia	o__Lachnospirales	f__Lachnospiraceae	g__Blautia	s__Blautia sp001304935
MGYG-HGUT-00124:s__Citrobacter pasteurii	0.550829887	0.159592883	0.968532073	d__Bacteria	p__Proteobacteria	c__Gammaproteobacteria	o__Enterobacterales	f__Enterobacteriaceae	g__Citrobacter	s__Citrobacter pasteurii
MGYG-HGUT-00126:s__Anaerovoracaceae unknown unknown_2	0.099039458	0.453340084	0.883307256	d__Bacteria	p__Firmicutes_A	c__Clostridia	o__Peptostreptococcales	f__Anaerovoracaceae	g__Anaerovoracaceae unknown	s__Anaerovoracaceae unknown unknown_2
MGYG-HGUT-00127:s__UBA7096 unknown_1	0.285773721	-0.287802232	0.929025913	d__Bacteria	p__Firmicutes_A	c__Clostridia	o__Lachnospirales	f__Lachnospiraceae	g__UBA7096	s__UBA7096 unknown_1
MGYG-HGUT-00128:s__Alistipes unknown_1	0.981457769	0.006497398	0.997647914	d__Bacteria	p__Bacteroidota	c__Bacteroidia	o__Bacteroidales	f__Rikenellaceae	g__Alistipes	s__Alistipes unknown_1
MGYG-HGUT-00129:s__Marseille-P4683 sp900232885	0.379644486	0.244750125	0.93970925	d__Bacteria	p__Firmicutes_A	c__Clostridia	o__Oscillospirales	f__Acutalibacteraceae	g__Marseille-P4683	s__Marseille-P4683 sp900232885
MGYG-HGUT-00131:s__Anaerotruncus rubiinfantis	0.78517	-0.06797	0.98119	d__Bact	p__Firmicutes_A	c__Clostridia	o__Oscillospirales	f__Ruminococcaceae	g__Anaerotruncus	s__Anaerotruncus rubiinfantis

	4998	8153	4211	eria						
MGYG-HGUT-00133:s__Blautia_A sp000433815	0.949996294	-0.0017394093	0.994479474	d__Bacteria	p__Firmicutes_A	c__Clostridia	o__Lachnospirales	f__Lachnospiraceae	g__Blautia_A	s__Blautia_A sp000433815
MGYG-HGUT-00136:s__Agathobacter sp000434275	0.28678307	0.28842292	0.929025913	d__Bacteria	p__Firmicutes_A	c__Clostridia	o__Lachnospirales	f__Lachnospiraceae	g__Agathobacter	s__Agathobacter sp000434275
MGYG-HGUT-00137:s__UBA7160 unknown_2	0.577001336	0.152709487	0.975671757	d__Bacteria	p__Firmicutes_A	c__Clostridia	o__Lachnospirales	f__Lachnospiraceae	g__UBA7160	s__UBA7160 unknown_2
MGYG-HGUT-00138:s__Parabacteroides johnsonii	0.139268221	-0.412251676	0.891825559	d__Bacteria	p__Bacteroidota	c__Bacteroidia	o__Bacteroidales	f__Tannerellaceae	g__Parabacteroides	s__Parabacteroides johnsonii
MGYG-HGUT-00139:s__Eubacterium_G sp000434315	0.590593758	0.149085952	0.975671757	d__Bacteria	p__Firmicutes_A	c__Clostridia	o__Lachnospirales	f__Lachnospiraceae	g__Eubacterium_G	s__Eubacterium_G sp000434315
MGYG-HGUT-00140:s__CAG-56 sp900066615	0.169187864	0.37951308	0.891825559	d__Bacteria	p__Firmicutes_A	c__Clostridia	o__Lachnospirales	f__Lachnospiraceae	g__CAG-56	s__CAG-56 sp900066615
MGYG-HGUT-00141:s__Lachnospiraceae unknown unknown_2	0.179503966	0.360851441	0.891825559	d__Bacteria	p__Firmicutes_A	c__Clostridia	o__Lachnospirales	f__Lachnospiraceae	g__Lachnospiraceae unknown	s__Lachnospiraceae unknown unknown_2
MGYG-HGUT-00142:s__Blautia_A massiliensis	0.347209113	0.246860787	0.93970925	d__Bacteria	p__Firmicutes_A	c__Clostridia	o__Lachnospirales	f__Lachnospiraceae	g__Blautia_A	s__Blautia_A massiliensis
MGYG-HGUT-00144:s__Barnesiella intestinihominis	0.25695	-0.31719	0.90566	d__Bact	p__Bacteroidota	c__Bacteroidia	o__Bacteroidales	f__Barnesiellaceae	g__Barnesiella	s__Barnesiella intestinihominis

	186	1716	6614	eria						
MGYG-HGUT-00146:s__Dorea formicigenerans	0.567181192	0.154616417	0.971339903	d__Bacteria	p__Firmicutes_A	c__Clostridia	o__Lachnospirales	f__Lachnospiraceae	g__Dorea	s__Dorea formicigenerans
MGYG-HGUT-00148:s__Tyzzerella nexilis	0.417437632	-0.21788484	0.939709925	d__Bacteria	p__Firmicutes_A	c__Clostridia	o__Lachnospirales	f__Lachnospiraceae	g__Tyzzerella	s__Tyzzerella nexilis
MGYG-HGUT-00149:s__Clostridium saudiense	0.669987797	-0.119512283	0.975671757	d__Bacteria	p__Firmicutes_A	c__Clostridia	o__Clostridiales	f__Clostridiaceae	g__Clostridium	s__Clostridium saudiense
MGYG-HGUT-00150:s__Hungatella hathewayi	0.759511778	0.080552115	0.979458475	d__Bacteria	p__Firmicutes_A	c__Clostridia	o__Lachnospirales	f__Lachnospiraceae	g__Hungatella	s__Hungatella hathewayi
MGYG-HGUT-00151:s__Clostridium paraputrificum	0.636400153	0.131357825	0.975671757	d__Bacteria	p__Firmicutes_A	c__Clostridia	o__Clostridiales	f__Clostridiaceae	g__Clostridium	s__Clostridium paraputrificum
MGYG-HGUT-00153:s__CAG-45 sp000438375	0.247926993	0.318979386	0.902181367	d__Bacteria	p__Firmicutes_A	c__Clostridia	o__Lachnospirales	f__Lachnospiraceae	g__CAG-45	s__CAG-45 sp000438375
MGYG-HGUT-00154:s__CAG-45 sp900066395	0.639617371	0.130482486	0.975671757	d__Bacteria	p__Firmicutes_A	c__Clostridia	o__Lachnospirales	f__Lachnospiraceae	g__CAG-45	s__CAG-45 sp900066395
MGYG-HGUT-00155:s__Weissella confusa	0.280743884	-0.295594898	0.929025913	d__Bacteria	p__Firmicutes	c__Bacilli	o__Lactobacillales	f__Lactobacillaceae	g__Weissella	s__Weissella confusa
MGYG-HGUT-00156:s__Collinsella sp002232035_1	0.05724	0.52192	0.85721	d__Bact	p__Actino	c__Coriobacteriia	o__Coriobacteriales	f__Coriobacteriaceae	g__Collinsella	s__Collinsella sp002232035_1

	7103	5686	132	eria	bacteriota					
MGYG-HGUT-00158:s__Collinsella unknown_3	0.111789894	0.435605329	0.883307256	d__Bacteria	p__Actinobacteriota	c__Coriobacteriia	o__Coriobacteriales	f__Coriobacteriaceae	g__Collinsella	s__Collinsella unknown_3
MGYG-HGUT-00159:s__Sellimonas intestinalis	0.250602502	-0.318357233	0.902181367	d__Bacteria	p__Firmicutes_A	c__Clostridia	o__Lachnospirales	f__Lachnospiraceae	g__Sellimonas	s__Sellimonas intestinalis
MGYG-HGUT-00164:s__Lachnospiraceae unknown unknown_3	0.293021589	0.293700055	0.929025913	d__Bacteria	p__Firmicutes_A	c__Clostridia	o__Lachnospirales	f__Lachnospiraceae	g__Lachnospiraceae unknown	s__Lachnospiraceae unknown unknown_3
MGYG-HGUT-00166:s__Lactobacillus_H fermentum	0.353520979	0.249864656	0.93970925	d__Bacteria	p__Firmicutes	c__Bacilli	o__Lactobacillales	f__Lactobacillaceae	g__Lactobacillus_H	s__Lactobacillus_H fermentum
MGYG-HGUT-00167:s__Collinsella sp002232035_2	0.242413854	0.317891844	0.902181367	d__Bacteria	p__Actinobacteriota	c__Coriobacteriia	o__Coriobacteriales	f__Coriobacteriaceae	g__Collinsella	s__Collinsella sp002232035_2
MGYG-HGUT-00168:s__Butyricimonas unknown_1	0.749802817	0.087974305	0.979458475	d__Bacteria	p__Bacteroidota	c__Bacteroidia	o__Bacteroidales	f__Marinifilaceae	g__Butyricimonas	s__Butyricimonas unknown_1
MGYG-HGUT-00169:s__Eubacterium_G sp000432355	0.382562708	0.244261667	0.93970925	d__Bacteria	p__Firmicutes_A	c__Clostridia	o__Lachnospirales	f__Lachnospiraceae	g__Eubacterium_G	s__Eubacterium_G sp000432355
MGYG-HGUT-00170:s__Alistipes_A sp900240235	0.059268782	-0.52344364	0.857211132	d__Bacteria	p__Bacteroidota	c__Bacteroidia	o__Bacteroidales	f__Rikenellaceae	g__Alistipes_A	s__Alistipes_A sp900240235
MGYG-HGUT-00171:s__Blautia_A sp900120195	0.21694	0.33623	0.89182	d__Bact	p__Firmicutes_A	c__Clostridia	o__Lachnospirales	f__Lachnospiraceae	g__Blautia_A	s__Blautia_A sp900120195

	9478	2659	5559	eria						
MGYG-HGUT-00172:s__Clostridium_M lavalense	0.594969292	0.147272858	0.975671757	d__Bacteria	p__Firmicutes_A	c__Clostridia	o__Lachnospirales	f__Lachnospiraceae	g__Clostridium_M	s__Clostridium_M lavalense
MGYG-HGUT-00174:s__Parabacteroides unknown_1	0.680793602	-0.113293476	0.975671757	d__Bacteria	p__Bacteroidota	c__Bacteroidia	o__Bacteroidales	f__Tannerellaceae	g__Parabacteroides	s__Parabacteroides unknown_1
MGYG-HGUT-00175:s__Faecalicatena sp000509105	0.484254589	0.195333388	0.968532073	d__Bacteria	p__Firmicutes_A	c__Clostridia	o__Lachnospirales	f__Lachnospiraceae	g__Faecalicatena	s__Faecalicatena sp000509105
MGYG-HGUT-00176:s__Faecalicatena orotica	0.552648651	-0.166731556	0.968532073	d__Bacteria	p__Firmicutes_A	c__Clostridia	o__Lachnospirales	f__Lachnospiraceae	g__Faecalicatena	s__Faecalicatena orotica
MGYG-HGUT-00177:s__Anaerotignum sp000436415	0.894003667	-0.035039301	0.994479474	d__Bacteria	p__Firmicutes_A	c__Clostridia	o__Lachnospirales	f__Anaerotignaceae	g__Anaerotignum	s__Anaerotignum sp000436415
MGYG-HGUT-00178:s__Lawsonibacter unknown_3	0.946476099	0.018337018	0.994479474	d__Bacteria	p__Firmicutes_A	c__Clostridia	o__Oscillospirales	f__Oscillospiraceae	g__Lawsonibacter	s__Lawsonibacter unknown_3
MGYG-HGUT-00179:s__Clostridium_M unknown_1	0.462763642	-0.205393487	0.965574692	d__Bacteria	p__Firmicutes_A	c__Clostridia	o__Lachnospirales	f__Lachnospiraceae	g__Clostridium_M	s__Clostridium_M unknown_1
MGYG-HGUT-00180:s__Collinsella unknown_5	0.032125572	0.58970082	0.768225834	d__Bacteria	p__Actinobacteriota	c__Coriobacteriia	o__Coriobacteriales	f__Coriobacteriaceae	g__Collinsella	s__Collinsella unknown_5

MGYG-HGUT-00181:s__UBA9414 unknown	0.292378607	0.285881554	0.929025913	d__Bacteria	p__Firmicutes_A	c__Clostridia	o__Lachnospirales	f__Lachnospiraceae	g__UBA9414	s__UBA9414 unknown
MGYG-HGUT-00183:s__Butyricimonas sp002161485	0.762404286	-0.0846986 86	0.979458475	d__Bacteria	p__Bacteroidota	c__Bacteroidia	o__Bacteroidales	f__Marinifilaceae	g__Butyricimonas	s__Butyricimonas sp002161485
MGYG-HGUT-00184:s__Blautia_A unknown_1	0.305241808	0.278925769	0.929025913	d__Bacteria	p__Firmicutes_A	c__Clostridia	o__Lachnospirales	f__Lachnospiraceae	g__Blautia_A	s__Blautia_A unknown_1
MGYG-HGUT-00185:s__Agathobaculum butyriciproducens	0.898931636	-0.0356358 53	0.994479474	d__Bacteria	p__Firmicutes_A	c__Clostridia	o__Oscillospirales	f__Butyricicoccaceae	g__Agathobaculum	s__Agathobaculum butyriciproducens
MGYG-HGUT-00186:s__Holdemanella unknown_1	0.941870712	0.0202733	0.994479474	d__Bacteria	p__Firmicutes	c__Bacilli	o__Erysipelotrichales	f__Erysipelotrichaceae	g__Holdemanella	s__Holdemanella unknown_1
MGYG-HGUT-00187:s__Lachnospira sp003537285	0.418327521	0.223487682	0.93970925	d__Bacteria	p__Firmicutes_A	c__Clostridia	o__Lachnospirales	f__Lachnospiraceae	g__Lachnospira	s__Lachnospira sp003537285
MGYG-HGUT-00188:s__Collinsella unknown_6	0.028900826	0.593991852	0.768225834	d__Bacteria	p__Actinobacteriota	c__Coriobacteriia	o__Coriobacteriales	f__Coriobacteriaceae	g__Collinsella	s__Collinsella unknown_6
MGYG-HGUT-00189:s__Coprococcus sp900066115	0.663613732	-0.1219681 77	0.975671757	d__Bacteria	p__Firmicutes_A	c__Clostridia	o__Lachnospirales	f__Lachnospiraceae	g__Coprococcus	s__Coprococcus sp900066115
MGYG-HGUT-00190:s__CAG-127 sp900319515	0.666926084	-0.11844	0.975671757	d__Bacteria	p__Firmicutes_A	c__Clostridia	o__Lachnospirales	f__Lachnospiraceae	g__CAG-127	s__CAG-127 sp900319515

		3869								
MGYG-HGUT-00192:s__Holdemania filiformis	0.625319813	-0.135783001	0.975671757	d__Bacteria	p__Firmicutes	c__Bacilli	o__Erysipelotrichales	f__Erysipelotrichaceae	g__Holdemania	s__Holdemania filiformis
MGYG-HGUT-00193:s__KLE1615 sp900066985	0.471343952	0.196849661	0.968194079	d__Bacteria	p__Firmicutes_A	c__Clostridia	o__Lachnospirales	f__Lachnospiraceae	g__KLE1615	s__KLE1615 sp900066985
MGYG-HGUT-00194:s__UBA9502 unknown_1	0.625565734	0.133332969	0.975671757	d__Bacteria	p__Firmicutes_A	c__Clostridia	o__Lachnospirales	f__Lachnospiraceae	g__UBA9502	s__UBA9502 unknown_1
MGYG-HGUT-00195:s__Faecalibacterium prausnitzii_E	0.259466123	0.312355473	0.910935093	d__Bacteria	p__Firmicutes_A	c__Clostridia	o__Oscillospirales	f__Ruminococcaceae	g__Faecalibacterium	s__Faecalibacterium prausnitzii_E
MGYG-HGUT-00196:s__Bacteroides thetaiotaomicron	0.567475068	-0.158002909	0.97133903	d__Bacteria	p__Bacteroidota	c__Bacteroidia	o__Bacteroidales	f__Bacteroidaceae	g__Bacteroides	s__Bacteroides thetaiotaomicron
MGYG-HGUT-00197:s__Collinsella unknown_7	0.652687976	0.12124068	0.975671757	d__Bacteria	p__Actinobacteriota	c__Coriobacteriia	o__Coriobacteriales	f__Coriobacteriaceae	g__Collinsella	s__Collinsella unknown_7
MGYG-HGUT-00198:s__Clostridium_M citroniae	0.556077573	0.163471844	0.968532073	d__Bacteria	p__Firmicutes_A	c__Clostridia	o__Lachnospirales	f__Lachnospiraceae	g__Clostridium_M	s__Clostridium_M citroniae
MGYG-HGUT-00199:s__Streptococcus parasanguinis_B	0.779075301	0.078535726	0.979520484	d__Bacteria	p__Firmicutes	c__Bacilli	o__Lactobacillales	f__Streptococcaceae	g__Streptococcus	s__Streptococcus parasanguinis_B
MGYG-HGUT-00200:s__Blautia_A unknown_2	0.54677	0.16439	0.96853	d__Bact	p__Firmicutes_A	c__Clostridia	o__Lachnospirales	f__Lachnospiraceae	g__Blautia_A	s__Blautia_A unknown_2

	0507	0722	2073	eria						
MGYG-HGUT-00201:s__Blautia_A sp900066145	0.939059828	0.021218922	0.994479474	d__Bacteria	p__Firmicutes_A	c__Clostridia	o__Lachnospirales	f__Lachnospiraceae	g__Blautia_A	s__Blautia_A sp900066145
MGYG-HGUT-00202:s__Lachnospiraceae unknown unknown_4	0.276311109	0.300972277	0.929025913	d__Bacteria	p__Firmicutes_A	c__Clostridia	o__Lachnospirales	f__Lachnospiraceae	g__Lachnospiraceae unknown	s__Lachnospiraceae unknown unknown_4
MGYG-HGUT-00203:s__Butyricimonas virosa	0.549064608	0.164343129	0.968532073	d__Bacteria	p__Bacteroidota	c__Bacteroidia	o__Bacteroidales	f__Marinifilaceae	g__Butyricimonas	s__Butyricimonas virosa
MGYG-HGUT-00204:s__Eubacterium_G sp000435815	0.991769885	-0.002875957	0.998803346	d__Bacteria	p__Firmicutes_A	c__Clostridia	o__Lachnospirales	f__Lachnospiraceae	g__Eubacterium_G	s__Eubacterium_G sp000435815
MGYG-HGUT-00205:s__CAG-81 sp900066535	0.427598436	0.219940786	0.950396026	d__Bacteria	p__Firmicutes_A	c__Clostridia	o__Lachnospirales	f__Lachnospiraceae	g__CAG-81	s__CAG-81 sp900066535
MGYG-HGUT-00206:s__Dorea sp900066555	0.792572336	-0.072843001	0.9829663	d__Bacteria	p__Firmicutes_A	c__Clostridia	o__Lachnospirales	f__Lachnospiraceae	g__Dorea	s__Dorea sp900066555
MGYG-HGUT-00207:s__Collinsella sp002232035_3	0.205838262	0.353850208	0.891825559	d__Bacteria	p__Actinobacteriota	c__Coriobacteriia	o__Coriobacteriales	f__Coriobacteriaceae	g__Collinsella	s__Collinsella sp002232035_3
MGYG-HGUT-00208:s__Eubacterium_G ventriosum	0.83297019	-0.058595827	0.99222019	d__Bacteria	p__Firmicutes_A	c__Clostridia	o__Lachnospirales	f__Lachnospiraceae	g__Eubacterium_G	s__Eubacterium_G ventriosum
MGYG-HGUT-00209:s__Eubacter	0.92401	-0.026	0.99447	d__Bact	p__Firmicutes_A	c__Clostridia	o__Lachnospirales	f__Lachnospiraceae	g__Eubacterium_F	s__Eubacterium_F sp003491505

ium_F sp003491505	1309	76248	9474	eria						
MGYG-HGUT-00211:s__Bacteroides intestinalis_A	0.197444008	-0.357649182	0.891825559	d__Bacteria	p__Bacteroidota	c__Bacteroidia	o__Bacteroidales	f__Bacteroidaceae	g__Bacteroides	s__Bacteroides intestinalis_A
MGYG-HGUT-00212:s__Blautia_A obeum	0.741832857	0.091659958	0.979458475	d__Bacteria	p__Firmicutes_A	c__Clostridia	o__Lachnospirales	f__Lachnospiraceae	g__Blautia_A	s__Blautia_A obeum
MGYG-HGUT-00213:s__Blautia_A unknown_3	0.261289022	0.301905599	0.913131421	d__Bacteria	p__Firmicutes_A	c__Clostridia	o__Lachnospirales	f__Lachnospiraceae	g__Blautia_A	s__Blautia_A unknown_3
MGYG-HGUT-00215:s__Prevotella stercorea	0.621293066	-0.138482108	0.975671757	d__Bacteria	p__Bacteroidota	c__Bacteroidia	o__Bacteroidales	f__Bacteroidaceae	g__Prevotella	s__Prevotella stercorea
MGYG-HGUT-00216:s__Blautia_A unknown_4	0.215388934	0.341319297	0.891825559	d__Bacteria	p__Firmicutes_A	c__Clostridia	o__Lachnospirales	f__Lachnospiraceae	g__Blautia_A	s__Blautia_A unknown_4
MGYG-HGUT-00217:s__Acetatifactor sp900066565	0.114483116	0.418463661	0.883307256	d__Bacteria	p__Firmicutes_A	c__Clostridia	o__Lachnospirales	f__Lachnospiraceae	g__Acetatifactor	s__Acetatifactor sp900066565
MGYG-HGUT-00219:s__Eubacterium limosum_A	0.006715567	0.693177133	0.750056424	d__Bacteria	p__Firmicutes_A	c__Clostridia	o__Eubacteriales	f__Eubacteriaceae	g__Eubacterium	s__Eubacterium limosum_A
MGYG-HGUT-00220:s__Butyricicoccaceae unknown unknown_1	0.58135784	-0.141336289	0.975671757	d__Bacteria	p__Firmicutes_A	c__Clostridia	o__Oscillospirales	f__Butyricicoccaceae	g__Butyricicoccaceae unknown	s__Butyricicoccaceae unknown unknown_1

MGYG-HGUT-00222:s__Parabacteroides unknown_2	0.910506615	0.031256869	0.994479474	d__Bacteria	p__Bacteroidota	c__Bacteroidia	o__Bacteroidales	f__Tannerellaceae	g__Parabacteroides	s__Parabacteroides unknown_2
MGYG-HGUT-00223:s__Absiella unknown_1	0.247768959	0.318004186	0.902181367	d__Bacteria	p__Firmicutes	c__Bacilli	o__Erysipelotrichales	f__Erysipelotrichaceae	g__Absiella	s__Absiella unknown_1
MGYG-HGUT-00224:s__Bacteroides sp003545565	0.841583598	0.056169799	0.992878088	d__Bacteria	p__Bacteroidota	c__Bacteroidia	o__Bacteroidales	f__Bacteroidaceae	g__Bacteroides	s__Bacteroides sp003545565
MGYG-HGUT-00226:s__Lactococcus lactis	0.755288157	0.086397375	0.979458475	d__Bacteria	p__Firmicutes	c__Bacilli	o__Lactobacillales	f__Streptococcaceae	g__Lactococcus	s__Lactococcus lactis
MGYG-HGUT-00228:s__Collinsella sp002232035_4	0.534138678	0.173868561	0.968532073	d__Bacteria	p__Actinobacteriota	c__Coriobacteriia	o__Coriobacteriales	f__Coriobacteriaceae	g__Collinsella	s__Collinsella sp002232035_4
MGYG-HGUT-00229:s__Dorea sp000433215	0.177770105	0.37181761	0.891825559	d__Bacteria	p__Firmicutes_A	c__Clostridia	o__Lachnospirales	f__Lachnospiraceae	g__Dorea	s__Dorea sp000433215
MGYG-HGUT-00231:s__Coprococcus eutactus_A	0.967454799	-0.011064023	0.994479474	d__Bacteria	p__Firmicutes_A	c__Clostridia	o__Lachnospirales	f__Lachnospiraceae	g__Coprococcus	s__Coprococcus eutactus_A
MGYG-HGUT-00232:s__Collinsella unknown_9	0.928563339	-0.024770339	0.994479474	d__Bacteria	p__Actinobacteriota	c__Coriobacteriia	o__Coriobacteriales	f__Coriobacteriaceae	g__Collinsella	s__Collinsella unknown_9
MGYG-HGUT-00233:s__CAG-81 sp900066785	0.281644304	0.301305387	0.929025913	d__Bacteria	p__Firmicutes_A	c__Clostridia	o__Lachnospirales	f__Lachnospiraceae	g__CAG-81	s__CAG-81 sp900066785

MGYG-HGUT-00235:s__Enterobacter kobei	0.491349692	0.182962496	0.968532073	d__Bacteria	p__Proteobacteria	c__Gammaproteobacteria	o__Enterobacterales	f__Enterobacteriaceae	g__Enterobacter	s__Enterobacter kobei
MGYG-HGUT-00236:s__Bacteroides fragilis_A	0.099357726	-0.455263831	0.883307256	d__Bacteria	p__Bacteroidota	c__Bacteroidia	o__Bacteroidales	f__Bacteroidaceae	g__Bacteroides	s__Bacteroides fragilis_A
MGYG-HGUT-00237:s__Collinsella unknown_10	0.915737247	-0.028288439	0.994479474	d__Bacteria	p__Actinobacteriota	c__Coriobacteriia	o__Coriobacteriales	f__Coriobacteriaceae	g__Collinsella	s__Collinsella unknown_10
MGYG-HGUT-00238:s__Enterococcus_D casseliflavus	0.496701926	-0.18747482	0.968532073	d__Bacteria	p__Firmicutes	c__Bacilli	o__Lactobacillales	f__Enterococcaceae	g__Enterococcus_D	s__Enterococcus_D casseliflavus
MGYG-HGUT-00239:s__Collinsella unknown_11	0.103216862	0.440058363	0.883307256	d__Bacteria	p__Actinobacteriota	c__Coriobacteriia	o__Coriobacteriales	f__Coriobacteriaceae	g__Collinsella	s__Collinsella unknown_11
MGYG-HGUT-00240:s__Absiella unknown_2	0.577882945	-0.153716318	0.975671757	d__Bacteria	p__Firmicutes	c__Bacilli	o__Erysipelotrichales	f__Erysipelotrichaceae	g__Absiella	s__Absiella unknown_2
MGYG-HGUT-00242:s__Clostridium_M sp000431375	0.905055757	0.033109856	0.994479474	d__Bacteria	p__Firmicutes_A	c__Clostridia	o__Lachnospirales	f__Lachnospiraceae	g__Clostridium_M	s__Clostridium_M sp000431375
MGYG-HGUT-00243:s__Bacteroides_B massiliensis	0.902958208	0.033999918	0.994479474	d__Bacteria	p__Bacteroidota	c__Bacteroidia	o__Bacteroidales	f__Bacteroidaceae	g__Bacteroides_B	s__Bacteroides_B massiliensis
MGYG-HGUT-00244:s__Catenibacterium sp000437715	0.77479	0.07830	0.97952	d__Bact	p__Firmicutes	c__Bacilli	o__Erysipelotrichales	f__Erysipelatoclostridiaceae	g__Catenibacterium	s__Catenibacterium sp000437715

	9329	3699	0484	eria						
MGYG-HGUT-00245:s__Roseburia unknown_1	0.041579606	0.554555746	0.779321052	d__Bacteria	p__Firmicutes_A	c__Clostridia	o__Lachnospirales	f__Lachnospiraceae	g__Roseburia	s__Roseburia unknown_1
MGYG-HGUT-00246:s__Streptococcus pasteurianus	0.39546026	-0.235251576	0.93970925	d__Bacteria	p__Firmicutes	c__Bacilli	o__Lactobacillales	f__Streptococcaceae	g__Streptococcus	s__Streptococcus pasteurianus
MGYG-HGUT-00248:s__Weissella cibaria	0.364823366	-0.244928912	0.93970925	d__Bacteria	p__Firmicutes	c__Bacilli	o__Lactobacillales	f__Lactobacillaceae	g__Weissella	s__Weissella cibaria
MGYG-HGUT-00249:s__Faecalicatena faecis	0.034470905	0.573262506	0.768225834	d__Bacteria	p__Firmicutes_A	c__Clostridia	o__Lachnospirales	f__Lachnospiraceae	g__Faecalicatena	s__Faecalicatena faecis
MGYG-HGUT-00250:s__TF01-11 sp001414325	0.527419593	-0.175211933	0.968532073	d__Bacteria	p__Firmicutes_A	c__Clostridia	o__Lachnospirales	f__Lachnospiraceae	g__TF01-11	s__TF01-11 sp001414325
MGYG-HGUT-00251:s__Fusicatenibacter saccharivorans	0.046123509	0.508160311	0.799667098	d__Bacteria	p__Firmicutes_A	c__Clostridia	o__Lachnospirales	f__Lachnospiraceae	g__Fusicatenibacter	s__Fusicatenibacter saccharivorans
MGYG-HGUT-00252:s__Blautia_A obeum_B	0.626102662	0.131567995	0.975671757	d__Bacteria	p__Firmicutes_A	c__Clostridia	o__Lachnospirales	f__Lachnospiraceae	g__Blautia_A	s__Blautia_A obeum_B
MGYG-HGUT-00253:s__Holdemanella sp002299315	0.60110753	0.142986464	0.975671757	d__Bacteria	p__Firmicutes	c__Bacilli	o__Erysipelotrichales	f__Erysipelotrichaceae	g__Holdemanella	s__Holdemanella sp002299315
MGYG-HGUT-00254:s__Odoribacter splanchnicus	0.23924	0.32430	0.90218	d__Bact	p__Bacter	c__Bacteroidia	o__Bacteroidales	f__Marinifilaceae	g__Odoribacter	s__Odoribacter splanchnicus

	8638	8748	1367	eria	oidota					
MGYG-HGUT-00255:s__CAG-81 sp900066055	0.359765018	0.251786256	0.9397092 5	d__Bacteria	p__Firmicutes_A	c__Clostridia	o__Lachnospirales	f__Lachnospiraceae	g__CAG-81	s__CAG-81 sp900066055
MGYG-HGUT-00256:s__Olsenella_B sp000752675	0.772442432	0.080845933	0.979520484	d__Bacteria	p__Actinobacteriota	c__Coriobacteriia	o__Coriobacteriales	f__Atopobiaceae	g__Olsenella_B	s__Olsenella_B sp000752675
MGYG-HGUT-00257:s__Collinsella sp002232035_5	0.156683333	0.389218255	0.891825559	d__Bacteria	p__Actinobacteriota	c__Coriobacteriia	o__Coriobacteriales	f__Coriobacteriaceae	g__Collinsella	s__Collinsella sp002232035_5
MGYG-HGUT-00258:s__Ruminococcus_E bromii_B	0.47417626	0.196769689	0.968532073	d__Bacteria	p__Firmicutes_A	c__Clostridia	o__Oscillospirales	f__Acutalibacteraceae	g__Ruminococcus_E	s__Ruminococcus_E bromii_B
MGYG-HGUT-00259:s__UBA9502 unknown_2	0.0915435	0.458466191	0.883307256	d__Bacteria	p__Firmicutes_A	c__Clostridia	o__Lachnospirales	f__Lachnospiraceae	g__UBA9502	s__UBA9502 unknown_2
MGYG-HGUT-00261:s__Collinsella unknown_13	0.019859272	0.633732278	0.768225834	d__Bacteria	p__Actinobacteriota	c__Coriobacteriia	o__Coriobacteriales	f__Coriobacteriaceae	g__Collinsella	s__Collinsella unknown_13
MGYG-HGUT-00262:s__Eubacterium_E hallii	0.340301927	0.266145527	0.934131403	d__Bacteria	p__Firmicutes_A	c__Clostridia	o__Lachnospirales	f__Lachnospiraceae	g__Eubacterium_E	s__Eubacterium_E hallii
MGYG-HGUT-00263:s__Blautia_A sp900066335	0.358244694	0.256565634	0.9397092 5	d__Bacteria	p__Firmicutes_A	c__Clostridia	o__Lachnospirales	f__Lachnospiraceae	g__Blautia_A	s__Blautia_A sp900066335
MGYG-HGUT-00265:s__Bacteroides nordii	0.302050714	-0.287946003	0.929025913	d__Bacteria	p__Bacteroidota	c__Bacteroidia	o__Bacteroidales	f__Bacteroidaceae	g__Bacteroides	s__Bacteroides nordii

MGYG-HGUT-00266:s__Collinsella sp002232035_6	0.0342949	0.582784922	0.768225834	d__Bacteria	p__Actinobacteriota	c__Coriobacteriia	o__Coriobacteriales	f__Coriobacteriaceae	g__Collinsella	s__Collinsella sp002232035_6
MGYG-HGUT-00267:s__CAG-536 sp000434355	0.443902995	-0.212325622	0.960630116	d__Bacteria	p__Firmicutes	c__Bacilli	o__Erysipelotrichales	f__Erysipelotrichaceae	g__CAG-536	s__CAG-536 sp000434355
MGYG-HGUT-00268:s__Faecalicatena sp900066545	0.143932178	0.400547418	0.891825559	d__Bacteria	p__Firmicutes_A	c__Clostridia	o__Lachnospirales	f__Lachnospiraceae	g__Faecalicatena	s__Faecalicatena sp900066545
MGYG-HGUT-00269:s__Erysipelatoclostridium unknown_1	0.316899093	0.270976721	0.929025913	d__Bacteria	p__Firmicutes	c__Bacilli	o__Erysipelotrichales	f__Erysipelatoclostridiaceae	g__Erysipelatoclostridium	s__Erysipelatoclostridium unknown_1
MGYG-HGUT-00270:s__Collinsella unknown_14	0.241111472	0.324736097	0.902181367	d__Bacteria	p__Actinobacteriota	c__Coriobacteriia	o__Coriobacteriales	f__Coriobacteriaceae	g__Collinsella	s__Collinsella unknown_14
MGYG-HGUT-00271:s__Roseburia inulinivorans	0.282259122	0.280034517	0.929025913	d__Bacteria	p__Firmicutes_A	c__Clostridia	o__Lachnospirales	f__Lachnospiraceae	g__Roseburia	s__Roseburia inulinivorans
MGYG-HGUT-00272:s__Prevotella sp000434515	0.597142849	0.140809541	0.975671757	d__Bacteria	p__Bacteroidota	c__Bacteroidia	o__Bacteroidales	f__Bacteroidaceae	g__Prevotella	s__Prevotella sp000434515
MGYG-HGUT-00273:s__Bacteroides_A coprophilus	0.499565193	-0.18955726	0.968532073	d__Bacteria	p__Bacteroidota	c__Bacteroidia	o__Bacteroidales	f__Bacteroidaceae	g__Bacteroides_A	s__Bacteroides_A coprophilus
MGYG-HGUT-00274:s__UBA9502 sp003506385	0.071484992	0.499646644	0.877826247	d__Bacteria	p__Firmicutes_A	c__Clostridia	o__Lachnospirales	f__Lachnospiraceae	g__UBA9502	s__UBA9502 sp003506385

MGYG-HGUT-00275:s__Collinsella sp002232035_7	0.146360576	0.399047346	0.891825559	d__Bacteria	p__Actinobacteriota	c__Coriobacteriia	o__Coriobacteriales	f__Coriobacteriaceae	g__Collinsella	s__Collinsella sp002232035_7
MGYG-HGUT-00276:s__Caecibacter unknown_1	0.651291214	0.127013553	0.975671757	d__Bacteria	p__Firmicutes_C	c__Negativicutes	o__Veillonellales	f__Megasphaeraceae	g__Caecibacter	s__Caecibacter unknown_1
MGYG-HGUT-00277:s__Streptococcus mutans	0.324027314	0.276518332	0.930624692	d__Bacteria	p__Firmicutes	c__Bacilli	o__Lactobacillales	f__Streptococcaceae	g__Streptococcus	s__Streptococcus mutans
MGYG-HGUT-00278:s__Hungatella_A unknown_1	0.406601605	0.231890189	0.93970925	d__Bacteria	p__Firmicutes_A	c__Clostridia	o__Lachnospirales	f__Lachnospiraceae	g__Hungatella_A	s__Hungatella_A unknown_1
MGYG-HGUT-00280:s__Lachnospiraceae unknown unknown_5	0.849064066	-0.050128471	0.992878088	d__Bacteria	p__Firmicutes_A	c__Clostridia	o__Lachnospirales	f__Lachnospiraceae	g__Lachnospiraceae unknown	s__Lachnospiraceae unknown unknown_5
MGYG-HGUT-00282:s__TF01-11 sp003524945	0.115822449	0.438264431	0.883307256	d__Bacteria	p__Firmicutes_A	c__Clostridia	o__Lachnospirales	f__Lachnospiraceae	g__TF01-11	s__TF01-11 sp003524945
MGYG-HGUT-00285:s__UBA7185 unknown_1	0.960817851	0.013652694	0.994479474	d__Bacteria	p__Firmicutes_B	c__Peptococcia	o__Peptococcales	f__Peptococcaceae	g__UBA7185	s__UBA7185 unknown_1
MGYG-HGUT-00287:s__Robinsoniella unknown_1	0.164631299	-0.380871596	0.891825559	d__Bacteria	p__Firmicutes_A	c__Clostridia	o__Lachnospirales	f__Lachnospiraceae	g__Robinsoniella	s__Robinsoniella unknown_1
MGYG-HGUT-00290:s__Collinsella unknown_17	0.451026648	0.201931628	0.961292208	d__Bacteria	p__Actinobacteriota	c__Coriobacteriia	o__Coriobacteriales	f__Coriobacteriaceae	g__Collinsella	s__Collinsella unknown_17

MGYG-HGUT-00291:s__CAG-1427 unknown_1	0.397263627	0.235708225	0.93970925	d__Bacteria	p__Actinobacteriota	c__Coriobacteriia	o__Coriobacteriales	f__Eggerthellaceae	g__CAG-1427	s__CAG-1427 unknown_1
MGYG-HGUT-00292:s__Collinsella unknown_18	0.328742809	0.272693128	0.930624692	d__Bacteria	p__Actinobacteriota	c__Coriobacteriia	o__Coriobacteriales	f__Coriobacteriaceae	g__Collinsella	s__Collinsella unknown_18
MGYG-HGUT-00298:s__Streptococcus unknown_3	0.605443628	0.144684858	0.975671757	d__Bacteria	p__Firmicutes	c__Bacilli	o__Lactobacillales	f__Streptococcaceae	g__Streptococcus	s__Streptococcus unknown_3
MGYG-HGUT-00301:s__Blautia_A unknown_5	0.848756884	-0.053158176	0.992878088	d__Bacteria	p__Firmicutes_A	c__Clostridia	o__Lachnospirales	f__Lachnospiraceae	g__Blautia_A	s__Blautia_A unknown_5
MGYG-HGUT-00306:s__Collinsella sp003487125	0.284177708	0.290028675	0.929025913	d__Bacteria	p__Actinobacteriota	c__Coriobacteriia	o__Coriobacteriales	f__Coriobacteriaceae	g__Collinsella	s__Collinsella sp003487125
MGYG-HGUT-00308:s__Pauljensenia keddieii	0.224381124	0.328744231	0.891825559	d__Bacteria	p__Actinobacteriota	c__Actinobacteria	o__Actinomycetales	f__Actinomycetaceae	g__Pauljensenia	s__Pauljensenia keddieii
MGYG-HGUT-00312:s__Eisenbergiella unknown_1	0.604618061	-0.145496043	0.975671757	d__Bacteria	p__Firmicutes_A	c__Clostridia	o__Lachnospirales	f__Lachnospiraceae	g__Eisenbergiella	s__Eisenbergiella unknown_1
MGYG-HGUT-00314:s__Collinsella unknown_20	0.468127286	-0.198958545	0.968194079	d__Bacteria	p__Actinobacteriota	c__Coriobacteriia	o__Coriobacteriales	f__Coriobacteriaceae	g__Collinsella	s__Collinsella unknown_20
MGYG-HGUT-00316:s__UBA945 unknown	0.546581699	0.167929677	0.968532073	d__Bacteria	p__Firmicutes_A	c__Clostridia	o__Oscillospirales	f__Acutalibacteraceae	g__UBA945	s__UBA945 unknown

MGYG-HGUT-00318:s__Streptococcus infantis_I	0.1628089 84	0.3887940 33	0.8918255 59	d__Bacteria	p__Firmicutes	c__Bacilli	o__Lactobacillales	f__Streptococcaceae	g__Streptococcus	s__Streptococcus infantis_I
MGYG-HGUT-00320:s__Collinsella unknown_21	0.2552478 52	0.3079946 4	0.9050156 96	d__Bacteria	p__Actinobacteriota	c__Coriobacteriia	o__Coriobacteriales	f__Coriobacteriaceae	g__Collinsella	s__Collinsella unknown_21
MGYG-HGUT-00321:s__Collinsella unknown_22	0.5370141 95	0.1717424 61	0.9685320 73	d__Bacteria	p__Actinobacteriota	c__Coriobacteriia	o__Coriobacteriales	f__Coriobacteriaceae	g__Collinsella	s__Collinsella unknown_22
MGYG-HGUT-00324:s__Collinsella unknown_23	0.0044885 27	0.7703095 21	0.6684164 33	d__Bacteria	p__Actinobacteriota	c__Coriobacteriia	o__Coriobacteriales	f__Coriobacteriaceae	g__Collinsella	s__Collinsella unknown_23
MGYG-HGUT-00325:s__CAG-95 unknown_1	0.9415199 06	0.0198436 78	0.9944794 74	d__Bacteria	p__Firmicutes_A	c__Clostridia	o__Lachnospirales	f__Lachnospiraceae	g__CAG-95	s__CAG-95 unknown_1
MGYG-HGUT-00328:s__CAG-272 unknown	0.9656733 33	-0.0116227 43	0.9944794 74	d__Bacteria	p__Firmicutes_A	c__Clostridia	o__Oscillospirales	f__CAG-272	g__CAG-272	s__CAG-272 unknown
MGYG-HGUT-00340:s__Clostridium_E unknown	0.3392619 46	0.2649897 37	0.9327093 82	d__Bacteria	p__Firmicutes_A	c__Clostridia	o__Oscillospirales	f__Acutalibacteraceae	g__Clostridium_E	s__Clostridium_E unknown
MGYG-HGUT-00343:s__Odoribacter unknown_2	0.7039279 59	-0.1043365 07	0.9794584 75	d__Bacteria	p__Bacteroidota	c__Bacteroidia	o__Bacteroidales	f__Marinifilaceae	g__Odoribacter	s__Odoribacter unknown_2
MGYG-HGUT-00346:s__Lachnospiraceae unknown unknown_6	0.6080641 28	-0.1433625 09	0.9756717 57	d__Bacteria	p__Firmicutes_A	c__Clostridia	o__Lachnospirales	f__Lachnospiraceae	g__Lachnospiraceae unknown	s__Lachnospiraceae unknown unknown_6

MGYG-HGUT-00355:s__Parabacteroides unknown_4	0.649434522	-0.127571764	0.975671757	d__Bacteria	p__Bacteroidota	c__Bacteroidia	o__Bacteroidales	f__Tannerellaceae	g__Parabacteroides	s__Parabacteroides unknown_4
MGYG-HGUT-00356:s__Lachnobacterium unknown	0.121929678	0.427240589	0.885742175	d__Bacteria	p__Firmicutes_A	c__Clostridia	o__Lachnospirales	f__Lachnospiraceae	g__Lachnobacterium	s__Lachnobacterium unknown
MGYG-HGUT-00359:s__UBA7182 unknown_1	0.043610579	0.550001608	0.779321052	d__Bacteria	p__Firmicutes_A	c__Clostridia	o__Lachnospirales	f__Lachnospiraceae	g__UBA7182	s__UBA7182 unknown_1
MGYG-HGUT-00361:s__SZUA-378 unknown_1	0.293214521	0.284206813	0.929025913	d__Bacteria	p__Firmicutes_A	c__Clostridia	o__Lachnospirales	f__Lachnospiraceae	g__SZUA-378	s__SZUA-378 unknown_1
MGYG-HGUT-00364:s__Coprococcus unknown_1	0.262764208	0.304358031	0.913131421	d__Bacteria	p__Firmicutes_A	c__Clostridia	o__Lachnospirales	f__Lachnospiraceae	g__Coprococcus	s__Coprococcus unknown_1
MGYG-HGUT-00369:s__CAG-170 sp003516765	0.460673178	0.201180509	0.965574692	d__Bacteria	p__Firmicutes_A	c__Clostridia	o__Oscillospirales	f__Oscillospiraceae	g__CAG-170	s__CAG-170 sp003516765
MGYG-HGUT-00370:s__CAG-170 unknown_1	0.927224184	-0.024600857	0.994479474	d__Bacteria	p__Firmicutes_A	c__Clostridia	o__Oscillospirales	f__Oscillospiraceae	g__CAG-170	s__CAG-170 unknown_1
MGYG-HGUT-00373:s__UBA1255 unknown unknown_1	0.645029333	-0.128886484	0.975671757	d__Bacteria	p__Firmicutes_A	c__Clostridia	o__UBA1212	f__UBA1255	g__UBA1255 unknown	s__UBA1255 unknown unknown_1
MGYG-HGUT-00378:s__Lachnospiraceae unknown unknown_7	0.940513709	0.020940642	0.994479474	d__Bacteria	p__Firmicutes_A	c__Clostridia	o__Lachnospirales	f__Lachnospiraceae	g__Lachnospiraceae unknown	s__Lachnospiraceae unknown unknown_7

MGYG-HGUT-00380:s__Merdibacter unknown_1	0.068031341	-0.498051943	0.874618746	d__Bacteria	p__Firmicutes	c__Bacilli	o__Erysipelotrichales	f__Erysipelotrichaceae	g__Merdibacter	s__Merdibacter unknown_1
MGYG-HGUT-00387:s__Lachnospiraceae unknown unknown_8	0.22230344	0.329700918	0.891825559	d__Bacteria	p__Firmicutes_A	c__Clostridia	o__Lachnospirales	f__Lachnospiraceae	g__Lachnospiraceae unknown	s__Lachnospiraceae unknown unknown_8
MGYG-HGUT-00389:s__Roseburia unknown_2	0.412496761	0.228198237	0.93970925	d__Bacteria	p__Firmicutes_A	c__Clostridia	o__Lachnospirales	f__Lachnospiraceae	g__Roseburia	s__Roseburia unknown_2
MGYG-HGUT-00398:s__TF01-11 unknown_1	0.090639609	0.457319174	0.883307256	d__Bacteria	p__Firmicutes_A	c__Clostridia	o__Lachnospirales	f__Lachnospiraceae	g__TF01-11	s__TF01-11 unknown_1
MGYG-HGUT-00402:s__GCA-900066995 unknown	0.888290363	-0.038532113	0.994479474	d__Bacteria	p__Firmicutes_A	c__Clostridia	o__Oscillospirales	f__Acutalibacteraceae	g__GCA-900066995	s__GCA-900066995 unknown
MGYG-HGUT-00404:s__UBA11774 sp003507655	0.874917596	0.043992619	0.994479474	d__Bacteria	p__Firmicutes_A	c__Clostridia	o__Lachnospirales	f__Lachnospiraceae	g__UBA11774	s__UBA11774 sp003507655
MGYG-HGUT-00409:s__QALS01 sp003150575	0.431110322	-0.211954513	0.950806594	d__Bacteria	p__Firmicutes_A	c__Clostridia	o__4C28d-15	f__CAG-727	g__QALS01	s__QALS01 sp003150575
MGYG-HGUT-00413:s__UBA7185 unknown_2	0.933804907	-0.023172121	0.994479474	d__Bacteria	p__Firmicutes_B	c__Peptococcia	o__Peptococcales	f__Peptococcaceae	g__UBA7185	s__UBA7185 unknown_2
MGYG-HGUT-00414:s__Alistipes unknown_2	0.24512	0.32134	0.90218	d__Bact	p__Bacteroidota	c__Bacteroidia	o__Bacteroidales	f__Rikenellaceae	g__Alistipes	s__Alistipes unknown_2

	8306	6263	1367	eria						
MGYG-HGUT-00415:s__UBA11471 unknown_1	0.442789744	0.212323933	0.960273389	d__Bacteria	p__Bacteroidota	c__Bacteroidia	o__Bacteroidales	f__UBA11471	g__UBA11471	s__UBA11471 unknown_1
MGYG-HGUT-00417:s__Acetatifactor unknown_1	0.046362669	0.550138447	0.799667098	d__Bacteria	p__Firmicutes_A	c__Clostridia	o__Lachnospirales	f__Lachnospiraceae	g__Acetatifactor	s__Acetatifactor unknown_1
MGYG-HGUT-00420:s__Lachnospirales unknown unknown unknown_1	0.989766562	0.003571553	0.998803346	d__Bacteria	p__Firmicutes_A	c__Clostridia	o__Lachnospirales	f__Lachnospirales unknown	g__Lachnospirales unknown unknown	s__Lachnospirales unknown unknown unknown_1
MGYG-HGUT-00425:s__Ruminococcaceae unknown unknown_1	0.66547189	0.119154058	0.975671757	d__Bacteria	p__Firmicutes_A	c__Clostridia	o__Oscillospirales	f__Ruminococcaceae	g__Ruminococcaceae unknown	s__Ruminococcaceae unknown unknown_1
MGYG-HGUT-00429:s__UBA7597 unknown_1	0.482435377	0.193380704	0.968532073	d__Bacteria	p__Firmicutes_A	c__Clostridia	o__4C28d-15	f__CAG-727	g__UBA7597	s__UBA7597 unknown_1
MGYG-HGUT-00433:s__UBA1737 unknown	0.113997337	0.437076004	0.883307256	d__Bacteria	p__Firmicutes	c__Bacilli	o__ML615J-28	f__CAG-313	g__UBA1737	s__UBA1737 unknown
MGYG-HGUT-00437:s__Alistipes sp001941065	0.193526233	0.347038923	0.891825559	d__Bacteria	p__Bacteroidota	c__Bacteroidia	o__Bacteroidales	f__Rikenellaceae	g__Alistipes	s__Alistipes sp001941065
MGYG-HGUT-00453:s__Eubacterium_R unknown_3	0.987747733	-0.004142683	0.998803346	d__Bacteria	p__Firmicutes_A	c__Clostridia	o__Oscillospirales	f__Acutalibacteraceae	g__Eubacterium_R	s__Eubacterium_R unknown_3
MGYG-HGUT-00456:s__Acutalibacteraceae unknown unknown_3	0.637188902	-0.1318568	0.975671757	d__Bacteria	p__Firmicutes_A	c__Clostridia	o__Oscillospirales	f__Acutalibacteraceae	g__Acutalibacteraceae unknown	s__Acutalibacteraceae unknown unknown_3

MGYG-HGUT-00457:s__UBA737 unknown_2	0.618315655	0.139151133	0.975671757	d__Bacteria	p__Firmicutes_A	c__Clostridia	o__Oscillospirales	f__Acutalibacteraceae	g__UBA737	s__UBA737 unknown_2
MGYG-HGUT-00468:s__CAG-272 unknown unknown_4	0.476146749	0.200070603	0.968532073	d__Bacteria	p__Firmicutes_A	c__Clostridia	o__Oscillospirales	f__CAG-272	g__CAG-272 unknown	s__CAG-272 unknown unknown_4
MGYG-HGUT-00474:s__Oscillibacter unknown_2	0.529304114	0.160867446	0.968532073	d__Bacteria	p__Firmicutes_A	c__Clostridia	o__Oscillospirales	f__Oscillospiraceae	g__Oscillibacter	s__Oscillibacter unknown_2
MGYG-HGUT-00476:s__CAG-382 unknown unknown_2	0.760533461	0.083601728	0.979458475	d__Bacteria	p__Firmicutes_A	c__Clostridia	o__Oscillospirales	f__CAG-382	g__CAG-382 unknown	s__CAG-382 unknown unknown_2
MGYG-HGUT-00484:s__Butyrivibrio_A sp000431815	0.335791163	0.268580905	0.930624692	d__Bacteria	p__Firmicutes_A	c__Clostridia	o__Lachnospirales	f__Lachnospiraceae	g__Butyrivibrio_A	s__Butyrivibrio_A sp000431815
MGYG-HGUT-00486:s__Phascolarctobacterium_A unknown_3	0.759596397	0.085832208	0.979458475	d__Bacteria	p__Firmicutes_C	c__Negativicutes	o__Acidaminococcales	f__Acidaminococcaceae	g__Phascolarctobacterium_A	s__Phascolarctobacterium_A unknown_3
MGYG-HGUT-00489:s__Agathobacter unknown_1	0.288786273	0.275187869	0.929025913	d__Bacteria	p__Firmicutes_A	c__Clostridia	o__Lachnospirales	f__Lachnospiraceae	g__Agathobacter	s__Agathobacter unknown_1
MGYG-HGUT-00500:s__CAG-110 unknown_1	0.020541997	0.626571353	0.768225834	d__Bacteria	p__Firmicutes_A	c__Clostridia	o__Oscillospirales	f__Oscillospiraceae	g__CAG-110	s__CAG-110 unknown_1
MGYG-HGUT-00501:s__CAG-127 unknown_1	0.236331051	-0.327068822	0.902181367	d__Bacteria	p__Firmicutes_A	c__Clostridia	o__Lachnospirales	f__Lachnospiraceae	g__CAG-127	s__CAG-127 unknown_1

MGYG-HGUT-00505:s__Acutalibacteraceae unknown unknown_4	0.196887182	0.360436824	0.891825559	d__Bacteria	p__Firmicutes_A	c__Clostridia	o__Oscillospirales	f__Acutalibacteraceae	g__Acutalibacteraceae unknown	s__Acutalibacteraceae unknown unknown_4
MGYG-HGUT-00512:s__Faecalibacterium unknown_1	0.671316056	0.118465545	0.975671757	d__Bacteria	p__Firmicutes_A	c__Clostridia	o__Oscillospirales	f__Ruminococcaceae	g__Faecalibacterium	s__Faecalibacterium unknown_1
MGYG-HGUT-00514:s__CAG-590 unknown_1	0.402056939	0.234786823	0.93970925	d__Bacteria	p__Firmicutes_A	c__Clostridia	o__Lachnospirales	f__Lachnospiraceae	g__CAG-590	s__CAG-590 unknown_1
MGYG-HGUT-00515:s__UBA2882 unknown_1	0.757981111	-0.086190047	0.979458475	d__Bacteria	p__Firmicutes_A	c__Clostridia	o__Lachnospirales	f__Lachnospiraceae	g__UBA2882	s__UBA2882 unknown_1
MGYG-HGUT-00522:s__Methanobrevibacter_A unknown_1	0.167563389	0.385780909	0.891825559	d__Archaea	p__Euryarchaeota	c__Methanobacteria	o__Methanobacteriales	f__Methanobacteriaceae	g__Methanobrevibacter_A	s__Methanobrevibacter_A unknown_1
MGYG-HGUT-00530:s__PeH17 sp000435055	0.761735859	-0.078042141	0.979458475	d__Bacteria	p__Firmicutes_A	c__Clostridia	o__Christensenellales	f__CAG-138	g__PeH17	s__PeH17 sp000435055
MGYG-HGUT-00531:s__Collinsella unknown_28	0.752065702	0.088116873	0.979458475	d__Bacteria	p__Actinobacteriota	c__Coriobacteriia	o__Coriobacteriales	f__Coriobacteriaceae	g__Collinsella	s__Collinsella unknown_28
MGYG-HGUT-00532:s__CAG-510 sp000434615	0.029675761	0.59835872	0.768225834	d__Bacteria	p__Firmicutes_A	c__Clostridia	o__Lachnospirales	f__Lachnospiraceae	g__CAG-510	s__CAG-510 sp000434615
MGYG-HGUT-00534:s__Prevotella unknown_2	0.543405004	-0.167546251	0.968532073	d__Bacteria	p__Bacteroidota	c__Bacteroidia	o__Bacteroidales	f__Bacteroidaceae	g__Prevotella	s__Prevotella unknown_2

MGYG-HGUT-00537:s__Ruminococcaceae unknown unknown_6	0.688641653	0.111672373	0.975768579	d__Bacteria	p__Firmicutes_A	c__Clostridia	o__Oscillospirales	f__Ruminococcaceae	g__Ruminococcaceae unknown	s__Ruminococcaceae unknown unknown_6
MGYG-HGUT-00543:s__Agathobacter unknown_2	0.332809935	0.266186007	0.930624692	d__Bacteria	p__Firmicutes_A	c__Clostridia	o__Lachnospirales	f__Lachnospiraceae	g__Agathobacter	s__Agathobacter unknown_2
MGYG-HGUT-00544:s__Zag1 unknown_4	0.539051543	0.171644741	0.968532073	d__Bacteria	p__Cyanobacteria	c__Vampirovibrionia	o__Gastranaerophilales	f__Gastranaerophilaceae	g__Zag1	s__Zag1 unknown_4
MGYG-HGUT-00545:s__Faecalicatena unknown_1	0.949251227	0.017807645	0.994479474	d__Bacteria	p__Firmicutes_A	c__Clostridia	o__Lachnospirales	f__Lachnospiraceae	g__Faecalicatena	s__Faecalicatena unknown_1
MGYG-HGUT-00546:s__Roseburia sp003483745	0.419813219	0.224124933	0.941287606	d__Bacteria	p__Firmicutes_A	c__Clostridia	o__Lachnospirales	f__Lachnospiraceae	g__Roseburia	s__Roseburia sp003483745
MGYG-HGUT-00547:s__Catenibacterium unknown_1	0.710901008	-0.102920804	0.979458475	d__Bacteria	p__Firmicutes	c__Bacilli	o__Erysipelotrichales	f__Erysipelatoclostridiaceae	g__Catenibacterium	s__Catenibacterium unknown_1
MGYG-HGUT-00550:s__Lawsonibacter sp002160305	0.401402094	-0.228417376	0.93970925	d__Bacteria	p__Firmicutes_A	c__Clostridia	o__Oscillospirales	f__Oscillospiraceae	g__Lawsonibacter	s__Lawsonibacter sp002160305
MGYG-HGUT-00553:s__Prevotella unknown_3	0.794670227	0.070946358	0.98296663	d__Bacteria	p__Bacteroidota	c__Bacteroidia	o__Bacteroidales	f__Bacteroidaceae	g__Prevotella	s__Prevotella unknown_3
MGYG-HGUT-00556:s__Collinsella sp002232035_9	0.019330108	0.640001126	0.768225834	d__Bacteria	p__Actinobacteriota	c__Coriobacteriia	o__Coriobacteriales	f__Coriobacteriaceae	g__Collinsella	s__Collinsella sp002232035_9

MGYG-HGUT-00559:s__Paraprevotella unknown_1	0.779998684	0.077912163	0.979520484	d__Bacteria	p__Bacteroidota	c__Bacteroidia	o__Bacteroidales	f__Bacteroidaceae	g__Paraprevotella	s__Paraprevotella unknown_1
MGYG-HGUT-00562:s__Lachnospira sp000437735	0.165770577	-0.382816692	0.891825559	d__Bacteria	p__Firmicutes_A	c__Clostridia	o__Lachnospirales	f__Lachnospiraceae	g__Lachnospira	s__Lachnospira sp000437735
MGYG-HGUT-00563:s__Collinsella unknown_29	0.120994988	0.430538208	0.885742175	d__Bacteria	p__Actinobacteriota	c__Coriobacteriia	o__Coriobacteriales	f__Coriobacteriaceae	g__Collinsella	s__Collinsella unknown_29
MGYG-HGUT-00568:s__CAG-279 unknown_1	0.209532534	0.348967966	0.891825559	d__Bacteria	p__Bacteroidota	c__Bacteroidia	o__Bacteroidales	f__Muribaculaceae	g__CAG-279	s__CAG-279 unknown_1
MGYG-HGUT-00573:s__Gemmiger unknown_1	0.016727718	0.656662792	0.768225834	d__Bacteria	p__Firmicutes_A	c__Clostridia	o__Oscillospirales	f__Ruminococcaceae	g__Gemmiger	s__Gemmiger unknown_1
MGYG-HGUT-00574:s__Lachnospiraceae unknown unknown_9	0.002934632	0.801594538	0.648124372	d__Bacteria	p__Firmicutes_A	c__Clostridia	o__Lachnospirales	f__Lachnospiraceae	g__Lachnospiraceae unknown	s__Lachnospiraceae unknown unknown_9
MGYG-HGUT-00575:s__UBA11452 sp003526375	0.397332674	0.232996533	0.93970925	d__Bacteria	p__Verrucomicrobiota	c__Lentisphaeria	o__Victivallales	f__UBA1829	g__UBA11452	s__UBA11452 sp003526375
MGYG-HGUT-00576:s__Streptococcus unknown_4	0.412427731	0.218751076	0.93970925	d__Bacteria	p__Firmicutes	c__Bacilli	o__Lactobacillales	f__Streptococcaceae	g__Streptococcus	s__Streptococcus unknown_4
MGYG-HGUT-00580:s__Streptococcus vestibularis_1	0.821420823	0.062978677	0.991136622	d__Bacteria	p__Firmicutes	c__Bacilli	o__Lactobacillales	f__Streptococcaceae	g__Streptococcus	s__Streptococcus vestibularis_1

MGYG-HGUT-00583:s__Acetatifactor unknown_2	0.011584661	0.685976042	0.768225834	d__Bacteria	p__Firmicutes_A	c__Clostridia	o__Lachnospirales	f__Lachnospiraceae	g__Acetatifactor	s__Acetatifactor unknown_2
MGYG-HGUT-00584:s__RC9 unknown_2	0.326019656	0.272929125	0.930624692	d__Bacteria	p__Bacteroidota	c__Bacteroidia	o__Bacteroidales	f__UBA932	g__RC9	s__RC9 unknown_2
MGYG-HGUT-00589:s__Faecalibacterium unknown_2	0.145373859	0.402412885	0.891825559	d__Bacteria	p__Firmicutes_A	c__Clostridia	o__Oscillospirales	f__Ruminococcaceae	g__Faecalibacterium	s__Faecalibacterium unknown_2
MGYG-HGUT-00591:s__Phascolarctobacterium_A succinatutens_A	0.764209614	0.082366603	0.979520484	d__Bacteria	p__Firmicutes_C	c__Negativicutes	o__Acidaminococcales	f__Acidaminococcaceae	g__Phascolarctobacterium_A	s__Phascolarctobacterium_A succinatutens_A
MGYG-HGUT-00592:s__RC9 unknown_3	0.046539104	0.548418648	0.799667098	d__Bacteria	p__Bacteroidota	c__Bacteroidia	o__Bacteroidales	f__UBA932	g__RC9	s__RC9 unknown_3
MGYG-HGUT-00593:s__Collinsella unknown_32	0.859423112	-0.048655538	0.992878088	d__Bacteria	p__Actinobacteriota	c__Coriobacteriia	o__Coriobacteriales	f__Coriobacteriaceae	g__Collinsella	s__Collinsella unknown_32
MGYG-HGUT-00594:s__Collinsella unknown_33	0.01173867	-0.693296817	0.768225834	d__Bacteria	p__Actinobacteriota	c__Coriobacteriia	o__Coriobacteriales	f__Coriobacteriaceae	g__Collinsella	s__Collinsella unknown_33
MGYG-HGUT-00597:s__Senegalimassilia unknown_1	0.946933459	-0.018538154	0.994479474	d__Bacteria	p__Actinobacteriota	c__Coriobacteriia	o__Coriobacteriales	f__Eggerthellaceae	g__Senegalimassilia	s__Senegalimassilia unknown_1
MGYG-HGUT-00598:s__Collinsella unknown_34	0.353950062	0.253619194	0.93970925	d__Bacteria	p__Actinobacteriota	c__Coriobacteriia	o__Coriobacteriales	f__Coriobacteriaceae	g__Collinsella	s__Collinsella unknown_34

MGYG-HGUT-00599:s__Collinsella unknown_35	0.883401563	0.040949339	0.994479474	d__Bacteria	p__Actinobacteriota	c__Coriobacteriia	o__Coriobacteriales	f__Coriobacteriaceae	g__Collinsella	s__Collinsella unknown_35
MGYG-HGUT-00602:s__CAG-1427 unknown_4	0.980463649	-0.006811729	0.997647914	d__Bacteria	p__Actinobacteriota	c__Coriobacteriia	o__Coriobacteriales	f__Eggerthellaceae	g__CAG-1427	s__CAG-1427 unknown_4
MGYG-HGUT-00605:s__Ruminococcus_C unknown_1	0.436111038	0.213641634	0.950806594	d__Bacteria	p__Firmicutes_A	c__Clostridia	o__Oscillospirales	f__Ruminococcaceae	g__Ruminococcus_C	s__Ruminococcus_C unknown_1
MGYG-HGUT-00606:s__RC9 sp000433355	0.374112426	0.249155455	0.93970925	d__Bacteria	p__Bacteroidota	c__Bacteroidia	o__Bacteroidales	f__UBA932	g__RC9	s__RC9 sp000433355
MGYG-HGUT-00608:s__Victivallaceae unknown unknown_2	0.168529604	0.382614815	0.891825559	d__Bacteria	p__Verrucomicrobiota	c__Lentisphaeria	o__Victivallales	f__Victivallaceae	g__Victivallaceae unknown	s__Victivallaceae unknown unknown_2
MGYG-HGUT-00611:s__Senegalimassilia unknown_2	0.289736412	0.296134242	0.929025913	d__Bacteria	p__Actinobacteriota	c__Coriobacteriia	o__Coriobacteriales	f__Eggerthellaceae	g__Senegalimassilia	s__Senegalimassilia unknown_2
MGYG-HGUT-00621:s__Acetatifactor unknown_3	0.951945586	0.01657738	0.994479474	d__Bacteria	p__Firmicutes_A	c__Clostridia	o__Lachnospirales	f__Lachnospiraceae	g__Acetatifactor	s__Acetatifactor unknown_3
MGYG-HGUT-00623:s__Blautia_A unknown_6	0.006204228	0.732777986	0.750056424	d__Bacteria	p__Firmicutes_A	c__Clostridia	o__Lachnospirales	f__Lachnospiraceae	g__Blautia_A	s__Blautia_A unknown_6
MGYG-HGUT-00625:s__CAG-127 unknown_2	0.741002217	-0.091797623	0.979458475	d__Bacteria	p__Firmicutes_A	c__Clostridia	o__Lachnospirales	f__Lachnospiraceae	g__CAG-127	s__CAG-127 unknown_2

MGYG-HGUT-00626:s__Eubacterium_J unknown	0.932120181	0.022673575	0.994479474	d__Bacteria	p__Firmicutes_A	c__Clostridia	o__Lachnospirales	f__Lachnospiraceae	g__Eubacterium_J	s__Eubacterium_J unknown
MGYG-HGUT-00629:s__CAG-196 unknown_2	0.272270755	0.307436154	0.926757771	d__Bacteria	p__Cyanobacteria	c__Vampirovibrionia	o__Gastranaerophilales	f__Gastranaerophilaceae	g__CAG-196	s__CAG-196 unknown_2
MGYG-HGUT-00631:s__Prevotella sp002299635	0.888723613	-0.038790228	0.994479474	d__Bacteria	p__Bacteroidota	c__Bacteroidia	o__Bacteroidales	f__Bacteroidaceae	g__Prevotella	s__Prevotella sp002299635
MGYG-HGUT-00636:s__Faecalibacterium unknown_3	0.054225584	0.529127346	0.857211132	d__Bacteria	p__Firmicutes_A	c__Clostridia	o__Oscillospirales	f__Ruminococcaceae	g__Faecalibacterium	s__Faecalibacterium unknown_3
MGYG-HGUT-00638:s__Lachnospira unknown_1	0.198246896	0.32433313	0.891825559	d__Bacteria	p__Firmicutes_A	c__Clostridia	o__Lachnospirales	f__Lachnospiraceae	g__Lachnospira	s__Lachnospira unknown_1
MGYG-HGUT-00641:s__Collinsella unknown_36	0.867084767	0.04692657	0.994479474	d__Bacteria	p__Actinobacteriota	c__Coriobacteriia	o__Coriobacteriales	f__Coriobacteriaceae	g__Collinsella	s__Collinsella unknown_36
MGYG-HGUT-00643:s__Senegalimassilia unknown_3	0.376664155	0.242247917	0.93970925	d__Bacteria	p__Actinobacteriota	c__Coriobacteriia	o__Coriobacteriales	f__Eggerthellaceae	g__Senegalimassilia	s__Senegalimassilia unknown_3
MGYG-HGUT-00644:s__Prevotella unknown_4	0.758584816	0.082857609	0.979458475	d__Bacteria	p__Bacteroidota	c__Bacteroidia	o__Bacteroidales	f__Bacteroidaceae	g__Prevotella	s__Prevotella unknown_4
MGYG-HGUT-00645:s__Collinsella unknown_37	0.448209268	-0.207957993	0.961292208	d__Bacteria	p__Actinobacteriota	c__Coriobacteriia	o__Coriobacteriales	f__Coriobacteriaceae	g__Collinsella	s__Collinsella unknown_37

MGYG-HGUT-00646:s__Eubacterium_R sp000436835	0.245681516	-0.323834078	0.902181367	d__Bacteria	p__Firmicutes_A	c__Clostridia	o__Oscillospirales	f__Acutalibacteraceae	g__Eubacterium_R	s__Eubacterium_R sp000436835
MGYG-HGUT-00650:s__Clostridium_M unknown_2	0.746252704	0.090857837	0.979458475	d__Bacteria	p__Firmicutes_A	c__Clostridia	o__Lachnospirales	f__Lachnospiraceae	g__Clostridium_M	s__Clostridium_M unknown_2
MGYG-HGUT-00651:s__CAG-170 sp002404795	0.165996798	0.374451989	0.891825559	d__Bacteria	p__Firmicutes_A	c__Clostridia	o__Oscillospirales	f__Oscillospiraceae	g__CAG-170	s__CAG-170 sp002404795
MGYG-HGUT-00652:s__Bacteroides togonis	0.535618991	0.173756292	0.968532073	d__Bacteria	p__Bacteroidota	c__Bacteroidia	o__Bacteroidales	f__Bacteroidaceae	g__Bacteroides	s__Bacteroides togonis
MGYG-HGUT-00653:s__UBA11471 sp000434215	0.050067713	-0.532838413	0.84406607	d__Bacteria	p__Bacteroidota	c__Bacteroidia	o__Bacteroidales	f__UBA11471	g__UBA11471	s__UBA11471 sp000434215
MGYG-HGUT-00655:s__Holdemanella unknown_2	0.798695312	0.070822396	0.985682681	d__Bacteria	p__Firmicutes	c__Bacilli	o__Erysipelotrichales	f__Erysipelotrichaceae	g__Holdemanella	s__Holdemanella unknown_2
MGYG-HGUT-00656:s__CAG-269 unknown_7	0.611801513	0.142310093	0.975671757	d__Bacteria	p__Firmicutes_A	c__Clostridia	o__TANB77	f__CAG-508	g__CAG-269	s__CAG-269 unknown_7
MGYG-HGUT-00660:s__Collinsella unknown_40	0.280558887	0.300670113	0.929025913	d__Bacteria	p__Actinobacteriota	c__Coriobacteriia	o__Coriobacteriales	f__Coriobacteriaceae	g__Collinsella	s__Collinsella unknown_40
MGYG-HGUT-00667:s__UBA7173 unknown_3	0.926531277	-0.025893857	0.994479474	d__Bacteria	p__Bacteroidota	c__Bacteroidia	o__Bacteroidales	f__Muribaculaceae	g__UBA7173	s__UBA7173 unknown_3

MGYG-HGUT-00668:s__CAG-145 unknown_1	0.775158268	-0.079352098	0.979520484	d__Bacteria	p__Firmicutes_A	c__Clostridia	o__Peptostreptococcales	f__Anaerovoracaceae	g__CAG-145	s__CAG-145 unknown_1
MGYG-HGUT-00669:s__CAG-488 sp000434055	0.529160623	0.174336653	0.968532073	d__Bacteria	p__Firmicutes_A	c__Clostridia	o__Oscillospirales	f__Acutalibacteraceae	g__CAG-488	s__CAG-488 sp000434055
MGYG-HGUT-00670:s__Erysipelatoclostridium unknown_2	0.792352831	0.073331988	0.98296663	d__Bacteria	p__Firmicutes	c__Bacilli	o__Erysipelotrichales	f__Erysipelatoclostridiaceae	g__Erysipelatoclostridium	s__Erysipelatoclostridium unknown_2
MGYG-HGUT-00671:s__Dialister sp002320515	0.635933577	0.132446262	0.975671757	d__Bacteria	p__Firmicutes_C	c__Negativicutes	o__Veillonellales	f__Dialisteraceae	g__Dialister	s__Dialister sp002320515
MGYG-HGUT-00672:s__RC9 sp000431015	0.43170997	0.218249367	0.950806594	d__Bacteria	p__Bacteroidota	c__Bacteroidia	o__Bacteroidales	f__UBA932	g__RC9	s__RC9 sp000431015
MGYG-HGUT-00674:s__Monoglobales unknown unknown unknown_2	0.89073061	-0.037714784	0.994479474	d__Bacteria	p__Firmicutes_A	c__Clostridia	o__Monoglobales	f__Monoglobales unknown	g__Monoglobales unknown unknown	s__Monoglobales unknown unknown unknown_2
MGYG-HGUT-00675:s__Bacteroides congonensis	0.228233639	-0.337180893	0.902181367	d__Bacteria	p__Bacteroidota	c__Bacteroidia	o__Bacteroidales	f__Bacteroidaceae	g__Bacteroides	s__Bacteroides congonensis
MGYG-HGUT-00680:s__Hungatella_A unknown_2	0.527901945	0.177125423	0.968532073	d__Bacteria	p__Firmicutes_A	c__Clostridia	o__Lachnospirales	f__Lachnospiraceae	g__Hungatella_A	s__Hungatella_A unknown_2
MGYG-HGUT-00681:s__Lactococcus raffinolactis	0.995498211	0.001574414	0.998803346	d__Bacteria	p__Firmicutes	c__Bacilli	o__Lactobacillales	f__Streptococcaceae	g__Lactococcus	s__Lactococcus raffinolactis

MGYG-HGUT-00682:s__UBA4334 unknown	0.642362648	0.124627837	0.975671757	d__Bacteria	p__Bacteroidota	c__Bacteroidia	o__Bacteroidales	f__Bacteroidaceae	g__UBA4334	s__UBA4334 unknown
MGYG-HGUT-00685:s__CAG-590 unknown_2	0.127194887	0.423437508	0.891825559	d__Bacteria	p__Firmicutes_A	c__Clostridia	o__Lachnospirales	f__Lachnospiraceae	g__CAG-590	s__CAG-590 unknown_2
MGYG-HGUT-00687:s__Anaerostipes unknown_1	0.217463528	0.332424012	0.891825559	d__Bacteria	p__Firmicutes_A	c__Clostridia	o__Lachnospirales	f__Lachnospiraceae	g__Anaerostipes	s__Anaerostipes unknown_1
MGYG-HGUT-00689:s__Angelakisella sp003453215	0.369794564	0.249170585	0.93970925	d__Bacteria	p__Firmicutes_A	c__Clostridia	o__Oscillospirales	f__Ruminococcaceae	g__Angelakisella	s__Angelakisella sp003453215
MGYG-HGUT-00690:s__CAG-590 sp000431135	0.836109424	-0.056578878	0.992775775	d__Bacteria	p__Firmicutes_A	c__Clostridia	o__Lachnospirales	f__Lachnospiraceae	g__CAG-590	s__CAG-590 sp000431135
MGYG-HGUT-00691:s__CAG-110 unknown_2	0.683611218	-0.113570364	0.975768579	d__Bacteria	p__Firmicutes_A	c__Clostridia	o__Oscillospirales	f__Oscillospiraceae	g__CAG-110	s__CAG-110 unknown_2
MGYG-HGUT-00692:s__Prevotellamassilia unknown_2	0.628210055	0.12915182	0.975671757	d__Bacteria	p__Bacteroidota	c__Bacteroidia	o__Bacteroidales	f__Bacteroidaceae	g__Prevotellamassilia	s__Prevotellamassilia unknown_2
MGYG-HGUT-00695:s__Prevotella sp000436035	0.607842287	0.139407801	0.975671757	d__Bacteria	p__Bacteroidota	c__Bacteroidia	o__Bacteroidales	f__Bacteroidaceae	g__Prevotella	s__Prevotella sp000436035
MGYG-HGUT-00696:s__Parabacteroides unknown_5	0.461350316	0.201843922	0.965574692	d__Bacteria	p__Bacteroidota	c__Bacteroidia	o__Bacteroidales	f__Tannerellaceae	g__Parabacteroides	s__Parabacteroides unknown_5

MGYG-HGUT-00697:s__CAG-488 unknown_1	0.203913667	0.3517867	0.891825559	d__Bacteria	p__Firmicutes_A	c__Clostridia	o__Oscillospirales	f__Acutalibacteraceae	g__CAG-488	s__CAG-488 unknown_1
MGYG-HGUT-00699:s__Solobacterium unknown_2	0.655549618	-0.122394457	0.975671757	d__Bacteria	p__Firmicutes	c__Bacilli	o__Erysipelotrichales	f__Erysipelotrichaceae	g__Solobacterium	s__Solobacterium unknown_2
MGYG-HGUT-00701:s__Prevotella unknown_5	0.939676473	0.020817628	0.994479474	d__Bacteria	p__Bacteroidota	c__Bacteroidia	o__Bacteroidales	f__Bacteroidaceae	g__Prevotella	s__Prevotella unknown_5
MGYG-HGUT-00702:s__Collinsella unknown_45	0.313390407	0.277896859	0.929025913	d__Bacteria	p__Actinobacteriota	c__Coriobacteriia	o__Coriobacteriales	f__Coriobacteriaceae	g__Collinsella	s__Collinsella unknown_45
MGYG-HGUT-00703:s__CAG-83 unknown_1	0.163101778	0.365001366	0.891825559	d__Bacteria	p__Firmicutes_A	c__Clostridia	o__Oscillospirales	f__Oscillospiraceae	g__CAG-83	s__CAG-83 unknown_1
MGYG-HGUT-00707:s__Prevotella unknown_6	0.368181788	-0.247620295	0.93970925	d__Bacteria	p__Bacteroidota	c__Bacteroidia	o__Bacteroidales	f__Bacteroidaceae	g__Prevotella	s__Prevotella unknown_6
MGYG-HGUT-00708:s__Succinivibrio unknown_1	0.769032014	0.082377753	0.979520484	d__Bacteria	p__Proteobacteria	c__Gammaproteobacteria	o__Enterobacterales	f__Succinivibrionaceae	g__Succinivibrio	s__Succinivibrio unknown_1
MGYG-HGUT-00709:s__UBA738 unknown_1	0.991960165	-0.0027763	0.998803346	d__Bacteria	p__Firmicutes_A	c__Clostridia	o__Oscillospirales	f__Oscillospiraceae	g__UBA738	s__UBA738 unknown_1
MGYG-HGUT-00710:s__CAG-110 unknown_3	0.769021591	-0.080647064	0.979520484	d__Bacteria	p__Firmicutes_A	c__Clostridia	o__Oscillospirales	f__Oscillospiraceae	g__CAG-110	s__CAG-110 unknown_3

MGYG-HGUT-00711:s__CAG-145 unknown_2	0.003869035	0.788671851	0.648124372	d__Bacteria	p__Firmicutes_A	c__Clostridia	o__Peptostreptococcales	f__Anaerovoracaceae	g__CAG-145	s__CAG-145 unknown_2
MGYG-HGUT-00713:s__CAG-83 unknown_2	0.054409182	0.510991855	0.85721132	d__Bacteria	p__Firmicutes_A	c__Clostridia	o__Oscillospirales	f__Oscillospiraceae	g__CAG-83	s__CAG-83 unknown_2
MGYG-HGUT-00715:s__CAG-110 unknown_4	0.779871482	0.075400507	0.979520484	d__Bacteria	p__Firmicutes_A	c__Clostridia	o__Oscillospirales	f__Oscillospiraceae	g__CAG-110	s__CAG-110 unknown_4
MGYG-HGUT-00718:s__CAG-138 unknown unknown_1	0.657007133	0.117578932	0.975671757	d__Bacteria	p__Firmicutes_A	c__Clostridia	o__Christensenellales	f__CAG-138	g__CAG-138 unknown	s__CAG-138 unknown unknown_1
MGYG-HGUT-00721:s__NC2004 unknown	0.926061195	0.025995632	0.994479474	d__Bacteria	p__Firmicutes_A	c__Clostridia	o__Lachnospirales	f__Lachnospiraceae	g__NC2004	s__NC2004 unknown
MGYG-HGUT-00723:s__CAG-177 sp003514385	0.702093251	-0.104893518	0.979458475	d__Bacteria	p__Firmicutes_A	c__Clostridia	o__Oscillospirales	f__Acutalibacteraceae	g__CAG-177	s__CAG-177 sp003514385
MGYG-HGUT-00724:s__Gemmiger unknown_2	0.12530642	0.425905679	0.891825559	d__Bacteria	p__Firmicutes_A	c__Clostridia	o__Oscillospirales	f__Ruminococcaceae	g__Gemmiger	s__Gemmiger unknown_2
MGYG-HGUT-00725:s__Dialister sp000434475	0.128972959	0.410922973	0.891825559	d__Bacteria	p__Firmicutes_C	c__Negativicutes	o__Veillonellales	f__Dialisteraceae	g__Dialister	s__Dialister sp000434475
MGYG-HGUT-00726:s__CAG-180 unknown_1	0.910945829	0.029833431	0.994479474	d__Bacteria	p__Firmicutes_A	c__Clostridia	o__Oscillospirales	f__Acutalibacteraceae	g__CAG-180	s__CAG-180 unknown_1

MGYG-HGUT-00728:s__UBA1255 unknown unknown_3	0.744677878	-0.0888854286	0.979458475	d__Bacteria	p__Firmicutes_A	c__Clostridia	o__UBA1212	f__UBA1255	g__UBA1255 unknown	s__UBA1255 unknown unknown_3
MGYG-HGUT-00729:s__F23-B02 unknown_2	0.287797829	0.287226149	0.929025913	d__Bacteria	p__Firmicutes_A	c__Clostridia	o__Oscillospirales	f__Oscillospiraceae	g__F23-B02	s__F23-B02 unknown_2
MGYG-HGUT-00730:s__Phil1 sp001940855	0.36118065	-0.246767858	0.93970925	d__Bacteria	p__Firmicutes_A	c__Clostridia	o__Christensenellales	f__CAG-138	g__Phil1	s__Phil1 sp001940855
MGYG-HGUT-00732:s__CAG-245 unknown_1	0.721458036	-0.099099463	0.979458475	d__Bacteria	p__Firmicutes_A	c__Clostridia	o__TANB77	f__CAG-508	g__CAG-245	s__CAG-245 unknown_1
MGYG-HGUT-00735:s__UBA738 unknown_2	0.401791405	0.228750615	0.93970925	d__Bacteria	p__Firmicutes_A	c__Clostridia	o__Oscillospirales	f__Oscillospiraceae	g__UBA738	s__UBA738 unknown_2
MGYG-HGUT-00741:s__CAG-170 unknown_2	0.015734344	0.639460275	0.768225834	d__Bacteria	p__Firmicutes_A	c__Clostridia	o__Oscillospirales	f__Oscillospiraceae	g__CAG-170	s__CAG-170 unknown_2
MGYG-HGUT-00743:s__CAG-110 sp000434635	0.714687221	-0.099226199	0.979458475	d__Bacteria	p__Firmicutes_A	c__Clostridia	o__Oscillospirales	f__Oscillospiraceae	g__CAG-110	s__CAG-110 sp000434635
MGYG-HGUT-00746:s__UBA1777 unknown_1	0.7846083	-0.074343032	0.981194211	d__Bacteria	p__Firmicutes_A	c__Clostridia	o__Oscillospirales	f__Oscillospiraceae	g__UBA1777	s__UBA1777 unknown_1
MGYG-HGUT-00752:s__UBA737 unknown_4	0.94454	-0.019	0.99447	d__Bact	p__Firmicutes_A	c__Clostridia	o__Oscillospirales	f__Acutalibacteraceae	g__UBA737	s__UBA737 unknown_4

	799	459627	9474	eria						
MGYG-HGUT-00753:s__ER4 unknown_1	0.391792035	0.229303995	0.939709925	d__Bacteria	p__Firmicutes_A	c__Clostridia	o__Oscillospirales	f__Oscillospiraceae	g__ER4	s__ER4 unknown_1
MGYG-HGUT-00754:s__Streptococcus unknown_5	0.711061754	0.104129326	0.979458475	d__Bacteria	p__Firmicutes	c__Bacilli	o__Lactobacillales	f__Streptococcaceae	g__Streptococcus	s__Streptococcus unknown_5
MGYG-HGUT-00756:s__Bifidobacterium sp002742445	0.63772364	0.128070114	0.975671757	d__Bacteria	p__Actinobacteriota	c__Actinobacteria	o__Actinomycetales	f__Bifidobacteriaceae	g__Bifidobacterium	s__Bifidobacterium sp002742445
MGYG-HGUT-00759:s__Corynebacterium unknown_1	0.073668735	0.486085357	0.877826247	d__Bacteria	p__Actinobacteriota	c__Actinobacteria	o__Mycobacteriales	f__Mycobacteriaceae	g__Corynebacterium	s__Corynebacterium unknown_1
MGYG-HGUT-00768:s__Collinsella unknown_50	0.053599095	0.528204591	0.85721132	d__Bacteria	p__Actinobacteriota	c__Coriobacteriia	o__Coriobacteriales	f__Coriobacteriaceae	g__Collinsella	s__Collinsella unknown_50
MGYG-HGUT-00769:s__Collinsella unknown_51	0.030736219	0.577697806	0.768225834	d__Bacteria	p__Actinobacteriota	c__Coriobacteriia	o__Coriobacteriales	f__Coriobacteriaceae	g__Collinsella	s__Collinsella unknown_51
MGYG-HGUT-00770:s__Collinsella unknown_52	0.033749627	0.586629633	0.768225834	d__Bacteria	p__Actinobacteriota	c__Coriobacteriia	o__Coriobacteriales	f__Coriobacteriaceae	g__Collinsella	s__Collinsella unknown_52
MGYG-HGUT-00771:s__Lactococcus lactis_E	0.977068733	0.008015408	0.996587801	d__Bacteria	p__Firmicutes	c__Bacilli	o__Lactobacillales	f__Streptococcaceae	g__Lactococcus	s__Lactococcus lactis_E
MGYG-HGUT-00774:s__Clostridium_M unknown_3	0.865015043	-0.04708	0.994479474	d__Bacteria	p__Firmicutes_A	c__Clostridia	o__Lachnospirales	f__Lachnospiraceae	g__Clostridium_M	s__Clostridium_M unknown_3

		9988								
MGYG-HGUT-00777:s__Collinsella unknown_54	0.024767347	0.611925152	0.768225834	d__Bacteria	p__Actinobacteriota	c__Coriobacteriia	o__Coriobacteriales	f__Coriobacteriaceae	g__Collinsella	s__Collinsella unknown_54
MGYG-HGUT-00778:s__Collinsella unknown_55	0.006610777	0.722667544	0.750056424	d__Bacteria	p__Actinobacteriota	c__Coriobacteriia	o__Coriobacteriales	f__Coriobacteriaceae	g__Collinsella	s__Collinsella unknown_55
MGYG-HGUT-00779:s__Prevotella sp000431975	0.328002449	-0.256920093	0.930624692	d__Bacteria	p__Bacteroidota	c__Bacteroidia	o__Bacteroidales	f__Bacteroidaceae	g__Prevotella	s__Prevotella sp000431975
MGYG-HGUT-00780:s__Collinsella unknown_56	0.372255517	0.241662619	0.93970925	d__Bacteria	p__Actinobacteriota	c__Coriobacteriia	o__Coriobacteriales	f__Coriobacteriaceae	g__Collinsella	s__Collinsella unknown_56
MGYG-HGUT-00784:s__CAG-417 sp000432835	0.742300393	-0.091921462	0.979458475	d__Bacteria	p__Firmicutes	c__Bacilli	o__RF39	f__CAG-611	g__CAG-417	s__CAG-417 sp000432835
MGYG-HGUT-00787:s__Collinsella sp002232035_11	0.375474888	0.24688324	0.93970925	d__Bacteria	p__Actinobacteriota	c__Coriobacteriia	o__Coriobacteriales	f__Coriobacteriaceae	g__Collinsella	s__Collinsella sp002232035_11
MGYG-HGUT-00788:s__Bacteroides pyogenes	0.563803086	-0.161466147	0.97133903	d__Bacteria	p__Bacteroidota	c__Bacteroidia	o__Bacteroidales	f__Bacteroidaceae	g__Bacteroides	s__Bacteroides pyogenes
MGYG-HGUT-00791:s__CAG-269 unknown_9	0.833618224	-0.058930877	0.99222019	d__Bacteria	p__Firmicutes_A	c__Clostridia	o__TANB77	f__CAG-508	g__CAG-269	s__CAG-269 unknown_9

MGYG-HGUT-00793:s__Succinivibrio unknown_2	0.920752258	0.0278892	0.994479474	d__Bacteria	p__Proteobacteria	c__Gammaproteobacteria	o__Enterobacterales	f__Succinivibrionaceae	g__Succinivibrio	s__Succinivibrio unknown_2
MGYG-HGUT-00795:s__Collinsella unknown_57	0.118079646	-0.435073984	0.883307256	d__Bacteria	p__Actinobacteriota	c__Coriobacteriia	o__Coriobacteriales	f__Coriobacteriaceae	g__Collinsella	s__Collinsella unknown_57
MGYG-HGUT-00797:s__Collinsella unknown_58	0.824238656	0.061431111	0.991148931	d__Bacteria	p__Actinobacteriota	c__Coriobacteriia	o__Coriobacteriales	f__Coriobacteriaceae	g__Collinsella	s__Collinsella unknown_58
MGYG-HGUT-00800:s__Fusobacterium_B unknown_2	0.831415725	0.05972853	0.99222019	d__Bacteria	p__Fusobacteriota	c__Fusobacteriia	o__Fusobacteriales	f__Fusobacteriaceae	g__Fusobacterium_B	s__Fusobacterium_B unknown_2
MGYG-HGUT-00801:s__Collinsella sp002232035_13	0.736568744	-0.093770464	0.979458475	d__Bacteria	p__Actinobacteriota	c__Coriobacteriia	o__Coriobacteriales	f__Coriobacteriaceae	g__Collinsella	s__Collinsella sp002232035_13
MGYG-HGUT-00805:s__Collinsella unknown_62	0.598954991	0.146953971	0.975671757	d__Bacteria	p__Actinobacteriota	c__Coriobacteriia	o__Coriobacteriales	f__Coriobacteriaceae	g__Collinsella	s__Collinsella unknown_62
MGYG-HGUT-00806:s__Blautia_A unknown_7	0.630315809	-0.134811965	0.975671757	d__Bacteria	p__Firmicutes_A	c__Clostridia	o__Lachnospirales	f__Lachnospiraceae	g__Blautia_A	s__Blautia_A unknown_7
MGYG-HGUT-00807:s__Anaerofustis unknown	0.801070136	-0.070218926	0.987142302	d__Bacteria	p__Firmicutes_A	c__Clostridia	o__Eubacteriales	f__Anaerofustaceae	g__Anaerofustis	s__Anaerofustis unknown
MGYG-HGUT-00808:s__Oscillibacter unknown_3	0.38861	0.23575	0.93970	d__Bact	p__Firmicutes_A	c__Clostridia	o__Oscillospirales	f__Oscillospiraceae	g__Oscillibacter	s__Oscillibacter unknown_3

	087	4841	925	eria						
MGYG-HGUT-00811:s__Collinsella unknown_63	0.110968362	0.444040825	0.883307256	d__Bacteria	p__Actinobacteriota	c__Coriobacteriia	o__Coriobacteriales	f__Coriobacteriaceae	g__Collinsella	s__Collinsella unknown_63
MGYG-HGUT-00817:s__Catenibacterium unknown_2	0.95539079	-0.015396603	0.994479474	d__Bacteria	p__Firmicutes	c__Bacilli	o__Erysipelotrichales	f__Erysipelatoclostridiaceae	g__Catenibacterium	s__Catenibacterium unknown_2
MGYG-HGUT-00826:s__Collinsella unknown_65	0.299408103	0.289997643	0.929025913	d__Bacteria	p__Actinobacteriota	c__Coriobacteriia	o__Coriobacteriales	f__Coriobacteriaceae	g__Collinsella	s__Collinsella unknown_65
MGYG-HGUT-00827:s__Collinsella sp002232035_14	0.953854508	-0.016019308	0.994479474	d__Bacteria	p__Actinobacteriota	c__Coriobacteriia	o__Coriobacteriales	f__Coriobacteriaceae	g__Collinsella	s__Collinsella sp002232035_14
MGYG-HGUT-00828:s__Eubacterium_I unknown_1	0.169513166	0.375841659	0.891825559	d__Bacteria	p__Firmicutes_A	c__Clostridia	o__Lachnospirales	f__Lachnospiraceae	g__Eubacterium_I	s__Eubacterium_I unknown_1
MGYG-HGUT-00830:s__Collinsella sp002232035_15	0.479339455	-0.195684566	0.968532073	d__Bacteria	p__Actinobacteriota	c__Coriobacteriia	o__Coriobacteriales	f__Coriobacteriaceae	g__Collinsella	s__Collinsella sp002232035_15
MGYG-HGUT-00833:s__Collinsella sp002232035_17	0.386955116	0.242048918	0.939709925	d__Bacteria	p__Actinobacteriota	c__Coriobacteriia	o__Coriobacteriales	f__Coriobacteriaceae	g__Collinsella	s__Collinsella sp002232035_17
MGYG-HGUT-00834:s__Oscillibacter unknown_4	0.079371708	0.458439303	0.877826247	d__Bacteria	p__Firmicutes_A	c__Clostridia	o__Oscillospirales	f__Oscillospiraceae	g__Oscillibacter	s__Oscillibacter unknown_4
MGYG-HGUT-00835:s__Collinsella sp002232035_18	0.98546	-0.004	0.99880	d__Bact	p__Actino	c__Coriobacteriia	o__Coriobacteriales	f__Coriobacteriaceae	g__Collinsella	s__Collinsella sp002232035_18

	8522	99878717	3346	eria	bacteriota					
MGYG-HGUT-00837:s__Oscillibacter unknown_5	0.3778934 38	0.2329340 89	0.9397092 5	d__Bacteria	p__Firmicutes_A	c__Clostridia	o__Oscillospirales	f__Oscillospiraceae	g__Oscillibacter	s__Oscillibacter unknown_5
MGYG-HGUT-00838:s__Agathobaculum unknown_1	0.8962555 48	-0.0360971 05	0.9944794 74	d__Bacteria	p__Firmicutes_A	c__Clostridia	o__Oscillospirales	f__Butyricicoccaceae	g__Agathobaculum	s__Agathobaculum unknown_1
MGYG-HGUT-00839:s__Clostridium unknown_2	0.3456909 68	-0.2632341 28	0.9397092 5	d__Bacteria	p__Firmicutes_A	c__Clostridia	o__Clostridiales	f__Clostridiaceae	g__Clostridium	s__Clostridium unknown_2
MGYG-HGUT-00841:s__Oscillibacter unknown_6	0.6684744 22	0.1156300 6	0.9756717 57	d__Bacteria	p__Firmicutes_A	c__Clostridia	o__Oscillospirales	f__Oscillospiraceae	g__Oscillibacter	s__Oscillibacter unknown_6
MGYG-HGUT-00845:s__Collinsella unknown_67	0.9085635 83	0.0315637 58	0.9944794 74	d__Bacteria	p__Actinobacteriota	c__Coriobacteriia	o__Coriobacteriales	f__Coriobacteriaceae	g__Collinsella	s__Collinsella unknown_67
MGYG-HGUT-00847:s__Acidaminococcus unknown_1	0.5030159 23	0.1865617 46	0.9685320 73	d__Bacteria	p__Firmicutes_C	c__Negativicutes	o__Acidaminococcales	f__Acidaminococcaceae	g__Acidaminococcus	s__Acidaminococcus unknown_1
MGYG-HGUT-00855:s__Collinsella unknown_70	0.7454134 14	-0.0898932 97	0.9794584 75	d__Bacteria	p__Actinobacteriota	c__Coriobacteriia	o__Coriobacteriales	f__Coriobacteriaceae	g__Collinsella	s__Collinsella unknown_70
MGYG-HGUT-00857:s__CAG-269 unknown_10	0.8889966 28	-0.0392045 15	0.9944794 74	d__Bacteria	p__Firmicutes_A	c__Clostridia	o__TANB77	f__CAG-508	g__CAG-269	s__CAG-269 unknown_10

MGYG-HGUT-00858:s__Collinsella unknown_72	0.771208497	0.080461119	0.979520484	d__Bacteria	p__Actinobacteriota	c__Coriobacteriia	o__Coriobacteriales	f__Coriobacteriaceae	g__Collinsella	s__Collinsella unknown_72
MGYG-HGUT-00859:s__Tyzzerella unknown_1	0.195214479	-0.358221343	0.891825559	d__Bacteria	p__Firmicutes_A	c__Clostridia	o__Lachnospirales	f__Lachnospiraceae	g__Tyzzerella	s__Tyzzerella unknown_1
MGYG-HGUT-00862:s__Collinsella unknown_73	0.924421174	-0.026337382	0.994479474	d__Bacteria	p__Actinobacteriota	c__Coriobacteriia	o__Coriobacteriales	f__Coriobacteriaceae	g__Collinsella	s__Collinsella unknown_73
MGYG-HGUT-00864:s__CAG-632 unknown_2	0.990366615	-0.003285709	0.998803346	d__Bacteria	p__Firmicutes_A	c__Clostridia	o__Lachnospirales	f__Lachnospiraceae	g__CAG-632	s__CAG-632 unknown_2
MGYG-HGUT-00865:s__Collinsella unknown_74	0.152603774	0.399291862	0.891825559	d__Bacteria	p__Actinobacteriota	c__Coriobacteriia	o__Coriobacteriales	f__Coriobacteriaceae	g__Collinsella	s__Collinsella unknown_74
MGYG-HGUT-00867:s__UBA1409 sp002338885	0.34403608	-0.263005106	0.93970925	d__Bacteria	p__Firmicutes_A	c__Clostridia	o__Oscillospirales	f__Ruminococcaceae	g__UBA1409	s__UBA1409 sp002338885
MGYG-HGUT-00868:s__Collinsella unknown_75	0.794404926	0.06956141	0.982829663	d__Bacteria	p__Actinobacteriota	c__Coriobacteriia	o__Coriobacteriales	f__Coriobacteriaceae	g__Collinsella	s__Collinsella unknown_75
MGYG-HGUT-00870:s__Akkermansia unknown_1	0.700890994	0.10682364	0.979458475	d__Bacteria	p__Verrucomicrobiota	c__Verrucomicrobiae	o__Verrucomicrobiales	f__Akkermansiaceae	g__Akkermansia	s__Akkermansia unknown_1
MGYG-HGUT-00876:s__Faecalibacterium unknown_4	0.14822	0.39584	0.89182	d__Bact	p__Firmicutes_A	c__Clostridia	o__Oscillospirales	f__Ruminococcaceae	g__Faecalibacterium	s__Faecalibacterium unknown_4

	3882	481	5559	eria						
MGYG-HGUT-00877:s__CAG-83 unknown_3	0.0815440 16	0.4621451 45	0.8778262 47	d__Bacteria	p__Firmicutes_A	c__Clostridia	o__Oscillospirales	f__Oscillospiraceae	g__CAG-83	s__CAG-83 unknown_3
MGYG-HGUT-00879:s__Collinsella unknown_77	0.9532675 14	-0.0163966 34	0.9944794 74	d__Bacteria	p__Actinobacteriota	c__Coriobacteriia	o__Coriobacteriales	f__Coriobacteriaceae	g__Collinsella	s__Collinsella unknown_77
MGYG-HGUT-00880:s__Collinsella unknown_78	0.8569843 5	0.0501481 74	0.9928780 88	d__Bacteria	p__Actinobacteriota	c__Coriobacteriia	o__Coriobacteriales	f__Coriobacteriaceae	g__Collinsella	s__Collinsella unknown_78
MGYG-HGUT-00881:s__Collinsella unknown_79	0.5402001 34	-0.1698373 35	0.9685320 73	d__Bacteria	p__Actinobacteriota	c__Coriobacteriia	o__Coriobacteriales	f__Coriobacteriaceae	g__Collinsella	s__Collinsella unknown_79
MGYG-HGUT-00883:s__CAG-170 unknown_3	0.4257179 53	0.2210691 47	0.9483970 24	d__Bacteria	p__Firmicutes_A	c__Clostridia	o__Oscillospirales	f__Oscillospiraceae	g__CAG-170	s__CAG-170 unknown_3
MGYG-HGUT-00884:s__CAG-103 sp900317855_1	0.5000857 08	-0.1861425 87	0.9685320 73	d__Bacteria	p__Firmicutes_A	c__Clostridia	o__Oscillospirales	f__Oscillospiraceae	g__CAG-103	s__CAG-103 sp900317855 _1
MGYG-HGUT-00888:s__ER4 unknown_2	0.4116395 16	0.2198855 9	0.9397092 5	d__Bacteria	p__Firmicutes_A	c__Clostridia	o__Oscillospirales	f__Oscillospiraceae	g__ER4	s__ER4 unknown_2
MGYG-HGUT-00892:s__CAG-115 sp002492075	0.4154142 19	0.2231384 16	0.9397092 5	d__Bacteria	p__Firmicutes_A	c__Clostridia	o__Oscillospirales	f__Ruminococcaceae	g__CAG-115	s__CAG-115 sp002492075
MGYG-HGUT-00901:s__Acidami	0.40527	0.22502	0.93970	d__Bact	p__Firmicutes_C	c__Negativicutes	o__Acidamin	f__Acidaminoc	g__Acidaminococcus	s__Acidaminococcus fermentans

nococcus fermentans	5889	9945	925	eria			ococcales	occaceae		
MGYG-HGUT-00904:s__Collinsella unknown_83	0.459710144	0.205269238	0.965574692	d__Bacteria	p__Actinobacteriota	c__Coriobacteriia	o__Coriobacteriales	f__Coriobacteriaceae	g__Collinsella	s__Collinsella unknown_83
MGYG-HGUT-00909:s__UBA9502 unknown_3	0.995238313	0.001638246	0.998803346	d__Bacteria	p__Firmicutes_A	c__Clostridia	o__Lachnospirales	f__Lachnospiraceae	g__UBA9502	s__UBA9502 unknown_3
MGYG-HGUT-00911:s__Slackia_A unknown_1	0.787556908	-0.074937814	0.982796225	d__Bacteria	p__Actinobacteriota	c__Coriobacteriia	o__Coriobacteriales	f__Eggerthellaceae	g__Slackia_A	s__Slackia_A unknown_1
MGYG-HGUT-00912:s__Collinsella sp002232035_19	0.832233764	0.05757436	0.99222019	d__Bacteria	p__Actinobacteriota	c__Coriobacteriia	o__Coriobacteriales	f__Coriobacteriaceae	g__Collinsella	s__Collinsella sp002232035_19
MGYG-HGUT-00913:s__Blautia unknown_1	0.767183249	0.082747817	0.979520484	d__Bacteria	p__Firmicutes_A	c__Clostridia	o__Lachnospirales	f__Lachnospiraceae	g__Blautia	s__Blautia unknown_1
MGYG-HGUT-00917:s__Phascolarctobacterium unknown_1	0.397910522	0.235897253	0.93970925	d__Bacteria	p__Firmicutes_C	c__Negativicutes	o__Acidaminococcales	f__Acidaminococcaceae	g__Phascolarctobacterium	s__Phascolarctobacterium unknown_1
MGYG-HGUT-00920:s__Gemmiger unknown_3	0.808945445	0.067862865	0.987142302	d__Bacteria	p__Firmicutes_A	c__Clostridia	o__Oscillospirales	f__Ruminococcaceae	g__Gemmiger	s__Gemmiger unknown_3
MGYG-HGUT-00923:s__Bacteroides_C unknown	0.449883968	-0.211936521	0.961292208	d__Bacteria	p__Bacteroidota	c__Bacteroidia	o__Bacteroidales	f__Bacteroidaceae	g__Bacteroides_C	s__Bacteroides_C unknown
MGYG-HGUT-00926:s__Veillonella unknown_5	0.63961	-0.13095	0.97567	d__Bact	p__Firmicutes_C	c__Negativicutes	o__Veillonellales	f__Veillonellaceae	g__Veillonella	s__Veillonella unknown_5

	3756	0005	1757	eria						
MGYG-HGUT-00929:s__CAG-273 sp000437855	0.491226621	-0.188904382	0.968532073	d__Bacteria	p__Firmicutes_A	c__Clostridia	o__TANB77	f__CAG-508	g__CAG-273	s__CAG-273 sp000437855
MGYG-HGUT-00932:s__Acutalibacteraceae unknown unknown_8	0.488393185	0.193522652	0.968532073	d__Bacteria	p__Firmicutes_A	c__Clostridia	o__Oscillospirales	f__Acutalibacteraceae	g__Acutalibacteraceae unknown	s__Acutalibacteraceae unknown unknown_8
MGYG-HGUT-00934:s__Collinsella unknown_88	0.501187507	0.184010762	0.968532073	d__Bacteria	p__Actinobacteriota	c__Coriobacteriia	o__Coriobacteriales	f__Coriobacteriaceae	g__Collinsella	s__Collinsella unknown_88
MGYG-HGUT-00935:s__Acutalibacteraceae unknown unknown_9	0.582113348	-0.154595173	0.975671757	d__Bacteria	p__Firmicutes_A	c__Clostridia	o__Oscillospirales	f__Acutalibacteraceae	g__Acutalibacteraceae unknown	s__Acutalibacteraceae unknown unknown_9
MGYG-HGUT-00936:s__Collinsella unknown_89	0.089847415	0.464500093	0.883307256	d__Bacteria	p__Actinobacteriota	c__Coriobacteriia	o__Coriobacteriales	f__Coriobacteriaceae	g__Collinsella	s__Collinsella unknown_89
MGYG-HGUT-00937:s__Acutalibacteraceae unknown unknown_10	0.105297466	0.450755792	0.883307256	d__Bacteria	p__Firmicutes_A	c__Clostridia	o__Oscillospirales	f__Acutalibacteraceae	g__Acutalibacteraceae unknown	s__Acutalibacteraceae unknown unknown_10
MGYG-HGUT-00938:s__UBA5446 unknown_2	0.420955239	0.217223872	0.942006355	d__Bacteria	p__Firmicutes_A	c__Clostridia	o__Oscillospirales	f__Oscillospiraceae	g__UBA5446	s__UBA5446 unknown_2
MGYG-HGUT-00940:s__Collinsella unknown_90	0.079330366	0.486975576	0.877826247	d__Bacteria	p__Actinobacteriota	c__Coriobacteriia	o__Coriobacteriales	f__Coriobacteriaceae	g__Collinsella	s__Collinsella unknown_90
MGYG-HGUT-00942:s__CAG-314 unknown unknown_1	0.50654	0.18585	0.96853	d__Bact	p__Firmicutes_A	c__Clostridia	o__4C28d-15	f__CAG-314	g__CAG-314 unknown	s__CAG-314 unknown unknown_1

	9234	3955	2073	eria						
MGYG-HGUT-00947:s__Collinsella unknown_91	0.132371416	0.410661019	0.891825559	d__Bacteria	p__Actinobacteriota	c__Coriobacteriia	o__Coriobacteriales	f__Coriobacteriaceae	g__Collinsella	s__Collinsella unknown_91
MGYG-HGUT-00949:s__Collinsella unknown_92	0.098283597	0.447340791	0.883307256	d__Bacteria	p__Actinobacteriota	c__Coriobacteriia	o__Coriobacteriales	f__Coriobacteriaceae	g__Collinsella	s__Collinsella unknown_92
MGYG-HGUT-00954:s__Blautia_A unknown_8	0.033341552	0.534421733	0.768225834	d__Bacteria	p__Firmicutes_A	c__Clostridia	o__Lachnospirales	f__Lachnospiraceae	g__Blautia_A	s__Blautia_A unknown_8
MGYG-HGUT-00957:s__Prevotella sp000436695	0.662212938	-0.121562264	0.975671757	d__Bacteria	p__Bacteroidota	c__Bacteroidia	o__Bacteroidales	f__Bacteroidaceae	g__Prevotella	s__Prevotella sp000436695
MGYG-HGUT-00959:s__Ruminococcus unknown_1	0.391798017	0.238814198	0.93970925	d__Bacteria	p__Firmicutes_A	c__Clostridia	o__Oscillospirales	f__Ruminococcaceae	g__Ruminococcus	s__Ruminococcus unknown_1
MGYG-HGUT-00961:s__Megamonas unknown_2	0.63634374	0.131101093	0.975671757	d__Bacteria	p__Firmicutes_C	c__Negativicutes	o__Selenomonadales	f__Selenomonadaceae	g__Megamonas	s__Megamonas unknown_2
MGYG-HGUT-00963:s__CAG-617 unknown_1	0.545378483	0.168499734	0.968532073	d__Bacteria	p__Bacteroidota	c__Bacteroidia	o__Bacteroidales	f__Bacteroidaceae	g__CAG-617	s__CAG-617 unknown_1
MGYG-HGUT-00966:s__Blautia_A unknown_9	0.890116706	0.038519808	0.994479474	d__Bacteria	p__Firmicutes_A	c__Clostridia	o__Lachnospirales	f__Lachnospiraceae	g__Blautia_A	s__Blautia_A unknown_9
MGYG-HGUT-00969:s__Eubacterium_I unknown_2	0.559509984	0.158437183	0.968841416	d__Bacteria	p__Firmicutes_A	c__Clostridia	o__Lachnospirales	f__Lachnospiraceae	g__Eubacterium_I	s__Eubacterium_I unknown_2

MGYG-HGUT-00979:s__Collinsella unknown_94	0.358150587	0.246808615	0.93970925	d__Bacteria	p__Actinobacteriota	c__Coriobacteriia	o__Coriobacteriales	f__Coriobacteriaceae	g__Collinsella	s__Collinsella unknown_94
MGYG-HGUT-00981:s__Collinsella unknown_95	0.522164007	0.177514841	0.968532073	d__Bacteria	p__Actinobacteriota	c__Coriobacteriia	o__Coriobacteriales	f__Coriobacteriaceae	g__Collinsella	s__Collinsella unknown_95
MGYG-HGUT-00982:s__CAG-354 unknown_2	0.902696438	0.03412505	0.994479474	d__Bacteria	p__Firmicutes_A	c__Clostridia	o__TANB77	f__CAG-508	g__CAG-354	s__CAG-354 unknown_2
MGYG-HGUT-00983:s__Odoribacter unknown_3	0.094128278	0.464362169	0.883307256	d__Bacteria	p__Bacteroidota	c__Bacteroidia	o__Bacteroidales	f__Marinifilaceae	g__Odoribacter	s__Odoribacter unknown_3
MGYG-HGUT-00984:s__Collinsella unknown_96	0.923498344	-0.026990184	0.994479474	d__Bacteria	p__Actinobacteriota	c__Coriobacteriia	o__Coriobacteriales	f__Coriobacteriaceae	g__Collinsella	s__Collinsella unknown_96
MGYG-HGUT-00986:s__Collinsella aerofaciens_F_3	0.072785273	0.489046397	0.877826247	d__Bacteria	p__Actinobacteriota	c__Coriobacteriia	o__Coriobacteriales	f__Coriobacteriaceae	g__Collinsella	s__Collinsella aerofaciens_F_3
MGYG-HGUT-00987:s__Dorea unknown_1	0.015548319	0.666099192	0.768225834	d__Bacteria	p__Firmicutes_A	c__Clostridia	o__Lachnospirales	f__Lachnospiraceae	g__Dorea	s__Dorea unknown_1
MGYG-HGUT-00988:s__Anaerostipes unknown_2	0.594161465	-0.142803321	0.975671757	d__Bacteria	p__Firmicutes_A	c__Clostridia	o__Lachnospirales	f__Lachnospiraceae	g__Anaerostipes	s__Anaerostipes unknown_2
MGYG-HGUT-00990:s__Blautia unknown_2	0.928884825	-0.024688659	0.994479474	d__Bacteria	p__Firmicutes_A	c__Clostridia	o__Lachnospirales	f__Lachnospiraceae	g__Blautia	s__Blautia unknown_2

MGYG-HGUT-00996:s__Collinsella unknown_99	0.920415775	-0.027387486	0.994479474	d__Bacteria	p__Actinobacteriota	c__Coriobacteriia	o__Coriobacteriales	f__Coriobacteriaceae	g__Collinsella	s__Collinsella unknown_99
MGYG-HGUT-00997:s__Roseburia unknown_3	0.942367267	0.020083047	0.994479474	d__Bacteria	p__Firmicutes_A	c__Clostridia	o__Lachnospirales	f__Lachnospiraceae	g__Roseburia	s__Roseburia unknown_3
MGYG-HGUT-01002:s__Collinsella aerofaciens_F_4	0.003351719	0.780918562	0.648124372	d__Bacteria	p__Actinobacteriota	c__Coriobacteriia	o__Coriobacteriales	f__Coriobacteriaceae	g__Collinsella	s__Collinsella aerofaciens_F_4
MGYG-HGUT-01003:s__Collinsella unknown_100	0.298703945	0.283674459	0.929025913	d__Bacteria	p__Actinobacteriota	c__Coriobacteriia	o__Coriobacteriales	f__Coriobacteriaceae	g__Collinsella	s__Collinsella unknown_100
MGYG-HGUT-01004:s__CAG-56 unknown_1	0.251120571	0.317249393	0.902181367	d__Bacteria	p__Firmicutes_A	c__Clostridia	o__Lachnospirales	f__Lachnospiraceae	g__CAG-56	s__CAG-56 unknown_1
MGYG-HGUT-01005:s__Tyzzerella unknown_2	0.877623486	-0.042763446	0.994479474	d__Bacteria	p__Firmicutes_A	c__Clostridia	o__Lachnospirales	f__Lachnospiraceae	g__Tyzzerella	s__Tyzzerella unknown_2
MGYG-HGUT-01006:s__Pauljensenia sp000278725	0.722821479	0.098142506	0.979458475	d__Bacteria	p__Actinobacteriota	c__Actinobacteria	o__Actinomycetales	f__Actinomycetaceae	g__Pauljensenia	s__Pauljensenia sp000278725
MGYG-HGUT-01007:s__Intestinibacter unknown	0.518378645	-0.177173676	0.968532073	d__Bacteria	p__Firmicutes_A	c__Clostridia	o__Peptostreptococcales	f__Peptostreptococcaceae	g__Intestinibacter	s__Intestinibacter unknown
MGYG-HGUT-01008:s__Absiella unknown_3	0.608723677	0.139532904	0.975671757	d__Bacteria	p__Firmicutes	c__Bacilli	o__Erysipelotrichales	f__Erysipelotrichaceae	g__Absiella	s__Absiella unknown_3

MGYG-HGUT-01015:s__Gemmiger unknown_4	0.727120488	0.095616698	0.979458475	d__Bacteria	p__Firmicutes_A	c__Clostridia	o__Oscillospirales	f__Ruminococcaceae	g__Gemmiger	s__Gemmiger unknown_4
MGYG-HGUT-01019:s__Collinsella unknown_102	0.414419527	0.228198604	0.93970925	d__Bacteria	p__Actinobacteriota	c__Coriobacteriia	o__Coriobacteriales	f__Coriobacteriaceae	g__Collinsella	s__Collinsella unknown_102
MGYG-HGUT-01021:s__Ruminococcus_C sp000980705	0.906463288	0.032807695	0.994479474	d__Bacteria	p__Firmicutes_A	c__Clostridia	o__Oscillospirales	f__Ruminococcaceae	g__Ruminococcus_C	s__Ruminococcus_C sp000980705
MGYG-HGUT-01024:s__Monoglobales unknown unknown unknown_4	0.003078002	0.787580019	0.648124372	d__Bacteria	p__Firmicutes_A	c__Clostridia	o__Monoglobales	f__Monoglobales unknown	g__Monoglobales unknown unknown	s__Monoglobales unknown unknown unknown_4
MGYG-HGUT-01027:s__CAG-56 unknown_2	0.033378098	0.589955554	0.768225834	d__Bacteria	p__Firmicutes_A	c__Clostridia	o__Lachnospirales	f__Lachnospiraceae	g__CAG-56	s__CAG-56 unknown_2
MGYG-HGUT-01029:s__CAG-217 sp000436335	0.273817035	0.302889681	0.929025913	d__Bacteria	p__Firmicutes_A	c__Clostridia	o__Oscillospirales	f__Acutalibacteraceae	g__CAG-217	s__CAG-217 sp000436335
MGYG-HGUT-01032:s__Eubacterium_G unknown_1	0.451866511	0.208113378	0.961292208	d__Bacteria	p__Firmicutes_A	c__Clostridia	o__Lachnospirales	f__Lachnospiraceae	g__Eubacterium_G	s__Eubacterium_G unknown_1
MGYG-HGUT-01036:s__CAG-1031 unknown	0.533645888	-0.173897187	0.968532073	d__Bacteria	p__Bacteroidota	c__Bacteroidia	o__Bacteroidales	f__Muribaculaceae	g__CAG-1031	s__CAG-1031 unknown
MGYG-HGUT-01038:s__Prevotellamassilia unknown_4	0.052400538	0.538192261	0.857211132	d__Bacteria	p__Bacteroidota	c__Bacteroidia	o__Bacteroidales	f__Bacteroidaceae	g__Prevotellamassilia	s__Prevotellamassilia unknown_4

MGYG-HGUT-01039:s__Bacteroides_A unknown_3	0.907244717	-0.032506615	0.994479474	d__Bacteria	p__Bacteroidota	c__Bacteroidia	o__Bacteroidales	f__Bacteroidaceae	g__Bacteroides_A	s__Bacteroides_A unknown_3
MGYG-HGUT-01040:s__Prevotella unknown_13	0.202727299	0.346676002	0.891825559	d__Bacteria	p__Bacteroidota	c__Bacteroidia	o__Bacteroidales	f__Bacteroidaceae	g__Prevotella	s__Prevotella unknown_13
MGYG-HGUT-01042:s__Prevotella sp000436595	0.321170804	-0.276621085	0.929425266	d__Bacteria	p__Bacteroidota	c__Bacteroidia	o__Bacteroidales	f__Bacteroidaceae	g__Prevotella	s__Prevotella sp000436595
MGYG-HGUT-01045:s__Clostridium_M unknown_4	0.176550041	-0.358125113	0.891825559	d__Bacteria	p__Firmicutes_A	c__Clostridia	o__Lachnospirales	f__Lachnospiraceae	g__Clostridium_M	s__Clostridium_M unknown_4
MGYG-HGUT-01046:s__CAG-103 unknown_1	0.703974925	0.100913531	0.979458475	d__Bacteria	p__Firmicutes_A	c__Clostridia	o__Oscillospirales	f__Oscillospiraceae	g__CAG-103	s__CAG-103 unknown_1
MGYG-HGUT-01051:s__Prevotella unknown_14	0.456322271	-0.204442331	0.965574692	d__Bacteria	p__Bacteroidota	c__Bacteroidia	o__Bacteroidales	f__Bacteroidaceae	g__Prevotella	s__Prevotella unknown_14
MGYG-HGUT-01052:s__Blautia unknown_3	0.600776317	0.146197989	0.975671757	d__Bacteria	p__Firmicutes_A	c__Clostridia	o__Lachnospirales	f__Lachnospiraceae	g__Blautia	s__Blautia unknown_3
MGYG-HGUT-01056:s__Prevotella unknown_15	0.604954259	-0.142679736	0.975671757	d__Bacteria	p__Bacteroidota	c__Bacteroidia	o__Bacteroidales	f__Bacteroidaceae	g__Prevotella	s__Prevotella unknown_15
MGYG-HGUT-01057:s__Negativi	0.77842	0.07699	0.97952	d__Bact	p__Firmicutes_A	c__Clostridia	o__Oscillospirales	f__Ruminococcaceae	g__Negativibacillus	s__Negativibacillus sp000435195

bacillus sp000435195	9834	6975	0484	eria						
MGYG-HGUT-01060:s__Dialister sp900343095	0.2180188852	-0.336305709	0.891825559	d__Bacteria	p__Firmicutes_C	c__Negativicutes	o__Veillonellales	f__Dialisteraceae	g__Dialister	s__Dialister sp900343095
MGYG-HGUT-01061:s__Collinsella unknown_105	0.186597623	0.367092818	0.891825559	d__Bacteria	p__Actinobacteriota	c__Coriobacteriia	o__Coriobacteriales	f__Coriobacteriaceae	g__Collinsella	s__Collinsella unknown_105
MGYG-HGUT-01064:s__Streptococcus unknown_6	0.247085641	0.319166195	0.902181367	d__Bacteria	p__Firmicutes	c__Bacilli	o__Lactobacillales	f__Streptococcaceae	g__Streptococcus	s__Streptococcus unknown_6
MGYG-HGUT-01065:s__Clostridium_M unknown_5	0.96999313	-0.0105626	0.994479474	d__Bacteria	p__Firmicutes_A	c__Clostridia	o__Lachnospirales	f__Lachnospiraceae	g__Clostridium_M	s__Clostridium_M unknown_5
MGYG-HGUT-01066:s__F0422 sp001553345	0.884358803	0.040846918	0.994479474	d__Bacteria	p__Firmicutes_C	c__Negativicutes	o__Veillonellales	f__Veillonellaceae	g__F0422	s__F0422 sp001553345
MGYG-HGUT-01068:s__Streptococcus sp000187445	0.550695542	0.16505739	0.968532073	d__Bacteria	p__Firmicutes	c__Bacilli	o__Lactobacillales	f__Streptococcaceae	g__Streptococcus	s__Streptococcus sp000187445
MGYG-HGUT-01069:s__UBA4871 unknown_1	0.927979701	-0.024915746	0.994479474	d__Bacteria	p__Firmicutes_A	c__Clostridia	o__Oscillospirales	f__Acutalibacteraceae	g__UBA4871	s__UBA4871 unknown_1
MGYG-HGUT-01072:s__Dorea unknown_2	0.49099267	-0.192225635	0.968532073	d__Bacteria	p__Firmicutes_A	c__Clostridia	o__Lachnospirales	f__Lachnospiraceae	g__Dorea	s__Dorea unknown_2
MGYG-HGUT-01074:s__Prevotella sp002251365	0.91065	-0.031	0.99447	d__Bact	p__Bacter	c__Bacteroidia	o__Bacteroidales	f__Bacteroidaceae	g__Prevotella	s__Prevotella sp002251365

	7815	543401	9474	eria	oidota					
MGYG-HGUT-01076:s__CAG-411 sp000437275	0.099665741	0.448618222	0.883307256	d__Bacteria	p__Firmicutes_A	c__Clostridia	o__Lachnospirales	f__Lachnospiraceae	g__CAG-411	s__CAG-411 sp000437275
MGYG-HGUT-01081:s__Acutalibacter unknown_1	0.321424071	-0.252005642	0.929425266	d__Bacteria	p__Firmicutes_A	c__Clostridia	o__Oscillospirales	f__Acutalibacteraceae	g__Acutalibacter	s__Acutalibacter unknown_1
MGYG-HGUT-01082:s__Ruminococcus_E unknown_1	0.174453307	0.361237123	0.891825559	d__Bacteria	p__Firmicutes_A	c__Clostridia	o__Oscillospirales	f__Acutalibacteraceae	g__Ruminococcus_E	s__Ruminococcus_E unknown_1
MGYG-HGUT-01084:s__Collinsella unknown_106	0.572529615	0.155712074	0.975319755	d__Bacteria	p__Actinobacteriota	c__Coriobacteriia	o__Coriobacteriales	f__Coriobacteriaceae	g__Collinsella	s__Collinsella unknown_106
MGYG-HGUT-01085:s__RC9 sp000434935	0.955442823	0.014998802	0.994479474	d__Bacteria	p__Bacteroidota	c__Bacteroidia	o__Bacteroidales	f__UBA932	g__RC9	s__RC9 sp000434935
MGYG-HGUT-01086:s__Collinsella unknown_107	0.156577989	0.388971118	0.891825559	d__Bacteria	p__Actinobacteriota	c__Coriobacteriia	o__Coriobacteriales	f__Coriobacteriaceae	g__Collinsella	s__Collinsella unknown_107
MGYG-HGUT-01087:s__Anaerostipes unknown_3	0.256462019	-0.317560441	0.905666614	d__Bacteria	p__Firmicutes_A	c__Clostridia	o__Lachnospirales	f__Lachnospiraceae	g__Anaerostipes	s__Anaerostipes unknown_3
MGYG-HGUT-01089:s__Clostridium sp000435835	0.108211379	-0.439568818	0.883307256	d__Bacteria	p__Firmicutes_A	c__Clostridia	o__Clostridiales	f__Clostridiaceae	g__Clostridium	s__Clostridium sp000435835

MGYG-HGUT-01092:s__Collinsella unknown_108	0.1124893 89	0.4212877 14	0.8833072 56	d__Bacteria	p__Actinobacteriota	c__Coriobacteriia	o__Coriobacteriales	f__Coriobacteriaceae	g__Collinsella	s__Collinsella unknown_108
MGYG-HGUT-01097:s__Prevotella sp000834015	0.2626389 48	-0.3115111 07	0.9131314 21	d__Bacteria	p__Bacteroidota	c__Bacteroidia	o__Bacteroidales	f__Bacteroidaceae	g__Prevotella	s__Prevotella sp000834015
MGYG-HGUT-01098:s__Eubacterium_R sp002493325	0.1185432 56	0.4287499 2	0.8833072 56	d__Bacteria	p__Firmicutes_A	c__Clostridia	o__Oscillospirales	f__Acutalibacteraceae	g__Eubacterium_R	s__Eubacterium_R sp002493325
MGYG-HGUT-01099:s__Collinsella unknown_109	0.2865495 15	0.2953779 97	0.9290259 13	d__Bacteria	p__Actinobacteriota	c__Coriobacteriia	o__Coriobacteriales	f__Coriobacteriaceae	g__Collinsella	s__Collinsella unknown_109
MGYG-HGUT-01100:s__Weissella viridescens	0.9451010 32	-0.0192510 26	0.9944794 74	d__Bacteria	p__Firmicutes	c__Bacilli	o__Lactobacillales	f__Lactobacillaceae	g__Weissella	s__Weissella viridescens
MGYG-HGUT-01103:s__CAG-74 unknown unknown_3	0.3023892 84	0.2710514 59	0.9290259 13	d__Bacteria	p__Firmicutes_A	c__Clostridia	o__Christensenellales	f__CAG-74	g__CAG-74 unknown	s__CAG-74 unknown unknown_3
MGYG-HGUT-01104:s__Oscillospiraceae unknown unknown_3	0.9882399 98	0.0040709 62	0.9988033 46	d__Bacteria	p__Firmicutes_A	c__Clostridia	o__Oscillospirales	f__Oscillospiraceae	g__Oscillospiraceae unknown	s__Oscillospiraceae unknown unknown_3
MGYG-HGUT-01106:s__Pygmaiobacter unknown	0.9965676 36	0.0012023 96	0.9988033 46	d__Bacteria	p__Firmicutes_A	c__Clostridia	o__Oscillospirales	f__Ruminococcaceae	g__Pygmaiobacter	s__Pygmaiobacter unknown
MGYG-HGUT-01108:s__Collinsella unknown_112	0.2473793 19	0.3163694 24	0.9021813 67	d__Bacteria	p__Actinobacteriota	c__Coriobacteriia	o__Coriobacteriales	f__Coriobacteriaceae	g__Collinsella	s__Collinsella unknown_112

MGYG-HGUT-01109:s__CAG-110 unknown_6	0.017802664	0.650825478	0.768225834	d__Bacteria	p__Firmicutes_A	c__Clostridia	o__Oscillospirales	f__Oscillospiraceae	g__CAG-110	s__CAG-110 unknown_6
MGYG-HGUT-01110:s__CAG-269 sp000431335	0.148052984	-0.396444052	0.891825559	d__Bacteria	p__Firmicutes_A	c__Clostridia	o__TANB77	f__CAG-508	g__CAG-269	s__CAG-269 sp000431335
MGYG-HGUT-01111:s__Roseburia unknown_4	0.212187969	0.333161034	0.891825559	d__Bacteria	p__Firmicutes_A	c__Clostridia	o__Lachnospirales	f__Lachnospiraceae	g__Roseburia	s__Roseburia unknown_4
MGYG-HGUT-01114:s__Collinsella unknown_113	0.685173226	0.112585615	0.975768579	d__Bacteria	p__Actinobacteriota	c__Coriobacteriia	o__Coriobacteriales	f__Coriobacteriaceae	g__Collinsella	s__Collinsella unknown_113
MGYG-HGUT-01115:s__Agathobaculum unknown_2	0.145921912	0.397937109	0.891825559	d__Bacteria	p__Firmicutes_A	c__Clostridia	o__Oscillospirales	f__Butyricicoccaceae	g__Agathobaculum	s__Agathobaculum unknown_2
MGYG-HGUT-01118:s__Anaerotignaceae unknown unknown	0.850421159	0.052633676	0.992878088	d__Bacteria	p__Firmicutes_A	c__Clostridia	o__Lachnospirales	f__Anaerotignaceae	g__Anaerotignaceae unknown	s__Anaerotignaceae unknown unknown
MGYG-HGUT-01120:s__Butyricicoccus_A sp002395695	0.324713597	0.268797638	0.930624692	d__Bacteria	p__Firmicutes_A	c__Clostridia	o__Oscillospirales	f__Butyricicoccaceae	g__Butyricicoccus_A	s__Butyricicoccus_A sp002395695
MGYG-HGUT-01126:s__Collinsella unknown_118	0.117074786	0.434109758	0.883307256	d__Bacteria	p__Actinobacteriota	c__Coriobacteriia	o__Coriobacteriales	f__Coriobacteriaceae	g__Collinsella	s__Collinsella unknown_118
MGYG-HGUT-01129:s__Ruminococcus_C sp000437255	0.862907472	-0.048293864	0.994479474	d__Bacteria	p__Firmicutes_A	c__Clostridia	o__Oscillospirales	f__Ruminococcaceae	g__Ruminococcus_C	s__Ruminococcus_C sp000437255

MGYG-HGUT-01131:s__Prevotella unknown_16	0.66018923	0.12142394	0.97567175	d__Bacteria	p__Bacteroidota	c__Bacteroidia	o__Bacteroidales	f__Bacteroidaceae	g__Prevotella	s__Prevotella unknown_16
MGYG-HGUT-01132:s__UBA1685 sp002320595	0.64189611 9	0.12927866 1	0.97567175 7	d__Bacteria	p__Firmicutes_A	c__Clostridia	o__Christensenellales	f__CAG-138	g__UBA1685	s__UBA1685 sp002320595
MGYG-HGUT-01133:s__CAG-272 unknown unknown_8	0.97920 29	0.00732880 1	0.99764791 4	d__Bacteria	p__Firmicutes_A	c__Clostridia	o__Oscillospirales	f__CAG-272	g__CAG-272 unknown	s__CAG-272 unknown unknown_8
MGYG-HGUT-01135:s__Collinsella unknown_122	0.37869755	0.24289987 3	0.93970925	d__Bacteria	p__Actinobacteriota	c__Coriobacteriia	o__Coriobacteriales	f__Coriobacteriaceae	g__Collinsella	s__Collinsella unknown_122
MGYG-HGUT-01136:s__CAG-103 unknown_2	0.85598781 8	-0.05048785 5	0.99287808 8	d__Bacteria	p__Firmicutes_A	c__Clostridia	o__Oscillospirales	f__Oscillospiraceae	g__CAG-103	s__CAG-103 unknown_2
MGYG-HGUT-01138:s__Collinsella sp002232035_20	0.07754697 9	0.48533408 9	0.87782624 7	d__Bacteria	p__Actinobacteriota	c__Coriobacteriia	o__Coriobacteriales	f__Coriobacteriaceae	g__Collinsella	s__Collinsella sp002232035_20
MGYG-HGUT-01139:s__Collinsella unknown_124	0.28590972 9	0.29342670 1	0.92902591 3	d__Bacteria	p__Actinobacteriota	c__Coriobacteriia	o__Coriobacteriales	f__Coriobacteriaceae	g__Collinsella	s__Collinsella unknown_124
MGYG-HGUT-01140:s__Collinsella unknown_125	0.13062526 6	0.41548243 9	0.89182555 9	d__Bacteria	p__Actinobacteriota	c__Coriobacteriia	o__Coriobacteriales	f__Coriobacteriaceae	g__Collinsella	s__Collinsella unknown_125
MGYG-HGUT-01141:s__CAG-303 sp000437755	0.26315762 8	0.30563729	0.91313142 1	d__Bacteria	p__Firmicutes_A	c__Clostridia	o__Lachnospirales	f__Lachnospiraceae	g__CAG-303	s__CAG-303 sp000437755

MGYG-HGUT-01142:s__Collinsella unknown_126	0.889018169	0.039065748	0.994479474	d__Bacteria	p__Actinobacteriota	c__Coriobacteriia	o__Coriobacteriales	f__Coriobacteriaceae	g__Collinsella	s__Collinsella unknown_126
MGYG-HGUT-01150:s__Collinsella unknown_127	0.064460963	0.509253808	0.85721132	d__Bacteria	p__Actinobacteriota	c__Coriobacteriia	o__Coriobacteriales	f__Coriobacteriaceae	g__Collinsella	s__Collinsella unknown_127
MGYG-HGUT-01151:s__Ruminococcus_C sp000433635	0.855631513	-0.048927757	0.992878088	d__Bacteria	p__Firmicutes_A	c__Clostridia	o__Oscillospirales	f__Ruminococcaceae	g__Ruminococcus_C	s__Ruminococcus_C sp000433635
MGYG-HGUT-01152:s__Collinsella sp002232035_21	0.389949643	0.236589155	0.93970925	d__Bacteria	p__Actinobacteriota	c__Coriobacteriia	o__Coriobacteriales	f__Coriobacteriaceae	g__Collinsella	s__Collinsella sp002232035_21
MGYG-HGUT-01153:s__Eggerthellaceae unknown unknown_6	0.858866864	-0.048664442	0.992878088	d__Bacteria	p__Actinobacteriota	c__Coriobacteriia	o__Coriobacteriales	f__Eggerthellaceae	g__Eggerthellaceae unknown	s__Eggerthellaceae unknown unknown_6
MGYG-HGUT-01155:s__UBA738 unknown_3	0.162503541	0.376543413	0.891825559	d__Bacteria	p__Firmicutes_A	c__Clostridia	o__Oscillospirales	f__Oscillospiraceae	g__UBA738	s__UBA738 unknown_3
MGYG-HGUT-01157:s__Gemmiger unknown_5	0.240302196	0.319301882	0.902181367	d__Bacteria	p__Firmicutes_A	c__Clostridia	o__Oscillospirales	f__Ruminococcaceae	g__Gemmiger	s__Gemmiger unknown_5
MGYG-HGUT-01158:s__Collinsella unknown_128	0.678647602	-0.116140841	0.975671757	d__Bacteria	p__Actinobacteriota	c__Coriobacteriia	o__Coriobacteriales	f__Coriobacteriaceae	g__Collinsella	s__Collinsella unknown_128
MGYG-HGUT-01159:s__Bifidobacterium pullorum	0.722051272	-0.09866	0.979458475	d__Bacteria	p__Actinobacteriota	c__Actinobacteria	o__Actinomycetales	f__Bifidobacteriaceae	g__Bifidobacterium	s__Bifidobacterium pullorum

		0332								
MGYG-HGUT-01160:s__CAG-274 sp000432155	0.3559197520	-0.254674077	0.93970925	d__Bacteria	p__Firmicutes_A	c__Clostridia	o__Lachnospirales	f__CAG-274	g__CAG-274	s__CAG-274 sp000432155
MGYG-HGUT-01161:s__Collinsella unknown_129	0.607131879	-0.143967382	0.975671757	d__Bacteria	p__Actinobacteriota	c__Coriobacteriia	o__Coriobacteriales	f__Coriobacteriaceae	g__Collinsella	s__Collinsella unknown_129
MGYG-HGUT-01164:s__Prevotella lascolaii	0.774249611	-0.077749586	0.979520484	d__Bacteria	p__Bacteroidota	c__Bacteroidia	o__Bacteroidales	f__Bacteroidaceae	g__Prevotella	s__Prevotella lascolaii
MGYG-HGUT-01165:s__Collinsella sp000763055_1	0.450986357	0.203286509	0.961292208	d__Bacteria	p__Actinobacteriota	c__Coriobacteriia	o__Coriobacteriales	f__Coriobacteriaceae	g__Collinsella	s__Collinsella sp000763055_1
MGYG-HGUT-01166:s__F23-B02 unknown_3	0.879068846	-0.040948006	0.994479474	d__Bacteria	p__Firmicutes_A	c__Clostridia	o__Oscillospirales	f__Oscillospiraceae	g__F23-B02	s__F23-B02 unknown_3
MGYG-HGUT-01168:s__CAG-1427 sp000436075	0.81339995	0.066172068	0.987142302	d__Bacteria	p__Actinobacteriota	c__Coriobacteriia	o__Coriobacteriales	f__Eggerthellaceae	g__CAG-1427	s__CAG-1427 sp000436075
MGYG-HGUT-01169:s__Acutalibacter unknown_2	0.790930879	0.070857896	0.982969663	d__Bacteria	p__Firmicutes_A	c__Clostridia	o__Oscillospirales	f__Acutalibacteraceae	g__Acutalibacter	s__Acutalibacter unknown_2
MGYG-HGUT-01171:s__Peptococcaceae unknown unknown_3	0.184527282	-0.367697142	0.891825559	d__Bacteria	p__Firmicutes_B	c__Peptococcia	o__Peptococcales	f__Peptococcaceae	g__Peptococcaceae unknown	s__Peptococcaceae unknown unknown_3

MGYG-HGUT-01173:s__Collinsella unknown_130	0.250399048	-0.309399576	0.902181367	d__Bacteria	p__Actinobacteriota	c__Coriobacteriia	o__Coriobacteriales	f__Coriobacteriaceae	g__Collinsella	s__Collinsella unknown_130
MGYG-HGUT-01177:s__OEMR01 sp900199515	0.971272035	0.010094077	0.994649356	d__Bacteria	p__Firmicutes	c__Bacilli	o__Erysipelotrichales	f__Erysipelotrichaceae	g__OEMR01	s__OEMR01 sp900199515
MGYG-HGUT-01178:s__GCA-900066575 unknown_1	0.612931985	0.140437515	0.975671757	d__Bacteria	p__Firmicutes_A	c__Clostridia	o__Lachnospirales	f__Lachnospiraceae	g__GCA-900066575	s__GCA-900066575 unknown_1
MGYG-HGUT-01182:s__CAG-269 sp000435535	0.032327176	-0.586029017	0.768225834	d__Bacteria	p__Firmicutes_A	c__Clostridia	o__TANB77	f__CAG-508	g__CAG-269	s__CAG-269 sp000435535
MGYG-HGUT-01186:s__Agathobacter unknown_3	0.98029651	-0.006660354	0.997647914	d__Bacteria	p__Firmicutes_A	c__Clostridia	o__Lachnospirales	f__Lachnospiraceae	g__Agathobacter	s__Agathobacter unknown_3
MGYG-HGUT-01189:s__Blautia_A unknown_10	0.268018609	0.30909419	0.920494192	d__Bacteria	p__Firmicutes_A	c__Clostridia	o__Lachnospirales	f__Lachnospiraceae	g__Blautia_A	s__Blautia_A unknown_10
MGYG-HGUT-01190:s__Collinsella unknown_133	0.496074504	0.188203097	0.968532073	d__Bacteria	p__Actinobacteriota	c__Coriobacteriia	o__Coriobacteriales	f__Coriobacteriaceae	g__Collinsella	s__Collinsella unknown_133
MGYG-HGUT-01191:s__Anaerovoracaceae unknown unknown_4	0.8424981	0.054582705	0.992878088	d__Bacteria	p__Firmicutes_A	c__Clostridia	o__Peptostreptococcales	f__Anaerovoracaceae	g__Anaerovoracaceae unknown	s__Anaerovoracaceae unknown unknown_4
MGYG-HGUT-01193:s__UBA4285 unknown_1	0.532305291	0.169994894	0.968532073	d__Bacteria	p__Firmicutes_A	c__Clostridia	o__Lachnospirales	f__Lachnospiraceae	g__UBA4285	s__UBA4285 unknown_1

MGYG-HGUT-01195:s__CAG-508 unknown unknown_5	0.613323753	-0.141850752	0.975671757	d__Bacteria	p__Firmicutes_A	c__Clostridia	o__TANB77	f__CAG-508	g__CAG-508 unknown	s__CAG-508 unknown unknown_5
MGYG-HGUT-01197:s__Streptococcus unknown_7	0.945987793	0.018906419	0.994479474	d__Bacteria	p__Firmicutes	c__Bacilli	o__Lactobacillales	f__Streptococcaceae	g__Streptococcus	s__Streptococcus unknown_7
MGYG-HGUT-01199:s__Lachnospiraceae unknown unknown_13	0.673657001	-0.116453018	0.975671757	d__Bacteria	p__Firmicutes_A	c__Clostridia	o__Lachnospirales	f__Lachnospiraceae	g__Lachnospiraceae unknown	s__Lachnospiraceae unknown unknown_13
MGYG-HGUT-01200:s__Collinsella unknown_135	0.408223663	0.230177116	0.939709925	d__Bacteria	p__Actinobacteriota	c__Coriobacteriia	o__Coriobacteriales	f__Coriobacteriaceae	g__Collinsella	s__Collinsella unknown_135
MGYG-HGUT-01202:s__Ruminococcus_D unknown_1	0.880379885	-0.040193771	0.994479474	d__Bacteria	p__Firmicutes_A	c__Clostridia	o__Oscillospirales	f__Ruminococcaceae	g__Ruminococcus_D	s__Ruminococcus_D unknown_1
MGYG-HGUT-01204:s__NK3B98 unknown_1	0.293393455	0.286644292	0.929025913	d__Bacteria	p__Firmicutes_A	c__Clostridia	o__Oscillospirales	f__Oscillospiraceae	g__NK3B98	s__NK3B98 unknown_1
MGYG-HGUT-01205:s__Gemmiger unknown_6	0.543746903	0.165055619	0.968532073	d__Bacteria	p__Firmicutes_A	c__Clostridia	o__Oscillospirales	f__Ruminococcaceae	g__Gemmiger	s__Gemmiger unknown_6
MGYG-HGUT-01210:s__Faecalicatena unknown_3	0.636241639	0.132576544	0.975671757	d__Bacteria	p__Firmicutes_A	c__Clostridia	o__Lachnospirales	f__Lachnospiraceae	g__Faecalicatena	s__Faecalicatena unknown_3
MGYG-HGUT-01212:s__Acutalibacter unknown_3	0.184885225	0.368587117	0.891825559	d__Bacteria	p__Firmicutes_A	c__Clostridia	o__Oscillospirales	f__Acutalibacteraceae	g__Acutalibacter	s__Acutalibacter unknown_3

MGYG-HGUT-01216:s__CAG-354 unknown_3	0.841948622	0.054574698	0.992878088	d__Bacteria	p__Firmicutes_A	c__Clostridia	o__TANB77	f__CAG-508	g__CAG-354	s__CAG-354 unknown_3
MGYG-HGUT-01219:s__CAG-56 unknown_3	0.264415392	0.310988751	0.914203083	d__Bacteria	p__Firmicutes_A	c__Clostridia	o__Lachnospirales	f__Lachnospiraceae	g__CAG-56	s__CAG-56 unknown_3
MGYG-HGUT-01224:s__Blautia_A unknown_11	0.747938056	0.089715599	0.979458475	d__Bacteria	p__Firmicutes_A	c__Clostridia	o__Lachnospirales	f__Lachnospiraceae	g__Blautia_A	s__Blautia_A unknown_11
MGYG-HGUT-01225:s__Ruminiclostridium_C unknown_2	0.186156133	0.361182124	0.891825559	d__Bacteria	p__Firmicutes_A	c__Clostridia	o__Oscillospirales	f__Oscillospiraceae	g__Ruminiclostridium_C	s__Ruminiclostridium_C unknown_2
MGYG-HGUT-01226:s__Oscillospiraceae unknown unknown_4	0.132019924	0.401736151	0.891825559	d__Bacteria	p__Firmicutes_A	c__Clostridia	o__Oscillospirales	f__Oscillospiraceae	g__Oscillospiraceae unknown	s__Oscillospiraceae unknown unknown_4
MGYG-HGUT-01229:s__D5 sp900113995	0.857155383	0.047819831	0.992878088	d__Bacteria	p__Firmicutes_A	c__Clostridia	o__Oscillospirales	f__Ruminococcaceae	g__D5	s__D5 sp900113995
MGYG-HGUT-01230:s__Eubacterium_R unknown_5	0.654457894	-0.125089446	0.975671757	d__Bacteria	p__Firmicutes_A	c__Clostridia	o__Oscillospirales	f__Acutalibacteraceae	g__Eubacterium_R	s__Eubacterium_R unknown_5
MGYG-HGUT-01237:s__CAG-74 unknown unknown_7	0.994089727	0.0020693	0.998803346	d__Bacteria	p__Firmicutes_A	c__Clostridia	o__Christensenellales	f__CAG-74	g__CAG-74 unknown	s__CAG-74 unknown unknown_7
MGYG-HGUT-01239:s__CAG-776 unknown_1	0.399961596	0.2350572	0.93970925	d__Bacteria	p__Firmicutes	c__Bacilli	o__RF39	f__CAG-1000	g__CAG-776	s__CAG-776 unknown_1

MGYG-HGUT-01240:s__Prevotella unknown_17	0.60403382 6	-0.14482883 9	0.97567175 7	d__Bacteria	p__Bacteroidota	c__Bacteroidia	o__Bacteroidales	f__Bacteroidaceae	g__Prevotella	s__Prevotella unknown_17
MGYG-HGUT-01244:s__UBA737 unknown_7	0.84630030 8	-0.05445284 9	0.99287808 8	d__Bacteria	p__Firmicutes_A	c__Clostridia	o__Oscillospirales	f__Acutalibacteraceae	g__UBA737	s__UBA737 unknown_7
MGYG-HGUT-01246:s__Clostridium_M unknown_6	0.65060345 7	0.11991919 8	0.97567175 7	d__Bacteria	p__Firmicutes_A	c__Clostridia	o__Lachnospirales	f__Lachnospiraceae	g__Clostridium_M	s__Clostridium_M unknown_6
MGYG-HGUT-01247:s__Lachnospiraceae unknown unknown_14	0.08834800 4	0.46917156 4	0.88271606 4	d__Bacteria	p__Firmicutes_A	c__Clostridia	o__Lachnospirales	f__Lachnospiraceae	g__Lachnospiraceae unknown	s__Lachnospiraceae unknown unknown_14
MGYG-HGUT-01248:s__Dialister unknown_6	0.36224779 3	0.25151332 6	0.93970925	d__Bacteria	p__Firmicutes_C	c__Negativicutes	o__Veillonellales	f__Dialisteraceae	g__Dialister	s__Dialister unknown_6
MGYG-HGUT-01252:s__Lachnospiraceae unknown unknown_15	0.12682025 2	-0.42547058 4	0.89182555 9	d__Bacteria	p__Firmicutes_A	c__Clostridia	o__Lachnospirales	f__Lachnospiraceae	g__Lachnospiraceae unknown	s__Lachnospiraceae unknown unknown_15
MGYG-HGUT-01254:s__TF01-11 unknown_3	0.17620094 6	-0.36859205 3	0.89182555 9	d__Bacteria	p__Firmicutes_A	c__Clostridia	o__Lachnospirales	f__Lachnospiraceae	g__TF01-11	s__TF01-11 unknown_3
MGYG-HGUT-01255:s__Faecalibacterium prausnitzii_F	0.40626526 6	-0.23154095 2	0.93970925	d__Bacteria	p__Firmicutes_A	c__Clostridia	o__Oscillospirales	f__Ruminococcaceae	g__Faecalibacterium	s__Faecalibacterium prausnitzii_F
MGYG-HGUT-01257:s__Massilio	0.50226	0.18829	0.96853	d__B act	p__Firmicutes	c__Bacilli	o__Erysipelot	f__Erysipelatocl	g__Massiliomicrobiota	s__Massiliomicrobiota timonensis

microbiota timonensis	8307	2077	2073	eria			richales	ostridiaceae		
MGYG-HGUT-01273:s__Pauljensenia unknown_2	0.38999777	0.236445497	0.93970925	d__Bacteria	p__Actinobacteriota	c__Actinobacteria	o__Actinomycetales	f__Actinomycetaceae	g__Pauljensenia	s__Pauljensenia unknown_2
MGYG-HGUT-01274:s__Streptococcus unknown_8	0.346925425	0.262376602	0.93970925	d__Bacteria	p__Firmicutes	c__Bacilli	o__Lactobacillales	f__Streptococcaceae	g__Streptococcus	s__Streptococcus unknown_8
MGYG-HGUT-01284:s__Pauljensenia unknown_3	0.313443088	-0.278424842	0.929025913	d__Bacteria	p__Actinobacteriota	c__Actinobacteria	o__Actinomycetales	f__Actinomycetaceae	g__Pauljensenia	s__Pauljensenia unknown_3
MGYG-HGUT-01285:s__Pauljensenia unknown_4	0.037309237	-0.576711591	0.768225834	d__Bacteria	p__Actinobacteriota	c__Actinobacteria	o__Actinomycetales	f__Actinomycetaceae	g__Pauljensenia	s__Pauljensenia unknown_4
MGYG-HGUT-01286:s__Oscillospiraceae unknown unknown_5	0.853601678	0.051430879	0.992878088	d__Bacteria	p__Firmicutes_A	c__Clostridia	o__Oscillospirales	f__Oscillospiraceae	g__Oscillospiraceae unknown	s__Oscillospiraceae unknown unknown_5
MGYG-HGUT-01289:s__Streptococcus unknown_9	0.171392457	0.368222946	0.891825559	d__Bacteria	p__Firmicutes	c__Bacilli	o__Lactobacillales	f__Streptococcaceae	g__Streptococcus	s__Streptococcus unknown_9
MGYG-HGUT-01292:s__Bifidobacterium infantis	0.541827196	0.168173818	0.968532073	d__Bacteria	p__Actinobacteriota	c__Actinobacteria	o__Actinomycetales	f__Bifidobacteriaceae	g__Bifidobacterium	s__Bifidobacterium infantis
MGYG-HGUT-01293:s__Lactobacillus_C rhamnosus	0.184458877	-0.340414049	0.891825559	d__Bacteria	p__Firmicutes	c__Bacilli	o__Lactobacillales	f__Lactobacillaceae	g__Lactobacillus_C	s__Lactobacillus_C rhamnosus
MGYG-HGUT-01294:s__Parasutt	0.76060	-0.085	0.97945	d__Bact	p__Proteob	c__Gammaproteo	o__Burkholderiales	f__Burkholderiaceae	g__Parasutterella	s__Parasutterella

erella excrementihominis	4926	091361	8475	eria	acteria	bacteria				excrementihominis
MGYG-HGUT-01299:s__Absiella dolichum	0.577635169	0.154121327	0.975671757	d__Bacteria	p__Firmicutes	c__Bacilli	o__Erysipelotrichales	f__Erysipelotrichaceae	g__Absiella	s__Absiella dolichum
MGYG-HGUT-01300:s__Faecalibacterium prausnitzii_K	0.041401783	0.544091269	0.779321052	d__Bacteria	p__Firmicutes_A	c__Clostridia	o__Oscillospirales	f__Ruminococcaceae	g__Faecalibacterium	s__Faecalibacterium prausnitzii_K
MGYG-HGUT-01302:s__Alistipes putredinis	0.732584273	-0.095214172	0.979458475	d__Bacteria	p__Bacteroidota	c__Bacteroidia	o__Bacteroidales	f__Rikenellaceae	g__Alistipes	s__Alistipes putredinis
MGYG-HGUT-01303:s__Dorea scindens	0.910149708	0.03132617	0.994479474	d__Bacteria	p__Firmicutes_A	c__Clostridia	o__Lachnospirales	f__Lachnospiraceae	g__Dorea	s__Dorea scindens
MGYG-HGUT-01304:s__Erysipelatoclostridium spiroforme	0.653523378	-0.125333772	0.975671757	d__Bacteria	p__Firmicutes	c__Bacilli	o__Erysipelotrichales	f__Erysipelatoclostridiaceae	g__Erysipelatoclostridium	s__Erysipelatoclostridium spiroforme
MGYG-HGUT-01306:s__Bacteroides_A coprocola	0.319230281	-0.274133148	0.929025913	d__Bacteria	p__Bacteroidota	c__Bacteroidia	o__Bacteroidales	f__Bacteroidaceae	g__Bacteroides_A	s__Bacteroides_A coprocola
MGYG-HGUT-01308:s__Streptococcus infantarius	0.913336253	0.029850021	0.994479474	d__Bacteria	p__Firmicutes	c__Bacilli	o__Lactobacillales	f__Streptococcaceae	g__Streptococcus	s__Streptococcus infantarius
MGYG-HGUT-01310:s__Faecalicatena lactaris	0.876522128	0.042526352	0.994479474	d__Bacteria	p__Firmicutes_A	c__Clostridia	o__Lachnospirales	f__Lachnospiraceae	g__Faecalicatena	s__Faecalicatena lactaris

MGYG-HGUT-01311:s__Clostridium_M sp000155435	0.39536166	-0.238223331	0.93970925	d__Bacteria	p__Firmicutes_A	c__Clostridia	o__Lachnospirales	f__Lachnospiraceae	g__Clostridium_M	s__Clostridium_M sp000155435
MGYG-HGUT-01313:s__Bacteroides eggerthii	0.271355954	0.307270274	0.925406661	d__Bacteria	p__Bacteroidota	c__Bacteroidia	o__Bacteroidales	f__Bacteroidaceae	g__Bacteroides	s__Bacteroides eggerthii
MGYG-HGUT-01314:s__Bacteroides_F pectinophilus	0.018861985	0.622310094	0.768225834	d__Bacteria	p__Firmicutes_A	c__Clostridia	o__Lachnospirales	f__Lachnospiraceae	g__Bacteroides_F	s__Bacteroides_F pectinophilus
MGYG-HGUT-01315:s__Coprococcus_B comes	0.28134946	0.294842836	0.929025913	d__Bacteria	p__Firmicutes_A	c__Clostridia	o__Lachnospirales	f__Lachnospiraceae	g__Coprococcus_B	s__Coprococcus_B comes
MGYG-HGUT-01316:s__Mitsuokella multacida	0.300752184	0.288169599	0.929025913	d__Bacteria	p__Firmicutes_C	c__Negativicutes	o__Selenomonadales	f__Selenomonadaceae	g__Mitsuokella	s__Mitsuokella multacida
MGYG-HGUT-01319:s__Butyrivibrio_A crossotus	0.762410038	-0.082463855	0.979458475	d__Bacteria	p__Firmicutes_A	c__Clostridia	o__Lachnospirales	f__Lachnospiraceae	g__Butyrivibrio_A	s__Butyrivibrio_A crossotus
MGYG-HGUT-01322:s__Gemmiger variabile	0.547946282	0.168068972	0.968532073	d__Bacteria	p__Firmicutes_A	c__Clostridia	o__Oscillospirales	f__Ruminococcaceae	g__Gemmiger	s__Gemmiger variabile
MGYG-HGUT-01332:s__Massilioclostridium methylpentosum	0.533283428	-0.158552939	0.968532073	d__Bacteria	p__Firmicutes_A	c__Clostridia	o__Oscillospirales	f__Ruminococcaceae	g__Massilioclostridium	s__Massilioclostridium methylpentosum
MGYG-HGUT-01334:s__Lactobacillus_B ruminis	0.735819941	0.085937892	0.979458475	d__Bacteria	p__Firmicutes	c__Bacilli	o__Lactobacillales	f__Lactobacillaceae	g__Lactobacillus_B	s__Lactobacillus_B ruminis

MGYG-HGUT-01337:s__Bacteroides fragilis	0.569579895	-0.155247538	0.973855764	d__Bacteria	p__Bacteroidota	c__Bacteroidia	o__Bacteroidales	f__Bacteroidaceae	g__Bacteroides	s__Bacteroides fragilis
MGYG-HGUT-01338:s__Blautia_A wexlerae	0.247589166	0.297531523	0.902181367	d__Bacteria	p__Firmicutes_A	c__Clostridia	o__Lachnospirales	f__Lachnospiraceae	g__Blautia_A	s__Blautia_A wexlerae
MGYG-HGUT-01342:s__Streptococcus gordonii	0.291910284	0.28383642	0.929025913	d__Bacteria	p__Firmicutes	c__Bacilli	o__Lactobacillales	f__Streptococcaceae	g__Streptococcus	s__Streptococcus gordonii
MGYG-HGUT-01345:s__Bacteroides xylanisolvens	0.306867302	-0.286129591	0.929025913	d__Bacteria	p__Bacteroidota	c__Bacteroidia	o__Bacteroidales	f__Bacteroidaceae	g__Bacteroides	s__Bacteroides xylanisolvens
MGYG-HGUT-01346:s__Bacteroides uniformis	0.859272077	-0.049809436	0.992878088	d__Bacteria	p__Bacteroidota	c__Bacteroidia	o__Bacteroidales	f__Bacteroidaceae	g__Bacteroides	s__Bacteroides uniformis
MGYG-HGUT-01349:s__Veillonella parvula_A	0.468576801	0.201992259	0.968194079	d__Bacteria	p__Firmicutes_C	c__Negativicutes	o__Veillonellales	f__Veillonellaceae	g__Veillonella	s__Veillonella parvula_A
MGYG-HGUT-01352:s__Absiella sp000165065	0.002226661	0.82817175	0.648124372	d__Bacteria	p__Firmicutes	c__Bacilli	o__Erysipelotrichales	f__Erysipelotrichaceae	g__Absiella	s__Absiella sp000165065
MGYG-HGUT-01353:s__Collinsella aerofaciens_2	0.195285751	0.347778235	0.891825559	d__Bacteria	p__Actinobacteriota	c__Coriobacteriia	o__Coriobacteriales	f__Coriobacteriaceae	g__Collinsella	s__Collinsella aerofaciens_2
MGYG-HGUT-01354:s__Bifidobacterium dentium	0.718466964	-0.10031	0.979458475	d__Bacteria	p__Actinobacteriota	c__Actinobacteria	o__Actinomycetales	f__Bifidobacteriaceae	g__Bifidobacterium	s__Bifidobacterium dentium

		3276								
MGYG-HGUT-01356:s__Lawsonibacter sp000177015	0.334660027	0.267311275	0.930624692	d__Bacteria	p__Firmicutes_A	c__Clostridia	o__Oscillospirales	f__Oscillospiraceae	g__Lawsonibacter	s__Lawsonibacter sp000177015
MGYG-HGUT-01359:s__Bilophila wadsworthia	0.022019342	0.620225375	0.768225834	d__Bacteria	p__Desulfobacterota_A	c__Desulfovibrionia	o__Desulfovibrionales	f__Desulfovibrionaceae	g__Bilophila	s__Bilophila wadsworthia
MGYG-HGUT-01360:s__Prevotella salivae	0.190066939	-0.352110994	0.891825559	d__Bacteria	p__Bacteroidota	c__Bacteroidia	o__Bacteroidales	f__Bacteroidaceae	g__Prevotella	s__Prevotella salivae
MGYG-HGUT-01361:s__Sutterella wadsworthensis_B	0.162273096	0.37500834	0.891825559	d__Bacteria	p__Proteobacteria	c__Gammaproteobacteria	o__Burkholderiales	f__Burkholderiaceae	g__Sutterella	s__Sutterella wadsworthensis_B
MGYG-HGUT-01362:s__Streptococcus anginosus	0.076427978	0.484650743	0.877826247	d__Bacteria	p__Firmicutes	c__Bacilli	o__Lactobacillales	f__Streptococcaceae	g__Streptococcus	s__Streptococcus anginosus
MGYG-HGUT-01364:s__Bacteroides_A plebeius	0.156181807	-0.395414257	0.891825559	d__Bacteria	p__Bacteroidota	c__Bacteroidia	o__Bacteroidales	f__Bacteroidaceae	g__Bacteroides_A	s__Bacteroides_A plebeius
MGYG-HGUT-01365:s__Phascolarctobacterium_A succinatutens	0.833975783	-0.057865608	0.99222019	d__Bacteria	p__Firmicutes_C	c__Negativicutes	o__Acidaminococcales	f__Acidaminococcaceae	g__Phascolarctobacterium_A	s__Phascolarctobacterium_A succinatutens
MGYG-HGUT-01367:s__Clostridium_Q symbiosum	0.245381397	0.320250288	0.902181367	d__Bacteria	p__Firmicutes_A	c__Clostridia	o__Lachnospirales	f__Lachnospiraceae	g__Clostridium_Q	s__Clostridium_Q symbiosum
MGYG-HGUT-01368:s__Faecalicatena fissicatena	0.45673	-0.197	0.96557	d__Bact	p__Firmicutes_A	c__Clostridia	o__Lachnospirales	f__Lachnospiraceae	g__Faecalicatena	s__Faecalicatena fissicatena

	6227	857143	4692	eria						
MGYG-HGUT-01369:s__Lactobacillus delbrueckii	0.319726322	0.276803781	0.929025913	d__Bacteria	p__Firmicutes	c__Bacilli	o__Lactobacillales	f__Lactobacillaceae	g__Lactobacillus	s__Lactobacillus delbrueckii
MGYG-HGUT-01370:s__Bacteroides fluxus	0.671437891	0.115608283	0.975671757	d__Bacteria	p__Bacteroidota	c__Bacteroidia	o__Bacteroidales	f__Bacteroidaceae	g__Bacteroides	s__Bacteroides fluxus
MGYG-HGUT-01372:s__Paraprevotella xylaniphila	0.781777707	-0.075379194	0.980737328	d__Bacteria	p__Bacteroidota	c__Bacteroidia	o__Bacteroidales	f__Bacteroidaceae	g__Paraprevotella	s__Paraprevotella xylaniphila
MGYG-HGUT-01373:s__Tyzzerella sp000209385	0.850141216	-0.053126139	0.992878088	d__Bacteria	p__Firmicutes_A	c__Clostridia	o__Lachnospirales	f__Lachnospiraceae	g__Tyzzerella	s__Tyzzerella sp000209385
MGYG-HGUT-01374:s__Faecalicatena torques	0.213827368	0.346841161	0.891825559	d__Bacteria	p__Firmicutes_A	c__Clostridia	o__Lachnospirales	f__Lachnospiraceae	g__Faecalicatena	s__Faecalicatena torques
MGYG-HGUT-01375:s__Ruminococcus_F champanellensis	0.605335219	-0.142161685	0.975671757	d__Bacteria	p__Firmicutes_A	c__Clostridia	o__Oscillospirales	f__Ruminococcaceae	g__Ruminococcus_F	s__Ruminococcus_F champanellensis
MGYG-HGUT-01378:s__Bacteroides ovatus	0.885399273	0.039813433	0.994479474	d__Bacteria	p__Bacteroidota	c__Bacteroidia	o__Bacteroidales	f__Bacteroidaceae	g__Bacteroides	s__Bacteroides ovatus
MGYG-HGUT-01379:s__Faecalicatena glycyrrhizinilyticum	0.183690434	-0.368785195	0.891825559	d__Bacteria	p__Firmicutes_A	c__Clostridia	o__Lachnospirales	f__Lachnospiraceae	g__Faecalicatena	s__Faecalicatena glycyrrhizinilyticum

MGYG-HGUT-01380:s__Faecalicatena gnavus	0.470493865	-0.19955927	0.9681940779	d__Bacteria	p__Firmicutes_A	c__Clostridia	o__Lachnospirales	f__Lachnospiraceae	g__Faecalicatena	s__Faecalicatena gnavus
MGYG-HGUT-01385:s__Alistipes_A indistinctus	0.432303322	0.2106383378	0.9508065 94	d__Bacteria	p__Bacteroidota	c__Bacteroidia	o__Bacteroidales	f__Rikenellaceae	g__Alistipes_A	s__Alistipes_A indistinctus
MGYG-HGUT-01386:s__Clostridium_M clostridioforme	0.8459618 46	0.0538301 83	0.9928780 88	d__Bacteria	p__Firmicutes_A	c__Clostridia	o__Lachnospirales	f__Lachnospiraceae	g__Clostridium_M	s__Clostridium_M clostridioforme
MGYG-HGUT-01387:s__Senegalimassilia anaerobia	0.5864523 45	0.1503702 07	0.9756717 57	d__Bacteria	p__Actinobacteriota	c__Coriobacteriia	o__Coriobacteriales	f__Eggerthellaceae	g__Senegalimassilia	s__Senegalimassilia anaerobia
MGYG-HGUT-01391:s__Coprobacter fastidiosus	0.7704804 18	-0.080470412	0.9795204 84	d__Bacteria	p__Bacteroidota	c__Bacteroidia	o__Bacteroidales	f__Coprobacteraceae	g__Coprobacter	s__Coprobacter fastidiosus
MGYG-HGUT-01396:s__Absiella innocuum	0.5325100 69	-0.17176433	0.9685320 73	d__Bacteria	p__Firmicutes	c__Bacilli	o__Erysipelotrichales	f__Erysipelotrichaceae	g__Absiella	s__Absiella innocuum
MGYG-HGUT-01397:s__Dialister succinatiphilus	0.7240208 91	0.0982839 34	0.9794584 75	d__Bacteria	p__Firmicutes_C	c__Negativicutes	o__Veillonellales	f__Dialisteraceae	g__Dialister	s__Dialister succinatiphilus
MGYG-HGUT-01398:s__Absiella sp000163515	0.8541045 27	0.0509631 41	0.9928780 88	d__Bacteria	p__Firmicutes	c__Bacilli	o__Erysipelotrichales	f__Erysipelotrichaceae	g__Absiella	s__Absiella sp000163515
MGYG-HGUT-01399:s__Odoribacter laneus	0.0842879 71	-0.46836	0.8827160 64	d__Bacteria	p__Bacteroidota	c__Bacteroidia	o__Bacteroidales	f__Marinifilaceae	g__Odoribacter	s__Odoribacter laneus

		7244								
MGYG-HGUT-01400:s__Erysipelatoclostridium ramosum	0.747922367	0.089803547	0.979458475	d__Bacteria	p__Firmicutes	c__Bacilli	o__Erysipelotrichales	f__Erysipelatoclostridiaceae	g__Erysipelatoclostridium	s__Erysipelatoclostridium ramosum
MGYG-HGUT-01402:s__Coprobacillus cateniformis	0.812364553	-0.066528465	0.987142302	d__Bacteria	p__Firmicutes	c__Bacilli	o__Erysipelotrichales	f__Erysipelatoclostridiaceae	g__Coprobacillus	s__Coprobacillus cateniformis
MGYG-HGUT-01411:s__Pauljensenia sp000308055	0.402583597	0.231143505	0.93970925	d__Bacteria	p__Actinobacteriota	c__Actinobacteria	o__Actinomycetales	f__Actinomycetaceae	g__Pauljensenia	s__Pauljensenia sp000308055
MGYG-HGUT-01413:s__Enorma massiliensis	0.7302748 8	0.095470083	0.979458475	d__Bacteria	p__Actinobacteriota	c__Coriobacteriia	o__Coriobacteriales	f__Coriobacteriaceae	g__Enorma	s__Enorma massiliensis
MGYG-HGUT-01415:s__Alistipes obesi	0.816201094	-0.063307011	0.987664539	d__Bacteria	p__Bacteroidota	c__Bacteroidia	o__Bacteroidales	f__Rikenellaceae	g__Alistipes	s__Alistipes obesi
MGYG-HGUT-01420:s__Alistipes senegalensis	0.718913644	0.099148874	0.979458475	d__Bacteria	p__Bacteroidota	c__Bacteroidia	o__Bacteroidales	f__Rikenellaceae	g__Alistipes	s__Alistipes senegalensis
MGYG-HGUT-01421:s__Dielma fastidiosa	0.107659463	0.441848924	0.883307256	d__Bacteria	p__Firmicutes	c__Bacilli	o__Erysipelotrichales	f__Erysipelotrichaceae	g__Dielma	s__Dielma fastidiosa
MGYG-HGUT-01422:s__Bacteroides oleiciplenus	0.137537175	-0.413386555	0.891825559	d__Bacteria	p__Bacteroidota	c__Bacteroidia	o__Bacteroidales	f__Bacteroidaceae	g__Bacteroides	s__Bacteroides oleiciplenus
MGYG-HGUT-01429:s__Alistipes_A ihumii_1	0.39574	-0.225	0.93970	d__Bact	p__Bacter	c__Bacteroidia	o__Bacteroidales	f__Rikenellaceae	g__Alistipes_A	s__Alistipes_A ihumii_1

	0378	842998	925	eria	oidota					
MGYG-HGUT-01430:s__Holdemania massiliensis	0.100046684	0.455560981	0.883307256	d__Bacteria	p__Firmicutes	c__Bacilli	o__Erysipelotrichales	f__Erysipelotrichaceae	g__Holdemania	s__Holdemania massiliensis
MGYG-HGUT-01433:s__Bacteroides salyersiae	0.555170748	-0.163010166	0.968532073	d__Bacteria	p__Bacteroidota	c__Bacteroidia	o__Bacteroidales	f__Bacteroidaceae	g__Bacteroides	s__Bacteroides salyersiae
MGYG-HGUT-01434:s__Butyricicoccus pullicaecorum	0.680364351	-0.115162442	0.975671757	d__Bacteria	p__Firmicutes_A	c__Clostridia	o__Oscillospirales	f__Butyricicoccaceae	g__Butyricicoccus	s__Butyricicoccus pullicaecorum
MGYG-HGUT-01439:s__Tyzzerella sp000411335	0.190783631	-0.350814337	0.891825559	d__Bacteria	p__Firmicutes_A	c__Clostridia	o__Lachnospirales	f__Lachnospiraceae	g__Tyzzerella	s__Tyzzerella sp000411335
MGYG-HGUT-01440:s__Acidaminococcus intestini	0.900813385	0.034553834	0.994479474	d__Bacteria	p__Firmicutes_C	c__Negativicutes	o__Acidaminococcales	f__Acidaminococcaceae	g__Acidaminococcus	s__Acidaminococcus intestini
MGYG-HGUT-01441:s__Pauljensenia sp000411415	0.48116688	0.194612298	0.968532073	d__Bacteria	p__Actinobacteriota	c__Actinobacteria	o__Actinomycetales	f__Actinomycetaceae	g__Pauljensenia	s__Pauljensenia sp000411415
MGYG-HGUT-01442:s__Streptococcus sp000411475	0.246547966	0.321302432	0.902181367	d__Bacteria	p__Firmicutes	c__Bacilli	o__Lactobacillales	f__Streptococcaceae	g__Streptococcus	s__Streptococcus sp000411475
MGYG-HGUT-01444:s__Veillonella atypica	0.643267043	0.12841876	0.975671757	d__Bacteria	p__Firmicutes_C	c__Negativicutes	o__Veillonellales	f__Veillonellaceae	g__Veillonella	s__Veillonella atypica
MGYG-HGUT-01454:s__Bittarella massiliensis	0.87263	-0.042	0.99447	d__Bact	p__Firmicutes_A	c__Clostridia	o__Oscillospirales	f__Ruminococcaceae	g__Bittarella	s__Bittarella massiliensis

	2734	565826	9474	eria						
MGYG-HGUT-01455:s__Faecalitalea cylindroides	0.290343395	-0.293461903	0.929025913	d__Bacteria	p__Firmicutes	c__Bacilli	o__Erysipelotrichales	f__Erysipelotrichaceae	g__Faecalitalea	s__Faecalitalea cylindroides
MGYG-HGUT-01456:s__Eubacterium_I ramulus	0.790177736	-0.074216588	0.982969663	d__Bacteria	p__Firmicutes_A	c__Clostridia	o__Lachnospirales	f__Lachnospiraceae	g__Eubacterium_I	s__Eubacterium_I ramulus
MGYG-HGUT-01478:s__Collinsella sp000763055_2	0.461754219	0.205167821	0.965574692	d__Bacteria	p__Actinobacteriota	c__Coriobacteriia	o__Coriobacteriales	f__Coriobacteriaceae	g__Collinsella	s__Collinsella sp000763055_2
MGYG-HGUT-01489:s__Parabacteroides goldsteinii	0.122736375	-0.387336794	0.885742175	d__Bacteria	p__Bacteroidota	c__Bacteroidia	o__Bacteroidales	f__Tannerellaceae	g__Parabacteroides	s__Parabacteroides goldsteinii
MGYG-HGUT-01490:s__Bifidobacterium catenulatum	0.100225832	0.442685743	0.883307256	d__Bacteria	p__Actinobacteriota	c__Actinobacteria	o__Actinomycetales	f__Bifidobacteriaceae	g__Bifidobacterium	s__Bifidobacterium catenulatum
MGYG-HGUT-01493:s__Clostridium_M bolteae	0.805997996	0.067473855	0.987142302	d__Bacteria	p__Firmicutes_A	c__Clostridia	o__Lachnospirales	f__Lachnospiraceae	g__Clostridium_M	s__Clostridium_M bolteae
MGYG-HGUT-01496:s__Dorea sp001185345	0.264489644	0.306314566	0.914203083	d__Bacteria	p__Firmicutes_A	c__Clostridia	o__Lachnospirales	f__Lachnospiraceae	g__Dorea	s__Dorea sp001185345
MGYG-HGUT-01499:s__Faecalicatena contorta_B	0.900959924	-0.033971571	0.994479474	d__Bacteria	p__Firmicutes_A	c__Clostridia	o__Lachnospirales	f__Lachnospiraceae	g__Faecalicatena	s__Faecalicatena contorta_B

MGYG-HGUT-01500:s__Lawsonibacter asaccharolyticus	0.998232105	0.000614961	0.998843763	d__Bacteria	p__Firmicutes_A	c__Clostridia	o__Oscillospirales	f__Oscillospiraceae	g__Lawsonibacter	s__Lawsonibacter asaccharolyticus
MGYG-HGUT-01502:s__Intestinimonas massiliensis	0.470165639	0.198248843	0.968194079	d__Bacteria	p__Firmicutes_A	c__Clostridia	o__Oscillospirales	f__Oscillospiraceae	g__Intestinimonas	s__Intestinimonas massiliensis
MGYG-HGUT-01512:s__Coprobacter secundus	0.714531843	-0.102662103	0.979458475	d__Bacteria	p__Bacteroidota	c__Bacteroidia	o__Bacteroidales	f__Coprobacteraceae	g__Coprobacter	s__Coprobacter secundus
MGYG-HGUT-01527:s__Fournierella massiliensis	0.519874774	-0.179539055	0.968532073	d__Bacteria	p__Firmicutes_A	c__Clostridia	o__Oscillospirales	f__Ruminococcaceae	g__Fournierella	s__Fournierella massiliensis
MGYG-HGUT-01528:s__Faecalicatena sp001487105	0.496230284	-0.190483613	0.968532073	d__Bacteria	p__Firmicutes_A	c__Clostridia	o__Lachnospirales	f__Lachnospiraceae	g__Faecalicatena	s__Faecalicatena sp001487105
MGYG-HGUT-01531:s__Clostridium_M sp001517625	0.920874711	0.027751695	0.994479474	d__Bacteria	p__Firmicutes_A	c__Clostridia	o__Lachnospirales	f__Lachnospiraceae	g__Clostridium_M	s__Clostridium_M sp001517625
MGYG-HGUT-01539:s__Acutalibacter timonensis	0.099177592	0.459649481	0.883307256	d__Bacteria	p__Firmicutes_A	c__Clostridia	o__Oscillospirales	f__Acutalibacteraceae	g__Acutalibacter	s__Acutalibacter timonensis
MGYG-HGUT-01546:s__Alistipes sp900083545	0.250364562	0.32147869	0.902181367	d__Bacteria	p__Bacteroidota	c__Bacteroidia	o__Bacteroidales	f__Rikenellaceae	g__Alistipes	s__Alistipes sp900083545
MGYG-HGUT-01547:s__Emergencia timonensis	0.063700555	0.483620432	0.85721132	d__Bacteria	p__Firmicutes_A	c__Clostridia	o__Peptostreptococcales	f__Anaerovoracaceae	g__Emergencia	s__Emergencia timonensis

MGYG-HGUT-01550:s__Christensenella_A timonensis	0.482367799	0.194034748	0.968532073	d__Bacteria	p__Firmicutes_A	c__Clostridia	o__Christensenellales	f__Christensenellaceae	g__Christensenella_A	s__Christensenella_A timonensis
MGYG-HGUT-01551:s__Odoribacter massiliensis	0.596278267	0.147043832	0.975671757	d__Bacteria	p__Bacteroidota	c__Bacteroidia	o__Bacteroidales	f__Marinifilaceae	g__Odoribacter	s__Odoribacter massiliensis
MGYG-HGUT-01558:s__Streptococcus timonensis	0.744082911	0.09170672	0.979458475	d__Bacteria	p__Firmicutes	c__Bacilli	o__Lactobacillales	f__Streptococcaceae	g__Streptococcus	s__Streptococcus timonensis
MGYG-HGUT-01560:s__Massiliolostridium coli	0.519019399	-0.172600471	0.968532073	d__Bacteria	p__Firmicutes_A	c__Clostridia	o__Oscillospirales	f__Ruminococcaceae	g__Massilioclostridium	s__Massilioclostridium coli
MGYG-HGUT-01562:s__Alistipes timonensis	0.693581863	0.110115656	0.97824056	d__Bacteria	p__Bacteroidota	c__Bacteroidia	o__Bacteroidales	f__Rikenellaceae	g__Alistipes	s__Alistipes timonensis
MGYG-HGUT-01564:s__Clostridium_M clostridioforme_A	0.337465344	-0.263117018	0.930624692	d__Bacteria	p__Firmicutes_A	c__Clostridia	o__Lachnospirales	f__Lachnospiraceae	g__Clostridium_M	s__Clostridium_M clostridioforme_A
MGYG-HGUT-01565:s__CAG-103 sp900317855_2	0.684939134	0.113079772	0.975768579	d__Bacteria	p__Firmicutes_A	c__Clostridia	o__Oscillospirales	f__Oscillospiraceae	g__CAG-103	s__CAG-103 sp900317855_2
MGYG-HGUT-01566:s__Tyzzerella unknown_3	0.189711298	0.338781087	0.891825559	d__Bacteria	p__Firmicutes_A	c__Clostridia	o__Lachnospirales	f__Lachnospiraceae	g__Tyzzerella	s__Tyzzerella unknown_3
MGYG-HGUT-01569:s__Eubacterium_R coprostanoligenes	0.315732419	0.281278483	0.929025913	d__Bacteria	p__Firmicutes_A	c__Clostridia	o__Oscillospirales	f__Acutalibacteraceae	g__Eubacterium_R	s__Eubacterium_R coprostanoligenes

MGYG-HGUT-01571:s__Hungatella sp000526575	0.309426766	-0.284443668	0.929025913	d__Bacteria	p__Firmicutes_A	c__Clostridia	o__Lachnospirales	f__Lachnospiraceae	g__Hungatella	s__Hungatella sp000526575
MGYG-HGUT-01576:s__Oscillibacter unknown_8	0.558789349	0.156964992	0.968532073	d__Bacteria	p__Firmicutes_A	c__Clostridia	o__Oscillospirales	f__Oscillospiraceae	g__Oscillibacter	s__Oscillibacter unknown_8
MGYG-HGUT-01577:s__CAG-145 unknown_3	0.533461712	-0.167913355	0.968532073	d__Bacteria	p__Firmicutes_A	c__Clostridia	o__Peptostreptococcales	f__Anaerovoraceae	g__CAG-145	s__CAG-145 unknown_3
MGYG-HGUT-01579:s__Collinsella unknown_147	0.221535217	0.333473146	0.891825559	d__Bacteria	p__Actinobacteriota	c__Coriobacteriia	o__Coriobacteriales	f__Coriobacteriaceae	g__Collinsella	s__Collinsella unknown_147
MGYG-HGUT-01580:s__Collinsella unknown_148	0.359590183	0.25152753	0.93970925	d__Bacteria	p__Actinobacteriota	c__Coriobacteriia	o__Coriobacteriales	f__Coriobacteriaceae	g__Collinsella	s__Collinsella unknown_148
MGYG-HGUT-01582:s__Collinsella unknown_150	0.488241114	0.19226759	0.968532073	d__Bacteria	p__Actinobacteriota	c__Coriobacteriia	o__Coriobacteriales	f__Coriobacteriaceae	g__Collinsella	s__Collinsella unknown_150
MGYG-HGUT-01583:s__Collinsella unknown_151	0.080849875	0.466080292	0.877826247	d__Bacteria	p__Actinobacteriota	c__Coriobacteriia	o__Coriobacteriales	f__Coriobacteriaceae	g__Collinsella	s__Collinsella unknown_151
MGYG-HGUT-01587:s__Collinsella unknown_155	0.254295049	0.315110107	0.905015696	d__Bacteria	p__Actinobacteriota	c__Coriobacteriia	o__Coriobacteriales	f__Coriobacteriaceae	g__Collinsella	s__Collinsella unknown_155
MGYG-HGUT-01594:s__Blautia_A unknown_12	0.403541888	0.225396693	0.93970925	d__Bacteria	p__Firmicutes_A	c__Clostridia	o__Lachnospirales	f__Lachnospiraceae	g__Blautia_A	s__Blautia_A unknown_12

MGYG-HGUT-01596:s__Blautia unknown_4	0.2463664 97	-0.3239544 1	0.9021813 67	d__Bacteria	p__Firmicutes_A	c__Clostridia	o__Lachnospirales	f__Lachnospiraceae	g__Blautia	s__Blautia unknown_4
MGYG-HGUT-01597:s__UBA866 unknown	0.6305938 19	-0.1261089 36	0.9756717 57	d__Bacteria	p__Firmicutes_A	c__Clostridia	o__Oscillospirales	f__Ruminococcaceae	g__UBA866	s__UBA866 unknown
MGYG-HGUT-01599:s__Bacteroides_A unknown_4	0.5644968 47	0.1599635 02	0.9713390 3	d__Bacteria	p__Bacteroidota	c__Bacteroidia	o__Bacteroidales	f__Bacteroidaceae	g__Bacteroides_A	s__Bacteroides_A unknown_4
MGYG-HGUT-01602:s__Blautia unknown_5	0.1430571 85	0.4068375 13	0.8918255 59	d__Bacteria	p__Firmicutes_A	c__Clostridia	o__Lachnospirales	f__Lachnospiraceae	g__Blautia	s__Blautia unknown_5
MGYG-HGUT-01603:s__Lachnospiraceae unknown unknown_16	0.3766387 79	0.2454141 4	0.9397092 5	d__Bacteria	p__Firmicutes_A	c__Clostridia	o__Lachnospirales	f__Lachnospiraceae	g__Lachnospiraceae unknown	s__Lachnospiraceae unknown unknown_16
MGYG-HGUT-01606:s__Clostridium_M unknown_8	0.8385780 38	-0.0566530 8	0.9928780 88	d__Bacteria	p__Firmicutes_A	c__Clostridia	o__Lachnospirales	f__Lachnospiraceae	g__Clostridium_M	s__Clostridium_M unknown_8
MGYG-HGUT-01607:s__Dorea sp900240315	0.5796575 52	0.1555328 15	0.9756717 57	d__Bacteria	p__Firmicutes_A	c__Clostridia	o__Lachnospirales	f__Lachnospiraceae	g__Dorea	s__Dorea sp900240315
MGYG-HGUT-01610:s__Sporanaerobacter acetigenes	0.9242748 82	0.0265129 19	0.9944794 74	d__Bacteria	p__Firmicutes_A	c__Clostridia	o__Tissierellales	f__Sporanaerobacteraceae	g__Sporanaerobacter	s__Sporanaerobacter acetigenes
MGYG-HGUT-01619:s__UBA9502 unknown_5	0.0573154 33	0.5156365 02	0.8572113 2	d__Bacteria	p__Firmicutes_A	c__Clostridia	o__Lachnospirales	f__Lachnospiraceae	g__UBA9502	s__UBA9502 unknown_5

MGYG-HGUT-01622:s__Clostridium_M unknown_9	0.0012926664	0.859058025	0.6481243 72	d__Bacteria	p__Firmicutes_A	c__Clostridia	o__Lachnospirales	f__Lachnospiraceae	g__Clostridium_M	s__Clostridium_M unknown_9
MGYG-HGUT-01626:s__Phascolarctobacterium unknown_2	0.9795549 85	0.0071748 79	0.9976479 14	d__Bacteria	p__Firmicutes_C	c__Negativicutes	o__Acidaminococcales	f__Acidaminococcaceae	g__Phascolarctobacterium	s__Phascolarctobacterium unknown_2
MGYG-HGUT-01627:s__Gemmiger unknown_7	0.1138336 35	0.4243268 28	0.8833072 56	d__Bacteria	p__Firmicutes_A	c__Clostridia	o__Oscillospirales	f__Ruminococcaceae	g__Gemmiger	s__Gemmiger unknown_7
MGYG-HGUT-01631:s__Bilophila unknown_2	0.2228632 17	0.3397334 01	0.8918255 59	d__Bacteria	p__Desulfobacterota_A	c__Desulfovibrionia	o__Desulfovibrionales	f__Desulfovibrionaceae	g__Bilophila	s__Bilophila unknown_2
MGYG-HGUT-01632:s__CAG-170 unknown_4	0.1182264 68	0.4091435 33	0.8833072 56	d__Bacteria	p__Firmicutes_A	c__Clostridia	o__Oscillospirales	f__Oscillospiraceae	g__CAG-170	s__CAG-170 unknown_4
MGYG-HGUT-01634:s__Collinsella sp002232035_22	0.5147261 91	0.1794028 48	0.9685320 73	d__Bacteria	p__Actinobacteriota	c__Coriobacteriia	o__Coriobacteriales	f__Coriobacteriaceae	g__Collinsella	s__Collinsella sp002232035_22
MGYG-HGUT-01635:s__KLE1796 unknown	0.1358457 83	-0.3905105 66	0.8918255 59	d__Bacteria	p__Firmicutes	c__Bacilli	o__Erysipelotrichales	f__Erysipelatoclostridiaceae	g__KLE1796	s__KLE1796 unknown
MGYG-HGUT-01637:s__Agathobacter unknown_4	0.9638136 9	0.0124561 05	0.9944794 74	d__Bacteria	p__Firmicutes_A	c__Clostridia	o__Lachnospirales	f__Lachnospiraceae	g__Agathobacter	s__Agathobacter unknown_4
MGYG-HGUT-01642:s__Collinsella unknown_158	0.7480129 63	0.0893912 65	0.9794584 75	d__Bacteria	p__Actinobacteriota	c__Coriobacteriia	o__Coriobacteriales	f__Coriobacteriaceae	g__Collinsella	s__Collinsella unknown_158

MGYG-HGUT-01645:s__Eubacterium_G unknown_2	0.877819689	-0.0417967 81	0.994479474	d__Bacteria	p__Firmicutes_A	c__Clostridia	o__Lachnospirales	f__Lachnospiraceae	g__Eubacterium_G	s__Eubacterium_G unknown_2
MGYG-HGUT-01652:s__V9D3004 sp002349525	0.813688087	0.064456336	0.987142302	d__Bacteria	p__Firmicutes_A	c__Clostridia	o__Lachnospirales	f__Lachnospiraceae	g__V9D3004	s__V9D3004 sp002349525
MGYG-HGUT-01654:s__Acutalibacteraceae unknown unknown_15	0.946969894	0.018443411	0.994479474	d__Bacteria	p__Firmicutes_A	c__Clostridia	o__Oscillospirales	f__Acutalibacteraceae	g__Acutalibacteraceae unknown	s__Acutalibacteraceae unknown unknown_15
MGYG-HGUT-01655:s__Butyricimonas sp900258545	0.117788584	0.435582751	0.883307256	d__Bacteria	p__Bacteroidota	c__Bacteroidia	o__Bacteroidales	f__Marinifilaceae	g__Butyricimonas	s__Butyricimonas sp900258545
MGYG-HGUT-01658:s__CAG-74 unknown unknown_8	0.114007873	0.430280356	0.883307256	d__Bacteria	p__Firmicutes_A	c__Clostridia	o__Christensenellales	f__CAG-74	g__CAG-74 unknown	s__CAG-74 unknown unknown_8
MGYG-HGUT-01659:s__Paraprevotella unknown_3	0.967583754	-0.0114044 28	0.994479474	d__Bacteria	p__Bacteroidota	c__Bacteroidia	o__Bacteroidales	f__Bacteroidaceae	g__Paraprevotella	s__Paraprevotella unknown_3
MGYG-HGUT-01661:s__Bacteroides gallinarum	0.337886785	0.266872438	0.930624692	d__Bacteria	p__Bacteroidota	c__Bacteroidia	o__Bacteroidales	f__Bacteroidaceae	g__Bacteroides	s__Bacteroides gallinarum
MGYG-HGUT-01665:s__QALW01 unknown unknown_1	0.976211414	0.008385292	0.996587801	d__Bacteria	p__Firmicutes_A	c__Clostridia	o__Christensenellales	f__QALW01	g__QALW01 unknown	s__QALW01 unknown unknown_1
MGYG-HGUT-01670:s__Bacteroides_B sartorii	0.396935402	0.236130867	0.93970925	d__Bacteria	p__Bacteroidota	c__Bacteroidia	o__Bacteroidales	f__Bacteroidaceae	g__Bacteroides_B	s__Bacteroides_B sartorii

MGYG-HGUT-01671:s__Clostridia unknown unknown unknown unknown_1	0.606727517	0.144425 23	0.975671757	d__Bacteria	p__Firmicutes_A	c__Clostridia	o__Clostridia unknown	f__Clostridia unknown unknown	g__Clostridia unknown unknown unknown	s__Clostridia unknown unknown unknown unknown_1
MGYG-HGUT-01673:s__UBA1255 unknown unknown_5	0.858791025	0.049783387	0.992878088	d__Bacteria	p__Firmicutes_A	c__Clostridia	o__UBA1212	f__UBA1255	g__UBA1255 unknown	s__UBA1255 unknown unknown_5
MGYG-HGUT-01677:s__CAG-452 sp000434035	0.996496435	-0.001231314	0.998803346	d__Bacteria	p__Firmicutes_A	c__Clostridia	o__TANB77	f__CAG-508	g__CAG-452	s__CAG-452 sp000434035
MGYG-HGUT-01681:s__Ruminococcus unknown_2	0.223126389	0.335649241	0.891825559	d__Bacteria	p__Firmicutes_A	c__Clostridia	o__Oscillospirales	f__Ruminococcaceae	g__Ruminococcus	s__Ruminococcus unknown_2
MGYG-HGUT-01683:s__UBA7096 unknown_2	0.713646168	0.103098187	0.979458475	d__Bacteria	p__Firmicutes_A	c__Clostridia	o__Lachnospirales	f__Lachnospiraceae	g__UBA7096	s__UBA7096 unknown_2
MGYG-HGUT-01687:s__Dorea sp000433535	0.298498912	0.289272928	0.929025913	d__Bacteria	p__Firmicutes_A	c__Clostridia	o__Lachnospirales	f__Lachnospiraceae	g__Dorea	s__Dorea sp000433535
MGYG-HGUT-01689:s__Blautia producta	0.433116954	-0.216761851	0.950806594	d__Bacteria	p__Firmicutes_A	c__Clostridia	o__Lachnospirales	f__Lachnospiraceae	g__Blautia	s__Blautia producta
MGYG-HGUT-01690:s__Ruminococcus_G gauvreauii	0.131125114	-0.391728504	0.891825559	d__Bacteria	p__Firmicutes_A	c__Clostridia	o__Lachnospirales	f__Lachnospiraceae	g__Ruminococcus_G	s__Ruminococcus_G gauvreauii
MGYG-HGUT-01694:s__Enterococcus faecalis	0.08314	-0.46133	0.88114	d__Bact	p__Firmicutes	c__Bacilli	o__Lactobacillales	f__Enterococcaceae	g__Enterococcus	s__Enterococcus faecalis

	1721	938	2843	eria						
MGYG-HGUT-01697:s__Erysipelatoclostridium saccharogumia	0.299188389	0.288998332	0.929025913	d__Bacteria	p__Firmicutes	c__Bacilli	o__Erysipelotrichales	f__Erysipelatoclostridiaceae	g__Erysipelatoclostridium	s__Erysipelatoclostridium saccharogumia
MGYG-HGUT-01698:s__Marvinbryantia formatexigens	0.003989574	0.75461836	0.648124372	d__Bacteria	p__Firmicutes_A	c__Clostridia	o__Lachnospirales	f__Lachnospiraceae	g__Marvinbryantia	s__Marvinbryantia formatexigens
MGYG-HGUT-01704:s__Blautia hansenii	0.848384151	0.052960516	0.992878088	d__Bacteria	p__Firmicutes_A	c__Clostridia	o__Lachnospirales	f__Lachnospiraceae	g__Blautia	s__Blautia hansenii
MGYG-HGUT-01705:s__Citrobacter portucalensis	0.927811663	0.024719106	0.994479474	d__Bacteria	p__Proteobacteria	c__Gammaproteobacteria	o__Enterobacterales	f__Enterobacteriaceae	g__Citrobacter	s__Citrobacter portucalensis
MGYG-HGUT-01707:s__Coprococcus sp000154245	0.644585295	-0.128876018	0.975671757	d__Bacteria	p__Firmicutes_A	c__Clostridia	o__Lachnospirales	f__Lachnospiraceae	g__Coprococcus	s__Coprococcus sp000154245
MGYG-HGUT-01711:s__Holdemanella biformis	0.351395705	0.258700973	0.93970925	d__Bacteria	p__Firmicutes	c__Bacilli	o__Erysipelotrichales	f__Erysipelotrichaceae	g__Holdemanella	s__Holdemanella biformis
MGYG-HGUT-01714:s__Coprococcus_A catus	0.850822003	0.05250137	0.992878088	d__Bacteria	p__Firmicutes_A	c__Clostridia	o__Lachnospirales	f__Lachnospiraceae	g__Coprococcus_A	s__Coprococcus_A catus
MGYG-HGUT-01718:s__Collinsella unknown_164	0.037849702	0.551601738	0.768225834	d__Bacteria	p__Actinobacteriota	c__Coriobacteriia	o__Coriobacteriales	f__Coriobacteriaceae	g__Collinsella	s__Collinsella unknown_164
MGYG-HGUT-01720:s__Prevotella sp002350355	0.970555296	0.009716893	0.994485272	d__Bacteria	p__Bacteroidota	c__Bacteroidia	o__Bacteroidales	f__Bacteroidaceae	g__Prevotella	s__Prevotella sp002350355

MGYG-HGUT-01722:s__Eubacterium_F unknown_1	0.177895679	0.3602491 83	0.8918255 59	d__Bacteria	p__Firmicutes_A	c__Clostridia	o__Lachnospirales	f__Lachnospiraceae	g__Eubacterium_F	s__Eubacterium_F unknown_1
MGYG-HGUT-01727:s__UBA1191 unknown_2	0.3631704 79	-0.2392424 5414	0.9397092 5	d__Bacteria	p__Firmicutes_A	c__Clostridia	o__Peptostreptococcales	f__Anaerovoraceae	g__UBA1191	s__UBA1191 unknown_2
MGYG-HGUT-01732:s__UBA1381 unknown	0.7409926 91	-0.0925737 36	0.9794584 75	d__Bacteria	p__Firmicutes_A	c__Clostridia	o__Monoglobales	f__UBA1381	g__UBA1381	s__UBA1381 unknown
MGYG-HGUT-01733:s__CAG-83 unknown_4	0.8632617 66	-0.0476325 32	0.9947947 4 74	d__Bacteria	p__Firmicutes_A	c__Clostridia	o__Oscillospirales	f__Oscillospiraceae	g__CAG-83	s__CAG-83 unknown_4
MGYG-HGUT-01734:s__Collinsella unknown_169	0.9320524 77	0.0237753 4	0.9944794 74	d__Bacteria	p__Actinobacteriota	c__Coriobacteriia	o__Coriobacteriales	f__Coriobacteriaceae	g__Collinsella	s__Collinsella unknown_169
MGYG-HGUT-01736:s__Collinsella unknown_170	0.4818069 55	0.1921159 58	0.9685320 73	d__Bacteria	p__Actinobacteriota	c__Coriobacteriia	o__Coriobacteriales	f__Coriobacteriaceae	g__Collinsella	s__Collinsella unknown_170
MGYG-HGUT-01737:s__Eubacterium_R sp000434995	0.4301599 47	-0.2182227 097	0.9508065 94	d__Bacteria	p__Firmicutes_A	c__Clostridia	o__Oscillospirales	f__Acutalibacteraceae	g__Eubacterium_R	s__Eubacterium_R sp000434995
MGYG-HGUT-01742:s__Oscillibacter unknown_10	0.2450211 85	-0.3005998 45	0.9021813 67	d__Bacteria	p__Firmicutes_A	c__Clostridia	o__Oscillospirales	f__Oscillospiraceae	g__Oscillibacter	s__Oscillibacter unknown_10
MGYG-HGUT-01744:s__Collinsella unknown_172	0.52779	0.17133	0.96853	d__Bact	p__Actino	c__Coriobacteriia	o__Coriobacteriales	f__Coriobacteriaceae	g__Collinsella	s__Collinsella unknown_172

	8906	8502	2073	eria	bacteriota					
MGYG-HGUT-01745:s__Coprococcus unknown_2	0.275966092	0.291564567	0.929025913	d__Bacteria	p__Firmicutes_A	c__Clostridia	o__Lachnospirales	f__Lachnospiraceae	g__Coprococcus	s__Coprococcus unknown_2
MGYG-HGUT-01748:s__CAG-56 unknown_4	0.760070441	0.08523798	0.979458475	d__Bacteria	p__Firmicutes_A	c__Clostridia	o__Lachnospirales	f__Lachnospiraceae	g__CAG-56	s__CAG-56 unknown_4
MGYG-HGUT-01749:s__An200 unknown_1	0.915176717	-0.028116463	0.994479474	d__Bacteria	p__Firmicutes_A	c__Clostridia	o__Oscillospirales	f__Acutalibacteraceae	g__An200	s__An200 unknown_1
MGYG-HGUT-01750:s__Bacteroides_A ilei	0.656395317	-0.125058473	0.975671757	d__Bacteria	p__Bacteroidota	c__Bacteroidia	o__Bacteroidales	f__Bacteroidaceae	g__Bacteroides_A	s__Bacteroides_A ilei
MGYG-HGUT-01751:s__Gemmiger unknown_8	0.545201281	0.16870439	0.968532073	d__Bacteria	p__Firmicutes_A	c__Clostridia	o__Oscillospirales	f__Ruminococcaceae	g__Gemmiger	s__Gemmiger unknown_8
MGYG-HGUT-01753:s__Collinsella unknown_173	0.034389372	0.570661344	0.768225834	d__Bacteria	p__Actinobacteriota	c__Coriobacteriia	o__Coriobacteriales	f__Coriobacteriaceae	g__Collinsella	s__Collinsella unknown_173
MGYG-HGUT-01754:s__CAG-110 unknown_8	0.727579279	-0.093392211	0.979458475	d__Bacteria	p__Firmicutes_A	c__Clostridia	o__Oscillospirales	f__Oscillospiraceae	g__CAG-110	s__CAG-110 unknown_8
MGYG-HGUT-01755:s__CAG-245 unknown_3	0.329862439	-0.272020384	0.930624692	d__Bacteria	p__Firmicutes_A	c__Clostridia	o__TANB77	f__CAG-508	g__CAG-245	s__CAG-245 unknown_3

MGYG-HGUT-01757:s__Ruminococcus unknown_3	0.6580185 97	-0.1218388 64	0.9756717 57	d__Bacteria	p__Firmicutes_A	c__Clostridia	o__Oscillospirales	f__Ruminococcaceae	g__Ruminococcus	s__Ruminococcus unknown_3
MGYG-HGUT-01758:s__Blautia_A unknown_13	0.9312693 61	0.0236501 09	0.9944794 74	d__Bacteria	p__Firmicutes_A	c__Clostridia	o__Lachnospirales	f__Lachnospiraceae	g__Blautia_A	s__Blautia_A unknown_13
MGYG-HGUT-01761:s__Gemmiger unknown_9	0.7491534 43	-0.0886254 79	0.9794584 75	d__Bacteria	p__Firmicutes_A	c__Clostridia	o__Oscillospirales	f__Ruminococcaceae	g__Gemmiger	s__Gemmiger unknown_9
MGYG-HGUT-01763:s__Prevotella sp000434975	0.9526922 57	-0.0164688 2	0.9944794 74	d__Bacteria	p__Bacteroidota	c__Bacteroidia	o__Bacteroidales	f__Bacteroidaceae	g__Prevotella	s__Prevotella sp000434975
MGYG-HGUT-01770:s__Prevotella sp900313215	0.9699392 67	0.0098924 93	0.9944794 74	d__Bacteria	p__Bacteroidota	c__Bacteroidia	o__Bacteroidales	f__Bacteroidaceae	g__Prevotella	s__Prevotella sp900313215
MGYG-HGUT-01777:s__Eubacterium_F sp000434115	0.6757429 24	-0.1159722 52	0.9756717 57	d__Bacteria	p__Firmicutes_A	c__Clostridia	o__Lachnospirales	f__Lachnospiraceae	g__Eubacterium_F	s__Eubacterium_F sp000434115
MGYG-HGUT-01780:s__Bacteroides unknown_2	0.4653297 76	0.2036764 31	0.9680376 13	d__Bacteria	p__Bacteroidota	c__Bacteroidia	o__Bacteroidales	f__Bacteroidaceae	g__Bacteroides	s__Bacteroides unknown_2
MGYG-HGUT-01787:s__Bacteroides_A unknown_6	0.6681271 42	0.1204215 49	0.9756717 57	d__Bacteria	p__Bacteroidota	c__Bacteroidia	o__Bacteroidales	f__Bacteroidaceae	g__Bacteroides_A	s__Bacteroides_A unknown_6
MGYG-HGUT-01789:s__Bacteroides_A sp002161565	0.01391	0.67868	0.76822	d__Bact	p__Bacteroidota	c__Bacteroidia	o__Bacteroidales	f__Bacteroidaceae	g__Bacteroides_A	s__Bacteroides_A sp002161565

	03 39	89 68	58 34	eri a						
MGYG-HGUT-01793:s__CAG-632 sp002406015	0.8 75 58 69 82	0.0 42 44 26 04	0.9 94 47 94 74	d__B act eri a	p__Fi rmicu tes_A	c__Clo stridia	o__Lac hnospi rales	f__Lach nospirac eae	g__CAG-632	s__CAG-632 sp002406015
MGYG-HGUT-01795:s__K10 sp001941205	0.3 27 99 24 87	0.2 73 99 78 55	0.9 30 62 46 92	d__B act eri a	p__B acter oidot a	c__Ba cteroi dia	o__Bac teroida les	f__Muri baculac eae	g__K10	s__K10 sp001941205
MGYG-HGUT-01797:s__CAG-590 unknown_3	0.1 56 23 83 48	0.3 78 79 57 14	0.8 91 82 55 59	d__B act eri a	p__Fi rmicu tes_A	c__Clo stridia	o__Lac hnospi rales	f__Lach nospirac eae	g__CAG-590	s__CAG-590 unknown_3
MGYG-HGUT-01806:s__Prevotella unknown_19	0.4 95 43 52 44	-0.1 91 36 17 05	0.9 68 53 20 73	d__B act eri a	p__B acter oidot a	c__Ba cteroi dia	o__Bac teroida les	f__Bact eroidac eae	g__Prevot ella	s__Prevotella unknown_19
MGYG-HGUT-01807:s__Bacteroides unknown_3	0.5 13 83 02 99	0.1 82 73 61 16	0.9 68 53 20 73	d__B act eri a	p__B acter oidot a	c__Ba cteroi dia	o__Bac teroida les	f__Bact eroidac eae	g__Bacter oides	s__Bacteroides unknown_3
MGYG-HGUT-01811:s__UBA11774 unknown	0.3 29 01 15 25	-0.2 70 65 58 99	0.9 30 62 46 92	d__B act eri a	p__Fi rmicu tes_A	c__Clo stridia	o__Lac hnospi rales	f__Lach nospirac eae	g__UBA11 774	s__UBA11774 unknown
MGYG-HGUT-01814:s__COE1 unknown	0.0 80 77 08 18	0.4 83 92 92 16	0.8 77 82 62 47	d__B act eri a	p__Fi rmicu tes_A	c__Clo stridia	o__Lac hnospi rales	f__Lach nospirac eae	g__COE1	s__COE1 unknown
MGYG-HGUT-01819:s__CAG-145 unknown_5	0.3 04 08 09 18	-0.2 87 10 19 98	0.9 29 02 59 13	d__B act eri a	p__Fi rmicu tes_A	c__Clo stridia	o__Pe ptostre ptococ cales	f__Anae rovorac aceae	g__CAG-145	s__CAG-145 unknown_5
MGYG-HGUT-01820:s__Anaeroti	0.0 65 23	-0.4 90	0.8 57 21	d__B act	p__Fi rmicu tes_A	c__Clo stridia	o__Lac hnospi rales	f__Anae rotignac eae	g__Anaero tignum	s__Anaerotig num

gnum lactatifermentans	8243	465231	132	eria						lactatifermentans
MGYG-HGUT-01823:s__CAG-74 unknown unknown_9	0.512183161	-0.180803315	0.968532073	d__Bacteria	p__Firmicutes_A	c__Clostridia	o__Christensenellales	f__CAG-74	g__CAG-74 unknown	s__CAG-74 unknown unknown_9
MGYG-HGUT-01824:s__CAG-83 unknown_5	0.384681229	0.232494079	0.93970925	d__Bacteria	p__Firmicutes_A	c__Clostridia	o__Oscillospirales	f__Oscillospiraceae	g__CAG-83	s__CAG-83 unknown_5
MGYG-HGUT-01825:s__CAG-269 unknown_16	0.091934814	0.468948651	0.883307256	d__Bacteria	p__Firmicutes_A	c__Clostridia	o__TANB77	f__CAG-508	g__CAG-269	s__CAG-269 unknown_16
MGYG-HGUT-01830:s__Alistipes finegoldii	0.478899898	-0.18341103	0.968532073	d__Bacteria	p__Bacteroidota	c__Bacteroidia	o__Bacteroidales	f__Rikenellaceae	g__Alistipes	s__Alistipes finegoldii
MGYG-HGUT-01831:s__TF01-11 sp000436755	0.82308156	0.062784785	0.991136622	d__Bacteria	p__Firmicutes_A	c__Clostridia	o__Lachnospirales	f__Lachnospiraceae	g__TF01-11	s__TF01-11 sp000436755
MGYG-HGUT-01835:s__Bacteroides_A sp000432735	0.545997115	0.167838435	0.968532073	d__Bacteria	p__Bacteroidota	c__Bacteroidia	o__Bacteroidales	f__Bacteroidaceae	g__Bacteroides_A	s__Bacteroides_A sp000432735
MGYG-HGUT-01841:s__CAG-170 unknown_5	0.224578344	0.330275152	0.891825559	d__Bacteria	p__Firmicutes_A	c__Clostridia	o__Oscillospirales	f__Oscillospiraceae	g__CAG-170	s__CAG-170 unknown_5
MGYG-HGUT-01848:s__Eubacterium_F sp000433735	0.794350203	-0.073101721	0.9829663	d__Bacteria	p__Firmicutes_A	c__Clostridia	o__Lachnospirales	f__Lachnospiraceae	g__Eubacterium_F	s__Eubacterium_F sp000433735

MGYG-HGUT-01850:s__QALW01 unknown unknown_2	0.981017839	-0.0066809065	0.997647914	d__Bacteria	p__Firmicutes_A	c__Clostridia	o__Christensenellales	f__QALW01	g__QALW01 unknown	s__QALW01 unknown unknown_2
MGYG-HGUT-01855:s__CAG-632 sp000431515	0.645959516	0.124860624	0.975671757	d__Bacteria	p__Firmicutes_A	c__Clostridia	o__Lachnospirales	f__Lachnospiraceae	g__CAG-632	s__CAG-632 sp000431515
MGYG-HGUT-01866:s__Prevotella sp003447235	0.300511624	0.276820548	0.929025913	d__Bacteria	p__Bacteroidota	c__Bacteroidia	o__Bacteroidales	f__Bacteroidaceae	g__Prevotella	s__Prevotella sp003447235
MGYG-HGUT-01876:s__Acetatifactor unknown_4	0.240816999	0.320173966	0.902021367	d__Bacteria	p__Firmicutes_A	c__Clostridia	o__Lachnospirales	f__Lachnospiraceae	g__Acetatifactor	s__Acetatifactor unknown_4
MGYG-HGUT-01878:s__Parabacteroides unknown_7	0.384889304	-0.241728434	0.93970925	d__Bacteria	p__Bacteroidota	c__Bacteroidia	o__Bacteroidales	f__Tannerellaceae	g__Parabacteroides	s__Parabacteroides unknown_7
MGYG-HGUT-01881:s__Akkermansia muciniphila_A	0.557341825	-0.155114785	0.968532073	d__Bacteria	p__Verrucomicrobiota	c__Verrucomicrobiae	o__Verrucomicrobiales	f__Akkermansiaceae	g__Akkermansia	s__Akkermansia muciniphila_A
MGYG-HGUT-01887:s__Ruminiclostridium_E sp003512525	0.516148808	0.176297088	0.968532073	d__Bacteria	p__Firmicutes_A	c__Clostridia	o__Oscillospirales	f__Ruminococcaceae	g__Ruminiclostridium_E	s__Ruminiclostridium_E sp003512525
MGYG-HGUT-01899:s__Zag1 unknown_8	0.851359344	-0.050814941	0.992878088	d__Bacteria	p__Cyanobacteria	c__Vampirovibrionia	o__Gastranaerophilales	f__Gastranaerophilaceae	g__Zag1	s__Zag1 unknown_8
MGYG-HGUT-01906:s__CAG-484 unknown_2	0.69689	0.10950	0.97945	d__Bact	p__Cyanobacteria	c__Vampirovibrionia	o__Gastranaerophilales	f__Gastranaerophilaceae	g__CAG-484	s__CAG-484 unknown_2

	4098	7692	8475	eria						
MGYG-HGUT-01908:s__Ruminococcus_D unknown_3	0.509156702	-0.182188076	0.968532073	d__Bacteria	p__Firmicutes_A	c__Clostridia	o__Oscillospirales	f__Ruminococcaceae	g__Ruminococcus_D	s__Ruminococcus_D unknown_3
MGYG-HGUT-01918:s__Prevotellamassilia unknown_6	0.242066023	0.325652341	0.902181367	d__Bacteria	p__Bacteroidota	c__Bacteroidia	o__Bacteroidales	f__Bacteroidaceae	g__Prevotellamassilia	s__Prevotellamassilia unknown_6
MGYG-HGUT-01919:s__F23-B02 sp002472405	0.570035327	0.155578722	0.973855764	d__Bacteria	p__Firmicutes_A	c__Clostridia	o__Oscillospirales	f__Oscillospiraceae	g__F23-B02	s__F23-B02 sp002472405
MGYG-HGUT-01920:s__Bacteroides_A unknown_7	0.553512229	-0.164424146	0.968532073	d__Bacteria	p__Bacteroidota	c__Bacteroidia	o__Bacteroidales	f__Bacteroidaceae	g__Bacteroides_A	s__Bacteroides_A unknown_7
MGYG-HGUT-01921:s__Akkermansia unknown_2	0.860664882	-0.046840455	0.993545313	d__Bacteria	p__Verrucomicrobiota	c__Verrucomicrobiae	o__Verrucomicrobiales	f__Akkermansiaceae	g__Akkermansia	s__Akkermansia unknown_2
MGYG-HGUT-01925:s__Bacteroides_A unknown_8	0.581562566	0.154755446	0.975671757	d__Bacteria	p__Bacteroidota	c__Bacteroidia	o__Bacteroidales	f__Bacteroidaceae	g__Bacteroides_A	s__Bacteroides_A unknown_8
MGYG-HGUT-01927:s__Prevotella unknown_21	0.679867982	-0.113591762	0.975671757	d__Bacteria	p__Bacteroidota	c__Bacteroidia	o__Bacteroidales	f__Bacteroidaceae	g__Prevotella	s__Prevotella unknown_21
MGYG-HGUT-01929:s__Paramuribaculum unknown_4	0.667191021	0.120775223	0.975671757	d__Bacteria	p__Bacteroidota	c__Bacteroidia	o__Bacteroidales	f__Muribaculaceae	g__Paramuribaculum	s__Paramuribaculum unknown_4

MGYG-HGUT-01931:s__CAG-279 unknown_3	0.3708385569	0.2429980044	0.939709 25	d__Bacteria	p__Bacteroidota	c__Bacteroidia	o__Bacteroidales	f__Muribaculaceae	g__CAG-279	s__CAG-279 unknown_3
MGYG-HGUT-01932:s__QAND01 unknown unknown_1	0.6379338 61	0.1275002 88	0.9756717 57	d__Bacteria	p__Firmicutes_A	c__Clostridia	o__Christensenellales	f__QAND01	g__QAND01 unknown	s__QAND01 unknown unknown_1
MGYG-HGUT-01933:s__Prevotella unknown_22	0.6090169 36	0.1358827 51	0.9756717 57	d__Bacteria	p__Bacteroidota	c__Bacteroidia	o__Bacteroidales	f__Bacteroidaceae	g__Prevotella	s__Prevotella unknown_22
MGYG-HGUT-01936:s__UBA5905 sp002437905	0.8793563 91	0.0420302 78	0.9944794 74	d__Bacteria	p__Firmicutes_A	c__Clostridia	o__Oscillospirales	f__Acutalibacteraceae	g__UBA5905	s__UBA5905 sp002437905
MGYG-HGUT-01940:s__Ruminococcus sp002438605	0.5658801 31	0.1551250 23	0.97133903	d__Bacteria	p__Firmicutes_A	c__Clostridia	o__Oscillospirales	f__Ruminococcaceae	g__Ruminococcus	s__Ruminococcus sp002438605
MGYG-HGUT-01942:s__CAG-269 unknown_17	0.9958057 44	0.0014586 35	0.9988033 46	d__Bacteria	p__Firmicutes_A	c__Clostridia	o__TANB77	f__CAG-508	g__CAG-269	s__CAG-269 unknown_17
MGYG-HGUT-01943:s__CAG-484 unknown_3	0.7248870 36	-0.0909313 26	0.9794584 75	d__Bacteria	p__Cyanobacteria	c__Vampirovibrionia	o__Gastranaerophilales	f__Gastranaerophilaceae	g__CAG-484	s__CAG-484 unknown_3
MGYG-HGUT-01947:s__UBA1777 unknown_6	0.3487624 54	0.2550220 73	0.93970925	d__Bacteria	p__Firmicutes_A	c__Clostridia	o__Oscillospirales	f__Oscillospiraceae	g__UBA1777	s__UBA1777 unknown_6
MGYG-HGUT-01949:s__Oscillibacter unknown_11	0.0433311 39	0.5520054 38	0.7793210 52	d__Bacteria	p__Firmicutes_A	c__Clostridia	o__Oscillospirales	f__Oscillospiraceae	g__Oscillibacter	s__Oscillibacter unknown_11

MGYG-HGUT-01950:s__Gastranaerophilus phascolarctosicola	0.364139661	-0.247182841	0.939709925	d__Bacteria	p__Cyanobacteria	c__Vampirovibrionia	o__Gastranaerophilales	f__Gastranaerophilaceae	g__Gastranaerophilus	s__Gastranaerophilus phascolarctosicola
MGYG-HGUT-01954:s__Clostridium_M unknown_10	0.421188068	0.224366787	0.942006355	d__Bacteria	p__Firmicutes_A	c__Clostridia	o__Lachnospirales	f__Lachnospiraceae	g__Clostridium_M	s__Clostridium_M unknown_10
MGYG-HGUT-01963:s__Bacteroides_A sp000434735	0.927842385	-0.025090906	0.994479474	d__Bacteria	p__Bacteroidota	c__Bacteroidia	o__Bacteroidales	f__Bacteroidaceae	g__Bacteroides_A	s__Bacteroides_A sp000434735
MGYG-HGUT-01964:s__Acetatifactor unknown_5	0.036849634	0.562720058	0.768225834	d__Bacteria	p__Firmicutes_A	c__Clostridia	o__Lachnospirales	f__Lachnospiraceae	g__Acetatifactor	s__Acetatifactor unknown_5
MGYG-HGUT-01965:s__CAG-510 unknown_1	0.884175699	0.040457302	0.994479474	d__Bacteria	p__Firmicutes_A	c__Clostridia	o__Lachnospirales	f__Lachnospiraceae	g__CAG-510	s__CAG-510 unknown_1
MGYG-HGUT-01969:s__CAG-110 unknown_9	0.962651893	-0.012958035	0.994479474	d__Bacteria	p__Firmicutes_A	c__Clostridia	o__Oscillospirales	f__Oscillospiraceae	g__CAG-110	s__CAG-110 unknown_9
MGYG-HGUT-01971:s__Clostridium_M unknown_11	0.218776902	0.338565002	0.891825559	d__Bacteria	p__Firmicutes_A	c__Clostridia	o__Lachnospirales	f__Lachnospiraceae	g__Clostridium_M	s__Clostridium_M unknown_11
MGYG-HGUT-01972:s__Prevotella sp900315955	0.675110605	0.10813114	0.975671757	d__Bacteria	p__Bacteroidota	c__Bacteroidia	o__Bacteroidales	f__Bacteroidaceae	g__Prevotella	s__Prevotella sp900315955
MGYG-HGUT-01975:s__Prevotella unknown_23	0.533431981	-0.169611973	0.968532073	d__Bacteria	p__Bacteroidota	c__Bacteroidia	o__Bacteroidales	f__Bacteroidaceae	g__Prevotella	s__Prevotella unknown_23

MGYG-HGUT-01977:s__Bacteroides unknown_4	0.337711812	-0.268440916	0.930624692	d__Bacteria	p__Bacteroidota	c__Bacteroidia	o__Bacteroidales	f__Bacteroidaceae	g__Bacteroides	s__Bacteroides unknown_4
MGYG-HGUT-01982:s__Collinsella unknown_181	0.502142237	0.185694015	0.968532073	d__Bacteria	p__Actinobacteriota	c__Coriobacteriia	o__Coriobacteriales	f__Coriobacteriaceae	g__Collinsella	s__Collinsella unknown_181
MGYG-HGUT-01983:s__Oscillibacter sp000436875	0.215234428	-0.320820428	0.891825559	d__Bacteria	p__Firmicutes_A	c__Clostridia	o__Oscillospirales	f__Oscillospiraceae	g__Oscillibacter	s__Oscillibacter sp000436875
MGYG-HGUT-01986:s__Collinsella aerofaciens_F_6	0.170077906	0.373459988	0.891825559	d__Bacteria	p__Actinobacteriota	c__Coriobacteriia	o__Coriobacteriales	f__Coriobacteriaceae	g__Collinsella	s__Collinsella aerofaciens_F_6
MGYG-HGUT-01987:s__Ruminococcaceae unknown unknown_9	0.583585957	-0.152224431	0.975671757	d__Bacteria	p__Firmicutes_A	c__Clostridia	o__Oscillospirales	f__Ruminococcaceae	g__Ruminococcaceae unknown	s__Ruminococcaceae unknown unknown_9
MGYG-HGUT-01988:s__Marvinbryantia unknown_2	0.598360366	0.143337482	0.975671757	d__Bacteria	p__Firmicutes_A	c__Clostridia	o__Lachnospirales	f__Lachnospiraceae	g__Marvinbryantia	s__Marvinbryantia unknown_2
MGYG-HGUT-01993:s__Collinsella unknown_183	0.481170101	0.194937735	0.968532073	d__Bacteria	p__Actinobacteriota	c__Coriobacteriia	o__Coriobacteriales	f__Coriobacteriaceae	g__Collinsella	s__Collinsella unknown_183
MGYG-HGUT-01994:s__Faecalicatena unknown_4	0.256762021	0.314649572	0.905666614	d__Bacteria	p__Firmicutes_A	c__Clostridia	o__Lachnospirales	f__Lachnospiraceae	g__Faecalicatena	s__Faecalicatena unknown_4
MGYG-HGUT-01995:s__Parabacteroides unknown_9	0.511934369	-0.180128988	0.968532073	d__Bacteria	p__Bacteroidota	c__Bacteroidia	o__Bacteroidales	f__Tannerellaceae	g__Parabacteroides	s__Parabacteroides unknown_9

MGYG-HGUT-01996:s__Collinsella unknown_184	0.808709122	0.066716787	0.987142302	d__Bacteria	p__Actinobacteriota	c__Coriobacteriia	o__Coriobacteriales	f__Coriobacteriaceae	g__Collinsella	s__Collinsella unknown_184
MGYG-HGUT-01997:s__Collinsella unknown_185	0.104295971	0.432280077	0.883307256	d__Bacteria	p__Actinobacteriota	c__Coriobacteriia	o__Coriobacteriales	f__Coriobacteriaceae	g__Collinsella	s__Collinsella unknown_185
MGYG-HGUT-01998:s__Lachnospira unknown_2	0.201527833	0.353486086	0.891825559	d__Bacteria	p__Firmicutes_A	c__Clostridia	o__Lachnospirales	f__Lachnospiraceae	g__Lachnospira	s__Lachnospira unknown_2
MGYG-HGUT-01999:s__Collinsella unknown_186	0.409805368	0.220945853	0.93970925	d__Bacteria	p__Actinobacteriota	c__Coriobacteriia	o__Coriobacteriales	f__Coriobacteriaceae	g__Collinsella	s__Collinsella unknown_186
MGYG-HGUT-02001:s__CAG-177 sp000431775	0.254591554	0.317256567	0.905015696	d__Bacteria	p__Firmicutes_A	c__Clostridia	o__Oscillospirales	f__Acutalibacteraceae	g__CAG-177	s__CAG-177 sp000431775
MGYG-HGUT-02006:s__Lawsonibacter unknown_5	0.571512826	-0.149741185	0.974686221	d__Bacteria	p__Firmicutes_A	c__Clostridia	o__Oscillospirales	f__Oscillospiraceae	g__Lawsonibacter	s__Lawsonibacter unknown_5
MGYG-HGUT-02007:s__Alistipes unknown_5	0.941623931	0.02014569	0.994479474	d__Bacteria	p__Bacteroidota	c__Bacteroidia	o__Bacteroidales	f__Rikenellaceae	g__Alistipes	s__Alistipes unknown_5
MGYG-HGUT-02012:s__CAG-145 unknown_7	0.281084441	0.29839861	0.929025913	d__Bacteria	p__Firmicutes_A	c__Clostridia	o__Peptostreptococcales	f__Anaerovoraceae	g__CAG-145	s__CAG-145 unknown_7
MGYG-HGUT-02020:s__Collinsella unknown_188	0.651714082	-0.126344922	0.975671757	d__Bacteria	p__Actinobacteriota	c__Coriobacteriia	o__Coriobacteriales	f__Coriobacteriaceae	g__Collinsella	s__Collinsella unknown_188

MGYG-HGUT-02024:s__CAG-877 unknown_4	0.354564881	-0.256189103	0.93970925	d__Bacteria	p__Firmicutes	c__Bacilli	o__RF39	f__CAG-611	g__CAG-877	s__CAG-877 unknown_4
MGYG-HGUT-02026:s__Oscillospiraceae unknown unknown_6	0.482402409	-0.192200269	0.968532073	d__Bacteria	p__Firmicutes_A	c__Clostridia	o__Oscillospirales	f__Oscillospiraceae	g__Oscillospiraceae unknown	s__Oscillospiraceae unknown unknown_6
MGYG-HGUT-02028:s__Collinsella unknown_189	0.446804432	0.205639271	0.961292208	d__Bacteria	p__Actinobacteriota	c__Coriobacteriia	o__Coriobacteriales	f__Coriobacteriaceae	g__Collinsella	s__Collinsella unknown_189
MGYG-HGUT-02029:s__GCA-900066575 unknown_2	0.290648814	0.293239186	0.929025913	d__Bacteria	p__Firmicutes_A	c__Clostridia	o__Lachnospirales	f__Lachnospiraceae	g__GCA-900066575	s__GCA-900066575 unknown_2
MGYG-HGUT-02032:s__Collinsella unknown_190	0.332671044	0.266068901	0.930624692	d__Bacteria	p__Actinobacteriota	c__Coriobacteriia	o__Coriobacteriales	f__Coriobacteriaceae	g__Collinsella	s__Collinsella unknown_190
MGYG-HGUT-02033:s__Parabacteroides sp900155425	0.96303569	-0.012860917	0.994479474	d__Bacteria	p__Bacteroidota	c__Bacteroidia	o__Bacteroidales	f__Tannerellaceae	g__Parabacteroides	s__Parabacteroides sp900155425
MGYG-HGUT-02034:s__CAG-170 unknown_6	0.180236847	0.353363599	0.891825559	d__Bacteria	p__Firmicutes_A	c__Clostridia	o__Oscillospirales	f__Oscillospiraceae	g__CAG-170	s__CAG-170 unknown_6
MGYG-HGUT-02037:s__Ruminococcaceae unknown unknown_10	0.199584497	0.358091762	0.891825559	d__Bacteria	p__Firmicutes_A	c__Clostridia	o__Oscillospirales	f__Ruminococcaceae	g__Ruminococcaceae unknown	s__Ruminococcaceae unknown unknown_10
MGYG-HGUT-02040:s__Faecalibacterium unknown_5	0.497519559	0.188174785	0.968532073	d__Bacteria	p__Firmicutes_A	c__Clostridia	o__Oscillospirales	f__Ruminococcaceae	g__Faecalibacterium	s__Faecalibacterium unknown_5

MGYG-HGUT-02043:s__Collinsella unknown_192	0.108625219	0.439758317	0.883307256	d__Bacteria	p__Actinobacteriota	c__Coriobacteriia	o__Coriobacteriales	f__Coriobacteriaceae	g__Collinsella	s__Collinsella unknown_192
MGYG-HGUT-02045:s__CAG-110 unknown_11	0.146396176	0.390094054	0.891825559	d__Bacteria	p__Firmicutes_A	c__Clostridia	o__Oscillospirales	f__Oscillospiraceae	g__CAG-110	s__CAG-110 unknown_11
MGYG-HGUT-02048:s__Collinsella sp002232035_24	0.301200209	0.278147014	0.929025913	d__Bacteria	p__Actinobacteriota	c__Coriobacteriia	o__Coriobacteriales	f__Coriobacteriaceae	g__Collinsella	s__Collinsella sp002232035_24
MGYG-HGUT-02055:s__Collinsella unknown_195	0.114470523	0.429182603	0.883307256	d__Bacteria	p__Actinobacteriota	c__Coriobacteriia	o__Coriobacteriales	f__Coriobacteriaceae	g__Collinsella	s__Collinsella unknown_195
MGYG-HGUT-02057:s__Oscillibacter sp001916835	0.998843763	0.000400336	0.998843763	d__Bacteria	p__Firmicutes_A	c__Clostridia	o__Oscillospirales	f__Oscillospiraceae	g__Oscillibacter	s__Oscillibacter sp001916835
MGYG-HGUT-02058:s__Collinsella sp002232035_25	0.047861122	0.546353078	0.814550715	d__Bacteria	p__Actinobacteriota	c__Coriobacteriia	o__Coriobacteriales	f__Coriobacteriaceae	g__Collinsella	s__Collinsella sp002232035_25
MGYG-HGUT-02059:s__Gemmiger unknown_10	0.224074475	0.335007922	0.891825559	d__Bacteria	p__Firmicutes_A	c__Clostridia	o__Oscillospirales	f__Ruminococcaceae	g__Gemmiger	s__Gemmiger unknown_10
MGYG-HGUT-02060:s__CAG-110 sp003525905	0.202626522	0.343085247	0.891825559	d__Bacteria	p__Firmicutes_A	c__Clostridia	o__Oscillospirales	f__Oscillospiraceae	g__CAG-110	s__CAG-110 sp003525905
MGYG-HGUT-02065:s__Collinsella sp002232035_26	0.681147621	0.111880672	0.975671757	d__Bacteria	p__Actinobacteriota	c__Coriobacteriia	o__Coriobacteriales	f__Coriobacteriaceae	g__Collinsella	s__Collinsella sp002232035_26
MGYG-HGUT-02067:s__Oscillosp	0.60051	0.14422	0.97567	d__Bact	p__Firmicutes_A	c__Clostridia	o__Oscillospirales	f__Oscillospiraceae	g__Oscillospiraceae unknown	s__Oscillospiraceae

iraceae unknown unknown_7	8014	8756	1757	eria						unknown unknown_7
MGYG-HGUT-02069:s__Collinsella unknown_197	0.28741098	0.290963113	0.929025913	d__Bacteria	p__Actinobacteriota	c__Coriobacteriia	o__Coriobacteriales	f__Coriobacteriaceae	g__Collinsella	s__Collinsella unknown_197
MGYG-HGUT-02070:s__Eubacterium_I unknown_3	0.772646891	0.078918559	0.979520484	d__Bacteria	p__Firmicutes_A	c__Clostridia	o__Lachnospirales	f__Lachnospiraceae	g__Eubacterium_I	s__Eubacterium_I unknown_3
MGYG-HGUT-02072:s__CAG-74 unknown unknown_11	0.851066081	-0.052213803	0.992878088	d__Bacteria	p__Firmicutes_A	c__Clostridia	o__Christensenellales	f__CAG-74	g__CAG-74 unknown	s__CAG-74 unknown unknown_11
MGYG-HGUT-02073:s__CAG-110 unknown_12	0.384710076	0.232121055	0.93970925	d__Bacteria	p__Firmicutes_A	c__Clostridia	o__Oscillospirales	f__Oscillospiraceae	g__CAG-110	s__CAG-110 unknown_12
MGYG-HGUT-02075:s__Lachnospiraceae unknown unknown_18	0.349477826	0.260575406	0.93970925	d__Bacteria	p__Firmicutes_A	c__Clostridia	o__Lachnospirales	f__Lachnospiraceae	g__Lachnospiraceae unknown	s__Lachnospiraceae unknown unknown_18
MGYG-HGUT-02080:s__Prevotella unknown_26	0.997222227	-0.000928653	0.998843763	d__Bacteria	p__Bacteroidota	c__Bacteroidia	o__Bacteroidales	f__Bacteroidaceae	g__Prevotella	s__Prevotella unknown_26
MGYG-HGUT-02083:s__Collinsella sp002232035_27	0.727310779	-0.096790584	0.979458475	d__Bacteria	p__Actinobacteriota	c__Coriobacteriia	o__Coriobacteriales	f__Coriobacteriaceae	g__Collinsella	s__Collinsella sp002232035_27
MGYG-HGUT-02085:s__CAG-170 unknown_7	0.888450712	0.035814561	0.994479474	d__Bacteria	p__Firmicutes_A	c__Clostridia	o__Oscillospirales	f__Oscillospiraceae	g__CAG-170	s__CAG-170 unknown_7
MGYG-HGUT-02087:s__Collinsella unknown_199	0.24069	0.31767	0.90218	d__Bact	p__Actino	c__Coriobacteriia	o__Coriobacteriales	f__Coriobacteriaceae	g__Collinsella	s__Collinsella unknown_199

	616	7479	1367	eria	bacteriota					
MGYG-HGUT-02090:s__CAG-83 unknown_6	0.142724235	0.396330892	0.891825559	d__Bacteria	p__Firmicutes_A	c__Clostridia	o__Oscillospirales	f__Oscillospiraceae	g__CAG-83	s__CAG-83 unknown_6
MGYG-HGUT-02096:s__Christensenellaceae unknown unknown_3	0.523837518	0.168882282	0.968532073	d__Bacteria	p__Firmicutes_A	c__Clostridia	o__Christensenellales	f__Christensenellaceae	g__Christensenellaceae unknown	s__Christensenellaceae unknown unknown_3
MGYG-HGUT-02097:s__CAG-74 unknown unknown_12	0.745164464	-0.0866895527	0.979458475	d__Bacteria	p__Firmicutes_A	c__Clostridia	o__Christensenellales	f__CAG-74	g__CAG-74 unknown	s__CAG-74 unknown unknown_12
MGYG-HGUT-02098:s__CAG-74 unknown unknown_13	0.010094986	0.656460255	0.768225834	d__Bacteria	p__Firmicutes_A	c__Clostridia	o__Christensenellales	f__CAG-74	g__CAG-74 unknown	s__CAG-74 unknown unknown_13
MGYG-HGUT-02099:s__UBA7102 unknown_1	0.171907252	0.359482592	0.891825559	d__Bacteria	p__Firmicutes_A	c__Clostridia	o__Christensenellales	f__UBA1750	g__UBA7102	s__UBA7102 unknown_1
MGYG-HGUT-02102:s__Angelakisella unknown_1	0.317733256	0.270451052	0.929025913	d__Bacteria	p__Firmicutes_A	c__Clostridia	o__Oscillospirales	f__Ruminococcaceae	g__Angelakisella	s__Angelakisella unknown_1
MGYG-HGUT-02105:s__Ruminococcaceae unknown unknown_13	0.875423687	-0.0431858 97	0.994479474	d__Bacteria	p__Firmicutes_A	c__Clostridia	o__Oscillospirales	f__Ruminococcaceae	g__Ruminococcaceae unknown	s__Ruminococcaceae unknown unknown_13
MGYG-HGUT-02107:s__Collinsella unknown_201	0.023666104	0.622989179	0.768225834	d__Bacteria	p__Actinobacteriota	c__Coriobacteriia	o__Coriobacteriales	f__Coriobacteriaceae	g__Collinsella	s__Collinsella unknown_201
MGYG-HGUT-02108:s__Prevotella sp900290275	0.54329	0.16556	0.96853	d__Bact	p__Bacteroidota	c__Bacteroidia	o__Bacteroidales	f__Bacteroidaceae	g__Prevotella	s__Prevotella sp900290275

	6763	1796	2073	eria						
MGYG-HGUT-02109:s__UBA1232 unknown_1	0.0100051204	-0.689887811	0.768225834	d__Bacteria	p__Bacteroidota	c__Bacteroidia	o__Bacteroidales	f__UBA932	g__UBA1232	s__UBA1232 unknown_1
MGYG-HGUT-02110:s__Collinsella unknown_202	0.024574988	0.618114924	0.768225834	d__Bacteria	p__Actinobacteriota	c__Coriobacteriia	o__Coriobacteriales	f__Coriobacteriaceae	g__Collinsella	s__Collinsella unknown_202
MGYG-HGUT-02111:s__Collinsella unknown_203	0.512358921	-0.183953851	0.968532073	d__Bacteria	p__Actinobacteriota	c__Coriobacteriia	o__Coriobacteriales	f__Coriobacteriaceae	g__Collinsella	s__Collinsella unknown_203
MGYG-HGUT-02114:s__Oscillibacter unknown_12	0.774103953	0.07953475	0.979520484	d__Bacteria	p__Firmicutes_A	c__Clostridia	o__Oscillospirales	f__Oscillospiraceae	g__Oscillibacter	s__Oscillibacter unknown_12
MGYG-HGUT-02115:s__CAG-110 unknown_13	0.110383271	0.424199457	0.883307256	d__Bacteria	p__Firmicutes_A	c__Clostridia	o__Oscillospirales	f__Oscillospiraceae	g__CAG-110	s__CAG-110 unknown_13
MGYG-HGUT-02116:s__ER4 unknown_3	0.268370159	0.296563665	0.920494192	d__Bacteria	p__Firmicutes_A	c__Clostridia	o__Oscillospirales	f__Oscillospiraceae	g__ER4	s__ER4 unknown_3
MGYG-HGUT-02117:s__Collinsella unknown_205	0.869171782	-0.045806185	0.994479474	d__Bacteria	p__Actinobacteriota	c__Coriobacteriia	o__Coriobacteriales	f__Coriobacteriaceae	g__Collinsella	s__Collinsella unknown_205
MGYG-HGUT-02122:s__Collinsella unknown_206	0.727381381	0.096717414	0.979458475	d__Bacteria	p__Actinobacteriota	c__Coriobacteriia	o__Coriobacteriales	f__Coriobacteriaceae	g__Collinsella	s__Collinsella unknown_206
MGYG-HGUT-02124:s__CAG-110 unknown_14	0.69675	-0.108	0.97945	d__Bact	p__Firmicutes_A	c__Clostridia	o__Oscillospirales	f__Oscillospiraceae	g__CAG-110	s__CAG-110 unknown_14

	7401	131911	8475	eria						
MGYG-HGUT-02126:s__UBA1390 unknown_2	0.057940018	0.523311637	0.857211132	d__Bacteria	p__Firmicutes_A	c__Clostridia	o__Lachnospirales	f__UBA1390	g__UBA1390	s__UBA1390 unknown_2
MGYG-HGUT-02127:s__Agathobaculum unknown_3	0.757733838	0.085925516	0.979458475	d__Bacteria	p__Firmicutes_A	c__Clostridia	o__Oscillospirales	f__Butyricicoccaceae	g__Agathobaculum	s__Agathobaculum unknown_3
MGYG-HGUT-02129:s__Negativibacillus unknown_1	0.372377686	0.244514263	0.93970925	d__Bacteria	p__Firmicutes_A	c__Clostridia	o__Oscillospirales	f__Ruminococcaceae	g__Negativibacillus	s__Negativibacillus unknown_1
MGYG-HGUT-02133:s__Prevotella sp000436915	0.632394958	-0.131046044	0.975671757	d__Bacteria	p__Bacteroidota	c__Bacteroidia	o__Bacteroidales	f__Bacteroidaceae	g__Prevotella	s__Prevotella sp000436915
MGYG-HGUT-02135:s__Anaeromassilibacillus sp002159845	0.315585113	-0.268735449	0.929025913	d__Bacteria	p__Firmicutes_A	c__Clostridia	o__Oscillospirales	f__Acutalibacteraceae	g__Anaeromassilibacillus	s__Anaeromassilibacillus sp002159845
MGYG-HGUT-02136:s__UC5-1-2E3 sp001304875	0.595112572	-0.147437939	0.975671757	d__Bacteria	p__Firmicutes_A	c__Clostridia	o__Lachnospirales	f__Lachnospiraceae	g__UC5-1-2E3	s__UC5-1-2E3 sp001304875
MGYG-HGUT-02138:s__Agathobaculum unknown_4	0.204277385	0.355245121	0.891825559	d__Bacteria	p__Firmicutes_A	c__Clostridia	o__Oscillospirales	f__Butyricicoccaceae	g__Agathobaculum	s__Agathobaculum unknown_4
MGYG-HGUT-02140:s__CAG-510 unknown_2	0.532407557	-0.174380089	0.968532073	d__Bacteria	p__Firmicutes_A	c__Clostridia	o__Lachnospirales	f__Lachnospiraceae	g__CAG-510	s__CAG-510 unknown_2

MGYG-HGUT-02141:s__CAG-95 unknown_2	0.8484372 46	-0.0537396 15	0.9928780 88	d__Bacteria	p__Firmicutes_A	c__Clostridia	o__Lachnospirales	f__Lachnospiraceae	g__CAG-95	s__CAG-95 unknown_2
MGYG-HGUT-02142:s__Collinsella unknown_209	0.0680174 77	0.5053845 33	0.8746187 46	d__Bacteria	p__Actinobacteriota	c__Coriobacteriia	o__Coriobacteriales	f__Coriobacteriaceae	g__Collinsella	s__Collinsella unknown_209
MGYG-HGUT-02143:s__CAG-83 unknown_8	0.1933313 35	0.3578122 1	0.8918255 59	d__Bacteria	p__Firmicutes_A	c__Clostridia	o__Oscillospirales	f__Oscillospiraceae	g__CAG-83	s__CAG-83 unknown_8
MGYG-HGUT-02144:s__CAG-83 unknown_9	0.9386528 86	0.0208510 06	0.9944794 74	d__Bacteria	p__Firmicutes_A	c__Clostridia	o__Oscillospirales	f__Oscillospiraceae	g__CAG-83	s__CAG-83 unknown_9
MGYG-HGUT-02145:s__Dorea phocaeense	0.1149116 45	-0.4373302 16	0.8833072 56	d__Bacteria	p__Firmicutes_A	c__Clostridia	o__Lachnospirales	f__Lachnospiraceae	g__Dorea	s__Dorea phocaeense
MGYG-HGUT-02146:s__CAG-145 sp003150015	0.0571935 86	0.5214282 66	0.8572113 2	d__Bacteria	p__Firmicutes_A	c__Clostridia	o__Peptostreptococcales	f__Anaerovoraceae	g__CAG-145	s__CAG-145 sp003150015
MGYG-HGUT-02149:s__Blautia sp003287895	0.7513946 78	0.0879072 32	0.9794584 75	d__Bacteria	p__Firmicutes_A	c__Clostridia	o__Lachnospirales	f__Lachnospiraceae	g__Blautia	s__Blautia sp003287895
MGYG-HGUT-02150:s__Faecalicatena unknown_5	0.1993727 22	0.3581500 75	0.8918255 59	d__Bacteria	p__Firmicutes_A	c__Clostridia	o__Lachnospirales	f__Lachnospiraceae	g__Faecalicatena	s__Faecalicatena unknown_5
MGYG-HGUT-02152:s__UBA5446 unknown_6	0.2678680 43	-0.2866548 43	0.9204941 92	d__Bacteria	p__Firmicutes_A	c__Clostridia	o__Oscillospirales	f__Oscillospiraceae	g__UBA5446	s__UBA5446 unknown_6

MGYG-HGUT-02153:s__CAG-484 sp000431315_2	0.377270524	0.237135213	0.939709925	d__Bacteria	p__Cyanobacteria	c__Vampirovibrionia	o__Gastranaerophilales	f__Gastranaerophilaceae	g__CAG-484	s__CAG-484 sp000431315_2
MGYG-HGUT-02155:s__Ruthenibacterium unknown_1	0.378980976	0.246744814	0.939709925	d__Bacteria	p__Firmicutes_A	c__Clostridia	o__Oscillospirales	f__Ruminococcaceae	g__Ruthenibacterium	s__Ruthenibacterium unknown_1
MGYG-HGUT-02156:s__CAG-83 unknown_10	0.081416631	0.478805088	0.877826247	d__Bacteria	p__Firmicutes_A	c__Clostridia	o__Oscillospirales	f__Oscillospiraceae	g__CAG-83	s__CAG-83 unknown_10
MGYG-HGUT-02163:s__Methanobrevibacter_A smithii	0.334892379	-0.260776946	0.930624692	d__Archaea	p__Euryarchaeota	c__Methanobacteria	o__Methanobacteriales	f__Methanobacteriaceae	g__Methanobrevibacter_A	s__Methanobrevibacter_A smithii
MGYG-HGUT-02166:s__CAG-433 sp000433675	0.936470996	-0.022199368	0.994479474	d__Bacteria	p__Firmicutes	c__Bacilli	o__RF39	f__CAG-433	g__CAG-433	s__CAG-433 sp000433675
MGYG-HGUT-02168:s__Prevotella unknown_28	0.896886261	0.034501796	0.994479474	d__Bacteria	p__Bacteroidota	c__Bacteroidia	o__Bacteroidales	f__Bacteroidaceae	g__Prevotella	s__Prevotella unknown_28
MGYG-HGUT-02171:s__Bacteroides_B sp002493165	0.235549641	0.331197975	0.902181367	d__Bacteria	p__Bacteroidota	c__Bacteroidia	o__Bacteroidales	f__Bacteroidaceae	g__Bacteroides_B	s__Bacteroides_B sp002493165
MGYG-HGUT-02176:s__Muribaculaceae unknown unknown_3	0.771158565	-0.079928894	0.979520484	d__Bacteria	p__Bacteroidota	c__Bacteroidia	o__Bacteroidales	f__Muribaculaceae	g__Muribaculaceae unknown	s__Muribaculaceae unknown unknown_3
MGYG-HGUT-02177:s__CAG-508 unknown unknown_10	0.471957998	-0.196666	0.968299589	d__Bacteria	p__Firmicutes_A	c__Clostridia	o__TANB77	f__CAG-508	g__CAG-508 unknown	s__CAG-508 unknown unknown_10

		2884								
MGYG-HGUT-02181:s__Paramuribaculum sp000431155	0.951276628	-0.016966485	0.994479474	d__Bacteria	p__Bacteroidota	c__Bacteroidia	o__Bacteroidales	f__Muribaculaceae	g__Paramuribaculum	s__Paramuribaculum sp000431155
MGYG-HGUT-02184:s__Collinsella unknown_211	0.270916922	0.303415146	0.925406661	d__Bacteria	p__Actinobacteriota	c__Coriobacteriia	o__Coriobacteriales	f__Coriobacteriaceae	g__Collinsella	s__Collinsella unknown_211
MGYG-HGUT-02186:s__Ruminococcus_A sp000432335	0.951596175	-0.017038088	0.994479474	d__Bacteria	p__Firmicutes_A	c__Clostridia	o__Lachnospirales	f__Lachnospiraceae	g__Ruminococcus_A	s__Ruminococcus_A sp000432335
MGYG-HGUT-02187:s__Collinsella sp000434535	0.036560593	0.568448345	0.768225834	d__Bacteria	p__Actinobacteriota	c__Coriobacteriia	o__Coriobacteriales	f__Coriobacteriaceae	g__Collinsella	s__Collinsella sp000434535
MGYG-HGUT-02190:s__Clostridium sp001916075	0.658602573	-0.123734944	0.975671757	d__Bacteria	p__Firmicutes_A	c__Clostridia	o__Clostridiales	f__Clostridiaceae	g__Clostridium	s__Clostridium sp001916075
MGYG-HGUT-02191:s__CAG-313 sp000433035	0.336927275	-0.264912959	0.930624692	d__Bacteria	p__Firmicutes	c__Bacilli	o__ML615J-28	f__CAG-313	g__CAG-313	s__CAG-313 sp000433035
MGYG-HGUT-02194:s__Acutalibacteraceae unknown unknown_24	0.949790661	-0.017024078	0.994479474	d__Bacteria	p__Firmicutes_A	c__Clostridia	o__Oscillospirales	f__Acutalibacteraceae	g__Acutalibacteraceae unknown	s__Acutalibacteraceae unknown unknown_24
MGYG-HGUT-02195:s__Emergencia unknown_2	0.314844428	-0.277181263	0.929025913	d__Bacteria	p__Firmicutes_A	c__Clostridia	o__Peptostreptococcales	f__Anaerovoraceae	g__Emergencia	s__Emergencia unknown_2

MGYG-HGUT-02202:s__Dorea sp900312975	0.515903761	-0.17871757581	0.9685320 73	d__Bacteria	p__Firmicutes_A	c__Clostridia	o__Lachnospirales	f__Lachnospiraceae	g__Dorea	s__Dorea sp900312975
MGYG-HGUT-02209:s__CAG-462 unknown_1	0.01008875	0.704866397	0.768225834	d__Bacteria	p__Bacteroidota	c__Bacteroidia	o__Bacteroidales	f__Bacteroidaceae	g__CAG-462	s__CAG-462 unknown_1
MGYG-HGUT-02210:s__Lachnospiraceae unknown unknown_19	0.666641254	0.116843284	0.975671757	d__Bacteria	p__Firmicutes_A	c__Clostridia	o__Lachnospirales	f__Lachnospiraceae	g__Lachnospiraceae unknown	s__Lachnospiraceae unknown unknown_19
MGYG-HGUT-02212:s__Blautia unknown_6	0.95336584	0.016163464	0.994479474	d__Bacteria	p__Firmicutes_A	c__Clostridia	o__Lachnospirales	f__Lachnospiraceae	g__Blautia	s__Blautia unknown_6
MGYG-HGUT-02213:s__Oscillospiraceae unknown unknown_10	0.668575451	-0.11933597 8	0.975671757	d__Bacteria	p__Firmicutes_A	c__Clostridia	o__Oscillospirales	f__Oscillospiraceae	g__Oscillospiraceae unknown	s__Oscillospiraceae unknown unknown_10
MGYG-HGUT-02214:s__Blautia_A unknown_14	0.478821742	0.197466334	0.968532073	d__Bacteria	p__Firmicutes_A	c__Clostridia	o__Lachnospirales	f__Lachnospiraceae	g__Blautia_A	s__Blautia_A unknown_14
MGYG-HGUT-02215:s__Blautia unknown_7	0.131269693	0.411958373	0.891825559	d__Bacteria	p__Firmicutes_A	c__Clostridia	o__Lachnospirales	f__Lachnospiraceae	g__Blautia	s__Blautia unknown_7
MGYG-HGUT-02216:s__Lachnospiraceae unknown unknown_20	0.320297654	-0.26923084 5	0.929175176	d__Bacteria	p__Firmicutes_A	c__Clostridia	o__Lachnospirales	f__Lachnospiraceae	g__Lachnospiraceae unknown	s__Lachnospiraceae unknown unknown_20
MGYG-HGUT-02217:s__Emergencia unknown_3	0.163948911	0.365858275	0.891825559	d__Bacteria	p__Firmicutes_A	c__Clostridia	o__Peptostreptococcales	f__Anaerovoraceae	g__Emergencia	s__Emergencia unknown_3

MGYG-HGUT-02218:s__Parabacteroides sp000436495	0.92529213	0.025697572	0.994479474	d__Bacteria	p__Bacteroidota	c__Bacteroidia	o__Bacteroidales	f__Tannerellaceae	g__Parabacteroides	s__Parabacteroides sp000436495
MGYG-HGUT-02219:s__UBA1731 unknown_1	0.118801965	0.429494981	0.883307256	d__Bacteria	p__Verrucomicrobiota	c__Kiritimatiellae	o__RFP12	f__UBA1067	g__UBA1731	s__UBA1731 unknown_1
MGYG-HGUT-02222:s__Dialister unknown_7	0.210620584	0.345709946	0.891825559	d__Bacteria	p__Firmicutes_C	c__Negativicutes	o__Veillonellales	f__Dialisteraceae	g__Dialister	s__Dialister unknown_7
MGYG-HGUT-02223:s__Faecalibacterium unknown_6	0.019354034	0.626369881	0.768225834	d__Bacteria	p__Firmicutes_A	c__Clostridia	o__Oscillospirales	f__Ruminococcaceae	g__Faecalibacterium	s__Faecalibacterium unknown_6
MGYG-HGUT-02224:s__Faecalibacterium unknown_7	0.065155003	0.511683238	0.85721132	d__Bacteria	p__Firmicutes_A	c__Clostridia	o__Oscillospirales	f__Ruminococcaceae	g__Faecalibacterium	s__Faecalibacterium unknown_7
MGYG-HGUT-02226:s__CAG-81 unknown_1	0.968749023	0.010902688	0.994479474	d__Bacteria	p__Firmicutes_A	c__Clostridia	o__Lachnospirales	f__Lachnospiraceae	g__CAG-81	s__CAG-81 unknown_1
MGYG-HGUT-02229:s__Oscillibacter unknown_13	0.079774878	0.472929818	0.877826247	d__Bacteria	p__Firmicutes_A	c__Clostridia	o__Oscillospirales	f__Oscillospiraceae	g__Oscillibacter	s__Oscillibacter unknown_13
MGYG-HGUT-02230:s__Ruminococcus unknown_4	0.816561591	0.06484569	0.987664539	d__Bacteria	p__Firmicutes_A	c__Clostridia	o__Oscillospirales	f__Ruminococcaceae	g__Ruminococcus	s__Ruminococcus unknown_4
MGYG-HGUT-02233:s__Acutalibacter unknown_6	0.901769368	-0.033457765	0.994479474	d__Bacteria	p__Firmicutes_A	c__Clostridia	o__Oscillospirales	f__Acutalibacteraceae	g__Acutalibacter	s__Acutalibacter unknown_6

MGYG-HGUT-02234:s__Coprococcus_B unknown_1	0.106163886	0.423061943	0.883307256	d__Bacteria	p__Firmicutes_A	c__Clostridia	o__Lachnospirales	f__Lachnospiraceae	g__Coprococcus_B	s__Coprococcus_B unknown_1
MGYG-HGUT-02238:s__CAG-110 unknown_15	0.675208457	0.113872324	0.975671757	d__Bacteria	p__Firmicutes_A	c__Clostridia	o__Oscillospirales	f__Oscillospiraceae	g__CAG-110	s__CAG-110 unknown_15
MGYG-HGUT-02247:s__Acetatifactor unknown_6	0.78351704	0.07641305	0.981180764	d__Bacteria	p__Firmicutes_A	c__Clostridia	o__Lachnospirales	f__Lachnospiraceae	g__Acetatifactor	s__Acetatifactor unknown_6
MGYG-HGUT-02254:s__CAG-110 unknown_17	0.663230624	0.120453085	0.975671757	d__Bacteria	p__Firmicutes_A	c__Clostridia	o__Oscillospirales	f__Oscillospiraceae	g__CAG-110	s__CAG-110 unknown_17
MGYG-HGUT-02256:s__Lachnospiraceae unknown unknown_21	0.278967873	0.302851077	0.929025913	d__Bacteria	p__Firmicutes_A	c__Clostridia	o__Lachnospirales	f__Lachnospiraceae	g__Lachnospiraceae unknown	s__Lachnospiraceae unknown unknown_21
MGYG-HGUT-02263:s__Ruminococcus_E unknown_2	0.319205067	0.269552093	0.929025913	d__Bacteria	p__Firmicutes_A	c__Clostridia	o__Oscillospirales	f__Acutalibacteraceae	g__Ruminococcus_E	s__Ruminococcus_E unknown_2
MGYG-HGUT-02267:s__Eisenbergiella unknown_5	0.838485572	-0.0572973	0.992878088	d__Bacteria	p__Firmicutes_A	c__Clostridia	o__Lachnospirales	f__Lachnospiraceae	g__Eisenbergiella	s__Eisenbergiella unknown_5
MGYG-HGUT-02268:s__Absiella unknown_4	0.887584466	0.039378407	0.994479474	d__Bacteria	p__Firmicutes	c__Bacilli	o__Erysipelotrichales	f__Erysipelotrichaceae	g__Absiella	s__Absiella unknown_4
MGYG-HGUT-02271:s__GCA-900066135 sp900066135	0.249597161	0.32145833	0.902181367	d__Bacteria	p__Firmicutes_A	c__Clostridia	o__Lachnospirales	f__Lachnospiraceae	g__GCA-900066135	s__GCA-900066135 sp900066135
MGYG-HGUT-02272:s__Faecalib	0.47133	-0.201 01	0.96819	d__Bact	p__Firmicutes_A	c__Clostridia	o__Oscillospirales	f__Ruminococcaceae	g__Faecalibacterium	s__Faecalibacterium prausnitzii_D

acterium prausnitzii_D	0176	652255	4079	eria						
MGYG-HGUT-02273:s__Bacteroides stercorirosoris	0.81751 9594	0.06443 6575	0.98766 4539	d__Bacteria	p__Bacteroidota	c__Bacteroidia	o__Bacteroidales	f__Bacteroidaceae	g__Bacteroides	s__Bacteroides stercorirosoris
MGYG-HGUT-02274:s__Faecalibacterium prausnitzii_I	0.14746 1286	0.40220 1153	0.89182 5559	d__Bacteria	p__Firmicutes_A	c__Clostridia	o__Oscillospirales	f__Ruminococcaceae	g__Faecalibacterium	s__Faecalibacterium prausnitzii_I
MGYG-HGUT-02275:s__Paraprevotella unknown_5	0.94699 5232	0.01859 9279	0.99447 9474	d__Bacteria	p__Bacteroidota	c__Bacteroidia	o__Bacteroidales	f__Bacteroidaceae	g__Paraprevotella	s__Paraprevotella unknown_5
MGYG-HGUT-02276:s__Lachnospiraceae unknown unknown_22	0.10640 1567	0.45043 8596	0.88330 7256	d__Bacteria	p__Firmicutes_A	c__Clostridia	o__Lachnospirales	f__Lachnospiraceae	g__Lachnospiraceae unknown	s__Lachnospiraceae unknown unknown_22
MGYG-HGUT-02277:s__Marvinbryantia sp900066075	0.24014 2931	0.32889 5631	0.90218 1367	d__Bacteria	p__Firmicutes_A	c__Clostridia	o__Lachnospirales	f__Lachnospiraceae	g__Marvinbryantia	s__Marvinbryantia sp900066075
MGYG-HGUT-02278:s__Eubacterium_I ramulus_A	0.34398 852	0.26247 6365	0.93970 925	d__Bacteria	p__Firmicutes_A	c__Clostridia	o__Lachnospirales	f__Lachnospiraceae	g__Eubacterium_I	s__Eubacterium_I ramulus_A
MGYG-HGUT-02279:s__Dorea longicatena_B	0.37910 7123	0.24111 7361	0.93970 925	d__Bacteria	p__Firmicutes_A	c__Clostridia	o__Lachnospirales	f__Lachnospiraceae	g__Dorea	s__Dorea longicatena_B
MGYG-HGUT-02280:s__UBA9502 unknown_6	0.50636 6844	0.18648 0617	0.96853 2073	d__Bacteria	p__Firmicutes_A	c__Clostridia	o__Lachnospirales	f__Lachnospiraceae	g__UBA9502	s__UBA9502 unknown_6
MGYG-HGUT-02281:s__Bacteroides faecis	0.77609 9803	0.07991 9145	0.97952 0484	d__Bacteria	p__Bacteroidota	c__Bacteroidia	o__Bacteroidales	f__Bacteroidaceae	g__Bacteroides	s__Bacteroides faecis

MGYG-HGUT-02282:s__Parabacteroides gordonii	0.676950545	-0.115006659	0.975671757	d__Bacteria	p__Bacteroidota	c__Bacteroidia	o__Bacteroidales	f__Tannerellaceae	g__Parabacteroides	s__Parabacteroides gordonii
MGYG-HGUT-02284:s__Megamonas funiformis	0.832348811	-0.058005107	0.99222019	d__Bacteria	p__Firmicutes_C	c__Negativicutes	o__Selenomonadales	f__Selenomonadaceae	g__Megamonas	s__Megamonas funiformis
MGYG-HGUT-02285:s__CAG-95 sp900066375	0.252196191	0.319044372	0.902181367	d__Bacteria	p__Firmicutes_A	c__Clostridia	o__Lachnospirales	f__Lachnospiraceae	g__CAG-95	s__CAG-95 sp900066375
MGYG-HGUT-02286:s__Blautia sp000436935	0.061609124	0.516443657	0.85721132	d__Bacteria	p__Firmicutes_A	c__Clostridia	o__Lachnospirales	f__Lachnospiraceae	g__Blautia	s__Blautia sp000436935
MGYG-HGUT-02287:s__Agathobaculum unknown_5	0.33798289	0.268533595	0.930624692	d__Bacteria	p__Firmicutes_A	c__Clostridia	o__Oscillospirales	f__Butyricicoccaceae	g__Agathobaculum	s__Agathobaculum unknown_5
MGYG-HGUT-02288:s__Bacillus_A sp001884105	0.584698979	0.146007677	0.975671757	d__Bacteria	p__Firmicutes	c__Bacilli	o__Bacillales	f__Bacillaceae_G	g__Bacillus_A	s__Bacillus_A sp001884105
MGYG-HGUT-02289:s__Coprococcus sp000433075	0.161262873	0.387873569	0.891825559	d__Bacteria	p__Firmicutes_A	c__Clostridia	o__Lachnospirales	f__Lachnospiraceae	g__Coprococcus	s__Coprococcus sp000433075
MGYG-HGUT-02290:s__Sellimonas sp002161525	0.390998736	-0.234346738	0.93970925	d__Bacteria	p__Firmicutes_A	c__Clostridia	o__Lachnospirales	f__Lachnospiraceae	g__Sellimonas	s__Sellimonas sp002161525
MGYG-HGUT-02291:s__Bacteroidaceae unknown unknown_2	0.623445479	-0.13078	0.975671757	d__Bacteria	p__Bacteroidota	c__Bacteroidia	o__Bacteroidales	f__Bacteroidaceae	g__Bacteroidaceae unknown	s__Bacteroidaceae unknown unknown_2

		7789								
MGYG-HGUT-02292:s__TF01-11 sp003529475	0.1390228 34	0.4097911 83	0.8918255 59	d__Bacteria	p__Firmicutes_A	c__Clostridia	o__Lachnospirales	f__Lachnospiraceae	g__TF01-11	s__TF01-11 sp003529475
MGYG-HGUT-02293:s__Prevotella copri	0.8941652 05	0.0365805 17	0.9944794 74	d__Bacteria	p__Bacteroidota	c__Bacteroidia	o__Bacteroidales	f__Bacteroidaceae	g__Prevotella	s__Prevotella copri
MGYG-HGUT-02294:s__Collinsella sp002232035_28	0.6626818 26	0.1189227 97	0.9756717 57	d__Bacteria	p__Actinobacteriota	c__Coriobacteriia	o__Coriobacteriales	f__Coriobacteriaceae	g__Collinsella	s__Collinsella sp002232035_28
MGYG-HGUT-02295:s__Ruminococcus_C callidus	0.2854332 4	-0.2885810 83	0.9290259 13	d__Bacteria	p__Firmicutes_A	c__Clostridia	o__Oscillospirales	f__Ruminococcaceae	g__Ruminococcus_C	s__Ruminococcus_C callidus
MGYG-HGUT-02296:s__Streptococcus sp000479315	0.1701354 01	0.3789910 15	0.8918255 59	d__Bacteria	p__Firmicutes	c__Bacilli	o__Lactobacillales	f__Streptococcaceae	g__Streptococcus	s__Streptococcus sp000479315
MGYG-HGUT-02297:s__TF01-11 sp001916135	0.6509797 33	0.1253083 2	0.9756717 57	d__Bacteria	p__Firmicutes_A	c__Clostridia	o__Lachnospirales	f__Lachnospiraceae	g__TF01-11	s__TF01-11 sp001916135
MGYG-HGUT-02298:s__Blautia_A sp000436615	0.2161267 13	0.3454939 73	0.8918255 59	d__Bacteria	p__Firmicutes_A	c__Clostridia	o__Lachnospirales	f__Lachnospiraceae	g__Blautia_A	s__Blautia_A sp000436615
MGYG-HGUT-02300:s__Bacteroides cutis	0.0421858 75	-0.5622482 84	0.7793210 52	d__Bacteria	p__Bacteroidota	c__Bacteroidia	o__Bacteroidales	f__Bacteroidaceae	g__Bacteroides	s__Bacteroides cutis
MGYG-HGUT-02302:s__Streptococcus vestibularis_2	0.54726	0.16732	0.96853	d__Bact	p__Firmicutes	c__Bacilli	o__Lactobacillales	f__Streptococcaceae	g__Streptococcus	s__Streptococcus vestibularis_2

	9754	7461	2073	eria						
MGYG-HGUT-02303:s__Ruminococcus_E unknown_3	0.406379217	0.225670395	0.93970925	d__Bacteria	p__Firmicutes_A	c__Clostridia	o__Oscillospirales	f__Acutalibacteraceae	g__Ruminococcus_E	s__Ruminococcus_E unknown_3
MGYG-HGUT-02304:s__UBA7182 unknown_2	0.059381525	0.514996494	0.85721132	d__Bacteria	p__Firmicutes_A	c__Clostridia	o__Lachnospirales	f__Lachnospiraceae	g__UBA7182	s__UBA7182 unknown_2
MGYG-HGUT-02312:s__Blautia_A sp000285855	0.73270625	0.092854467	0.979458475	d__Bacteria	p__Firmicutes_A	c__Clostridia	o__Lachnospirales	f__Lachnospiraceae	g__Blautia_A	s__Blautia_A sp000285855
MGYG-HGUT-02320:s__Enterococcus_B faecium_B	0.009126851	-0.713272373	0.768225834	d__Bacteria	p__Firmicutes	c__Bacilli	o__Lactobacillales	f__Enterococcaceae	g__Enterococcus_B	s__Enterococcus_B faecium_B
MGYG-HGUT-02321:s__Ruminococcus_D bicirculans	0.873118431	-0.043222827	0.994479474	d__Bacteria	p__Firmicutes_A	c__Clostridia	o__Oscillospirales	f__Ruminococcaceae	g__Ruminococcus_D	s__Ruminococcus_D bicirculans
MGYG-HGUT-02322:s__Escherichia marmotae	0.804941462	-0.066646663	0.987142302	d__Bacteria	p__Proteobacteria	c__Gammaproteobacteria	o__Enterobacterales	f__Enterobacteriaceae	g__Escherichia	s__Escherichia marmotae
MGYG-HGUT-02323:s__Escherichia sp000208585	0.900540775	-0.03424106	0.994479474	d__Bacteria	p__Proteobacteria	c__Gammaproteobacteria	o__Enterobacterales	f__Enterobacteriaceae	g__Escherichia	s__Escherichia sp000208585
MGYG-HGUT-02324:s__Lactobacillus_B salivarius	0.678754353	0.115775278	0.975671757	d__Bacteria	p__Firmicutes	c__Bacilli	o__Lactobacillales	f__Lactobacillaceae	g__Lactobacillus_B	s__Lactobacillus_B salivarius

MGYG-HGUT-02327:s__Oscillibacter sp900066435	0.46155596 47	-0.19224873	0.96557469 2	d__Bacteria	p__Firmicutes_A	c__Clostridia	o__Oscillospirales	f__Oscillospiraceae	g__Oscillibacter	s__Oscillibacter sp900066435
MGYG-HGUT-02328:s__Clostridium_M asparagiforme	0.62776600 9	-0.13496446 7	0.97567175 7	d__Bacteria	p__Firmicutes_A	c__Clostridia	o__Lachnospirales	f__Lachnospiraceae	g__Clostridium_M	s__Clostridium_M asparagiforme
MGYG-HGUT-02329:s__Dielma sp001305055	0.70739457	0.10172293 2	0.97945847 5	d__Bacteria	p__Firmicutes	c__Bacilli	o__Erysipelotrichales	f__Erysipelotrichaceae	g__Dielma	s__Dielma sp001305055
MGYG-HGUT-02330:s__Eisenbergiella tayi	0.74185163	0.08997802	0.97945847 5	d__Bacteria	p__Firmicutes_A	c__Clostridia	o__Lachnospirales	f__Lachnospiraceae	g__Eisenbergiella	s__Eisenbergiella tayi
MGYG-HGUT-02336:s__Megasphaera sp000417505	0.60541489 5	-0.14259015 9	0.97567175 7	d__Bacteria	p__Firmicutes_C	c__Negativicutes	o__Veillonellales	f__Megasphaeraceae	g__Megasphaera	s__Megasphaera sp000417505
MGYG-HGUT-02340:s__Intestinimonas butyriciproducens	0.59487242 5	0.14415727 4	0.97567175 7	d__Bacteria	p__Firmicutes_A	c__Clostridia	o__Oscillospirales	f__Oscillospiraceae	g__Intestinimonas	s__Intestinimonas butyriciproducens
MGYG-HGUT-02347:s__Streptococcus anginosus_C	0.50583054	0.18272923 1	0.96853207 3	d__Bacteria	p__Firmicutes	c__Bacilli	o__Lactobacillales	f__Streptococcaceae	g__Streptococcus	s__Streptococcus anginosus_C
MGYG-HGUT-02349:s__Streptococcus agalactiae	0.61643266 2	-0.13843670 1	0.97567175 7	d__Bacteria	p__Firmicutes	c__Bacilli	o__Lactobacillales	f__Streptococcaceae	g__Streptococcus	s__Streptococcus agalactiae
MGYG-HGUT-02351:s__Klebsiella_A michiganensis	0.82209	-0.05875	0.99113	d__Bact	p__Proteobacteria	c__Gammaproteo	o__Enterobacterales	f__Enterobacteriaceae	g__Klebsiella_A	s__Klebsiella_A michiganensis

	7691	2342	6622	eria		bacteria				
MGYG-HGUT-02352:s__Gordonibacter urolithinfaciens	0.335227906	0.269686907	0.930624692	d__Bacteria	p__Actinobacteriota	c__Coriobacteriia	o__Coriobacteriales	f__Eggerthellaceae	g__Gordonibacter	s__Gordonibacter urolithinfaciens
MGYG-HGUT-02353:s__Enterococcus_B faecium	0.0527437346	-0.537369804	0.85721132	d__Bacteria	p__Firmicutes	c__Bacilli	o__Lactobacillales	f__Enterococcaceae	g__Enterococcus_B	s__Enterococcus_B faecium
MGYG-HGUT-02363:s__Raoultella ornithinolytica	0.396530474	0.224454547	0.93970925	d__Bacteria	p__Proteobacteria	c__Gammaproteobacteria	o__Enterobacterales	f__Enterobacteriaceae	g__Raoultella	s__Raoultella ornithinolytica
MGYG-HGUT-02366:s__Salmonella enterica	0.931569095	-0.023123015	0.994479474	d__Bacteria	p__Proteobacteria	c__Gammaproteobacteria	o__Enterobacterales	f__Enterobacteriaceae	g__Salmonella	s__Salmonella enterica
MGYG-HGUT-02369:s__Clostridioides difficile	0.436235045	-0.207629528	0.950806594	d__Bacteria	p__Firmicutes_A	c__Clostridia	o__Peptostreptococcales	f__Peptostreptococcaceae	g__Clostridioides	s__Clostridioides difficile
MGYG-HGUT-02376:s__Dorea sp000765215	0.183932262	-0.370069602	0.891825559	d__Bacteria	p__Firmicutes_A	c__Clostridia	o__Lachnospirales	f__Lachnospiraceae	g__Dorea	s__Dorea sp000765215
MGYG-HGUT-02378:s__Akkermansia sp001580195	0.541243498	0.163949237	0.968532073	d__Bacteria	p__Verrucomicrobiota	c__Verrucomicrobiae	o__Verrucomicrobiales	f__Akkermansiaceae	g__Akkermansia	s__Akkermansia sp001580195
MGYG-HGUT-02387:s__Lactobacillus gasseri_A	0.063279098	0.496530146	0.85721132	d__Bacteria	p__Firmicutes	c__Bacilli	o__Lactobacillales	f__Lactobacillaceae	g__Lactobacillus	s__Lactobacillus gasseri_A

MGYG-HGUT-02388:s__Lactobacillus_C paracasei	0.395186991	-0.235646839	0.939709925	d__Bacteria	p__Firmicutes	c__Bacilli	o__Lactobacillales	f__Lactobacillaceae	g__Lactobacillus_C	s__Lactobacillus_C paracasei
MGYG-HGUT-02393:s__Dorea faecis	0.124349122	-0.426122479	0.891825559	d__Bacteria	p__Firmicutes_A	c__Clostridia	o__Lachnospirales	f__Lachnospiraceae	g__Dorea	s__Dorea faecis
MGYG-HGUT-02394:s__Prevotella sp001275135	0.566509014	0.160415156	0.971333903	d__Bacteria	p__Bacteroidota	c__Bacteroidia	o__Bacteroidales	f__Bacteroidaceae	g__Prevotella	s__Prevotella sp001275135
MGYG-HGUT-02395:s__Bifidobacterium adolescentis	0.5953611	-0.140708 42	0.975671757	d__Bacteria	p__Actinobacteriota	c__Actinobacteria	o__Actinomycetales	f__Bifidobacteriaceae	g__Bifidobacterium	s__Bifidobacterium adolescentis
MGYG-HGUT-02396:s__Bifidobacterium bifidum	0.352759887	0.253811099	0.939709925	d__Bacteria	p__Actinobacteriota	c__Actinobacteria	o__Actinomycetales	f__Bifidobacteriaceae	g__Bifidobacterium	s__Bifidobacterium bifidum
MGYG-HGUT-02397:s__Bifidobacterium angulatum	0.407067323	0.225829916	0.939709925	d__Bacteria	p__Actinobacteriota	c__Actinobacteria	o__Actinomycetales	f__Bifidobacteriaceae	g__Bifidobacterium	s__Bifidobacterium angulatum
MGYG-HGUT-02410:s__Provencibacterium massiliense	0.742408336	0.081838158	0.979458475	d__Bacteria	p__Firmicutes_A	c__Clostridia	o__Oscillospirales	f__Ruminococcaceae	g__Provencibacterium	s__Provencibacterium massiliense
MGYG-HGUT-02412:s__Prevotellamassilia timonensis	0.4975137	0.186454771	0.968532073	d__Bacteria	p__Bacteroidota	c__Bacteroidia	o__Bacteroidales	f__Bacteroidaceae	g__Prevotellamassilia	s__Prevotellamassilia timonensis
MGYG-HGUT-02416:s__An200 sp003268275	0.646982779	0.127449285	0.975671757	d__Bacteria	p__Firmicutes_A	c__Clostridia	o__Oscillospirales	f__Acutalibacteraceae	g__An200	s__An200 sp003268275

MGYG-HGUT-02417:s__Lachnoclostridium_A edouardi	0.191984162	0.362936 83	0.891825559	d__Bacteria	p__Firmicutes_A	c__Clostridia	o__Lachnospirales	f__Lachnospiraceae	g__Lachnoclostridium_A	s__Lachnoclostridium_A edouardi
MGYG-HGUT-02418:s__Parabacteroides chinchillae	0.246642142	-0.308693184	0.902181367	d__Bacteria	p__Bacteroidota	c__Bacteroidia	o__Bacteroidales	f__Tannerellaceae	g__Parabacteroides	s__Parabacteroides chinchillae
MGYG-HGUT-02424:s__Monoglobus pectinilyticus	0.806095852	0.069002998	0.987142302	d__Bacteria	p__Firmicutes_A	c__Clostridia	o__Monoglobales	f__Monoglobaceae	g__Monoglobus	s__Monoglobus pectinilyticus
MGYG-HGUT-02437:s__Blautia hominis	0.299271626	-0.288930105	0.929025913	d__Bacteria	p__Firmicutes_A	c__Clostridia	o__Lachnospirales	f__Lachnospiraceae	g__Blautia	s__Blautia hominis
MGYG-HGUT-02438:s__Parabacteroides distasonis	0.063407482	-0.500041096	0.85721132	d__Bacteria	p__Bacteroidota	c__Bacteroidia	o__Bacteroidales	f__Tannerellaceae	g__Parabacteroides	s__Parabacteroides distasonis
MGYG-HGUT-02439:s__CHH4-2 sp002899675	0.5010147 1	-0.185374009	0.968532073	d__Bacteria	p__Firmicutes_A	c__Clostridia	o__Lachnospirales	f__Lachnospiraceae	g__CHH4-2	s__CHH4-2 sp002899675
MGYG-HGUT-02442:s__Gordonibacter unknown	0.463065199	0.205703481	0.965574692	d__Bacteria	p__Actinobacteriota	c__Coriobacteriia	o__Coriobacteriales	f__Eggerthellaceae	g__Gordonibacter	s__Gordonibacter unknown
MGYG-HGUT-02444:s__Erysipelatoclostridium sp003024675	0.962055627	-0.013336 91	0.994479474	d__Bacteria	p__Firmicutes	c__Bacilli	o__Erysipelotrichales	f__Erysipelatoclostridiaceae	g__Erysipelatoclostridium	s__Erysipelatoclostridium sp003024675
MGYG-HGUT-02445:s__Clostridi	0.63523	0.12941	0.97567	d__Bact	p__Firmicutes_A	c__Clostridia	o__Lachnospirales	f__Lachnospiraceae	g__Clostridium_Q	s__Clostridium_Q sp003024715

um_Q sp003024715	4065	943	1757	eria						
MGYG-HGUT-02446:s__Methanobrevibacter_A smithii_A	0.152624323	-0.377612826	0.891825559	d__Archaea	p__Euryarchaeota	c__Methanobacteria	o__Methanobacteriales	f__Methanobacteriaceae	g__Methanobrevibacter_A	s__Methanobrevibacter_A smithii_A
MGYG-HGUT-02448:s__Ruminiclostridium_C sp000435295	0.533201193	-0.169230999	0.968532073	d__Bacteria	p__Firmicutes_A	c__Clostridia	o__Oscillospirales	f__Oscillospiraceae	g__Ruminiclostridium_C	s__Ruminiclostridium_C sp000435295
MGYG-HGUT-02449:s__Parabacteroides unknown_10	0.204825211	-0.353197559	0.891825559	d__Bacteria	p__Bacteroidota	c__Bacteroidia	o__Bacteroidales	f__Tannerellaceae	g__Parabacteroides	s__Parabacteroides unknown_10
MGYG-HGUT-02450:s__Olsenella_E unknown_1	0.923384655	0.026721382	0.994479474	d__Bacteria	p__Actinobacteriota	c__Coriobacteriia	o__Coriobacteriales	f__Atopobiaceae	g__Olsenella_E	s__Olsenella_E unknown_1
MGYG-HGUT-02453:s__Akkermansia muciniphila_B	0.864979666	0.046852064	0.994479474	d__Bacteria	p__Verrucomicrobiota	c__Verrucomicrobiae	o__Verrucomicrobiales	f__Akkermansiaceae	g__Akkermansia	s__Akkermansia muciniphila_B
MGYG-HGUT-02454:s__Akkermansia muciniphila	0.714396099	-0.098228664	0.979458475	d__Bacteria	p__Verrucomicrobiota	c__Verrucomicrobiae	o__Verrucomicrobiales	f__Akkermansiaceae	g__Akkermansia	s__Akkermansia muciniphila
MGYG-HGUT-02455:s__Bacteroides cellulosilyticus	0.144295826	-0.403490294	0.891825559	d__Bacteria	p__Bacteroidota	c__Bacteroidia	o__Bacteroidales	f__Bacteroidaceae	g__Bacteroides	s__Bacteroides cellulosilyticus
MGYG-HGUT-02459:s__Bifidobacterium animalis	0.938621076	0.021609647	0.994479474	d__Bacteria	p__Actinobacteriota	c__Actinobacteria	o__Actinomycetales	f__Bifidobacteriaceae	g__Bifidobacterium	s__Bifidobacterium animalis

MGYG-HGUT-02464:s__Acetivibrio_A ethanolgignens	0.77579553 8	-0.078717171 19	0.97952048 4	d__Bacteria	p__Firmicutes_A	c__Clostridia	o__Lachnospirales	f__Lachnospiraceae	g__Acetivibrio_A	s__Acetivibrio_A ethanolgignens
MGYG-HGUT-02469:s__Bifidobacterium breve	0.77969776	-0.076895454 57	0.97952048 4	d__Bacteria	p__Actinobacteriota	c__Actinobacteria	o__Actinomycetales	f__Bifidobacteriaceae	g__Bifidobacterium	s__Bifidobacterium breve
MGYG-HGUT-02470:s__Bacteroides intestinalis	0.87417336 5	-0.042814000 3	0.99447947 4	d__Bacteria	p__Bacteroidota	c__Bacteroidia	o__Bacteroidales	f__Bacteroidaceae	g__Bacteroides	s__Bacteroides intestinalis
MGYG-HGUT-02475:s__Lactonifactor longoviformis	0.12518059 3	-0.42419534	0.89182555 9	d__Bacteria	p__Firmicutes_A	c__Clostridia	o__Lachnospirales	f__Lachnospiraceae	g__Lactonifactor	s__Lactonifactor longoviformis
MGYG-HGUT-02477:s__Citrobacter_A amalonaticus_D	0.93653966 4	0.021992707	0.99447947 4	d__Bacteria	p__Proteobacteria	c__Gammaproteobacteria	o__Enterobacterales	f__Enterobacteriaceae	g__Citrobacter_A	s__Citrobacter_A amalonaticus_D
MGYG-HGUT-02478:s__Bacteroides_B dorei	0.37100624 8	0.24868335 4	0.93970925	d__Bacteria	p__Bacteroidota	c__Bacteroidia	o__Bacteroidales	f__Bacteroidaceae	g__Bacteroides_B	s__Bacteroides_B dorei
MGYG-HGUT-02480:s__Adlercreutzia equolifaciens	0.30338149 4	-0.278871644 4	0.92902591 3	d__Bacteria	p__Actinobacteriota	c__Coriobacteriia	o__Coriobacteriales	f__Eggerthellaceae	g__Adlercreutzia	s__Adlercreutzia equolifaciens
MGYG-HGUT-02482:s__Ruminococcus_E bromii	0.43466902 2	0.21566245 7	0.95080659 4	d__Bacteria	p__Firmicutes_A	c__Clostridia	o__Oscillospirales	f__Acutalibacteraceae	g__Ruminococcus_E	s__Ruminococcus_E bromii
MGYG-HGUT-02483:s__Clostridi	0.57777 77	0.15481	0.97567	d__Bact	p__Firmicutes_A	c__Clostridia	o__Lachnospirales	f__Lachnospiraceae	g__Clostridium_Q	s__Clostridium_Q

um_Q saccharolyticum	796	5941	1757	eria						saccharolyticum
MGYG-HGUT-02487:s__Gordonibacter pamelaeae	0.761896574	0.0846366694	0.979458475	d__Bacteria	p__Actinobacteriota	c__Coriobacteriia	o__Coriobacteriales	f__Eggerthellaceae	g__Gordonibacter	s__Gordonibacter pamelaeae
MGYG-HGUT-02491:s__Kluyvera ascorbata_B	0.938770437	-0.02100932	0.994479474	d__Bacteria	p__Proteobacteria	c__Gammaproteobacteria	o__Enterobacterales	f__Enterobacteriaceae	g__Kluyvera	s__Kluyvera ascorbata_B
MGYG-HGUT-02492:s__Agathobacter rectalis	0.991427157	-0.002730479	0.998803346	d__Bacteria	p__Firmicutes_A	c__Clostridia	o__Lachnospirales	f__Lachnospiraceae	g__Agathobacter	s__Agathobacter rectalis
MGYG-HGUT-02494:s__Citrobacter_B koseri	0.770534026	0.080735569	0.979520484	d__Bacteria	p__Proteobacteria	c__Gammaproteobacteria	o__Enterobacterales	f__Enterobacteriaceae	g__Citrobacter_B	s__Citrobacter_B koseri
MGYG-HGUT-02500:s__Enterobacter cloacae	0.289036249	-0.286940079	0.929025913	d__Bacteria	p__Proteobacteria	c__Gammaproteobacteria	o__Enterobacterales	f__Enterobacteriaceae	g__Enterobacter	s__Enterobacter cloacae
MGYG-HGUT-02504:s__Enterobacter himalayensis	0.865674048	0.043956729	0.994479474	d__Bacteria	p__Proteobacteria	c__Gammaproteobacteria	o__Enterobacterales	f__Enterobacteriaceae	g__Enterobacter	s__Enterobacter himalayensis
MGYG-HGUT-02506:s__Escherichia coli_D	0.806404511	-0.067078011	0.987142302	d__Bacteria	p__Proteobacteria	c__Gammaproteobacteria	o__Enterobacterales	f__Enterobacteriaceae	g__Escherichia	s__Escherichia coli_D
MGYG-HGUT-02507:s__Escherichia albertii	0.93377576	0.022408263	0.994479474	d__Bacteria	p__Proteobacteria	c__Gammaproteobacteria	o__Enterobacterales	f__Enterobacteriaceae	g__Escherichia	s__Escherichia albertii

MGYG-HGUT-02509:s__Slackia_A unknown_3	0.4496432 21	0.2066726 42	0.9612922 08	d__Bacteria	p__Actinobacteriota	c__Coriobacteriia	o__Coriobacteriales	f__Eggerthellaceae	g__Slackia_A	s__Slackia_A unknown_3
MGYG-HGUT-02510:s__Klebsiella quasipneumoniae	0.0942090 45	0.4379427 38	0.8833072 56	d__Bacteria	p__Proteobacteria	c__Gammaproteobacteria	o__Enterobacterales	f__Enterobacteriaceae	g__Klebsiella	s__Klebsiella quasipneumoniae
MGYG-HGUT-02511:s__Klebsiella variicola	0.7325503 81	0.0871739 91	0.9794584 75	d__Bacteria	p__Proteobacteria	c__Gammaproteobacteria	o__Enterobacterales	f__Enterobacteriaceae	g__Klebsiella	s__Klebsiella variicola
MGYG-HGUT-02515:s__Escherichia fergusonii	0.4821018 33	-0.1903549 65	0.9685320 73	d__Bacteria	p__Proteobacteria	c__Gammaproteobacteria	o__Enterobacterales	f__Enterobacteriaceae	g__Escherichia	s__Escherichia fergusonii
MGYG-HGUT-02517:s__Roseburia hominis	0.2862058 77	0.2898287 68	0.9290259 13	d__Bacteria	p__Firmicutes_A	c__Clostridia	o__Lachnospirales	f__Lachnospiraceae	g__Roseburia	s__Roseburia hominis
MGYG-HGUT-02519:s__Enterobacter sesami	0.9665138 51	0.0116955 93	0.9944794 74	d__Bacteria	p__Proteobacteria	c__Gammaproteobacteria	o__Enterobacterales	f__Enterobacteriaceae	g__Enterobacter	s__Enterobacter sesami
MGYG-HGUT-02521:s__Metakosakonia intermedia	0.8154971 28	-0.0626803 45	0.9876645 39	d__Bacteria	p__Proteobacteria	c__Gammaproteobacteria	o__Enterobacterales	f__Enterobacteriaceae	g__Metakosakonia	s__Metakosakonia intermedia
MGYG-HGUT-02523:s__Christensenella minuta	0.5632562 44	0.1591370 04	0.9713390 3	d__Bacteria	p__Firmicutes_A	c__Clostridia	o__Christensenellales	f__Christensenellaceae	g__Christensenella	s__Christensenella minuta
MGYG-HGUT-02528:s__Anaerostipes hadrus	0.6899610 64	0.1070851 6	0.9757685 79	d__Bacteria	p__Firmicutes_A	c__Clostridia	o__Lachnospirales	f__Lachnospiraceae	g__Anaerostipes	s__Anaerostipes hadrus

MGYG-HGUT-02530:s__Ruminiclostridium_E siraeum	0.509589545	0.174058297	0.968532073	d__Bacteria	p__Firmicutes_A	c__Clostridia	o__Oscillospirales	f__Ruminococcaceae	g__Ruminiclostridium_E	s__Ruminiclostridium_E siraeum
MGYG-HGUT-02534:s__Citrobacter_A farmeri	0.920088734	0.026986672	0.994479474	d__Bacteria	p__Proteobacteria	c__Gammaproteobacteria	o__Enterobacterales	f__Enterobacteriaceae	g__Citrobacter_A	s__Citrobacter_A farmeri
MGYG-HGUT-02538:s__Klebsiella pneumoniae	0.708008204	-0.093956279	0.979458475	d__Bacteria	p__Proteobacteria	c__Gammaproteobacteria	o__Enterobacterales	f__Enterobacteriaceae	g__Klebsiella	s__Klebsiella pneumoniae
MGYG-HGUT-02539:s__Collinsella aerofaciens_A_1	0.50976059	0.183497973	0.968532073	d__Bacteria	p__Actinobacteriota	c__Coriobacteriia	o__Coriobacteriales	f__Coriobacteriaceae	g__Collinsella	s__Collinsella aerofaciens_A_1
MGYG-HGUT-02540:s__Paraprevotella clara	0.815148982	-0.065308363	0.987664539	d__Bacteria	p__Bacteroidota	c__Bacteroidia	o__Bacteroidales	f__Bacteroidaceae	g__Paraprevotella	s__Paraprevotella clara
MGYG-HGUT-02544:s__Eggerthella lenta	0.678715122	-0.115438453	0.975671757	d__Bacteria	p__Actinobacteriota	c__Coriobacteriia	o__Coriobacteriales	f__Eggerthellaceae	g__Eggerthella	s__Eggerthella lenta
MGYG-HGUT-02545:s__Faecalibacterium prausnitzii_G	0.399583458	0.236136604	0.93970925	d__Bacteria	p__Firmicutes_A	c__Clostridia	o__Oscillospirales	f__Ruminococcaceae	g__Faecalibacterium	s__Faecalibacterium prausnitzii_G
MGYG-HGUT-02546:s__Neobitarella massiliensis	0.45788932	0.208249966	0.965574692	d__Bacteria	p__Firmicutes_A	c__Clostridia	o__Oscillospirales	f__Ruminococcaceae	g__Neobitarella	s__Neobitarella massiliensis
MGYG-HGUT-02547:s__Megasphaera elsdenii	0.728633193	0.095044269	0.979458475	d__Bacteria	p__Firmicutes_C	c__Negativicutes	o__Veillonellales	f__Megasphaeraceae	g__Megasphaera	s__Megasphaera elsdenii

MGYG-HGUT-02549:s__Bacteroides caccae	0.213321738	-0.346690931	0.891825559	d__Bacteria	p__Bacteroidota	c__Bacteroidia	o__Bacteroidales	f__Bacteroidaceae	g__Bacteroides	s__Bacteroides caccae
MGYG-HGUT-02550:s__Streptococcus sp001556435	0.605409772	0.144559928	0.975671757	d__Bacteria	p__Firmicutes	c__Bacilli	o__Lactobacillales	f__Streptococcaceae	g__Streptococcus	s__Streptococcus sp001556435
MGYG-HGUT-02552:s__Lachnospira sp000436535	0.960362627	0.013743481	0.994479474	d__Bacteria	p__Firmicutes_A	c__Clostridia	o__Lachnospirales	f__Lachnospiraceae	g__Lachnospira	s__Lachnospira sp000436535
MGYG-HGUT-02554:s__Streptococcus oralis_AE	0.0436005	0.553977019	0.779321052	d__Bacteria	p__Firmicutes	c__Bacilli	o__Lactobacillales	f__Streptococcaceae	g__Streptococcus	s__Streptococcus oralis_AE
MGYG-HGUT-02556:s__CAG-279 sp000437795	0.750574965	0.08901521	0.979458475	d__Bacteria	p__Bacteroidota	c__Bacteroidia	o__Bacteroidales	f__Muribaculaceae	g__CAG-279	s__CAG-279 sp000437795
MGYG-HGUT-02557:s__Collinsella unknown_217	0.794781628	-0.072774208	0.9829663	d__Bacteria	p__Actinobacteriota	c__Coriobacteriia	o__Coriobacteriales	f__Coriobacteriaceae	g__Collinsella	s__Collinsella unknown_217
MGYG-HGUT-02559:s__Lachnospira unknown_3	0.718071297	0.099782892	0.979458475	d__Bacteria	p__Firmicutes_A	c__Clostridia	o__Lachnospirales	f__Lachnospiraceae	g__Lachnospira	s__Lachnospira unknown_3
MGYG-HGUT-02560:s__Bacteroides_B unknown	0.149968408	0.398864145	0.891825559	d__Bacteria	p__Bacteroidota	c__Bacteroidia	o__Bacteroidales	f__Bacteroidaceae	g__Bacteroides_B	s__Bacteroides_B unknown
MGYG-HGUT-02561:s__Bacteroides unknown_5	0.713636277	0.102565463	0.979458475	d__Bacteria	p__Bacteroidota	c__Bacteroidia	o__Bacteroidales	f__Bacteroidaceae	g__Bacteroides	s__Bacteroides unknown_5

MGYG-HGUT-02563:s__Romboutsia timonensis	0.7516089 94	-0.088635246	0.9794584 75	d__Bacteria	p__Firmicutes_A	c__Clostridia	o__Peptostreptococcales	f__Peptostreptococcaceae	g__Romboutsia	s__Romboutsia timonensis
MGYG-HGUT-02570:s__Ruminococcus_E sp003526955	0.8282786 75	-0.060144354	0.9922201 9	d__Bacteria	p__Firmicutes_A	c__Clostridia	o__Oscillospirales	f__Acutalibacteraceae	g__Ruminococcus_E	s__Ruminococcus_E sp003526955
MGYG-HGUT-02573:s__Ruminococcus_C unknown_2	0.9482150 94	0.0181129 88	0.9944794 74	d__Bacteria	p__Firmicutes_A	c__Clostridia	o__Oscillospirales	f__Ruminococcaceae	g__Ruminococcus_C	s__Ruminococcus_C unknown_2
MGYG-HGUT-02574:s__Fusobacterium_A unknown_2	0.6503267 74	0.1257690 51	0.9756717 57	d__Bacteria	p__Fusobacteriota	c__Fusobacteriia	o__Fusobacteriales	f__Fusobacteriaceae	g__Fusobacterium_A	s__Fusobacterium_A unknown_2
MGYG-HGUT-02575:s__Bacteroidaceae unknown unknown_3	0.1924989 75	0.3463115 13	0.8918255 59	d__Bacteria	p__Bacteroidota	c__Bacteroidia	o__Bacteroidales	f__Bacteroidaceae	g__Bacteroidaceae unknown	s__Bacteroidaceae unknown unknown_3
MGYG-HGUT-02580:s__Lachnospira sp000436475	0.8703199 75	-0.045474661	0.9944794 74	d__Bacteria	p__Firmicutes_A	c__Clostridia	o__Lachnospirales	f__Lachnospiraceae	g__Lachnospira	s__Lachnospira sp000436475
MGYG-HGUT-02585:s__Bacteroides unknown_6	0.8325594 17	0.0590810 12	0.9922201 9	d__Bacteria	p__Bacteroidota	c__Bacteroidia	o__Bacteroidales	f__Bacteroidaceae	g__Bacteroides	s__Bacteroides unknown_6
MGYG-HGUT-02587:s__Parabacteroides unknown_11	0.1559875 91	-0.396023201	0.8918255 59	d__Bacteria	p__Bacteroidota	c__Bacteroidia	o__Bacteroidales	f__Tannerellaceae	g__Parabacteroides	s__Parabacteroides unknown_11
MGYG-HGUT-02588:s__Lawsonibacter unknown_7	0.80589	-0.06812	0.98714	d__Bact	p__Firmicutes_A	c__Clostridia	o__Oscillospirales	f__Oscillospiraceae	g__Lawsonibacter	s__Lawsonibacter unknown_7

	8432	145	2302	eria						
MGYG-HGUT-02589:s__CAG-110 unknown_19	0.235462079	0.3183593 61	0.9021813 67	d__Bacteria	p__Firmicutes_A	c__Clostridia	o__Oscillospirales	f__Oscillospiraceae	g__CAG-110	s__CAG-110 unknown_19
MGYG-HGUT-02592:s__Alistipes unknown_8	0.331835 44	-0.2575712 5	0.9306246 92	d__Bacteria	p__Bacteroidota	c__Bacteroidia	o__Bacteroidales	f__Rikenellaceae	g__Alistipes	s__Alistipes unknown_8
MGYG-HGUT-02594:s__Streptococcus unknown_10	0.9325718 01	0.0232785 05	0.9944794 74	d__Bacteria	p__Firmicutes	c__Bacilli	o__Lactobacillales	f__Streptococcaceae	g__Streptococcus	s__Streptococcus unknown_10
MGYG-HGUT-02595:s__CAG-83 unknown_11	0.9888262 81	0.0038149 06	0.9988033 46	d__Bacteria	p__Firmicutes_A	c__Clostridia	o__Oscillospirales	f__Oscillospiraceae	g__CAG-83	s__CAG-83 unknown_11
MGYG-HGUT-02596:s__Faecalicatena unknown_6	0.1907594 39	-0.3577223 97	0.8918255 59	d__Bacteria	p__Firmicutes_A	c__Clostridia	o__Lachnospirales	f__Lachnospiraceae	g__Faecalicatena	s__Faecalicatena unknown_6
MGYG-HGUT-02598:s__Blautia unknown_8	0.4424860 41	0.2094893 17	0.9602733 89	d__Bacteria	p__Firmicutes_A	c__Clostridia	o__Lachnospirales	f__Lachnospiraceae	g__Blautia	s__Blautia unknown_8
MGYG-HGUT-02601:s__CAG-110 unknown_20	0.7232522 92	0.0985080 19	0.9794584 75	d__Bacteria	p__Firmicutes_A	c__Clostridia	o__Oscillospirales	f__Oscillospiraceae	g__CAG-110	s__CAG-110 unknown_20
MGYG-HGUT-02602:s__CAG-274 unknown	0.1324841 02	-0.4180583 46	0.8918255 59	d__Bacteria	p__Firmicutes_A	c__Clostridia	o__Lachnospirales	f__CAG-274	g__CAG-274	s__CAG-274 unknown
MGYG-HGUT-02603:s__Prevotella unknown_30	0.72522	0.09487	0.97945	d__Bact	p__Bacter	c__Bacteroidia	o__Bacteroidales	f__Bacteroidaceae	g__Prevotella	s__Prevotella unknown_30

	648	1183	8475	eria	oidota					
MGYG-HGUT-02609:s__CAG-882 sp003486385	0.839220672	-0.057019907	0.992878088	d__Bacteria	p__Firmicutes_A	c__Clostridia	o__Lachnospirales	f__Lachnospiraceae	g__CAG-882	s__CAG-882 sp003486385
MGYG-HGUT-02610:s__Faecalibacterium unknown_8	0.060470314	0.516328562	0.857211132	d__Bacteria	p__Firmicutes_A	c__Clostridia	o__Oscillospirales	f__Ruminococcaceae	g__Faecalibacterium	s__Faecalibacterium unknown_8
MGYG-HGUT-02612:s__CAG-83 sp003539495	0.244651537	0.320276074	0.902181367	d__Bacteria	p__Firmicutes_A	c__Clostridia	o__Oscillospirales	f__Oscillospiraceae	g__CAG-83	s__CAG-83 sp003539495
MGYG-HGUT-02614:s__Bacteroides_A unknown_10	0.904706281	0.033504505	0.994479474	d__Bacteria	p__Bacteroidota	c__Bacteroidia	o__Bacteroidales	f__Bacteroidaceae	g__Bacteroides_A	s__Bacteroides_A unknown_10
MGYG-HGUT-02617:s__Oscillibacter unknown_14	0.064931828	0.506723437	0.857211132	d__Bacteria	p__Firmicutes_A	c__Clostridia	o__Oscillospirales	f__Oscillospiraceae	g__Oscillibacter	s__Oscillibacter unknown_14
MGYG-HGUT-02619:s__Faecalibacterium prausnitzii_J	0.019980887	0.623496161	0.768225834	d__Bacteria	p__Firmicutes_A	c__Clostridia	o__Oscillospirales	f__Ruminococcaceae	g__Faecalibacterium	s__Faecalibacterium prausnitzii_J
MGYG-HGUT-02625:s__Negativibacillus massiliensis	0.083331136	-0.480279165	0.881142843	d__Bacteria	p__Firmicutes_A	c__Clostridia	o__Oscillospirales	f__Ruminococcaceae	g__Negativibacillus	s__Negativibacillus massiliensis
MGYG-HGUT-02633:s__TF01-11 sp003149875	0.976836597	0.007861863	0.996587801	d__Bacteria	p__Firmicutes_A	c__Clostridia	o__Lachnospirales	f__Lachnospiraceae	g__TF01-11	s__TF01-11 sp003149875
MGYG-HGUT-02640:s__Veillonella parvula	0.31501	0.27431175	0.92902	d__Bact	p__Firmicutes_C	c__Negativicutes	o__Veillonellales	f__Veillonellaceae	g__Veillonella	s__Veillonella parvula

	42<br>22		59<br>13	eri<br>a						
MGYG-HGUT-02641:s__Faecalibacterium sp003449675	0.1<br>33<br>10<br>80<br>95	0.4<br>12<br>36<br>49<br>67	0.8<br>91<br>82<br>55<br>59	d__Bacteria	p__Firmicutes_A	c__Clostridia	o__Oscillospirales	f__Ruminococcaceae	g__Faecalibacterium	s__Faecalibacterium sp003449675
MGYG-HGUT-02642:s__Veillonella unknown_9	0.5<br>19<br>91<br>95<br>43	0.1<br>80<br>18<br>42<br>63	0.9<br>68<br>53<br>20<br>73	d__Bacteria	p__Firmicutes_C	c__Negativicutes	o__Veillonellales	f__Veillonellaceae	g__Veillonella	s__Veillonella unknown_9
MGYG-HGUT-02651:s__Faecalibacterium unknown_9	0.0<br>86<br>32<br>60<br>62	0.4<br>62<br>79<br>94<br>79	0.8<br>82<br>71<br>60<br>64	d__Bacteria	p__Firmicutes_A	c__Clostridia	o__Oscillospirales	f__Ruminococcaceae	g__Faecalibacterium	s__Faecalibacterium unknown_9
MGYG-HGUT-02653:s__Prevotella sp002251295	0.7<br>95<br>39<br>41<br>07	-<br>0.0<br>72<br>84<br>66<br>07	0.9<br>82<br>96<br>63	d__Bacteria	p__Bacteroidota	c__Bacteroidia	o__Bacteroidales	f__Bacteroidaceae	g__Prevotella	s__Prevotella sp002251295
MGYG-HGUT-02657:s__CAG-95 unknown_3	0.1<br>89<br>55<br>11<br>02	0.3<br>62<br>41<br>96<br>22	0.8<br>91<br>82<br>55<br>59	d__Bacteria	p__Firmicutes_A	c__Clostridia	o__Lachnospirales	f__Lachnospiraceae	g__CAG-95	s__CAG-95 unknown_3
MGYG-HGUT-02659:s__Phascolarctobacterium_A unknown_10	0.7<br>47<br>33<br>50<br>22	0.0<br>90<br>04<br>16<br>02	0.9<br>79<br>45<br>84<br>75	d__Bacteria	p__Firmicutes_C	c__Negativicutes	o__Acidaminococcales	f__Acidaminococcaceae	g__Phascolarctobacterium_A	s__Phascolarctobacterium_A unknown_10
MGYG-HGUT-02667:s__F23-B02 unknown_6	0.6<br>48<br>36<br>30<br>11	-<br>0.1<br>25<br>44<br>31<br>22	0.9<br>75<br>67<br>17<br>57	d__Bacteria	p__Firmicutes_A	c__Clostridia	o__Oscillospirales	f__Oscillospiraceae	g__F23-B02	s__F23-B02 unknown_6
MGYG-HGUT-02668:s__Prevotella unknown_33	0.6<br>50<br>51<br>82<br>63	0.1<br>25<br>58<br>57<br>91	0.9<br>75<br>67<br>17<br>57	d__Bacteria	p__Bacteroidota	c__Bacteroidia	o__Bacteroidales	f__Bacteroidaceae	g__Prevotella	s__Prevotella unknown_33
MGYG-HGUT-02670:s__Agathobacter unknown_5	0.8<br>53<br>08	-<br>0.0<br>48<br>91	0.9<br>92<br>87	d__Bact	p__Firmicutes_A	c__Clostridia	o__Lachnospirales	f__Lachnospiraceae	g__Agathobacter	s__Agathobacter unknown_5

	00<br>48	19<br>58	80<br>88	eri<br>a						
MGYG-HGUT-02673:s__UBA9475 unknown_2	0.586166137	0.147677109	0.975671757	d__Bacteria	p__Firmicutes_A	c__Clostridia	o__Oscillospirales	f__Oscillospiraceae	g__UBA9475	s__UBA9475 unknown_2
MGYG-HGUT-02680:s__Oscillibacter unknown_15	0.038275179	-0.552606499	0.768225834	d__Bacteria	p__Firmicutes_A	c__Clostridia	o__Oscillospirales	f__Oscillospiraceae	g__Oscillibacter	s__Oscillibacter unknown_15
MGYG-HGUT-02681:s__QAND01 unknown unknown_4	0.25507026	-0.317604492	0.905015696	d__Bacteria	p__Firmicutes_A	c__Clostridia	o__Christensenellales	f__QAND01	g__QAND01 unknown	s__QAND01 unknown unknown_4
MGYG-HGUT-02682:s__Oscillibacter unknown_16	0.604034161	0.131507276	0.975671757	d__Bacteria	p__Firmicutes_A	c__Clostridia	o__Oscillospirales	f__Oscillospiraceae	g__Oscillibacter	s__Oscillibacter unknown_16
MGYG-HGUT-02684:s__UBA3818 unknown_2	0.833385935	-0.058425603	0.99222019	d__Bacteria	p__Firmicutes_A	c__Clostridia	o__Oscillospirales	f__Ruminococcaceae	g__UBA3818	s__UBA3818 unknown_2
MGYG-HGUT-02685:s__UBA9506 unknown unknown_1	0.881623526	-0.041621247	0.994479474	d__Bacteria	p__Firmicutes_A	c__Clostridia	o__Monoglobales	f__UBA9506	g__UBA9506 unknown	s__UBA9506 unknown unknown_1
MGYG-HGUT-02689:s__Ruminococcaceae unknown unknown_16	0.934460272	-0.023021704	0.994479474	d__Bacteria	p__Firmicutes_A	c__Clostridia	o__Oscillospirales	f__Ruminococcaceae	g__Ruminococcaceae unknown	s__Ruminococcaceae unknown unknown_16
MGYG-HGUT-02691:s__Oscillibacter unknown_17	0.565511258	0.156621589	0.97133903	d__Bacteria	p__Firmicutes_A	c__Clostridia	o__Oscillospirales	f__Oscillospiraceae	g__Oscillibacter	s__Oscillibacter unknown_17

MGYG-HGUT-02693:s__Eubacterium_E sp002161065	0.6754301 97	0.1148989 49	0.9756717 57	d__Bacteria	p__Firmicutes_A	c__Clostridia	o__Lachnospirales	f__Lachnospiraceae	g__Eubacterium_E	s__Eubacterium_E sp002161065
MGYG-HGUT-02694:s__Acutalibacteraceae unknown unknown_27	0.9468199 05	-0.0187132 65	0.9944794 74	d__Bacteria	p__Firmicutes_A	c__Clostridia	o__Oscillospirales	f__Acutalibacteraceae	g__Acutalibacteraceae unknown	s__Acutalibacteraceae unknown unknown_27
MGYG-HGUT-02696:s__Oscillospiraceae unknown unknown_11	0.1880585 49	-0.3477970 61	0.8918255 59	d__Bacteria	p__Firmicutes_A	c__Clostridia	o__Oscillospirales	f__Oscillospiraceae	g__Oscillospiraceae unknown	s__Oscillospiraceae unknown unknown_11
MGYG-HGUT-02697:s__Acutalibacteraceae unknown unknown_28	0.7269024 74	0.0950401 81	0.9794584 75	d__Bacteria	p__Firmicutes_A	c__Clostridia	o__Oscillospirales	f__Acutalibacteraceae	g__Acutalibacteraceae unknown	s__Acutalibacteraceae unknown unknown_28
MGYG-HGUT-02704:s__Oscillibacter unknown_18	0.9643867 39	0.0118410 25	0.9944794 74	d__Bacteria	p__Firmicutes_A	c__Clostridia	o__Oscillospirales	f__Oscillospiraceae	g__Oscillibacter	s__Oscillibacter unknown_18
MGYG-HGUT-02707:s__Flavonifractor unknown_2	0.2524290 34	0.2986607 32	0.9021813 67	d__Bacteria	p__Firmicutes_A	c__Clostridia	o__Oscillospirales	f__Oscillospiraceae	g__Flavonifractor	s__Flavonifractor unknown_2
MGYG-HGUT-02708:s__UBA3818 unknown_3	0.6240280 99	0.1369156 97	0.9756717 57	d__Bacteria	p__Firmicutes_A	c__Clostridia	o__Oscillospirales	f__Ruminococcaceae	g__UBA3818	s__UBA3818 unknown_3
MGYG-HGUT-02710:s__CAG-170 unknown_8	0.2981225 06	0.2673728 99	0.9290259 13	d__Bacteria	p__Firmicutes_A	c__Clostridia	o__Oscillospirales	f__Oscillospiraceae	g__CAG-170	s__CAG-170 unknown_8
MGYG-HGUT-02711:s__Acutalibacteraceae unknown unknown_33	0.6405505 72	-0.1164952 2	0.9756717 57	d__Bacteria	p__Firmicutes_A	c__Clostridia	o__Oscillospirales	f__Acutalibacteraceae	g__Acutalibacteraceae unknown	s__Acutalibacteraceae unknown unknown_33

MGYG-HGUT-02712:s__Adlercreutzia unknown	0.713416878	-0.102372777	0.979458475	d__Bacteria	p__Actinobacteriota	c__Coriobacteriia	o__Coriobacteriales	f__Eggerthellaceae	g__Adlercreutzia	s__Adlercreutzia unknown
MGYG-HGUT-02713:s__UBA4716 unknown_3	0.368236292	-0.248494619	0.93970925	d__Bacteria	p__Firmicutes_A	c__Clostridia	o__Monoglobales	f__UBA1381	g__UBA4716	s__UBA4716 unknown_3
MGYG-HGUT-02715:s__ER4 sp900317525	0.077077114	0.439903652	0.877826247	d__Bacteria	p__Firmicutes_A	c__Clostridia	o__Oscillospirales	f__Oscillospiraceae	g__ER4	s__ER4 sp900317525
MGYG-HGUT-02716:s__F23-B02 unknown_7	0.110272484	0.442302427	0.883307256	d__Bacteria	p__Firmicutes_A	c__Clostridia	o__Oscillospirales	f__Oscillospiraceae	g__F23-B02	s__F23-B02 unknown_7
MGYG-HGUT-02717:s__Bacteroides unknown_7	0.215952955	0.345637615	0.891825559	d__Bacteria	p__Bacteroidota	c__Bacteroidia	o__Bacteroidales	f__Bacteroidaceae	g__Bacteroides	s__Bacteroides unknown_7
MGYG-HGUT-02718:s__Butyricicoccus unknown_2	0.675254456	0.117680221	0.975671757	d__Bacteria	p__Firmicutes_A	c__Clostridia	o__Oscillospirales	f__Butyricicoccaceae	g__Butyricicoccus	s__Butyricicoccus unknown_2
MGYG-HGUT-02719:s__Clostridia unknown unknown unknown unknown_4	0.179444453	0.373536858	0.891825559	d__Bacteria	p__Firmicutes_A	c__Clostridia	o__Clostridia unknown	f__Clostridia unknown unknown	g__Clostridia unknown unknown unknown	s__Clostridia unknown unknown unknown unknown_4
MGYG-HGUT-02720:s__CAG-83 sp000435555	0.41315388	0.216458605	0.93970925	d__Bacteria	p__Firmicutes_A	c__Clostridia	o__Oscillospirales	f__Oscillospiraceae	g__CAG-83	s__CAG-83 sp000435555
MGYG-HGUT-02724:s__CAG-83 unknown_12	0.519307366	0.170465438	0.968532073	d__Bacteria	p__Firmicutes_A	c__Clostridia	o__Oscillospirales	f__Oscillospiraceae	g__CAG-83	s__CAG-83 unknown_12

MGYG-HGUT-02726:s__Oscillospiraceae unknown unknown_12	0.507253926	-0.18428282825	0.968532073	d__Bacteria	p__Firmicutes_A	c__Clostridia	o__Oscillospirales	f__Oscillospiraceae	g__Oscillospiraceae unknown	s__Oscillospiraceae unknown unknown_12
MGYG-HGUT-02727:s__Oscillospiraceae unknown unknown_13	0.305289263	0.282286619	0.929025913	d__Bacteria	p__Firmicutes_A	c__Clostridia	o__Oscillospirales	f__Oscillospiraceae	g__Oscillospiraceae unknown	s__Oscillospiraceae unknown unknown_13
MGYG-HGUT-02733:s__Collinsella unknown_226	0.75400215	0.085529798	0.979458475	d__Bacteria	p__Actinobacteriota	c__Coriobacteriia	o__Coriobacteriales	f__Coriobacteriaceae	g__Collinsella	s__Collinsella unknown_226
MGYG-HGUT-02735:s__Collinsella unknown_228	0.954443197	0.015973709	0.994479474	d__Bacteria	p__Actinobacteriota	c__Coriobacteriia	o__Coriobacteriales	f__Coriobacteriaceae	g__Collinsella	s__Collinsella unknown_228
MGYG-HGUT-02736:s__Acutalibacteraceae unknown unknown_34	0.295140892	0.291549091	0.929025913	d__Bacteria	p__Firmicutes_A	c__Clostridia	o__Oscillospirales	f__Acutalibacteraceae	g__Acutalibacteraceae unknown	s__Acutalibacteraceae unknown unknown_34
MGYG-HGUT-02737:s__Prevotella unknown_34	0.222165567	0.310813374	0.891825559	d__Bacteria	p__Bacteroidota	c__Bacteroidia	o__Bacteroidales	f__Bacteroidaceae	g__Prevotella	s__Prevotella unknown_34
MGYG-HGUT-02738:s__Blautia unknown_9	0.716920386	0.101163384	0.979458475	d__Bacteria	p__Firmicutes_A	c__Clostridia	o__Lachnospirales	f__Lachnospiraceae	g__Blautia	s__Blautia unknown_9
MGYG-HGUT-02743:s__Collinsella unknown_233	0.735486215	0.092086192	0.979458475	d__Bacteria	p__Actinobacteriota	c__Coriobacteriia	o__Coriobacteriales	f__Coriobacteriaceae	g__Collinsella	s__Collinsella unknown_233
MGYG-HGUT-02744:s__Collinsella unknown_234	0.530685049	0.169679957	0.968532073	d__Bacteria	p__Actinobacteriota	c__Coriobacteriia	o__Coriobacteriales	f__Coriobacteriaceae	g__Collinsella	s__Collinsella unknown_234

MGYG-HGUT-02745:s__Collinsella unknown_235	0.627871574	0.133345521	0.975671757	d__Bacteria	p__Actinobacteriota	c__Coriobacteriia	o__Coriobacteriales	f__Coriobacteriaceae	g__Collinsella	s__Collinsella unknown_235
MGYG-HGUT-02746:s__Collinsella unknown_236	0.037743365	0.567438792	0.768225834	d__Bacteria	p__Actinobacteriota	c__Coriobacteriia	o__Coriobacteriales	f__Coriobacteriaceae	g__Collinsella	s__Collinsella unknown_236
MGYG-HGUT-02748:s__Collinsella unknown_238	0.156063575	0.387929953	0.891825559	d__Bacteria	p__Actinobacteriota	c__Coriobacteriia	o__Coriobacteriales	f__Coriobacteriaceae	g__Collinsella	s__Collinsella unknown_238
MGYG-HGUT-02750:s__Collinsella unknown_240	0.033751482	0.579551614	0.768225834	d__Bacteria	p__Actinobacteriota	c__Coriobacteriia	o__Coriobacteriales	f__Coriobacteriaceae	g__Collinsella	s__Collinsella unknown_240
MGYG-HGUT-02753:s__CAG-103 unknown_3	0.448772871	0.212035149	0.961292208	d__Bacteria	p__Firmicutes_A	c__Clostridia	o__Oscillospirales	f__Oscillospiraceae	g__CAG-103	s__CAG-103 unknown_3
MGYG-HGUT-02754:s__Collinsella aerofaciens_F_9	0.809765567	0.066630959	0.987142302	d__Bacteria	p__Actinobacteriota	c__Coriobacteriia	o__Coriobacteriales	f__Coriobacteriaceae	g__Collinsella	s__Collinsella aerofaciens_F_9
MGYG-HGUT-02758:s__UBA1381 unknown unknown_4	0.713924893	0.095443735	0.979458475	d__Bacteria	p__Firmicutes_A	c__Clostridia	o__Monoglobales	f__UBA1381	g__UBA1381 unknown	s__UBA1381 unknown unknown_4
MGYG-HGUT-02759:s__Collinsella unknown_245	0.63766924	0.130881678	0.975671757	d__Bacteria	p__Actinobacteriota	c__Coriobacteriia	o__Coriobacteriales	f__Coriobacteriaceae	g__Collinsella	s__Collinsella unknown_245
MGYG-HGUT-02762:s__CAG-485 unknown_11	0.312424796	-0.282873059	0.929025913	d__Bacteria	p__Bacteroidota	c__Bacteroidia	o__Bacteroidales	f__Muribaculaceae	g__CAG-485	s__CAG-485 unknown_11

MGYG-HGUT-02763:s__Collinsella aerofaciens_F_11	0.333688539	0.268820674	0.930624692	d__Bacteria	p__Actinobacteriota	c__Coriobacteriia	o__Coriobacteriales	f__Coriobacteriaceae	g__Collinsella	s__Collinsella aerofaciens_F_11
MGYG-HGUT-02764:s__Collinsella sp000763055_3	0.362545773	0.244487585	0.93970925	d__Bacteria	p__Actinobacteriota	c__Coriobacteriia	o__Coriobacteriales	f__Coriobacteriaceae	g__Collinsella	s__Collinsella sp000763055_3
MGYG-HGUT-02765:s__Collinsella unknown_246	0.483405262	0.195913063	0.968532073	d__Bacteria	p__Actinobacteriota	c__Coriobacteriia	o__Coriobacteriales	f__Coriobacteriaceae	g__Collinsella	s__Collinsella unknown_246
MGYG-HGUT-02766:s__Collinsella unknown_247	0.678202934	0.112293827	0.975671757	d__Bacteria	p__Actinobacteriota	c__Coriobacteriia	o__Coriobacteriales	f__Coriobacteriaceae	g__Collinsella	s__Collinsella unknown_247
MGYG-HGUT-02769:s__CAG-465 unknown unknown_2	0.210002275	0.350404247	0.891825559	d__Bacteria	p__Firmicutes_A	c__Clostridia	o__TANB77	f__CAG-465	g__CAG-465 unknown	s__CAG-465 unknown unknown_2
MGYG-HGUT-02770:s__Catenibacterium unknown_3	0.453634153	-0.207191412	0.96390515	d__Bacteria	p__Firmicutes	c__Bacilli	o__Erysipelotrichales	f__Erysipelatoclostridiaceae	g__Catenibacterium	s__Catenibacterium unknown_3
MGYG-HGUT-02772:s__Faecalicatena unknown_7	0.172216615	0.378780757	0.891825559	d__Bacteria	p__Firmicutes_A	c__Clostridia	o__Lachnospirales	f__Lachnospiraceae	g__Faecalicatena	s__Faecalicatena unknown_7
MGYG-HGUT-02773:s__CAG-245 sp000435175	0.81156123	-0.066386586	0.987142302	d__Bacteria	p__Firmicutes_A	c__Clostridia	o__TANB77	f__CAG-508	g__CAG-245	s__CAG-245 sp000435175
MGYG-HGUT-02776:s__Oscillospiraceae unknown unknown_14	0.134447704	-0.408718877	0.891825559	d__Bacteria	p__Firmicutes_A	c__Clostridia	o__Oscillospirales	f__Oscillospiraceae	g__Oscillospiraceae unknown	s__Oscillospiraceae unknown unknown_14

MGYG-HGUT-02778:s__Collinsella unknown_250	0.346100871	0.258250388	0.939709925	d__Bacteria	p__Actinobacteriota	c__Coriobacteriia	o__Coriobacteriales	f__Coriobacteriaceae	g__Collinsella	s__Collinsella unknown_250
MGYG-HGUT-02779:s__Holdemania sp900120005	0.16429141	-0.382159777	0.891825559	d__Bacteria	p__Firmicutes	c__Bacilli	o__Erysipelotrichales	f__Erysipelotrichaceae	g__Holdemania	s__Holdemania sp900120005
MGYG-HGUT-02780:s__Fournierella unknown_2	0.558278119	-0.160325293	0.968532073	d__Bacteria	p__Firmicutes_A	c__Clostridia	o__Oscillospirales	f__Ruminococcaceae	g__Fournierella	s__Fournierella unknown_2
MGYG-HGUT-02783:s__Prevotellamassilia unknown_7	0.024988587	0.62008115	0.768225834	d__Bacteria	p__Bacteroidota	c__Bacteroidia	o__Bacteroidales	f__Bacteroidaceae	g__Prevotellamassilia	s__Prevotellamassilia unknown_7
MGYG-HGUT-02785:s__Robinsoniella unknown_3	0.547948476	0.168548607	0.968532073	d__Bacteria	p__Firmicutes_A	c__Clostridia	o__Lachnospirales	f__Lachnospiraceae	g__Robinsoniella	s__Robinsoniella unknown_3
MGYG-HGUT-02794:s__CAG-83 sp003487665	0.707402353	-0.102334908	0.979458475	d__Bacteria	p__Firmicutes_A	c__Clostridia	o__Oscillospirales	f__Oscillospiraceae	g__CAG-83	s__CAG-83 sp003487665
MGYG-HGUT-02796:s__CAG-273 sp000435755	0.849859649	0.050973619	0.992878088	d__Bacteria	p__Firmicutes_A	c__Clostridia	o__TANB77	f__CAG-508	g__CAG-273	s__CAG-273 sp000435755
MGYG-HGUT-02797:s__CAG-508 unknown unknown_14	0.216395827	0.345167331	0.891825559	d__Bacteria	p__Firmicutes_A	c__Clostridia	o__TANB77	f__CAG-508	g__CAG-508 unknown	s__CAG-508 unknown unknown_14
MGYG-HGUT-02802:s__UBA1777 unknown_8	0.673115441	0.115691699	0.975671757	d__Bacteria	p__Firmicutes_A	c__Clostridia	o__Oscillospirales	f__Oscillospiraceae	g__UBA1777	s__UBA1777 unknown_8

MGYG-HGUT-02809:s__Faecalibacterium unknown_10	0.000632262	0.925209432	0.648124372	d__Bacteria	p__Firmicutes_A	c__Clostridia	o__Oscillospirales	f__Ruminococcaceae	g__Faecalibacterium	s__Faecalibacterium unknown_10
MGYG-HGUT-02816:s__Citrobacter braakii	0.658868198	0.123316868	0.975671757	d__Bacteria	p__Proteobacteria	c__Gammaproteobacteria	o__Enterobacterales	f__Enterobacteriaceae	g__Citrobacter	s__Citrobacter braakii
MGYG-HGUT-02819:s__Veillonella tobetsuensis	0.982557711	-0.006107119	0.998198197	d__Bacteria	p__Firmicutes_C	c__Negativicutes	o__Veillonellales	f__Veillonellaceae	g__Veillonella	s__Veillonella tobetsuensis
MGYG-HGUT-02823:s__Enterobacter bugandensis	0.414373654	0.222786808	0.93970925	d__Bacteria	p__Proteobacteria	c__Gammaproteobacteria	o__Enterobacterales	f__Enterobacteriaceae	g__Enterobacter	s__Enterobacter bugandensis
MGYG-HGUT-02824:s__Collinsella unknown_253	0.231904051	-0.333753712	0.902181367	d__Bacteria	p__Actinobacteriota	c__Coriobacteriia	o__Coriobacteriales	f__Coriobacteriaceae	g__Collinsella	s__Collinsella unknown_253
MGYG-HGUT-02826:s__Streptococcus unknown_11	0.45097463	0.207434218	0.961292208	d__Bacteria	p__Firmicutes	c__Bacilli	o__Lactobacillales	f__Streptococcaceae	g__Streptococcus	s__Streptococcus unknown_11
MGYG-HGUT-02831:s__CAG-349 sp003539515	0.137438837	-0.409493044	0.891825559	d__Bacteria	p__Firmicutes_A	c__Clostridia	o__4C28d-15	f__CAG-917	g__CAG-349	s__CAG-349 sp003539515
MGYG-HGUT-02832:s__Prevotella unknown_35	0.949671256	0.017503119	0.994479474	d__Bacteria	p__Bacteroidota	c__Bacteroidia	o__Bacteroidales	f__Bacteroidaceae	g__Prevotella	s__Prevotella unknown_35
MGYG-HGUT-02833:s__ER4 unknown_4	0.813687247	0.065848725	0.987142302	d__Bacteria	p__Firmicutes_A	c__Clostridia	o__Oscillospirales	f__Oscillospiraceae	g__ER4	s__ER4 unknown_4

MGYG-HGUT-02834:s__Prevotella unknown_36	0.663430096	0.113109828	0.975671757	d__Bacteria	p__Bacteroidota	c__Bacteroidia	o__Bacteroidales	f__Bacteroidaceae	g__Prevotella	s__Prevotella unknown_36
MGYG-HGUT-02835:s__Fusicatenibacter unknown_1	0.536246655	-0.172597511	0.968532073	d__Bacteria	p__Firmicutes_A	c__Clostridia	o__Lachnospirales	f__Lachnospiraceae	g__Fusicatenibacter	s__Fusicatenibacter unknown_1
MGYG-HGUT-02837:s__Lachnospiraceae unknown unknown_25	0.631787671	0.133271878	0.975671757	d__Bacteria	p__Firmicutes_A	c__Clostridia	o__Lachnospirales	f__Lachnospiraceae	g__Lachnospiraceae unknown	s__Lachnospiraceae unknown unknown_25
MGYG-HGUT-02838:s__Faecalicatena unknown_8	0.567444298	-0.156904198	0.97133903	d__Bacteria	p__Firmicutes_A	c__Clostridia	o__Lachnospirales	f__Lachnospiraceae	g__Faecalicatena	s__Faecalicatena unknown_8
MGYG-HGUT-02839:s__Granulicatella sp001071995	0.882376063	0.041572943	0.994479474	d__Bacteria	p__Firmicutes	c__Bacilli	o__Lactobacillales	f__Aerococcaceae	g__Granulicatella	s__Granulicatella sp001071995
MGYG-HGUT-02840:s__Oscillospiraceae unknown unknown_15	0.742773717	0.090445151	0.979458475	d__Bacteria	p__Firmicutes_A	c__Clostridia	o__Oscillospirales	f__Oscillospiraceae	g__Oscillospiraceae unknown	s__Oscillospiraceae unknown unknown_15
MGYG-HGUT-02843:s__Streptococcus unknown_12	0.234018037	0.332069401	0.902181367	d__Bacteria	p__Firmicutes	c__Bacilli	o__Lactobacillales	f__Streptococcaceae	g__Streptococcus	s__Streptococcus unknown_12
MGYG-HGUT-02844:s__Collinsella unknown_254	0.314998408	0.273277727	0.929025913	d__Bacteria	p__Actinobacteriota	c__Coriobacteriia	o__Coriobacteriales	f__Coriobacteriaceae	g__Collinsella	s__Collinsella unknown_254
MGYG-HGUT-02845:s__Dorea unknown_3	0.074664763	-0.494352407	0.877826247	d__Bacteria	p__Firmicutes_A	c__Clostridia	o__Lachnospirales	f__Lachnospiraceae	g__Dorea	s__Dorea unknown_3

MGYG-HGUT-02848:s__Acutalibacteraceae unknown unknown_36	0.5897954 09	-0.1501086 91	0.9756717 57	d__Bacteria	p__Firmicutes_A	c__Clostridia	o__Oscillospirales	f__Acutalibacteraceae	g__Acutalibacteraceae unknown	s__Acutalibacteraceae unknown unknown_36
MGYG-HGUT-02854:s__Clostridium_U unknown_2	0.1162712 83	-0.4355178 85	0.8833072 56	d__Bacteria	p__Firmicutes_A	c__Clostridia	o__Peptostreptococcales	f__Peptostreptococcaceae	g__Clostridium_U	s__Clostridium_U unknown_2
MGYG-HGUT-02863:s__Agathobaculum desmolans	0.6822216 39	-0.1140898 21	0.9756717 57	d__Bacteria	p__Firmicutes_A	c__Clostridia	o__Oscillospirales	f__Butyricicoccaceae	g__Agathobaculum	s__Agathobaculum desmolans
MGYG-HGUT-02864:s__Collinsella unknown_265	0.0664264 14	0.4887467 6	0.8664525 71	d__Bacteria	p__Actinobacteriota	c__Coriobacteriia	o__Coriobacteriales	f__Coriobacteriaceae	g__Collinsella	s__Collinsella unknown_265
MGYG-HGUT-02867:s__Alistipes unknown_11	0.0773845 65	0.4765603 75	0.8778262 47	d__Bacteria	p__Bacteroidota	c__Bacteroidia	o__Bacteroidales	f__Rikenellaceae	g__Alistipes	s__Alistipes unknown_11
MGYG-HGUT-02869:s__Collinsella unknown_267	0.7579538 8	0.0862740 09	0.9794584 75	d__Bacteria	p__Actinobacteriota	c__Coriobacteriia	o__Coriobacteriales	f__Coriobacteriaceae	g__Collinsella	s__Collinsella unknown_267
MGYG-HGUT-02871:s__Collinsella unknown_268	0.0619520 82	0.5162294 23	0.8572113 2	d__Bacteria	p__Actinobacteriota	c__Coriobacteriia	o__Coriobacteriales	f__Coriobacteriaceae	g__Collinsella	s__Collinsella unknown_268
MGYG-HGUT-02872:s__CAG-272 unknown unknown_18	0.5616566 19	0.1600599 62	0.9713390 3	d__Bacteria	p__Firmicutes_A	c__Clostridia	o__Oscillospirales	f__CAG-272	g__CAG-272 unknown	s__CAG-272 unknown unknown_18
MGYG-HGUT-02874:s__UBA1777 sp002320035	0.3088580 23	0.2800731 2	0.9290259 13	d__Bacteria	p__Firmicutes_A	c__Clostridia	o__Oscillospirales	f__Oscillospiraceae	g__UBA1777	s__UBA1777 sp002320035

MGYG-HGUT-02876:s__CAG-145 sp000435715	0.495161406	-0.183538633	0.968532073	d__Bacteria	p__Firmicutes_A	c__Clostridia	o__Peptostreptococcales	f__Anaerovoraceae	g__CAG-145	s__CAG-145 sp000435715
MGYG-HGUT-02878:s__RC9 unknown_8	0.925742803	-0.0260456	0.994479474	d__Bacteria	p__Bacteroidota	c__Bacteroidia	o__Bacteroidales	f__UBA932	g__RC9	s__RC9 unknown_8
MGYG-HGUT-02880:s__Ruminococcus_D unknown_4	0.173954707	-0.378852512	0.891825559	d__Bacteria	p__Firmicutes_A	c__Clostridia	o__Oscillospirales	f__Ruminococcaceae	g__Ruminococcus_D	s__Ruminococcus_D unknown_4
MGYG-HGUT-02882:s__CAG-83 sp000435975	0.098846612	0.440825155	0.883307256	d__Bacteria	p__Firmicutes_A	c__Clostridia	o__Oscillospirales	f__Oscillospiraceae	g__CAG-83	s__CAG-83 sp000435975
MGYG-HGUT-02883:s__Gastranaerophilales unknown unknown unknown_3	0.210215997	0.348543758	0.891825559	d__Bacteria	p__Cyanobacteria	c__Vampirovibrionia	o__Gastranaerophilales	f__Gastranaerophilales unknown	g__Gastranaerophilales unknown unknown	s__Gastranaerophilales unknown unknown unknown_3
MGYG-HGUT-02905:s__CAG-279 unknown_5	0.177591632	-0.374318024	0.891825559	d__Bacteria	p__Bacteroidota	c__Bacteroidia	o__Bacteroidales	f__Muribaculaceae	g__CAG-279	s__CAG-279 unknown_5
MGYG-HGUT-02906:s__UBA2856 unknown_1	0.785072778	-0.07668082	0.981194211	d__Bacteria	p__Firmicutes_A	c__Clostridia	o__Lachnospirales	f__Lachnospiraceae	g__UBA2856	s__UBA2856 unknown_1
MGYG-HGUT-02911:s__Collinsella unknown_269	0.16669787	0.379818064	0.891825559	d__Bacteria	p__Actinobacteriota	c__Coriobacteriia	o__Coriobacteriales	f__Coriobacteriaceae	g__Collinsella	s__Collinsella unknown_269
MGYG-HGUT-02913:s__CAG-382 unknown unknown_5	0.69496	0.10874	0.97942	d__Bact	p__Firmicutes_A	c__Clostridia	o__Oscillospirales	f__CAG-382	g__CAG-382 unknown	s__CAG-382 unknown unknown_5

	8071	1469	2669	eria						
MGYG-HGUT-02917:s__Collinsella unknown_272	0.966977854	-0.011229356	0.994479474	d__Bacteria	p__Actinobacteriota	c__Coriobacteriia	o__Coriobacteriales	f__Coriobacteriaceae	g__Collinsella	s__Collinsella unknown_272
MGYG-HGUT-02918:s__Collinsella unknown_273	0.305446537	0.284000803	0.929025913	d__Bacteria	p__Actinobacteriota	c__Coriobacteriia	o__Coriobacteriales	f__Coriobacteriaceae	g__Collinsella	s__Collinsella unknown_273
MGYG-HGUT-02920:s__CAG-485 unknown_12	0.316233024	0.276677253	0.929025913	d__Bacteria	p__Bacteroidota	c__Bacteroidia	o__Bacteroidales	f__Muribaculaceae	g__CAG-485	s__CAG-485 unknown_12
MGYG-HGUT-02921:s__Collinsella unknown_274	0.949883118	0.01737673	0.994479474	d__Bacteria	p__Actinobacteriota	c__Coriobacteriia	o__Coriobacteriales	f__Coriobacteriaceae	g__Collinsella	s__Collinsella unknown_274
MGYG-HGUT-02925:s__CAG-877 unknown_6	0.525400692	0.177677434	0.968532073	d__Bacteria	p__Firmicutes	c__Bacilli	o__RF39	f__CAG-611	g__CAG-877	s__CAG-877 unknown_6
MGYG-HGUT-02926:s__CAG-83 sp000431575	0.120113969	0.431181258	0.883307256	d__Bacteria	p__Firmicutes_A	c__Clostridia	o__Oscillospirales	f__Oscillospiraceae	g__CAG-83	s__CAG-83 sp000431575
MGYG-HGUT-02929:s__Collinsella unknown_278	0.025042805	0.618631904	0.768225834	d__Bacteria	p__Actinobacteriota	c__Coriobacteriia	o__Coriobacteriales	f__Coriobacteriaceae	g__Collinsella	s__Collinsella unknown_278
MGYG-HGUT-02930:s__Prevotellamassilia unknown_8	0.67531156	0.116906564	0.975671757	d__Bacteria	p__Bacteroidota	c__Bacteroidia	o__Bacteroidales	f__Bacteroidaceae	g__Prevotellamassilia	s__Prevotellamassilia unknown_8
MGYG-HGUT-02932:s__Collinsella unknown_279	0.032420447	0.574977304	0.768225834	d__Bacteria	p__Actinobacteriota	c__Coriobacteriia	o__Coriobacteriales	f__Coriobacteriaceae	g__Collinsella	s__Collinsella unknown_279

MGYG-HGUT-02933:s__Bacteroides_A unknown_12	0.527490202	0.1719033325	0.9685320073	d__Bacteria	p__Bacteroidota	c__Bacteroidia	o__Bacteroidales	f__Bacteroidaceae	g__Bacteroides_A	s__Bacteroides_A unknown_12
MGYG-HGUT-02935:s__Bacteroides_A sp002161765	0.412574558	0.222626621	0.939709295	d__Bacteria	p__Bacteroidota	c__Bacteroidia	o__Bacteroidales	f__Bacteroidaceae	g__Bacteroides_A	s__Bacteroides_A sp002161765
MGYG-HGUT-02938:s__Clostridium_B unknown	0.425914209	-0.218444642	0.9483970024	d__Bacteria	p__Firmicutes_A	c__Clostridia	o__Clostridiales	f__Clostridiaceae	g__Clostridium_B	s__Clostridium_B unknown
MGYG-HGUT-02941:s__Actinomyces graevenitzii_1	0.6907371 31	0.1092906664	0.9757685679	d__Bacteria	p__Actinobacteriota	c__Actinobacteria	o__Actinomycetales	f__Actinomycetaceae	g__Actinomyces	s__Actinomyces graevenitzii_1
MGYG-HGUT-02943:s__Actinomyces viscosus	0.7740519 23	0.0776651 29	0.9795204 84	d__Bacteria	p__Actinobacteriota	c__Actinobacteria	o__Actinomycetales	f__Actinomycetaceae	g__Actinomyces	s__Actinomyces viscosus
MGYG-HGUT-02945:s__Bariatricus massiliensis	0.3322470 24	-0.271532335	0.9306246 92	d__Bacteria	p__Firmicutes_A	c__Clostridia	o__Lachnospirales	f__Lachnospiraceae	g__Bariatricus	s__Bariatricus massiliensis
MGYG-HGUT-02946:s__Clostridium_M unknown_13	0.0563496 46	-0.525529381	0.857211 32	d__Bacteria	p__Firmicutes_A	c__Clostridia	o__Lachnospirales	f__Lachnospiraceae	g__Clostridium_M	s__Clostridium_M unknown_13
MGYG-HGUT-02947:s__Collinsella unknown_281	0.0284561 18	0.5939031 35	0.7682258 34	d__Bacteria	p__Actinobacteriota	c__Coriobacteriia	o__Coriobacteriales	f__Coriobacteriaceae	g__Collinsella	s__Collinsella unknown_281
MGYG-HGUT-02949:s__Lachnospiraceae unknown unknown_26	0.4598994 25	0.2073127 76	0.9655746 92	d__Bacteria	p__Firmicutes_A	c__Clostridia	o__Lachnospirales	f__Lachnospiraceae	g__Lachnospiraceae unknown	s__Lachnospiraceae unknown unknown_26

MGYG-HGUT-02954:s__UBA1191 unknown_3	0.001483323	0.852462817	0.648124372	d__Bacteria	p__Firmicutes_A	c__Clostridia	o__Peptostreptococcales	f__Anaerovoraceae	g__UBA1191	s__UBA1191 unknown_3
MGYG-HGUT-02957:s__Eggerthellaceae unknown unknown_10	0.356878573	0.256936952	0.93970925	d__Bacteria	p__Actinobacteriota	c__Coriobacteriia	o__Coriobacteriales	f__Eggerthellaceae	g__Eggerthellaceae unknown	s__Eggerthellaceae unknown unknown_10
MGYG-HGUT-02960:s__Prevotella unknown_38	0.503209848	0.181735862	0.968532073	d__Bacteria	p__Bacteroidota	c__Bacteroidia	o__Bacteroidales	f__Bacteroidaceae	g__Prevotella	s__Prevotella unknown_38
MGYG-HGUT-02962:s__Clostridium unknown_5	0.923953963	-0.02679363	0.994479474	d__Bacteria	p__Firmicutes_A	c__Clostridia	o__Clostridiales	f__Clostridiaceae	g__Clostridium	s__Clostridium unknown_5
MGYG-HGUT-02966:s__Blautia_A unknown_15	0.432381499	-0.218944294	0.950806594	d__Bacteria	p__Firmicutes_A	c__Clostridia	o__Lachnospirales	f__Lachnospiraceae	g__Blautia_A	s__Blautia_A unknown_15
MGYG-HGUT-02968:s__Collinsella unknown_285	0.103546264	0.45235595	0.883307256	d__Bacteria	p__Actinobacteriota	c__Coriobacteriia	o__Coriobacteriales	f__Coriobacteriaceae	g__Collinsella	s__Collinsella unknown_285
MGYG-HGUT-02973:s__UBA7096 unknown_5	0.418583415	0.223713914	0.93970925	d__Bacteria	p__Firmicutes_A	c__Clostridia	o__Lachnospirales	f__Lachnospiraceae	g__UBA7096	s__UBA7096 unknown_5
MGYG-HGUT-02978:s__Collinsella unknown_288	0.246146681	0.323798109	0.902181367	d__Bacteria	p__Actinobacteriota	c__Coriobacteriia	o__Coriobacteriales	f__Coriobacteriaceae	g__Collinsella	s__Collinsella unknown_288
MGYG-HGUT-02982:s__Streptococcus unknown_14	0.432548953	0.217334882	0.950806594	d__Bacteria	p__Firmicutes	c__Bacilli	o__Lactobacillales	f__Streptococcaceae	g__Streptococcus	s__Streptococcus unknown_14

MGYG-HGUT-02985:s__Blautia_A unknown_16	0.5360687 79	0.1707061 3	0.9685320 73	d__Bacteria	p__Firmicutes_A	c__Clostridia	o__Lachnospirales	f__Lachnospiraceae	g__Blautia_A	s__Blautia_A unknown_16
MGYG-HGUT-02986:s__Gemella sanguinis	0.7296774 83	0.0942210 19	0.9794584 75	d__Bacteria	p__Firmicutes	c__Bacilli	o__Staphylococcales	f__Gemellaceae	g__Gemella	s__Gemella sanguinis
MGYG-HGUT-02988:s__Pauljensenia unknown_6	0.3320415 8	0.2711862 61	0.9306246 92	d__Bacteria	p__Actinobacteriota	c__Actinobacteria	o__Actinomycetales	f__Actinomycetaceae	g__Pauljensenia	s__Pauljensenia unknown_6
MGYG-HGUT-02989:s__Marvinbryantia unknown_3	0.9947044 06	0.0018218 02	0.9988033 46	d__Bacteria	p__Firmicutes_A	c__Clostridia	o__Lachnospirales	f__Lachnospiraceae	g__Marvinbryantia	s__Marvinbryantia unknown_3
MGYG-HGUT-02990:s__Allisonella histaminiformans	0.8595312 82	-0.0492457 71	0.9928780 88	d__Bacteria	p__Firmicutes_C	c__Negativicutes	o__Veillonellales	f__Dialisteraceae	g__Allisonella	s__Allisonella histaminiformans
MGYG-HGUT-02991:s__Collinsella unknown_289	0.7165710 23	-0.1019048 6	0.9794584 75	d__Bacteria	p__Actinobacteriota	c__Coriobacteriia	o__Coriobacteriales	f__Coriobacteriaceae	g__Collinsella	s__Collinsella unknown_289
MGYG-HGUT-02992:s__Dorea unknown_4	0.2785739 01	0.2845176 3	0.9290259 13	d__Bacteria	p__Firmicutes_A	c__Clostridia	o__Lachnospirales	f__Lachnospiraceae	g__Dorea	s__Dorea unknown_4
MGYG-HGUT-02993:s__CAG-170 sp000432135	0.9983497 92	-0.0005507 96	0.9988437 63	d__Bacteria	p__Firmicutes_A	c__Clostridia	o__Oscillospirales	f__Oscillospiraceae	g__CAG-170	s__CAG-170 sp000432135
MGYG-HGUT-02995:s__Clostridium_M unknown_14	0.4762144 41	-0.1979 2	0.9685320 73	d__Bacteria	p__Firmicutes_A	c__Clostridia	o__Lachnospirales	f__Lachnospiraceae	g__Clostridium_M	s__Clostridium_M unknown_14

		1017								
MGYG-HGUT-03001:s__Stomatobaculum longum	0.8641556	0.046958013	0.994479474	d__Bacteria	p__Firmicutes_A	c__Clostridia	o__Lachnospirales	f__Lachnospiraceae	g__Stomatobaculum	s__Stomatobaculum longum
MGYG-HGUT-03002:s__Streptococcus unknown_15	0.616603843	-0.137469382	0.975671757	d__Bacteria	p__Firmicutes	c__Bacilli	o__Lactobacillales	f__Streptococcaceae	g__Streptococcus	s__Streptococcus unknown_15
MGYG-HGUT-03004:s__Anaeromassilibacillus sp001305115	0.313263443	0.276784749	0.929025913	d__Bacteria	p__Firmicutes_A	c__Clostridia	o__Oscillospirales	f__Acutalibacteraceae	g__Anaeromassilibacillus	s__Anaeromassilibacillus sp001305115
MGYG-HGUT-03005:s__Ruminococcus unknown_6	0.525115203	-0.170571279	0.968532073	d__Bacteria	p__Firmicutes_A	c__Clostridia	o__Oscillospirales	f__Ruminococcaceae	g__Ruminococcus	s__Ruminococcus unknown_6
MGYG-HGUT-03007:s__Collinsella unknown_291	0.158029155	0.380416265	0.891825559	d__Bacteria	p__Actinobacteriota	c__Coriobacteriia	o__Coriobacteriales	f__Coriobacteriaceae	g__Collinsella	s__Collinsella unknown_291
MGYG-HGUT-03010:s__Granulicatella unknown_2	0.67027108	-0.117483479	0.975671757	d__Bacteria	p__Firmicutes	c__Bacilli	o__Lactobacillales	f__Aerococcaceae	g__Granulicatella	s__Granulicatella unknown_2
MGYG-HGUT-03012:s__Blautia_A unknown_17	0.380282226	0.244413366	0.93970925	d__Bacteria	p__Firmicutes_A	c__Clostridia	o__Lachnospirales	f__Lachnospiraceae	g__Blautia_A	s__Blautia_A unknown_17
MGYG-HGUT-03014:s__Actinomyces graevenitzii_2	0.958874967	0.014460885	0.994479474	d__Bacteria	p__Actinobacteriota	c__Actinobacteria	o__Actinomycetales	f__Actinomycetaceae	g__Actinomyces	s__Actinomyces graevenitzii_2
MGYG-HGUT-03015:s__Haemop	0.55103	-0.166	0.96853	d__Bact	p__Proteob	c__Gammaproteo	o__Enterobacterales	f__Pasteurellaceae	g__Haemophilus_D	s__Haemophilus_D unknown_6

hilus_D unknown_6	8922	876624	2073	eria	acteria	bacteria				
MGYG-HGUT-03018:s__Alistipes_A unknown_1	0.552634454	-0.165585873	0.968532073	d__Bacteria	p__Bacteroidota	c__Bacteroidia	o__Bacteroidales	f__Rikenellaceae	g__Alistipes_A	s__Alistipes_A unknown_1
MGYG-HGUT-03020:s__Collinsella unknown_293	0.365294482	0.236628429	0.93970925	d__Bacteria	p__Actinobacteriota	c__Coriobacteriia	o__Coriobacteriales	f__Coriobacteriaceae	g__Collinsella	s__Collinsella unknown_293
MGYG-HGUT-03024:s__Collinsella unknown_294	0.215988468	0.341065209	0.891825559	d__Bacteria	p__Actinobacteriota	c__Coriobacteriia	o__Coriobacteriales	f__Coriobacteriaceae	g__Collinsella	s__Collinsella unknown_294
MGYG-HGUT-03026:s__Collinsella unknown_296	0.056968946	0.524020971	0.85721132	d__Bacteria	p__Actinobacteriota	c__Coriobacteriia	o__Coriobacteriales	f__Coriobacteriaceae	g__Collinsella	s__Collinsella unknown_296
MGYG-HGUT-03027:s__Collinsella unknown_297	0.035446518	0.580965933	0.768225834	d__Bacteria	p__Actinobacteriota	c__Coriobacteriia	o__Coriobacteriales	f__Coriobacteriaceae	g__Collinsella	s__Collinsella unknown_297
MGYG-HGUT-03028:s__Collinsella unknown_298	0.746534637	0.087383129	0.979458475	d__Bacteria	p__Actinobacteriota	c__Coriobacteriia	o__Coriobacteriales	f__Coriobacteriaceae	g__Collinsella	s__Collinsella unknown_298
MGYG-HGUT-03030:s__Collinsella unknown_300	0.919101321	-0.027922237	0.994479474	d__Bacteria	p__Actinobacteriota	c__Coriobacteriia	o__Coriobacteriales	f__Coriobacteriaceae	g__Collinsella	s__Collinsella unknown_300
MGYG-HGUT-03032:s__Collinsella unknown_302	0.181925747	0.369526537	0.891825559	d__Bacteria	p__Actinobacteriota	c__Coriobacteriia	o__Coriobacteriales	f__Coriobacteriaceae	g__Collinsella	s__Collinsella unknown_302
MGYG-HGUT-03034:s__Collinsella unknown_304	0.07757	0.46711	0.87782	d__Bact	p__Actino	c__Coriobacteriia	o__Coriobacteriales	f__Coriobacteriaceae	g__Collinsella	s__Collinsella unknown_304

	639	8039	6247	eria	bacteriota					
MGYG-HGUT-03036:s__Collinsella unknown_305	0.823045989	0.062087586	0.991136622	d__Bacteria	p__Actinobacteriota	c__Coriobacteriia	o__Coriobacteriales	f__Coriobacteriaceae	g__Collinsella	s__Collinsella unknown_305
MGYG-HGUT-03038:s__Collinsella unknown_307	0.6806745	0.114957616	0.9756717157	d__Bacteria	p__Actinobacteriota	c__Coriobacteriia	o__Coriobacteriales	f__Coriobacteriaceae	g__Collinsella	s__Collinsella unknown_307
MGYG-HGUT-03039:s__Collinsella aerofaciens_F_16	0.069215 62	0.48723631	0.8778262 47	d__Bacteria	p__Actinobacteriota	c__Coriobacteriia	o__Coriobacteriales	f__Coriobacteriaceae	g__Collinsella	s__Collinsella aerofaciens_F_16
MGYG-HGUT-03042:s__Gemmiger unknown_11	0.601278903	-0.144809226	0.9756717157	d__Bacteria	p__Firmicutes_A	c__Clostridia	o__Oscillospirales	f__Ruminococcaceae	g__Gemmiger	s__Gemmiger unknown_11
MGYG-HGUT-03044:s__Collinsella unknown_311	0.025679098	0.600287591	0.768225834	d__Bacteria	p__Actinobacteriota	c__Coriobacteriia	o__Coriobacteriales	f__Coriobacteriaceae	g__Collinsella	s__Collinsella unknown_311
MGYG-HGUT-03045:s__Collinsella unknown_312	0.243597278	0.314696354	0.902181367	d__Bacteria	p__Actinobacteriota	c__Coriobacteriia	o__Coriobacteriales	f__Coriobacteriaceae	g__Collinsella	s__Collinsella unknown_312
MGYG-HGUT-03046:s__Collinsella unknown_313	0.5646365 3	0.156849431	0.9713390 3	d__Bacteria	p__Actinobacteriota	c__Coriobacteriia	o__Coriobacteriales	f__Coriobacteriaceae	g__Collinsella	s__Collinsella unknown_313
MGYG-HGUT-03047:s__Collinsella unknown_314	0.209701712	0.341645239	0.891825559	d__Bacteria	p__Actinobacteriota	c__Coriobacteriia	o__Coriobacteriales	f__Coriobacteriaceae	g__Collinsella	s__Collinsella unknown_314
MGYG-HGUT-03052:s__Collinsella unknown_318	0.130453563	0.389047999	0.891825559	d__Bacteria	p__Actinobacteriota	c__Coriobacteriia	o__Coriobacteriales	f__Coriobacteriaceae	g__Collinsella	s__Collinsella unknown_318

MGYG-HGUT-03053:s__Collinsella unknown_319	0.110760736	0.429900243	0.883307256	d__Bacteria	p__Actinobacteriota	c__Coriobacteriia	o__Coriobacteriales	f__Coriobacteriaceae	g__Collinsella	s__Collinsella unknown_319
MGYG-HGUT-03054:s__Eubacterium_R unknown_15	0.224311139	0.333515452	0.891825559	d__Bacteria	p__Firmicutes_A	c__Clostridia	o__Oscillospirales	f__Acutalibacteraceae	g__Eubacterium_R	s__Eubacterium_R unknown_15
MGYG-HGUT-03055:s__Acutalibacteraceae unknown unknown_37	0.362421905	-0.247669445	0.93970925	d__Bacteria	p__Firmicutes_A	c__Clostridia	o__Oscillospirales	f__Acutalibacteraceae	g__Acutalibacteraceae unknown	s__Acutalibacteraceae unknown unknown_37
MGYG-HGUT-03057:s__CAG-110 unknown_22	0.010639598	0.691943094	0.768225834	d__Bacteria	p__Firmicutes_A	c__Clostridia	o__Oscillospirales	f__Oscillospiraceae	g__CAG-110	s__CAG-110 unknown_22
MGYG-HGUT-03062:s__Collinsella unknown_321	0.69282038	0.106278305	0.977938404	d__Bacteria	p__Actinobacteriota	c__Coriobacteriia	o__Coriobacteriales	f__Coriobacteriaceae	g__Collinsella	s__Collinsella unknown_321
MGYG-HGUT-03065:s__Faecalicatena unknown_9	0.317565919	-0.277006751	0.929025913	d__Bacteria	p__Firmicutes_A	c__Clostridia	o__Lachnospirales	f__Lachnospiraceae	g__Faecalicatena	s__Faecalicatena unknown_9
MGYG-HGUT-03066:s__Collinsella unknown_322	0.005497186	0.750154972	0.750056424	d__Bacteria	p__Actinobacteriota	c__Coriobacteriia	o__Coriobacteriales	f__Coriobacteriaceae	g__Collinsella	s__Collinsella unknown_322
MGYG-HGUT-03067:s__Collinsella unknown_323	0.309201309	-0.281207905	0.929025913	d__Bacteria	p__Actinobacteriota	c__Coriobacteriia	o__Coriobacteriales	f__Coriobacteriaceae	g__Collinsella	s__Collinsella unknown_323
MGYG-HGUT-03069:s__Bifidobacterium gallinarum	0.454842476	0.208041717	0.965324827	d__Bacteria	p__Actinobacteriota	c__Actinobacteria	o__Actinomycetales	f__Bifidobacteriaceae	g__Bifidobacterium	s__Bifidobacterium gallinarum

MGYG-HGUT-03071:s__Veillonella unknown_12	0.749726646	-0.089662073	0.979458475	d__Bacteria	p__Firmicutes_C	c__Negativicutes	o__Veillonellales	f__Veillonellaceae	g__Veillonella	s__Veillonella unknown_12
MGYG-HGUT-03074:s__Clostridium_Q sp000435655	0.098982946	0.441782067	0.883307256	d__Bacteria	p__Firmicutes_A	c__Clostridia	o__Lachnospirales	f__Lachnospiraceae	g__Clostridium_Q	s__Clostridium_Q sp000435655
MGYG-HGUT-03075:s__Collinsella unknown_324	0.312458848	0.277647483	0.929025913	d__Bacteria	p__Actinobacteriota	c__Coriobacteriia	o__Coriobacteriales	f__Coriobacteriaceae	g__Collinsella	s__Collinsella unknown_324
MGYG-HGUT-03076:s__Odoribacter unknown_4	0.629987072	-0.134857692	0.975671757	d__Bacteria	p__Bacteroidota	c__Bacteroidia	o__Bacteroidales	f__Marinifilaceae	g__Odoribacter	s__Odoribacter unknown_4
MGYG-HGUT-03078:s__Collinsella unknown_325	0.062021844	0.518810794	0.857211132	d__Bacteria	p__Actinobacteriota	c__Coriobacteriia	o__Coriobacteriales	f__Coriobacteriaceae	g__Collinsella	s__Collinsella unknown_325
MGYG-HGUT-03079:s__Collinsella unknown_326	0.037664267	0.562748647	0.768225834	d__Bacteria	p__Actinobacteriota	c__Coriobacteriia	o__Coriobacteriales	f__Coriobacteriaceae	g__Collinsella	s__Collinsella unknown_326
MGYG-HGUT-03080:s__Collinsella unknown_327	0.214848719	0.346417781	0.891825559	d__Bacteria	p__Actinobacteriota	c__Coriobacteriia	o__Coriobacteriales	f__Coriobacteriaceae	g__Collinsella	s__Collinsella unknown_327
MGYG-HGUT-03082:s__Collinsella unknown_329	0.113677632	0.433624535	0.883307256	d__Bacteria	p__Actinobacteriota	c__Coriobacteriia	o__Coriobacteriales	f__Coriobacteriaceae	g__Collinsella	s__Collinsella unknown_329
MGYG-HGUT-03084:s__CAG-724 unknown_2	0.964418041	0.012502111	0.994479474	d__Bacteria	p__Firmicutes_A	c__Clostridia	o__Oscillospirales	f__CAG-272	g__CAG-724	s__CAG-724 unknown_2

MGYG-HGUT-03091:s__Parabacteroides unknown_12	0.577984917	0.152993561	0.975671757	d__Bacteria	p__Bacteroidota	c__Bacteroidia	o__Bacteroidales	f__Tannerellaceae	g__Parabacteroides	s__Parabacteroides unknown_12
MGYG-HGUT-03094:s__Bacteroides_A barnesiae	0.65599353	-0.124815831	0.975671757	d__Bacteria	p__Bacteroidota	c__Bacteroidia	o__Bacteroidales	f__Bacteroidaceae	g__Bacteroides_A	s__Bacteroides_A barnesiae
MGYG-HGUT-03095:s__CAG-460 unknown_3	0.483633809	-0.194379445	0.968532073	d__Bacteria	p__Firmicutes	c__Bacilli	o__RF39	f__CAG-1000	g__CAG-460	s__CAG-460 unknown_3
MGYG-HGUT-03096:s__Collinsella unknown_332	0.016920088	0.652241704	0.768225834	d__Bacteria	p__Actinobacteriota	c__Coriobacteriia	o__Coriobacteriales	f__Coriobacteriaceae	g__Collinsella	s__Collinsella unknown_332
MGYG-HGUT-03097:s__Bacteroides_A sp000436795	0.878858538	0.042717146	0.994479474	d__Bacteria	p__Bacteroidota	c__Bacteroidia	o__Bacteroidales	f__Bacteroidaceae	g__Bacteroides_A	s__Bacteroides_A sp000436795
MGYG-HGUT-03101:s__Collinsella unknown_333	0.485818377	0.192940482	0.968532073	d__Bacteria	p__Actinobacteriota	c__Coriobacteriia	o__Coriobacteriales	f__Coriobacteriaceae	g__Collinsella	s__Collinsella unknown_333
MGYG-HGUT-03103:s__Collinsella unknown_334	0.187360067	-0.361572676	0.891825559	d__Bacteria	p__Actinobacteriota	c__Coriobacteriia	o__Coriobacteriales	f__Coriobacteriaceae	g__Collinsella	s__Collinsella unknown_334
MGYG-HGUT-03105:s__QAMX01 unknown unknown	0.836000354	0.056808821	0.992775775	d__Bacteria	p__Firmicutes_A	c__Clostridia	o__Oscillospirales	f__QAMX01	g__QAMX01 unknown	s__QAMX01 unknown unknown
MGYG-HGUT-03107:s__Collinsella unknown_335	0.6558468	0.121938819	0.975671757	d__Bacteria	p__Actinobacteriota	c__Coriobacteriia	o__Coriobacteriales	f__Coriobacteriaceae	g__Collinsella	s__Collinsella unknown_335

MGYG-HGUT-03108:s__Collinsella unknown_336	0.687451846	-0.111373202	0.975768579	d__Bacteria	p__Actinobacteriota	c__Coriobacteriia	o__Coriobacteriales	f__Coriobacteriaceae	g__Collinsella	s__Collinsella unknown_336
MGYG-HGUT-03112:s__Ruminococcus_E unknown_4	0.89335152	0.037465848	0.994479474	d__Bacteria	p__Firmicutes_A	c__Clostridia	o__Oscillospirales	f__Acutalibacteraceae	g__Ruminococcus_E	s__Ruminococcus_E unknown_4
MGYG-HGUT-03113:s__Pauljensenia sp001838165	0.024446003	0.616536075	0.768225834	d__Bacteria	p__Actinobacteriota	c__Actinobacteria	o__Actinomycetales	f__Actinomycetaceae	g__Pauljensenia	s__Pauljensenia sp001838165
MGYG-HGUT-03114:s__Clostridium unknown_6	0.223614844	-0.338361298	0.891825559	d__Bacteria	p__Firmicutes_A	c__Clostridia	o__Clostridiales	f__Clostridiaceae	g__Clostridium	s__Clostridium unknown_6
MGYG-HGUT-03134:s__Streptococcus unknown_16	0.34667472	0.262535206	0.93970925	d__Bacteria	p__Firmicutes	c__Bacilli	o__Lactobacillales	f__Streptococcaceae	g__Streptococcus	s__Streptococcus unknown_16
MGYG-HGUT-03136:s__Streptococcus unknown_17	0.409797732	-0.231140777	0.93970925	d__Bacteria	p__Firmicutes	c__Bacilli	o__Lactobacillales	f__Streptococcaceae	g__Streptococcus	s__Streptococcus unknown_17
MGYG-HGUT-03138:s__Streptococcus pseudopneumoniae_O	0.248016168	0.322691206	0.902181367	d__Bacteria	p__Firmicutes	c__Bacilli	o__Lactobacillales	f__Streptococcaceae	g__Streptococcus	s__Streptococcus pseudopneumoniae_O
MGYG-HGUT-03146:s__Streptococcus oralis_S	0.373169119	0.249554314	0.93970925	d__Bacteria	p__Firmicutes	c__Bacilli	o__Lactobacillales	f__Streptococcaceae	g__Streptococcus	s__Streptococcus oralis_S
MGYG-HGUT-03151:s__Blautia_A unknown_18	0.220998889	0.341989925	0.891825559	d__Bacteria	p__Firmicutes_A	c__Clostridia	o__Lachnospirales	f__Lachnospiraceae	g__Blautia_A	s__Blautia_A unknown_18

MGYG-HGUT-03152:s__Prevotella denticola	0.218684149	-0.341734266	0.891825559	d__Bacteria	p__Bacteroidota	c__Bacteroidia	o__Bacteroidales	f__Bacteroidaceae	g__Prevotella	s__Prevotella denticola
MGYG-HGUT-03153:s__RUG420 unknown_1	0.729232725	0.094322652	0.979458475	d__Bacteria	p__Firmicutes_A	c__Clostridia	o__Oscillospirales	f__Acutalibacteraceae	g__RUG420	s__RUG420 unknown_1
MGYG-HGUT-03160:s__Acutalibacteraceae unknown unknown_38	0.212615917	0.347041954	0.891825559	d__Bacteria	p__Firmicutes_A	c__Clostridia	o__Oscillospirales	f__Acutalibacteraceae	g__Acutalibacteraceae unknown	s__Acutalibacteraceae unknown unknown_38
MGYG-HGUT-03163:s__Bacteroides_A unknown_13	0.885191382	0.040572341	0.994479474	d__Bacteria	p__Bacteroidota	c__Bacteroidia	o__Bacteroidales	f__Bacteroidaceae	g__Bacteroides_A	s__Bacteroides_A unknown_13
MGYG-HGUT-03165:s__Coprococcus unknown_3	0.912343552	-0.029896419	0.994479474	d__Bacteria	p__Firmicutes_A	c__Clostridia	o__Lachnospirales	f__Lachnospiraceae	g__Coprococcus	s__Coprococcus unknown_3
MGYG-HGUT-03166:s__Faecalibacterium unknown_11	0.995871473	-0.001441477	0.998803346	d__Bacteria	p__Firmicutes_A	c__Clostridia	o__Oscillospirales	f__Ruminococcaceae	g__Faecalibacterium	s__Faecalibacterium unknown_11
MGYG-HGUT-03167:s__Collinsella unknown_341	0.828668176	0.060317201	0.99222019	d__Bacteria	p__Actinobacteriota	c__Coriobacteriia	o__Coriobacteriales	f__Coriobacteriaceae	g__Collinsella	s__Collinsella unknown_341
MGYG-HGUT-03173:s__Collinsella aerofaciens_F_17	0.670226696	0.116415061	0.975671757	d__Bacteria	p__Actinobacteriota	c__Coriobacteriia	o__Coriobacteriales	f__Coriobacteriaceae	g__Collinsella	s__Collinsella aerofaciens_F_17
MGYG-HGUT-03178:s__Klebsiella quasivariicola	0.204435558	0.327956876	0.891825559	d__Bacteria	p__Proteobacteria	c__Gammaproteobacteria	o__Enterobacterales	f__Enterobacteriaceae	g__Klebsiella	s__Klebsiella quasivariicola

MGYG-HGUT-03181:s__Collinsella unknown_346	0.166475012	0.384787589	0.891825559	d__Bacteria	p__Actinobacteriota	c__Coriobacteriia	o__Coriobacteriales	f__Coriobacteriaceae	g__Collinsella	s__Collinsella unknown_346
MGYG-HGUT-03183:s__Collinsella unknown_347	0.368348691	0.249119375	0.93970925	d__Bacteria	p__Actinobacteriota	c__Coriobacteriia	o__Coriobacteriales	f__Coriobacteriaceae	g__Collinsella	s__Collinsella unknown_347
MGYG-HGUT-03188:s__Veillonella dispar_2	0.887814564	0.038825656	0.994479474	d__Bacteria	p__Firmicutes_C	c__Negativicutes	o__Veillonellales	f__Veillonellaceae	g__Veillonella	s__Veillonella dispar_2
MGYG-HGUT-03190:s__Veillonella rogosae	0.866951653	0.046974271	0.994479474	d__Bacteria	p__Firmicutes_C	c__Negativicutes	o__Veillonellales	f__Veillonellaceae	g__Veillonella	s__Veillonella rogosae
MGYG-HGUT-03192:s__UBA5416 unknown	0.153961301	0.391760147	0.891825559	d__Bacteria	p__Firmicutes_A	c__Clostridia	o__Lachnospirales	f__Lachnospiraceae	g__UBA5416	s__UBA5416 unknown
MGYG-HGUT-03197:s__F0422 unknown_3	0.975466254	-0.008536718	0.996587801	d__Bacteria	p__Firmicutes_C	c__Negativicutes	o__Veillonellales	f__Veillonellaceae	g__F0422	s__F0422 unknown_3
MGYG-HGUT-03206:s__Collinsella unknown_349	0.46268816	0.200439405	0.965574692	d__Bacteria	p__Actinobacteriota	c__Coriobacteriia	o__Coriobacteriales	f__Coriobacteriaceae	g__Collinsella	s__Collinsella unknown_349
MGYG-HGUT-03210:s__Anaerovoracaceae unknown unknown_6	0.823893859	-0.061132786	0.991148931	d__Bacteria	p__Firmicutes_A	c__Clostridia	o__Peptostreptococcales	f__Anaerovoracaceae	g__Anaerovoracaceae unknown	s__Anaerovoracaceae unknown unknown_6
MGYG-HGUT-03211:s__Acutalibacteraceae unknown unknown_40	0.675819425	0.115575041	0.975671757	d__Bacteria	p__Firmicutes_A	c__Clostridia	o__Oscillospirales	f__Acutalibacteraceae	g__Acutalibacteraceae unknown	s__Acutalibacteraceae unknown unknown_40

MGYG-HGUT-03214:s__Lachnospirales unknown unknown unknown_3	0.802324624	0.068710149	0.987142302	d__Bacteria	p__Firmicutes_A	c__Clostridia	o__Lachnospirales	f__Lachnospirales unknown	g__Lachnospirales unknown unknown	s__Lachnospirales unknown unknown unknown_3
MGYG-HGUT-03215:s__Lachnospiraceae unknown unknown_27	0.487015464	-0.191927797	0.968532073	d__Bacteria	p__Firmicutes_A	c__Clostridia	o__Lachnospirales	f__Lachnospiraceae	g__Lachnospiraceae unknown	s__Lachnospiraceae unknown unknown_27
MGYG-HGUT-03221:s__Bacteroidaceae unknown unknown_4	0.5540931 1	0.163951538	0.968532073	d__Bacteria	p__Bacteroidota	c__Bacteroidia	o__Bacteroidales	f__Bacteroidaceae	g__Bacteroidaceae unknown	s__Bacteroidaceae unknown unknown_4
MGYG-HGUT-03222:s__CAG-582 unknown	0.194865732	0.362234107	0.891825559	d__Bacteria	p__Firmicutes	c__Bacilli	o__RF39	f__CAG-1000	g__CAG-582	s__CAG-582 unknown
MGYG-HGUT-03224:s__CAG-74 unknown unknown_16	0.292556616	0.287748371	0.929025913	d__Bacteria	p__Firmicutes_A	c__Clostridia	o__Christensenellales	f__CAG-74	g__CAG-74 unknown	s__CAG-74 unknown unknown_16
MGYG-HGUT-03226:s__QALS01 unknown_3	0.81000857	0.066708723	0.987142302	d__Bacteria	p__Firmicutes_A	c__Clostridia	o__4C28d-15	f__CAG-727	g__QALS01	s__QALS01 unknown_3
MGYG-HGUT-03227:s__Christensenellaceae unknown unknown_4	0.334044859	-0.270878471	0.930624692	d__Bacteria	p__Firmicutes_A	c__Clostridia	o__Christensenellales	f__Christensenellaceae	g__Christensenellaceae unknown	s__Christensenellaceae unknown unknown_4
MGYG-HGUT-03231:s__Collinsella unknown_355	0.622754497	0.135489307	0.975671757	d__Bacteria	p__Actinobacteriota	c__Coriobacteriia	o__Coriobacteriales	f__Coriobacteriaceae	g__Collinsella	s__Collinsella unknown_355
MGYG-HGUT-03232:s__Pseudoflavonifractor unknown	0.515453013	0.182640628	0.968532073	d__Bacteria	p__Firmicutes_A	c__Clostridia	o__Oscillospirales	f__Oscillospiraceae	g__Pseudoflavonifractor	s__Pseudoflavonifractor unknown

MGYG-HGUT-03234:s__Collinsella unknown_356	0.202534126	0.3439013 72	0.8918255 59	d__Bacteria	p__Actinobacteriota	c__Coriobacteriia	o__Coriobacteriales	f__Coriobacteriaceae	g__Collinsella	s__Collinsella unknown_356
MGYG-HGUT-03237:s__Victivallis sp002998355	0.154108 16	0.3874925 83	0.8918255 59	d__Bacteria	p__Verrucomicrobiota	c__Lentisphaeria	o__Victivallales	f__Victivallaceae	g__Victivallis	s__Victivallis sp002998355
MGYG-HGUT-03240:s__Butyricimonas unknown_3	0.9228029 84	0.0268226 59	0.9944794 74	d__Bacteria	p__Bacteroidota	c__Bacteroidia	o__Bacteroidales	f__Marinifilaceae	g__Butyricimonas	s__Butyricimonas unknown_3
MGYG-HGUT-03242:s__Prevotellamassilia sp000437675	0.5737325 62	-0.1567306 22	0.9756717 57	d__Bacteria	p__Bacteroidota	c__Bacteroidia	o__Bacteroidales	f__Bacteroidaceae	g__Prevotellamassilia	s__Prevotellamassilia sp000437675
MGYG-HGUT-03245:s__Faecalicatena unknown_10	0.3817250 39	-0.2436484 31	0.9397092 5	d__Bacteria	p__Firmicutes_A	c__Clostridia	o__Lachnospirales	f__Lachnospiraceae	g__Faecalicatena	s__Faecalicatena unknown_10
MGYG-HGUT-03248:s__CAG-727 unknown unknown_19	0.6296545 23	-0.1301443 09	0.9756717 57	d__Bacteria	p__Firmicutes_A	c__Clostridia	o__4C28d-15	f__CAG-727	g__CAG-727 unknown	s__CAG-727 unknown unknown_19
MGYG-HGUT-03252:s__Bacteroides unknown_8	0.3755407 29	-0.2427435 36	0.9397092 5	d__Bacteria	p__Bacteroidota	c__Bacteroidia	o__Bacteroidales	f__Bacteroidaceae	g__Bacteroides	s__Bacteroides unknown_8
MGYG-HGUT-03263:s__CAG-110 unknown_23	0.4261683 33	-0.2174468 62	0.9483970 24	d__Bacteria	p__Firmicutes_A	c__Clostridia	o__Oscillospirales	f__Oscillospiraceae	g__CAG-110	s__CAG-110 unknown_23
MGYG-HGUT-03266:s__Collinsella unknown_360	0.43016	0.22099	0.95080	d__Bact	p__Actino	c__Coriobacteriia	o__Coriobacteriales	f__Coriobacteriaceae	g__Collinsella	s__Collinsella unknown_360

	0413	9047	6594	eria	bacteriota					
MGYG-HGUT-03268:s__Prevotella unknown_39	0.83345376	0.056835059	0.99222019	d__Bacteria	p__Bacteroidota	c__Bacteroidia	o__Bacteroidales	f__Bacteroidaceae	g__Prevotella	s__Prevotella unknown_39
MGYG-HGUT-03270:s__Muribaculaceae unknown unknown_6	0.7490933	0.088121698	0.979458475	d__Bacteria	p__Bacteroidota	c__Bacteroidia	o__Bacteroidales	f__Muribaculaceae	g__Muribaculaceae unknown	s__Muribaculaceae unknown unknown_6
MGYG-HGUT-03272:s__Collinsella unknown_361	0.953480974	-0.016136674	0.994479474	d__Bacteria	p__Actinobacteriota	c__Coriobacteriia	o__Coriobacteriales	f__Coriobacteriaceae	g__Collinsella	s__Collinsella unknown_361
MGYG-HGUT-03273:s__Lachnospiraceae unknown unknown_28	0.675948019	-0.116717181	0.975671757	d__Bacteria	p__Firmicutes_A	c__Clostridia	o__Lachnospirales	f__Lachnospiraceae	g__Lachnospiraceae unknown	s__Lachnospiraceae unknown unknown_28
MGYG-HGUT-03274:s__Collinsella unknown_362	0.901181188	-0.033313931	0.994479474	d__Bacteria	p__Actinobacteriota	c__Coriobacteriia	o__Coriobacteriales	f__Coriobacteriaceae	g__Collinsella	s__Collinsella unknown_362
MGYG-HGUT-03275:s__Collinsella unknown_363	0.898007117	0.0354741	0.994479474	d__Bacteria	p__Actinobacteriota	c__Coriobacteriia	o__Coriobacteriales	f__Coriobacteriaceae	g__Collinsella	s__Collinsella unknown_363
MGYG-HGUT-03276:s__Collinsella unknown_364	0.375968763	0.247818509	0.93970925	d__Bacteria	p__Actinobacteriota	c__Coriobacteriia	o__Coriobacteriales	f__Coriobacteriaceae	g__Collinsella	s__Collinsella unknown_364
MGYG-HGUT-03279:s__Alistipes unknown_13	0.968786751	-0.010615204	0.994479474	d__Bacteria	p__Bacteroidota	c__Bacteroidia	o__Bacteroidales	f__Rikenellaceae	g__Alistipes	s__Alistipes unknown_13
MGYG-HGUT-03281:s__Collinsella unknown_365	0.50570	0.18621	0.96853	d__Bact	p__Actino	c__Coriobacteriia	o__Coriobacteriales	f__Coriobacteriaceae	g__Collinsella	s__Collinsella unknown_365

	9886	6448	2073	eria	bacteriota					
MGYG-HGUT-03284:s__CAG-269 unknown_21	0.905032024	0.033277096	0.994479474	d__Bacteria	p__Firmicutes_A	c__Clostridia	o__TANB77	f__CAG-508	g__CAG-269	s__CAG-269 unknown_21
MGYG-HGUT-03286:s__Coprobacter unknown_2	0.742293602	0.091539968	0.979458475	d__Bacteria	p__Bacteroidota	c__Bacteroidia	o__Bacteroidales	f__Coprobacteraceae	g__Coprobacter	s__Coprobacter unknown_2
MGYG-HGUT-03287:s__Prevotella melaninogenica	0.421728777	-0.223793423	0.942036655	d__Bacteria	p__Bacteroidota	c__Bacteroidia	o__Bacteroidales	f__Bacteroidaceae	g__Prevotella	s__Prevotella melaninogenica
MGYG-HGUT-03291:s__Faecalibacterium unknown_12	0.119881291	0.431828593	0.883307256	d__Bacteria	p__Firmicutes_A	c__Clostridia	o__Oscillospirales	f__Ruminococcaceae	g__Faecalibacterium	s__Faecalibacterium unknown_12
MGYG-HGUT-03293:s__CAG-110 unknown_25	0.085555218	0.457705283	0.882716064	d__Bacteria	p__Firmicutes_A	c__Clostridia	o__Oscillospirales	f__Oscillospiraceae	g__CAG-110	s__CAG-110 unknown_25
MGYG-HGUT-03294:s__Selenomonas_A unknown	0.026450614	0.607549628	0.768225834	d__Bacteria	p__Firmicutes_C	c__Negativicutes	o__Selenomonadales	f__Selenomonadaceae	g__Selenomonas_A	s__Selenomonas_A unknown
MGYG-HGUT-03297:s__Oscillospiraceae unknown unknown_17	0.413807271	-0.217470531	0.93970925	d__Bacteria	p__Firmicutes_A	c__Clostridia	o__Oscillospirales	f__Oscillospiraceae	g__Oscillospiraceae unknown	s__Oscillospiraceae unknown unknown_17
MGYG-HGUT-03303:s__Senegalimassilia unknown_4	0.729024592	0.096648949	0.979458475	d__Bacteria	p__Actinobacteriota	c__Coriobacteriia	o__Coriobacteriales	f__Eggerthellaceae	g__Senegalimassilia	s__Senegalimassilia unknown_4
MGYG-HGUT-03304:s__QANA01 unknown_3	0.70093	0.10040	0.97945	d__Bact	p__Firmicutes_A	c__Clostridia	o__Christensenellales	f__Christensenellaceae	g__QANA01	s__QANA01 unknown_3

	0118	103	8475	eria						
MGYG-HGUT-03306:s__CAG-448 sp003150135	0.952267025	0.015989939	0.994479474	d__Bacteria	p__Firmicutes_A	c__Clostridia	o__Oscillospirales	f__CAG-272	g__CAG-448	s__CAG-448 sp003150135
MGYG-HGUT-03311:s__Angelakisella unknown_2	0.003178249	0.769491738	0.648124372	d__Bacteria	p__Firmicutes_A	c__Clostridia	o__Oscillospirales	f__Ruminococcaceae	g__Angelakisella	s__Angelakisella unknown_2
MGYG-HGUT-03312:s__Bacteroides unknown_9	0.959729897	0.014152272	0.994479474	d__Bacteria	p__Bacteroidota	c__Bacteroidia	o__Bacteroidales	f__Bacteroidaceae	g__Bacteroides	s__Bacteroides unknown_9
MGYG-HGUT-03315:s__Collinsella unknown_366	0.689505498	-0.111850168	0.975768579	d__Bacteria	p__Actinobacteriota	c__Coriobacteriia	o__Coriobacteriales	f__Coriobacteriaceae	g__Collinsella	s__Collinsella unknown_366
MGYG-HGUT-03316:s__Faecalicatena unknown_11	0.386928228	-0.239366783	0.93970925	d__Bacteria	p__Firmicutes_A	c__Clostridia	o__Lachnospirales	f__Lachnospiraceae	g__Faecalicatena	s__Faecalicatena unknown_11
MGYG-HGUT-03318:s__Duodenibacillus massiliensis	0.957023084	0.014413097	0.994479474	d__Bacteria	p__Proteobacteria	c__Gammaproteobacteria	o__Burkholderiales	f__Burkholderiaceae	g__Duodenibacillus	s__Duodenibacillus massiliensis
MGYG-HGUT-03320:s__Catenibacterium unknown_4	0.808254304	0.067376968	0.987142302	d__Bacteria	p__Firmicutes	c__Bacilli	o__Erysipelotrichales	f__Erysipelatoclostridiaceae	g__Catenibacterium	s__Catenibacterium unknown_4
MGYG-HGUT-03322:s__Collinsella unknown_367	0.633673079	0.12708267	0.975671757	d__Bacteria	p__Actinobacteriota	c__Coriobacteriia	o__Coriobacteriales	f__Coriobacteriaceae	g__Collinsella	s__Collinsella unknown_367
MGYG-HGUT-03323:s__Faecalibacterium unknown_13	0.27617	0.29759	0.92902	d__Bact	p__Firmicutes_A	c__Clostridia	o__Oscillospirales	f__Ruminococcaceae	g__Faecalibacterium	s__Faecalibacterium unknown_13

	8342	469	5913	eria						
MGYG-HGUT-03324:s__Mogibacterium unknown_1	0.957291353	0.015023112	0.994479474	d__Bacteria	p__Firmicutes_A	c__Clostridia	o__Peptostreptococcales	f__Anaerovoracaceae	g__Mogibacterium	s__Mogibacterium unknown_1
MGYG-HGUT-03326:s__Blautia unknown_11	0.795171394	0.072954314	0.982829663	d__Bacteria	p__Firmicutes_A	c__Clostridia	o__Lachnospirales	f__Lachnospiraceae	g__Blautia	s__Blautia unknown_11
MGYG-HGUT-03327:s__Lawsonibacter unknown_9	0.496095431	-0.189073854	0.968532073	d__Bacteria	p__Firmicutes_A	c__Clostridia	o__Oscillospirales	f__Oscillospiraceae	g__Lawsonibacter	s__Lawsonibacter unknown_9
MGYG-HGUT-03331:s__Megasphaera sp001546855	0.59065472	-0.149552299	0.975671757	d__Bacteria	p__Firmicutes_C	c__Negativicutes	o__Veillonellales	f__Megasphaeraceae	g__Megasphaera	s__Megasphaera sp001546855
MGYG-HGUT-03332:s__Prevotella unknown_40	0.578028745	0.143473613	0.975671757	d__Bacteria	p__Bacteroidota	c__Bacteroidia	o__Bacteroidales	f__Bacteroidaceae	g__Prevotella	s__Prevotella unknown_40
MGYG-HGUT-03335:s__GCA-900066135 unknown	0.189053453	0.352270795	0.891825559	d__Bacteria	p__Firmicutes_A	c__Clostridia	o__Lachnospirales	f__Lachnospiraceae	g__GCA-900066135	s__GCA-900066135 unknown
MGYG-HGUT-03336:s__Veillonella dispar_3	0.590892925	0.150405081	0.975671757	d__Bacteria	p__Firmicutes_C	c__Negativicutes	o__Veillonellales	f__Veillonellaceae	g__Veillonella	s__Veillonella dispar_3
MGYG-HGUT-03337:s__Ruminococcaceae unknown unknown_20	0.850120002	-0.052053648	0.992878088	d__Bacteria	p__Firmicutes_A	c__Clostridia	o__Oscillospirales	f__Ruminococcaceae	g__Ruminococcaceae unknown	s__Ruminococcaceae unknown unknown_20
MGYG-HGUT-03339:s__Collinsella unknown_369	0.20185	0.35494	0.89182	d__Bact	p__Actino	c__Coriobacteriia	o__Coriobacteriales	f__Coriobacteriaceae	g__Collinsella	s__Collinsella unknown_369

	8723	7585	5559	eria	bacteriota					
MGYG-HGUT-03340:s__Blautia sp900120295	0.120037166	0.381258874	0.883307256	d__Bacteria	p__Firmicutes_A	c__Clostridia	o__Lachnospirales	f__Lachnospiraceae	g__Blautia	s__Blautia sp900120295
MGYG-HGUT-03341:s__Collinsella unknown_370	0.105785602	0.427749237	0.883307256	d__Bacteria	p__Actinobacteriota	c__Coriobacteriia	o__Coriobacteriales	f__Coriobacteriaceae	g__Collinsella	s__Collinsella unknown_370
MGYG-HGUT-03342:s__Collinsella sp002232035_31	0.627341329	0.136056567	0.975671757	d__Bacteria	p__Actinobacteriota	c__Coriobacteriia	o__Coriobacteriales	f__Coriobacteriaceae	g__Collinsella	s__Collinsella sp002232035_31
MGYG-HGUT-03344:s__Dialister unknown_11	0.817156117	-0.064931447	0.987664539	d__Bacteria	p__Firmicutes_C	c__Negativicutes	o__Veillonellales	f__Dialisteraceae	g__Dialister	s__Dialister unknown_11
MGYG-HGUT-03346:s__Prevotella unknown_41	0.200817998	-0.357646824	0.891825559	d__Bacteria	p__Bacteroidota	c__Bacteroidia	o__Bacteroidales	f__Bacteroidaceae	g__Prevotella	s__Prevotella unknown_41
MGYG-HGUT-03347:s__Prevotella unknown_42	0.411648934	-0.220619866	0.93970925	d__Bacteria	p__Bacteroidota	c__Bacteroidia	o__Bacteroidales	f__Bacteroidaceae	g__Prevotella	s__Prevotella unknown_42
MGYG-HGUT-03348:s__Dialister unknown_12	0.260755969	0.313674958	0.913131421	d__Bacteria	p__Firmicutes_C	c__Negativicutes	o__Veillonellales	f__Dialisteraceae	g__Dialister	s__Dialister unknown_12
MGYG-HGUT-03351:s__Bacteroides unknown_10	0.22919234	-0.336559957	0.902181367	d__Bacteria	p__Bacteroidota	c__Bacteroidia	o__Bacteroidales	f__Bacteroidaceae	g__Bacteroides	s__Bacteroides unknown_10

MGYG-HGUT-03352:s__Parasutterella unknown_3	0.890494596	0.038429192	0.994479474	d__Bacteria	p__Proteobacteria	c__Gammaproteobacteria	o__Burkholderiales	f__Burkholderiaceae	g__Parasutterella	s__Parasutterella unknown_3
MGYG-HGUT-03353:s__Prevotella corporis	0.287459894	0.294398871	0.929025913	d__Bacteria	p__Bacteroidota	c__Bacteroidia	o__Bacteroidales	f__Bacteroidaceae	g__Prevotella	s__Prevotella corporis
MGYG-HGUT-03354:s__Faecalicatena unknown_12	0.497599517	-0.189744114	0.968532073	d__Bacteria	p__Firmicutes_A	c__Clostridia	o__Lachnospirales	f__Lachnospiraceae	g__Faecalicatena	s__Faecalicatena unknown_12
MGYG-HGUT-03355:s__Lachnoclostridium phytofermentans_A	0.087557959	-0.455511165	0.882716064	d__Bacteria	p__Firmicutes_A	c__Clostridia	o__Lachnospirales	f__Lachnospiraceae	g__Lachnoclostridium	s__Lachnoclostridium phytofermentans_A
MGYG-HGUT-03358:s__Kluyvera ascorbata	0.768939296	0.077334549	0.979520484	d__Bacteria	p__Proteobacteria	c__Gammaproteobacteria	o__Enterobacterales	f__Enterobacteriaceae	g__Kluyvera	s__Kluyvera ascorbata
MGYG-HGUT-03361:s__Collinsella unknown_371	0.483637361	0.187892236	0.968532073	d__Bacteria	p__Actinobacteriota	c__Coriobacteriia	o__Coriobacteriales	f__Coriobacteriaceae	g__Collinsella	s__Collinsella unknown_371
MGYG-HGUT-03365:s__Pseudescherichia sp002298805	0.544795464	-0.166510852	0.968532073	d__Bacteria	p__Proteobacteria	c__Gammaproteobacteria	o__Enterobacterales	f__Enterobacteriaceae	g__Pseudescherichia	s__Pseudescherichia sp002298805
MGYG-HGUT-03370:s__Streptococcus mitis_AC	0.834913843	-0.057983625	0.992675341	d__Bacteria	p__Firmicutes	c__Bacilli	o__Lactobacillales	f__Streptococcaceae	g__Streptococcus	s__Streptococcus mitis_AC
MGYG-HGUT-03372:s__Citrobacter werkmanii	0.37504	0.22306	0.93970	d__Bact	p__Proteobacteria	c__Gammaproteo	o__Enterobacterales	f__Enterobacteriaceae	g__Citrobacter	s__Citrobacter werkmanii

	4544	8556	925	eria		bacteria				
MGYG-HGUT-03374:s__Prevotella bivia	0.43693508	-0.217139879	0.951038963	d__Bacteria	p__Bacteroidota	c__Bacteroidia	o__Bacteroidales	f__Bacteroidaceae	g__Prevotella	s__Prevotella bivia
MGYG-HGUT-03383:s__Bifidobacterium scardovii	0.702761543	0.104987002	0.979458475	d__Bacteria	p__Actinobacteriota	c__Actinobacteria	o__Actinomycetales	f__Bifidobacteriaceae	g__Bifidobacterium	s__Bifidobacterium scardovii
MGYG-HGUT-03385:s__Ureaplasma parvum	0.952116718	-0.016600405	0.994479474	d__Bacteria	p__Firmicutes	c__Bacilli	o__Mycoplasmatales	f__Mycoplasmoidaceae	g__Ureaplasma	s__Ureaplasma parvum
MGYG-HGUT-03392:s__Streptococcus mitis	0.060965371	0.51743434	0.85721132	d__Bacteria	p__Firmicutes	c__Bacilli	o__Lactobacillales	f__Streptococcaceae	g__Streptococcus	s__Streptococcus mitis
MGYG-HGUT-03395:s__RIT-PI-d unknown	0.777549829	-0.074717324	0.979520484	d__Bacteria	p__Proteobacteria	c__Gammaproteobacteria	o__Enterobacterales	f__Enterobacteriaceae	g__RIT-PI-d	s__RIT-PI-d unknown
MGYG-HGUT-03397:s__F0422 unknown_4	0.714438425	0.102440854	0.979458475	d__Bacteria	p__Firmicutes_C	c__Negativicutes	o__Veillonellales	f__Veillonellaceae	g__F0422	s__F0422 unknown_4
MGYG-HGUT-03398:s__Helicobacteraceae unknown unknown	0.274319459	-0.301005891	0.929025913	d__Bacteria	p__Campylobacterota	c__Campylobacteria	o__Campylobacterales	f__Helicobacteraceae	g__Helicobacteraceae unknown	s__Helicobacteraceae unknown unknown
MGYG-HGUT-03402:s__Paenibacillus lactis	0.318322155	0.27816093	0.929025913	d__Bacteria	p__Firmicutes_I	c__Bacilli_A	o__Paenibacillales	f__Paenibacillaceae	g__Paenibacillus	s__Paenibacillus lactis

MGYG-HGUT-03409:s__CAG-110 unknown_26	0.18931768	0.351030613	0.891825559	d__Bacteria	p__Firmicutes_A	c__Clostridia	o__Oscillospirales	f__Oscillospiraceae	g__CAG-110	s__CAG-110 unknown_26
MGYG-HGUT-03411:s__CAG-568 sp000434395	0.554831736	-0.162177433	0.968532073	d__Bacteria	p__Firmicutes	c__Bacilli	o__RFN20	f__CAG-288	g__CAG-568	s__CAG-568 sp000434395
MGYG-HGUT-03417:s__CAG-74 unknown unknown_17	0.19311562	0.357774759	0.891825559	d__Bacteria	p__Firmicutes_A	c__Clostridia	o__Christensenellales	f__CAG-74	g__CAG-74 unknown	s__CAG-74 unknown unknown_17
MGYG-HGUT-03418:s__Collinsella unknown_373	0.48031605	0.188676272	0.968532073	d__Bacteria	p__Actinobacteriota	c__Coriobacteriia	o__Coriobacteriales	f__Coriobacteriaceae	g__Collinsella	s__Collinsella unknown_373
MGYG-HGUT-03425:s__CAG-127 unknown_3	0.356083397	0.253988526	0.93970925	d__Bacteria	p__Firmicutes_A	c__Clostridia	o__Lachnospirales	f__Lachnospiraceae	g__CAG-127	s__CAG-127 unknown_3
MGYG-HGUT-03427:s__Lachnospiraceae unknown unknown_30	0.636017067	0.132183406	0.975671757	d__Bacteria	p__Firmicutes_A	c__Clostridia	o__Lachnospirales	f__Lachnospiraceae	g__Lachnospiraceae unknown	s__Lachnospiraceae unknown unknown_30
MGYG-HGUT-03439:s__Phascolarctobacterium faecium	0.764004508	-0.083300521	0.979520484	d__Bacteria	p__Firmicutes_C	c__Negativicutes	o__Acidaminococcales	f__Acidaminococcaceae	g__Phascolarctobacterium	s__Phascolarctobacterium faecium
MGYG-HGUT-03452:s__Bifidobacterium ruminantium	0.667520583	0.115884917	0.975671757	d__Bacteria	p__Actinobacteriota	c__Actinobacteria	o__Actinomycetales	f__Bifidobacteriaceae	g__Bifidobacterium	s__Bifidobacterium ruminantium
MGYG-HGUT-03458:s__CAG-127 unknown_4	0.65947086	0.122112584	0.975671757	d__Bacteria	p__Firmicutes_A	c__Clostridia	o__Lachnospirales	f__Lachnospiraceae	g__CAG-127	s__CAG-127 unknown_4

MGYG-HGUT-03461:s__Prevotella unknown_43	0.49674265 9	0.18375891	0.96853207 3	d__Bacteria	p__Bacteroidota	c__Bacteroidia	o__Bacteroidales	f__Bacteroidaceae	g__Prevotella	s__Prevotella unknown_43
MGYG-HGUT-03462:s__Collinsella unknown_374	0.38747580 1	0.23502491 2	0.93970925	d__Bacteria	p__Actinobacteriota	c__Coriobacteriia	o__Coriobacteriales	f__Coriobacteriaceae	g__Collinsella	s__Collinsella unknown_374
MGYG-HGUT-03465:s__CAG-841 unknown	0.94706017 3	-0.01690453 4	0.99447947 4	d__Bacteria	p__Firmicutes_A	c__Clostridia	o__Oscillospirales	f__CAG-272	g__CAG-841	s__CAG-841 unknown
MGYG-HGUT-03480:s__Prevotella unknown_44	0.27090721 9	-0.29900328 2	0.92540666 1	d__Bacteria	p__Bacteroidota	c__Bacteroidia	o__Bacteroidales	f__Bacteroidaceae	g__Prevotella	s__Prevotella unknown_44
MGYG-HGUT-03481:s__CAG-269 sp003525075	0.26203325 4	-0.30896538 1	0.91313142 1	d__Bacteria	p__Firmicutes_A	c__Clostridia	o__TANB77	f__CAG-508	g__CAG-269	s__CAG-269 sp003525075
MGYG-HGUT-03484:s__RUG806 sp900313475	0.72453010 3	-0.09776901 5	0.97945847 5	d__Bacteria	p__Firmicutes_A	c__Clostridia	o__Oscillospirales	f__Acutalibacteraceae	g__RUG806	s__RUG806 sp900313475
MGYG-HGUT-03486:s__CAG-95 sp000436115	0.01269313 1	0.67708242 2	0.76822583 4	d__Bacteria	p__Firmicutes_A	c__Clostridia	o__Lachnospirales	f__Lachnospiraceae	g__CAG-95	s__CAG-95 sp000436115
MGYG-HGUT-03487:s__Olsenella_B unknown_2	0.95781938 8	0.01439530 7	0.99447947 4	d__Bacteria	p__Actinobacteriota	c__Coriobacteriia	o__Coriobacteriales	f__Atopobiaceae	g__Olsenella_B	s__Olsenella_B unknown_2
MGYG-HGUT-03488:s__CAG-488 unknown_2	0.66686	0.11940	0.97567	d__Bact	p__Firmicutes_A	c__Clostridia	o__Oscillospirales	f__Acutalibacteraceae	g__CAG-488	s__CAG-488 unknown_2

	0643	8195	1757	eria						
MGYG-HGUT-03489:s__Prevotellamassilia unknown_10	0.167906337	-0.383823355	0.891825559	d__Bacteria	p__Bacteroidota	c__Bacteroidia	o__Bacteroidales	f__Bacteroidaceae	g__Prevotellamassilia	s__Prevotellamassilia unknown_10
MGYG-HGUT-03493:s__Prevotella sp002299275	0.634001959	0.128964379	0.975671757	d__Bacteria	p__Bacteroidota	c__Bacteroidia	o__Bacteroidales	f__Bacteroidaceae	g__Prevotella	s__Prevotella sp002299275
MGYG-HGUT-03543:s__Bacteroidaceae unknown unknown_5	0.216303526	-0.338010931	0.891825559	d__Bacteria	p__Bacteroidota	c__Bacteroidia	o__Bacteroidales	f__Bacteroidaceae	g__Bacteroidaceae unknown	s__Bacteroidaceae unknown unknown_5
MGYG-HGUT-03551:s__Zag1 unknown_11	0.112403009	0.412352359	0.883307256	d__Bacteria	p__Cyanobacteria	c__Vampirovibrionia	o__Gastranaerophilales	f__Gastranaerophilaceae	g__Zag1	s__Zag1 unknown_11
MGYG-HGUT-03552:s__CAG-127 unknown_5	0.359213733	-0.250232985	0.93970925	d__Bacteria	p__Firmicutes_A	c__Clostridia	o__Lachnospirales	f__Lachnospiraceae	g__CAG-127	s__CAG-127 unknown_5
MGYG-HGUT-03581:s__Acutalibacteraceae unknown unknown_49	0.552039488	0.16546355	0.968532073	d__Bacteria	p__Firmicutes_A	c__Clostridia	o__Oscillospirales	f__Acutalibacteraceae	g__Acutalibacteraceae unknown	s__Acutalibacteraceae unknown unknown_49
MGYG-HGUT-03583:s__Ruminococcaceae unknown unknown_21	0.721885141	0.096825049	0.979458475	d__Bacteria	p__Firmicutes_A	c__Clostridia	o__Oscillospirales	f__Ruminococcaceae	g__Ruminococcaceae unknown	s__Ruminococcaceae unknown unknown_21
MGYG-HGUT-03589:s__Oscillibacter unknown_19	0.576069628	0.152901188	0.975671757	d__Bacteria	p__Firmicutes_A	c__Clostridia	o__Oscillospirales	f__Oscillospiraceae	g__Oscillibacter	s__Oscillibacter unknown_19
MGYG-HGUT-03593:s__Clostridi	0.93704	-0.022	0.99447	d__Bact	p__Firmicutes_A	c__Clostridia	o__Lachnospirales	f__Lachnospiraceae	g__Clostridium_M	s__Clostridium_M unknown_15

um_M unknown_15	3249	195599	9474	eria						
MGYG-HGUT-03597:s__Oribacterium unknown_5	0.557427958	0.163194513	0.968532073	d__Bacteria	p__Firmicutes_A	c__Clostridia	o__Lachnospirales	f__Lachnospiraceae	g__Oribacterium	s__Oribacterium unknown_5
MGYG-HGUT-03598:s__UBA9502 unknown_7	0.616481566	0.13934809	0.975671757	d__Bacteria	p__Firmicutes_A	c__Clostridia	o__Lachnospirales	f__Lachnospiraceae	g__UBA9502	s__UBA9502 unknown_7
MGYG-HGUT-03613:s__Lachnospiraceae unknown unknown_34	0.757470828	-0.0863053 57	0.979458475	d__Bacteria	p__Firmicutes_A	c__Clostridia	o__Lachnospirales	f__Lachnospiraceae	g__Lachnospiraceae unknown	s__Lachnospiraceae unknown unknown_34
MGYG-HGUT-03626:s__RC9 unknown_17	0.983992144	0.005633391	0.998803346	d__Bacteria	p__Bacteroidota	c__Bacteroidia	o__Bacteroidales	f__UBA932	g__RC9	s__RC9 unknown_17
MGYG-HGUT-03647:s__Anaerotignum unknown_4	0.641851406	-0.130148229	0.975671757	d__Bacteria	p__Firmicutes_A	c__Clostridia	o__Lachnospirales	f__Anaerotignaceae	g__Anaerotignum	s__Anaerotignum unknown_4
MGYG-HGUT-03653:s__Catenibacterium unknown_5	0.451471789	-0.208557597	0.961292208	d__Bacteria	p__Firmicutes	c__Bacilli	o__Erysipelotrichales	f__Erysipelatoclostridiaceae	g__Catenibacterium	s__Catenibacterium unknown_5
MGYG-HGUT-03656:s__UBA1417 sp003531055	0.612704668	0.135395049	0.975671757	d__Bacteria	p__Firmicutes_A	c__Clostridia	o__Oscillospirales	f__Acutalibacteraceae	g__UBA1417	s__UBA1417 sp003531055
MGYG-HGUT-03657:s__Collinsella unknown_375	0.99300911	-0.002461588	0.998803346	d__Bacteria	p__Actinobacteriota	c__Coriobacteriia	o__Coriobacteriales	f__Coriobacteriaceae	g__Collinsella	s__Collinsella unknown_375

MGYG-HGUT-03661:s__Acetatifactor unknown_7	0.407662898	0.231508958	0.939709925	d__Bacteria	p__Firmicutes_A	c__Clostridia	o__Lachnospirales	f__Lachnospiraceae	g__Acetatifactor	s__Acetatifactor unknown_7
MGYG-HGUT-03662:s__Prevotella unknown_51	0.500122132	0.187521348	0.968532073	d__Bacteria	p__Bacteroidota	c__Bacteroidia	o__Bacteroidales	f__Bacteroidaceae	g__Prevotella	s__Prevotella unknown_51
MGYG-HGUT-03664:s__CAG-170 unknown_9	0.857441102	0.046685963	0.992878088	d__Bacteria	p__Firmicutes_A	c__Clostridia	o__Oscillospirales	f__Oscillospiraceae	g__CAG-170	s__CAG-170 unknown_9
MGYG-HGUT-03665:s__CAG-611 unknown_3	0.709891531	-0.103231622	0.979458475	d__Bacteria	p__Firmicutes	c__Bacilli	o__RF39	f__CAG-611	g__CAG-611	s__CAG-611 unknown_3
MGYG-HGUT-03668:s__UBA1777 unknown_9	0.830554388	-0.059891636	0.99222019	d__Bacteria	p__Firmicutes_A	c__Clostridia	o__Oscillospirales	f__Oscillospiraceae	g__UBA1777	s__UBA1777 unknown_9
MGYG-HGUT-03669:s__CAG-508 unknown unknown_18	0.450351973	0.21101941	0.961292208	d__Bacteria	p__Firmicutes_A	c__Clostridia	o__TANB77	f__CAG-508	g__CAG-508 unknown	s__CAG-508 unknown unknown_18
MGYG-HGUT-03673:s__Gemmiger unknown_12	0.101486232	0.443532098	0.883307256	d__Bacteria	p__Firmicutes_A	c__Clostridia	o__Oscillospirales	f__Ruminococcaceae	g__Gemmiger	s__Gemmiger unknown_12
MGYG-HGUT-03674:s__Prevotellamassilia unknown_13	0.19838918	0.358151533	0.891825559	d__Bacteria	p__Bacteroidota	c__Bacteroidia	o__Bacteroidales	f__Bacteroidaceae	g__Prevotellamassilia	s__Prevotellamassilia unknown_13
MGYG-HGUT-03675:s__Faecalibacterium unknown_14	0.179038832	0.372884043	0.891825559	d__Bacteria	p__Firmicutes_A	c__Clostridia	o__Oscillospirales	f__Ruminococcaceae	g__Faecalibacterium	s__Faecalibacterium unknown_14

MGYG-HGUT-03676:s__Fusicatenibacter unknown_2	0.1050113 94	0.4190480 72	0.8833072 56	d__Bacteria	p__Firmicutes_A	c__Clostridia	o__Lachnospirales	f__Lachnospiraceae	g__Fusicatenibacter	s__Fusicatenibacter unknown_2
MGYG-HGUT-03678:s__CAG-95 unknown_4	0.1005821 73	0.4577229 54	0.8833072 56	d__Bacteria	p__Firmicutes_A	c__Clostridia	o__Lachnospirales	f__Lachnospiraceae	g__CAG-95	s__CAG-95 unknown_4
MGYG-HGUT-03680:s__Prevotella sp000433175	0.7351803 63	-0.0944318 24	0.9794584 75	d__Bacteria	p__Bacteroidota	c__Bacteroidia	o__Bacteroidales	f__Bacteroidaceae	g__Prevotella	s__Prevotella sp000433175
MGYG-HGUT-03681:s__Bacteroides stercoris	0.3744453 7	0.2490892 27	0.9397092 5	d__Bacteria	p__Bacteroidota	c__Bacteroidia	o__Bacteroidales	f__Bacteroidaceae	g__Bacteroides	s__Bacteroides stercoris
MGYG-HGUT-03682:s__Ruthenibacterium lactatiformans	0.1622670 68	-0.3836420 27	0.8918255 59	d__Bacteria	p__Firmicutes_A	c__Clostridia	o__Oscillospirales	f__Ruminococcaceae	g__Ruthenibacterium	s__Ruthenibacterium lactatiformans
MGYG-HGUT-03683:s__Bifidobacterium pseudocatenulatum	0.2775111 94	0.2900992 58	0.9290259 13	d__Bacteria	p__Actinobacteriota	c__Actinobacteria	o__Actinomycetales	f__Bifidobacteriaceae	g__Bifidobacterium	s__Bifidobacterium pseudocatenulatum
MGYG-HGUT-03684:s__Eisenbergiella massiliensis	0.3034314 83	-0.2765466 65	0.9290259 13	d__Bacteria	p__Firmicutes_A	c__Clostridia	o__Lachnospirales	f__Lachnospiraceae	g__Eisenbergiella	s__Eisenbergiella massiliensis
MGYG-HGUT-03686:s__ER4 sp000765235	0.1557538 87	0.3870222 85	0.8918255 59	d__Bacteria	p__Firmicutes_A	c__Clostridia	o__Oscillospirales	f__Oscillospiraceae	g__ER4	s__ER4 sp000765235
MGYG-HGUT-03688:s__Collinsella unknown_377	0.4977734 58	0.1901744 22	0.9685320 73	d__Bacteria	p__Actinobacteriota	c__Coriobacteriia	o__Coriobacteriales	f__Coriobacteriaceae	g__Collinsella	s__Collinsella unknown_377

MGYG-HGUT-03689:s__Faecalicoccus unknown_1	0.574773741	-0.156710966	0.975671757	d__Bacteria	p__Firmicutes	c__Bacilli	o__Erysipelotrichales	f__Erysipelotrichaceae	g__Faecalicoccus	s__Faecalicoccus unknown_1
MGYG-HGUT-03691:s__Prevotella disiens	0.690290156	-0.111754458	0.975768579	d__Bacteria	p__Bacteroidota	c__Bacteroidia	o__Bacteroidales	f__Bacteroidaceae	g__Prevotella	s__Prevotella disiens
MGYG-HGUT-03692:s__Collinsella sp002232035_32	0.526993363	-0.177661167	0.968532073	d__Bacteria	p__Actinobacteriota	c__Coriobacteriia	o__Coriobacteriales	f__Coriobacteriaceae	g__Collinsella	s__Collinsella sp002232035_32
MGYG-HGUT-03693:s__Bacteroides_A plebeius_A	0.249183313	-0.322413858	0.902181367	d__Bacteria	p__Bacteroidota	c__Bacteroidia	o__Bacteroidales	f__Bacteroidaceae	g__Bacteroides_A	s__Bacteroides_A plebeius_A
MGYG-HGUT-03694:s__Agathobacter faecis	0.024470046	0.619582782	0.768225834	d__Bacteria	p__Firmicutes_A	c__Clostridia	o__Lachnospirales	f__Lachnospiraceae	g__Agathobacter	s__Agathobacter faecis
MGYG-HGUT-03695:s__Ruminococcus_A sp003011855	0.404781777	0.232738868	0.93970925	d__Bacteria	p__Firmicutes_A	c__Clostridia	o__Lachnospirales	f__Lachnospiraceae	g__Ruminococcus_A	s__Ruminococcus_A sp003011855
MGYG-HGUT-03696:s__Collinsella sp002232035_33	0.468543434	0.196421061	0.968194079	d__Bacteria	p__Actinobacteriota	c__Coriobacteriia	o__Coriobacteriales	f__Coriobacteriaceae	g__Collinsella	s__Collinsella sp002232035_33
MGYG-HGUT-03697:s__Prevotella copri_A	0.965677198	-0.011641781	0.994479474	d__Bacteria	p__Bacteroidota	c__Bacteroidia	o__Bacteroidales	f__Bacteroidaceae	g__Prevotella	s__Prevotella copri_A
MGYG-HGUT-03698:s__Collinsella unknown_378	0.39725	0.23558	0.93970	d__Bact	p__Actino	c__Coriobacteriia	o__Coriobacteriales	f__Coriobacteriaceae	g__Collinsella	s__Collinsella unknown_378

	6742	1899	925	eria	bacteriota					
MGYG-HGUT-03699:s__Holdemanella unknown_3	0.902485044	0.033961069	0.994479474	d__Bacteria	p__Firmicutes	c__Bacilli	o__Erysipelotrichales	f__Erysipelotrichaceae	g__Holdemanella	s__Holdemanella unknown_3
MGYG-HGUT-03700:s__Collinsella unknown_379	0.183683638	0.370587399	0.891825559	d__Bacteria	p__Actinobacteriota	c__Coriobacteriia	o__Coriobacteriales	f__Coriobacteriaceae	g__Collinsella	s__Collinsella unknown_379
MGYG-HGUT-03701:s__Parabacteroides unknown_14	0.71913072	-0.10030464676	0.979458475	d__Bacteria	p__Bacteroidota	c__Bacteroidia	o__Bacteroidales	f__Tannerellaceae	g__Parabacteroides	s__Parabacteroides unknown_14
MGYG-HGUT-03702:s__Eisenbergiella sp900066775	0.036879516	0.57665329	0.768225834	d__Bacteria	p__Firmicutes_A	c__Clostridia	o__Lachnospirales	f__Lachnospiraceae	g__Eisenbergiella	s__Eisenbergiella sp900066775
MGYG-HGUT-03703:s__Collinsella unknown_380	0.122211891	0.421602491	0.885742175	d__Bacteria	p__Actinobacteriota	c__Coriobacteriia	o__Coriobacteriales	f__Coriobacteriaceae	g__Collinsella	s__Collinsella unknown_380
MGYG-HGUT-03706:s__An181 sp002160325	0.623612216	0.137363775	0.975671757	d__Bacteria	p__Firmicutes_A	c__Clostridia	o__Lachnospirales	f__Lachnospiraceae	g__An181	s__An181 sp002160325
MGYG-HGUT-03709:s__Enterococcus_A devriesei	0.396433926	-0.233176044	0.93970925	d__Bacteria	p__Firmicutes	c__Bacilli	o__Lactobacillales	f__Enterococcaceae	g__Enterococcus_A	s__Enterococcus_A devriesei
MGYG-HGUT-03715:s__Faecalicatena unknown_13	0.091574198	0.460963631	0.883307256	d__Bacteria	p__Firmicutes_A	c__Clostridia	o__Lachnospirales	f__Lachnospiraceae	g__Faecalicatena	s__Faecalicatena unknown_13
MGYG-HGUT-03717:s__Streptococcus unknown_18	0.01684	0.65611	0.76822	d__Bact	p__Firmicutes	c__Bacilli	o__Lactobacillales	f__Streptococcaceae	g__Streptococcus	s__Streptococcus unknown_18

	9286	1772	5834	eria						
MGYG-HGUT-03718:s__S5-A14a unknown_1	0.249299813	0.322164215	0.902181367	d__Bacteria	p__Firmicutes_A	c__Clostridia	o__Peptostreptococcales	f__Anaerovoraceae	g__S5-A14a	s__S5-A14a unknown_1
MGYG-HGUT-03739:s__Streptococcus oralis_E	0.961709514	-0.013382473	0.994479474	d__Bacteria	p__Firmicutes	c__Bacilli	o__Lactobacillales	f__Streptococcaceae	g__Streptococcus	s__Streptococcus oralis_E
MGYG-HGUT-03741:s__Levyella massiliensis	0.893035893	-0.037563925	0.994479474	d__Bacteria	p__Firmicutes_A	c__Clostridia	o__Tissierellales	f__Helcococcaceae	g__Levyella	s__Levyella massiliensis
MGYG-HGUT-03752:s__CAG-180 unknown_7	0.712684212	0.103236893	0.979458475	d__Bacteria	p__Firmicutes_A	c__Clostridia	o__Oscillospirales	f__Acutalibacteraceae	g__CAG-180	s__CAG-180 unknown_7
MGYG-HGUT-03763:s__Prevotella unknown_52	0.777866952	0.079226168	0.979520484	d__Bacteria	p__Bacteroidota	c__Bacteroidia	o__Bacteroidales	f__Bacteroidaceae	g__Prevotella	s__Prevotella unknown_52
MGYG-HGUT-03765:s__Prevotella bergensis	0.471364773	0.202155724	0.968194079	d__Bacteria	p__Bacteroidota	c__Bacteroidia	o__Bacteroidales	f__Bacteroidaceae	g__Prevotella	s__Prevotella bergensis
MGYG-HGUT-03766:s__Mahellia unknown unknown unknown unknown	0.654373802	-0.125819741	0.975671757	d__Bacteria	p__Firmicutes_A	c__Mahellia	o__Mahellia unknown	f__Mahellia unknown unknown	g__Mahellia unknown unknown unknown	s__Mahellia unknown unknown unknown unknown
MGYG-HGUT-03768:s__Acutalibacteraceae unknown unknown_52	0.459119277	-0.207619536	0.965574692	d__Bacteria	p__Firmicutes_A	c__Clostridia	o__Oscillospirales	f__Acutalibacteraceae	g__Acutalibacteraceae unknown	s__Acutalibacteraceae unknown unknown_52

MGYG-HGUT-03770:s__Flavonifractor unknown_4	0.853625075	0.049705802	0.992878088	d__Bacteria	p__Firmicutes_A	c__Clostridia	o__Oscillospirales	f__Oscillospiraceae	g__Flavonifractor	s__Flavonifractor unknown_4
MGYG-HGUT-03771:s__Blautia sp002161285	0.548501378	-0.168563323	0.968532073	d__Bacteria	p__Firmicutes_A	c__Clostridia	o__Lachnospirales	f__Lachnospiraceae	g__Blautia	s__Blautia sp002161285
MGYG-HGUT-03778:s__Ezakiella unknown_3	0.885268308	0.040076227	0.994479474	d__Bacteria	p__Firmicutes_A	c__Clostridia	o__Tissierellales	f__Ezakiellaceae	g__Ezakiella	s__Ezakiella unknown_3
MGYG-HGUT-03786:s__Stomatobaculum naviforme	0.063584424	0.515273407	0.85721132	d__Bacteria	p__Firmicutes_A	c__Clostridia	o__Lachnospirales	f__Lachnospiraceae	g__Stomatobaculum	s__Stomatobaculum naviforme
MGYG-HGUT-03797:s__Streptococcus unknown_19	0.805427348	0.069151994	0.987142302	d__Bacteria	p__Firmicutes	c__Bacilli	o__Lactobacillales	f__Streptococcaceae	g__Streptococcus	s__Streptococcus unknown_19
MGYG-HGUT-03820:s__UBA5446 unknown_9	0.52793475	-0.169966002	0.968532073	d__Bacteria	p__Firmicutes_A	c__Clostridia	o__Oscillospirales	f__Oscillospiraceae	g__UBA5446	s__UBA5446 unknown_9
MGYG-HGUT-03821:s__Blautia_A unknown_19	0.469102006	0.201224814	0.968194079	d__Bacteria	p__Firmicutes_A	c__Clostridia	o__Lachnospirales	f__Lachnospiraceae	g__Blautia_A	s__Blautia_A unknown_19
MGYG-HGUT-03822:s__Blautia_A unknown_20	0.434292582	0.219449937	0.950806594	d__Bacteria	p__Firmicutes_A	c__Clostridia	o__Lachnospirales	f__Lachnospiraceae	g__Blautia_A	s__Blautia_A unknown_20
MGYG-HGUT-03824:s__CAG-110 unknown_31	0.731090998	0.096458863	0.979458475	d__Bacteria	p__Firmicutes_A	c__Clostridia	o__Oscillospirales	f__Oscillospiraceae	g__CAG-110	s__CAG-110 unknown_31

MGYG-HGUT-03825:s__UBA1777 unknown_10	0.018787502	0.641919337	0.768225834	d__Bacteria	p__Firmicutes_A	c__Clostridia	o__Oscillospirales	f__Oscillospiraceae	g__UBA1777	s__UBA1777 unknown_10
MGYG-HGUT-03834:s__Eisenbergiella unknown_7	0.410327536	0.230145521	0.93970925	d__Bacteria	p__Firmicutes_A	c__Clostridia	o__Lachnospirales	f__Lachnospiraceae	g__Eisenbergiella	s__Eisenbergiella unknown_7
MGYG-HGUT-03835:s__UBA7173_A sp001915385	0.143981396	0.40336149	0.891825559	d__Bacteria	p__Bacteroidota	c__Bacteroidia	o__Bacteroidales	f__Muribaculaceae	g__UBA7173_A	s__UBA7173_A sp001915385
MGYG-HGUT-03840:s__CAG-272 unknown unknown_23	0.000866969	0.902815195	0.648124372	d__Bacteria	p__Firmicutes_A	c__Clostridia	o__Oscillospirales	f__CAG-272	g__CAG-272 unknown	s__CAG-272 unknown unknown_23
MGYG-HGUT-03844:s__Ruminiclostridium_E unknown_5	0.201893182	0.349555616	0.891825559	d__Bacteria	p__Firmicutes_A	c__Clostridia	o__Oscillospirales	f__Ruminococcaceae	g__Ruminiclostridium_E	s__Ruminiclostridium_E unknown_5
MGYG-HGUT-03847:s__Collinsella aerofaciens_F_20	0.724398131	0.099086979	0.979458475	d__Bacteria	p__Actinobacteriota	c__Coriobacteriia	o__Coriobacteriales	f__Coriobacteriaceae	g__Collinsella	s__Collinsella aerofaciens_F_20
MGYG-HGUT-03850:s__Oscillospiraceae unknown unknown_20	0.186519547	0.369310668	0.891825559	d__Bacteria	p__Firmicutes_A	c__Clostridia	o__Oscillospirales	f__Oscillospiraceae	g__Oscillospiraceae unknown	s__Oscillospiraceae unknown unknown_20
MGYG-HGUT-03860:s__NK3B98 unknown_2	0.838303664	0.055362729	0.992878088	d__Bacteria	p__Firmicutes_A	c__Clostridia	o__Oscillospirales	f__Oscillospiraceae	g__NK3B98	s__NK3B98 unknown_2
MGYG-HGUT-03863:s__An200 unknown_3	0.524818709	-0.176158714	0.968532073	d__Bacteria	p__Firmicutes_A	c__Clostridia	o__Oscillospirales	f__Acutalibacteraceae	g__An200	s__An200 unknown_3

MGYG-HGUT-03864:s__Collinsella aerofaciens_F_21	0.904103548	-0.0337418620	0.99479474	d__Bacteria	p__Actinobacteriota	c__Coriobacteriia	o__Coriobacteriales	f__Coriobacteriaceae	g__Collinsella	s__Collinsella aerofaciens_F_21
MGYG-HGUT-03866:s__Bacteroides unknown_13	0.91388522	0.030069442	0.99479474	d__Bacteria	p__Bacteroidota	c__Bacteroidia	o__Bacteroidales	f__Bacteroidaceae	g__Bacteroides	s__Bacteroides unknown_13
MGYG-HGUT-03872:s__Clostridium_A unknown	0.719545166	0.098104151	0.979458475	d__Bacteria	p__Firmicutes_A	c__Clostridia	o__Oscillospirales	f__Acutalibacteraceae	g__Clostridium_A	s__Clostridium_A unknown
MGYG-HGUT-03873:s__Collinsella unknown_392	0.156942784	0.380038216	0.891825559	d__Bacteria	p__Actinobacteriota	c__Coriobacteriia	o__Coriobacteriales	f__Coriobacteriaceae	g__Collinsella	s__Collinsella unknown_392
MGYG-HGUT-03874:s__Merdibacter unknown_4	0.732539366	-0.094922265	0.979458475	d__Bacteria	p__Firmicutes	c__Bacilli	o__Erysipelotrichales	f__Erysipelotrichaceae	g__Merdibacter	s__Merdibacter unknown_4
MGYG-HGUT-03875:s__CAG-74 unknown unknown_21	0.888296756	0.038278101	0.99479474	d__Bacteria	p__Firmicutes_A	c__Clostridia	o__Christensenellales	f__CAG-74	g__CAG-74 unknown	s__CAG-74 unknown_21
MGYG-HGUT-03878:s__Prevotella oris	0.343148138	0.252331461	0.93970925	d__Bacteria	p__Bacteroidota	c__Bacteroidia	o__Bacteroidales	f__Bacteroidaceae	g__Prevotella	s__Prevotella oris
MGYG-HGUT-03884:s__CAG-110 unknown_32	0.079102573	0.476637082	0.877826247	d__Bacteria	p__Firmicutes_A	c__Clostridia	o__Oscillospirales	f__Oscillospiraceae	g__CAG-110	s__CAG-110 unknown_32
MGYG-HGUT-03885:s__Collinsella unknown_393	0.18294301	0.362891385	0.891825559	d__Bacteria	p__Actinobacteriota	c__Coriobacteriia	o__Coriobacteriales	f__Coriobacteriaceae	g__Collinsella	s__Collinsella unknown_393

MGYG-HGUT-03886:s__CAG-1427 unknown_18	0.936175442	0.022522713	0.994479474	d__Bacteria	p__Actinobacteriota	c__Coriobacteriia	o__Coriobacteriales	f__Eggerthellaceae	g__CAG-1427	s__CAG-1427 unknown_18
MGYG-HGUT-03887:s__Acutalibacteraceae unknown unknown_56	0.864815738	0.047776718	0.994479474	d__Bacteria	p__Firmicutes_A	c__Clostridia	o__Oscillospirales	f__Acutalibacteraceae	g__Acutalibacteraceae unknown	s__Acutalibacteraceae unknown unknown_56
MGYG-HGUT-03891:s__UBA11524 sp000437595	0.632935572	0.134017425	0.975671757	d__Bacteria	p__Firmicutes_A	c__Clostridia	o__Christensenellales	f__CAG-74	g__UBA11524	s__UBA11524 sp000437595
MGYG-HGUT-03892:s__Roseburia unknown_7	0.510960522	0.182128677	0.968532073	d__Bacteria	p__Firmicutes_A	c__Clostridia	o__Lachnospirales	f__Lachnospiraceae	g__Roseburia	s__Roseburia unknown_7
MGYG-HGUT-03893:s__Prevotella multisaccharivorax	0.904422928	0.033316759	0.994479474	d__Bacteria	p__Bacteroidota	c__Bacteroidia	o__Bacteroidales	f__Bacteroidaceae	g__Prevotella	s__Prevotella multisaccharivorax
MGYG-HGUT-03898:s__S5-A14a unknown_2	0.033328651	0.568868761	0.768225834	d__Bacteria	p__Firmicutes_A	c__Clostridia	o__Peptostreptococcales	f__Anaerovoraceae	g__S5-A14a	s__S5-A14a unknown_2
MGYG-HGUT-03899:s__Faecalibacterium unknown_15	0.404864284	0.231882446	0.93970925	d__Bacteria	p__Firmicutes_A	c__Clostridia	o__Oscillospirales	f__Ruminococcaceae	g__Faecalibacterium	s__Faecalibacterium unknown_15
MGYG-HGUT-03900:s__Collinsella unknown_396	0.897889683	-0.035270995	0.994479474	d__Bacteria	p__Actinobacteriota	c__Coriobacteriia	o__Coriobacteriales	f__Coriobacteriaceae	g__Collinsella	s__Collinsella unknown_396
MGYG-HGUT-03903:s__Lawsonibacter unknown_10	0.514535976	-0.177369367	0.968532073	d__Bacteria	p__Firmicutes_A	c__Clostridia	o__Oscillospirales	f__Oscillospiraceae	g__Lawsonibacter	s__Lawsonibacter unknown_10

MGYG-HGUT-03906:s__Collinsella sp002232035_34	0.778151812	0.077155594	0.979520484	d__Bacteria	p__Actinobacteriota	c__Coriobacteriia	o__Coriobacteriales	f__Coriobacteriaceae	g__Collinsella	s__Collinsella sp002232035_34
MGYG-HGUT-03911:s__QAMM01 unknown_2	0.470418996	-0.194870913	0.968194079	d__Bacteria	p__Firmicutes_A	c__Clostridia	o__Oscillospirales	f__Ruminococcaceae	g__QAMM01	s__QAMM01 unknown_2
MGYG-HGUT-03912:s__Prevotella sp000435635	0.940963112	-0.019709969	0.994479474	d__Bacteria	p__Bacteroidota	c__Bacteroidia	o__Bacteroidales	f__Bacteroidaceae	g__Prevotella	s__Prevotella sp000435635
MGYG-HGUT-03920:s__Collinsella unknown_400	0.768954242	0.081473615	0.979520484	d__Bacteria	p__Actinobacteriota	c__Coriobacteriia	o__Coriobacteriales	f__Coriobacteriaceae	g__Collinsella	s__Collinsella unknown_400
MGYG-HGUT-03921:s__Gemmiger unknown_13	0.015158193	0.653784325	0.768225834	d__Bacteria	p__Firmicutes_A	c__Clostridia	o__Oscillospirales	f__Ruminococcaceae	g__Gemmiger	s__Gemmiger unknown_13
MGYG-HGUT-03922:s__Bacteroides unknown_14	0.586224092	0.149134999	0.975671757	d__Bacteria	p__Bacteroidota	c__Bacteroidia	o__Bacteroidales	f__Bacteroidaceae	g__Bacteroides	s__Bacteroides unknown_14
MGYG-HGUT-03923:s__Collinsella unknown_401	0.369758526	0.249200043	0.939709925	d__Bacteria	p__Actinobacteriota	c__Coriobacteriia	o__Coriobacteriales	f__Coriobacteriaceae	g__Collinsella	s__Collinsella unknown_401
MGYG-HGUT-03925:s__CAG-882 unknown_2	0.196692411	0.336752455	0.891825559	d__Bacteria	p__Firmicutes_A	c__Clostridia	o__Lachnospirales	f__Lachnospiraceae	g__CAG-882	s__CAG-882 unknown_2
MGYG-HGUT-03927:s__Ruminiclostridium_E unknown_6	0.66450029	0.118459525	0.975671757	d__Bacteria	p__Firmicutes_A	c__Clostridia	o__Oscillospirales	f__Ruminococcaceae	g__Ruminiclostridium_E	s__Ruminiclostridium_E unknown_6

MGYG-HGUT-03928:s__Collinsella unknown_402	0.402188797	0.226019634	0.939709925	d__Bacteria	p__Actinobacteriota	c__Coriobacteriia	o__Coriobacteriales	f__Coriobacteriaceae	g__Collinsella	s__Collinsella unknown_402
MGYG-HGUT-03929:s__CAG-590 unknown_4	0.478220079	0.196828706	0.968532073	d__Bacteria	p__Firmicutes_A	c__Clostridia	o__Lachnospirales	f__Lachnospiraceae	g__CAG-590	s__CAG-590 unknown_4
MGYG-HGUT-03932:s__CAG-964 sp000435335	0.843165896	0.055516975	0.992878088	d__Bacteria	p__Firmicutes_A	c__Clostridia	o__Oscillospirales	f__Acutalibacteraceae	g__CAG-964	s__CAG-964 sp000435335
MGYG-HGUT-03937:s__Gemmiger sp003476825	0.075602343	0.490525736	0.877826247	d__Bacteria	p__Firmicutes_A	c__Clostridia	o__Oscillospirales	f__Ruminococcaceae	g__Gemmiger	s__Gemmiger sp003476825
MGYG-HGUT-03940:s__CAG-302 sp000431795	0.366755606	0.249116621	0.939709925	d__Bacteria	p__Firmicutes	c__Bacilli	o__RF39	f__CAG-302	g__CAG-302	s__CAG-302 sp000431795
MGYG-HGUT-03949:s__Eubacterium_F unknown_2	0.584591497	-0.150004439	0.975671757	d__Bacteria	p__Firmicutes_A	c__Clostridia	o__Lachnospirales	f__Lachnospiraceae	g__Eubacterium_F	s__Eubacterium_F unknown_2
MGYG-HGUT-03950:s__NK3B98 unknown_3	0.598742142	0.144884996	0.975671757	d__Bacteria	p__Firmicutes_A	c__Clostridia	o__Oscillospirales	f__Oscillospiraceae	g__NK3B98	s__NK3B98 unknown_3
MGYG-HGUT-03954:s__CAG-303 unknown	0.795130028	-0.072968891	0.9829663	d__Bacteria	p__Firmicutes_A	c__Clostridia	o__Lachnospirales	f__Lachnospiraceae	g__CAG-303	s__CAG-303 unknown
MGYG-HGUT-03957:s__Parabacteroides unknown_15	0.824755714	-0.062260349	0.991148931	d__Bacteria	p__Bacteroidota	c__Bacteroidia	o__Bacteroidales	f__Tannerellaceae	g__Parabacteroides	s__Parabacteroides unknown_15

MGYG-HGUT-03963:s__CAG-272 unknown unknown_25	0.8102723216	-0.0658488883	0.9871423202	d__Bacteria	p__Firmicutes_A	c__Clostridia	o__Oscillospirales	f__CAG-272	g__CAG-272 unknown	s__CAG-272 unknown unknown_25
MGYG-HGUT-03966:s__UBA11512 sp003522145	0.326508603	-0.270226502	0.930624692	d__Bacteria	p__Firmicutes_A	c__Clostridia	o__Oscillospirales	f__CAG-272	g__UBA11512	s__UBA11512 sp003522145
MGYG-HGUT-03967:s__QALW01 unknown unknown_5	0.23471861	0.319474858	0.902181367	d__Bacteria	p__Firmicutes_A	c__Clostridia	o__Christensenellales	f__QALW01	g__QALW01 unknown	s__QALW01 unknown unknown_5
MGYG-HGUT-03970:s__Lachnospiraceae unknown unknown_36	0.111607566	0.437053108	0.883307256	d__Bacteria	p__Firmicutes_A	c__Clostridia	o__Lachnospirales	f__Lachnospiraceae	g__Lachnospiraceae unknown	s__Lachnospiraceae unknown unknown_36
MGYG-HGUT-03971:s__Mogibacterium sp002299625	0.311191202	0.283908774	0.929025913	d__Bacteria	p__Firmicutes_A	c__Clostridia	o__Peptostreptococcales	f__Anaerovoracaceae	g__Mogibacterium	s__Mogibacterium sp002299625
MGYG-HGUT-03975:s__UBA9475 unknown_3	0.679946467	0.112841691	0.975671757	d__Bacteria	p__Firmicutes_A	c__Clostridia	o__Oscillospirales	f__Oscillospiraceae	g__UBA9475	s__UBA9475 unknown_3
MGYG-HGUT-03978:s__UBA1777 unknown_13	0.430658218	0.218291247	0.950806594	d__Bacteria	p__Firmicutes_A	c__Clostridia	o__Oscillospirales	f__Oscillospiraceae	g__UBA1777	s__UBA1777 unknown_13
MGYG-HGUT-03980:s__Oscillospiraceae unknown unknown_21	0.098433477	0.428744292	0.883307256	d__Bacteria	p__Firmicutes_A	c__Clostridia	o__Oscillospirales	f__Oscillospiraceae	g__Oscillospiraceae unknown	s__Oscillospiraceae unknown unknown_21
MGYG-HGUT-03984:s__Faecalicatena unknown_15	0.520158342	0.179754281	0.968532073	d__Bacteria	p__Firmicutes_A	c__Clostridia	o__Lachnospirales	f__Lachnospiraceae	g__Faecalicatena	s__Faecalicatena unknown_15

MGYG-HGUT-03987:s__UBA1691 unknown_4	0.0782744 04	-0.469123038	0.8778262 47	d__Bacteria	p__Firmicutes_A	c__Clostridia	o__Oscillospirales	f__Acutalibacteraceae	g__UBA1691	s__UBA1691 unknown_4
MGYG-HGUT-03989:s__Alistipes sp002161445	0.1940884 24	-0.360192998	0.8918255 59	d__Bacteria	p__Bacteroidota	c__Bacteroidia	o__Bacteroidales	f__Rikenellaceae	g__Alistipes	s__Alistipes sp002161445
MGYG-HGUT-03990:s__Ruminococcus_E sp900314705	0.0329515 93	-0.577610789	0.7682258 34	d__Bacteria	p__Firmicutes_A	c__Clostridia	o__Oscillospirales	f__Acutalibacteraceae	g__Ruminococcus_E	s__Ruminococcus_E sp900314705
MGYG-HGUT-04003:s__Bacteroides unknown_15	0.682153 44	-0.114242433	0.9756717 57	d__Bacteria	p__Bacteroidota	c__Bacteroidia	o__Bacteroidales	f__Bacteroidaceae	g__Bacteroides	s__Bacteroides unknown_15
MGYG-HGUT-04004:s__CAG-83 unknown_13	0.1267427 11	0.4053931 52	0.8918255 59	d__Bacteria	p__Firmicutes_A	c__Clostridia	o__Oscillospirales	f__Oscillospiraceae	g__CAG-83	s__CAG-83 unknown_13
MGYG-HGUT-04005:s__CAG-354 unknown_6	0.9488694 78	0.0172351 78	0.9944794 74	d__Bacteria	p__Firmicutes_A	c__Clostridia	o__TANB77	f__CAG-508	g__CAG-354	s__CAG-354 unknown_6
MGYG-HGUT-04006:s__Alistipes sp000434235	0.5118832 46	0.1838369 45	0.9685320 73	d__Bacteria	p__Bacteroidota	c__Bacteroidia	o__Bacteroidales	f__Rikenellaceae	g__Alistipes	s__Alistipes sp000434235
MGYG-HGUT-04011:s__Collinsella unknown_406	0.3189750 78	0.2775794 36	0.9290259 13	d__Bacteria	p__Actinobacteriota	c__Coriobacteriia	o__Coriobacteriales	f__Coriobacteriaceae	g__Collinsella	s__Collinsella unknown_406
MGYG-HGUT-04015:s__FJAT-21963 unknown	0.77737	0.07946	0.97952	d__Bact	p__Firmicutes	c__Bacilli	o__Bacillales_A	f__Planococcaceae	g__FJAT-21963	s__FJAT-21963 unknown

	3332	5558	0484	eria						
MGYG-HGUT-04016:s__UBA1191 unknown_6	0.528603166	0.172396921	0.968532073	d__Bacteria	p__Firmicutes_A	c__Clostridia	o__Peptostreptococcales	f__Anaerovoraceae	g__UBA1191	s__UBA1191 unknown_6
MGYG-HGUT-04017:s__CAG-74 unknown unknown_22	0.909751597	0.031124154	0.994479474	d__Bacteria	p__Firmicutes_A	c__Clostridia	o__Christensenellales	f__CAG-74	g__CAG-74 unknown	s__CAG-74 unknown unknown_22
MGYG-HGUT-04019:s__Bacteroides massiliensis	0.552748612	0.166555599	0.968532073	d__Bacteria	p__Bacteroidota	c__Bacteroidia	o__Bacteroidales	f__Bacteroidaceae	g__Bacteroides	s__Bacteroides massiliensis
MGYG-HGUT-04022:s__Ruminococcaceae unknown unknown_24	0.415959983	0.225984129	0.93970925	d__Bacteria	p__Firmicutes_A	c__Clostridia	o__Oscillospirales	f__Ruminococcaceae	g__Ruminococcaceae unknown	s__Ruminococcaceae unknown unknown_24
MGYG-HGUT-04028:s__CAG-83 unknown_14	0.404520638	0.227371402	0.93970925	d__Bacteria	p__Firmicutes_A	c__Clostridia	o__Oscillospirales	f__Oscillospiraceae	g__CAG-83	s__CAG-83 unknown_14
MGYG-HGUT-04035:s__CAG-272 unknown unknown_32	0.17611352	0.363435915	0.891825559	d__Bacteria	p__Firmicutes_A	c__Clostridia	o__Oscillospirales	f__CAG-272	g__CAG-272 unknown	s__CAG-272 unknown unknown_32
MGYG-HGUT-04039:s__Lachnospiraceae unknown unknown_37	0.062041109	0.508207075	0.857211132	d__Bacteria	p__Firmicutes_A	c__Clostridia	o__Lachnospirales	f__Lachnospiraceae	g__Lachnospiraceae unknown	s__Lachnospiraceae unknown unknown_37
MGYG-HGUT-04041:s__Eggerthellaceae unknown unknown_12	0.992894918	-0.002468787	0.998803346	d__Bacteria	p__Actinobacteriota	c__Coriobacteriia	o__Coriobacteriales	f__Eggerthellaceae	g__Eggerthellaceae unknown	s__Eggerthellaceae unknown unknown_12
MGYG-HGUT-04044:s__CAG-74 unknown unknown_23	0.216415669	0.336917165	0.891825559	d__Bacteria	p__Firmicutes_A	c__Clostridia	o__Christensenellales	f__CAG-74	g__CAG-74 unknown	s__CAG-74 unknown unknown_23

MGYG-HGUT-04045:s__Lactobacillus unknown	0.513224056	0.179691148	0.968532073	d__Bacteria	p__Firmicutes	c__Bacilli	o__Lactobacillales	f__Lactobacillaceae	g__Lactobacillus	s__Lactobacillus unknown
MGYG-HGUT-04047:s__CAG-269 sp001916035	0.697249897	-0.109332321	0.979458475	d__Bacteria	p__Firmicutes_A	c__Clostridia	o__TANB77	f__CAG-508	g__CAG-269	s__CAG-269 sp001916035
MGYG-HGUT-04055:s__Eubacterium_G unknown_3	0.547393335	-0.168486572	0.968532073	d__Bacteria	p__Firmicutes_A	c__Clostridia	o__Lachnospirales	f__Lachnospiraceae	g__Eubacterium_G	s__Eubacterium_G unknown_3
MGYG-HGUT-04059:s__UBA1777 sp003150355	0.353228134	0.251133552	0.93970925	d__Bacteria	p__Firmicutes_A	c__Clostridia	o__Oscillospirales	f__Oscillospiraceae	g__UBA1777	s__UBA1777 sp003150355
MGYG-HGUT-04060:s__Collinsella unknown_410	0.307418796	0.283060204	0.929025913	d__Bacteria	p__Actinobacteriota	c__Coriobacteriia	o__Coriobacteriales	f__Coriobacteriaceae	g__Collinsella	s__Collinsella unknown_410
MGYG-HGUT-04063:s__Oxalobacter unknown_4	0.782883661	0.076626041	0.981075107	d__Bacteria	p__Proteobacteria	c__Gammaproteobacteria	o__Burkholderiales	f__Burkholderiaceae	g__Oxalobacter	s__Oxalobacter unknown_4
MGYG-HGUT-04066:s__Collinsella unknown_411	0.086916734	0.463611009	0.882716064	d__Bacteria	p__Actinobacteriota	c__Coriobacteriia	o__Coriobacteriales	f__Coriobacteriaceae	g__Collinsella	s__Collinsella unknown_411
MGYG-HGUT-04071:s__CAG-83 unknown_15	0.009495673	0.674158095	0.768225834	d__Bacteria	p__Firmicutes_A	c__Clostridia	o__Oscillospirales	f__Oscillospiraceae	g__CAG-83	s__CAG-83 unknown_15
MGYG-HGUT-04074:s__Oscillospiraceae unknown unknown_22	0.246011104	0.297370762	0.902181367	d__Bacteria	p__Firmicutes_A	c__Clostridia	o__Oscillospirales	f__Oscillospiraceae	g__Oscillospiraceae unknown	s__Oscillospiraceae unknown unknown_22

MGYG-HGUT-04078:s__CAG-273 unknown_3	0.345442066	0.263204006	0.939709925	d__Bacteria	p__Firmicutes_A	c__Clostridia	o__TANB77	f__CAG-508	g__CAG-273	s__CAG-273 unknown_3
MGYG-HGUT-04079:s__Clostridia unknown unknown unknown unknown_5	0.9719045 62	-0.0098724 35	0.99472706 3	d__Bacteria	p__Firmicutes_A	c__Clostridia	o__Clostridia unknown	f__Clostridia unknown unknown	g__Clostridia unknown unknown unknown	s__Clostridia unknown unknown unknown unknown_5
MGYG-HGUT-04082:s__Lachnospiraceae unknown unknown_38	0.283337731	-0.290640034	0.929025913	d__Bacteria	p__Firmicutes_A	c__Clostridia	o__Lachnospirales	f__Lachnospiraceae	g__Lachnospiraceae unknown	s__Lachnospiraceae unknown unknown_38
MGYG-HGUT-04083:s__CAG-1427 sp000435675	0.534207397	0.173159981	0.968532073	d__Bacteria	p__Actinobacteriota	c__Coriobacteriia	o__Coriobacteriales	f__Eggerthellaceae	g__CAG-1427	s__CAG-1427 sp000435675
MGYG-HGUT-04087:s__Clostridium_M unknown_16	0.614718326	-0.136864485	0.975671757	d__Bacteria	p__Firmicutes_A	c__Clostridia	o__Lachnospirales	f__Lachnospiraceae	g__Clostridium_M	s__Clostridium_M unknown_16
MGYG-HGUT-04088:s__CAG-74 unknown unknown_25	0.350869346	0.254622812	0.939709925	d__Bacteria	p__Firmicutes_A	c__Clostridia	o__Christensenellales	f__CAG-74	g__CAG-74 unknown	s__CAG-74 unknown unknown_25
MGYG-HGUT-04091:s__Collinsella unknown_413	0.685676227	0.110874231	0.975768579	d__Bacteria	p__Actinobacteriota	c__Coriobacteriia	o__Coriobacteriales	f__Coriobacteriaceae	g__Collinsella	s__Collinsella unknown_413
MGYG-HGUT-04092:s__CAG-83 unknown_16	0.177530213	0.368877352	0.891825559	d__Bacteria	p__Firmicutes_A	c__Clostridia	o__Oscillospirales	f__Oscillospiraceae	g__CAG-83	s__CAG-83 unknown_16
MGYG-HGUT-04093:s__UBA7185 unknown_4	0.308810216	0.285223463	0.929025913	d__Bacteria	p__Firmicutes_B	c__Peptococcia	o__Peptococcales	f__Peptococcaceae	g__UBA7185	s__UBA7185 unknown_4

MGYG-HGUT-04094:s__Bacteroides unknown_16	0.8134061 65	0.0663133 91	0.9871423 02	d__Bacteria	p__Bacteroidota	c__Bacteroidia	o__Bacteroidales	f__Bacteroidaceae	g__Bacteroides	s__Bacteroides unknown_16
MGYG-HGUT-04104:s__Ruminococcaceae unknown unknown_25	0.5479836 5	0.1658049 82	0.9685320 73	d__Bacteria	p__Firmicutes_A	c__Clostridia	o__Oscillospirales	f__Ruminococcaceae	g__Ruminococcaceae unknown	s__Ruminococcaceae unknown unknown_25
MGYG-HGUT-04105:s__Alistipes_A unknown_3	0.6222634 91	-0.1322965 39	0.9756717 57	d__Bacteria	p__Bacteroidota	c__Bacteroidia	o__Bacteroidales	f__Rikenellaceae	g__Alistipes_A	s__Alistipes_A unknown_3
MGYG-HGUT-04111:s__Angelakisella unknown_3	0.2520075 79	0.3166004 16	0.9021813 67	d__Bacteria	p__Firmicutes_A	c__Clostridia	o__Oscillospirales	f__Ruminococcaceae	g__Angelakisella	s__Angelakisella unknown_3
MGYG-HGUT-04114:s__CAG-95 sp000438155	0.3926202 63	0.2343911 86	0.9397092 5	d__Bacteria	p__Firmicutes_A	c__Clostridia	o__Lachnospirales	f__Lachnospiraceae	g__CAG-95	s__CAG-95 sp000438155
MGYG-HGUT-04115:s__Acutalibacteraceae unknown unknown_65	0.2211188 31	-0.3412333 84	0.8918255 59	d__Bacteria	p__Firmicutes_A	c__Clostridia	o__Oscillospirales	f__Acutalibacteraceae	g__Acutalibacteraceae unknown	s__Acutalibacteraceae unknown unknown_65
MGYG-HGUT-04116:s__Acutalibacteraceae unknown unknown_66	0.0434031 07	-0.5303387 77	0.7793210 52	d__Bacteria	p__Firmicutes_A	c__Clostridia	o__Oscillospirales	f__Acutalibacteraceae	g__Acutalibacteraceae unknown	s__Acutalibacteraceae unknown unknown_66
MGYG-HGUT-04117:s__CAG-302 sp001916775	0.7601869 61	-0.0827650 81	0.9794584 75	d__Bacteria	p__Firmicutes	c__Bacilli	o__RF39	f__CAG-302	g__CAG-302	s__CAG-302 sp001916775
MGYG-HGUT-04122:s__Butyricicoccaceae unknown unknown_5	0.83020	0.05978	0.99222	d__Bact	p__Firmicutes_A	c__Clostridia	o__Oscillospirales	f__Butyricicoccaceae	g__Butyricicoccaceae unknown	s__Butyricicoccaceae unknown unknown_5

	2093	8107	019	eria						
MGYG-HGUT-04123:s__Anaerovoracaceae unknown unknown_8	0.334320135	-0.269780081	0.930624692	d__Bacteria	p__Firmicutes_A	c__Clostridia	o__Peptostreptococcales	f__Anaerovoracaceae	g__Anaerovoracaceae unknown	s__Anaerovoracaceae unknown unknown_8
MGYG-HGUT-04130:s__UBA1777 unknown_15	0.826424978	-0.059946022	0.991936936	d__Bacteria	p__Firmicutes_A	c__Clostridia	o__Oscillospirales	f__Oscillospiraceae	g__UBA1777	s__UBA1777 unknown_15
MGYG-HGUT-04132:s__Lachnospiraceae unknown unknown_39	0.906428545	0.032664103	0.994479474	d__Bacteria	p__Firmicutes_A	c__Clostridia	o__Lachnospirales	f__Lachnospiraceae	g__Lachnospiraceae unknown	s__Lachnospiraceae unknown unknown_39
MGYG-HGUT-04136:s__Eisenbergiella unknown_8	0.975753958	0.00848268	0.996587801	d__Bacteria	p__Firmicutes_A	c__Clostridia	o__Lachnospirales	f__Lachnospiraceae	g__Eisenbergiella	s__Eisenbergiella unknown_8
MGYG-HGUT-04137:s__UBA738 sp003522945	0.435427861	0.21072058	0.950806594	d__Bacteria	p__Firmicutes_A	c__Clostridia	o__Oscillospirales	f__Oscillospiraceae	g__UBA738	s__UBA738 sp003522945
MGYG-HGUT-04140:s__CAG-56 unknown_5	0.944570185	0.019275742	0.994479474	d__Bacteria	p__Firmicutes_A	c__Clostridia	o__Lachnospirales	f__Lachnospiraceae	g__CAG-56	s__CAG-56 unknown_5
MGYG-HGUT-04142:s__Marseille-P3106 sp900169975	0.916474808	-0.028644807	0.994479474	d__Bacteria	p__Firmicutes_A	c__Clostridia	o__Oscillospirales	f__Oscillospiraceae	g__Marseille-P3106	s__Marseille-P3106 sp900169975
MGYG-HGUT-04143:s__Ruthenibacterium sp003149955	0.817987419	-0.064224063	0.987664539	d__Bacteria	p__Firmicutes_A	c__Clostridia	o__Oscillospirales	f__Ruminococcaceae	g__Ruthenibacterium	s__Ruthenibacterium sp003149955

MGYG-HGUT-04152:s__Collinsella unknown_416	0.351559227	0.251196564	0.93970925	d__Bacteria	p__Actinobacteriota	c__Coriobacteriia	o__Coriobacteriales	f__Coriobacteriaceae	g__Collinsella	s__Collinsella unknown_416
MGYG-HGUT-04153:s__UBA7102 unknown_2	0.879124571	-0.0418462 25	0.99447947 4	d__Bacteria	p__Firmicutes_A	c__Clostridia	o__Christensenellales	f__UBA1750	g__UBA7102	s__UBA7102 unknown_2
MGYG-HGUT-04157:s__Monoglobales unknown unknown_16	0.444607411	0.214058 22	0.96071758 5	d__Bacteria	p__Firmicutes_A	c__Clostridia	o__Monoglobales	f__Monoglobales unknown	g__Monoglobales unknown unknown	s__Monoglobales unknown unknown_16
MGYG-HGUT-04158:s__Dialister invisus	0.751562477	-0.0882984 21	0.97945847 5	d__Bacteria	p__Firmicutes_C	c__Negativicutes	o__Veillonellales	f__Dialisteraceae	g__Dialister	s__Dialister invisus
MGYG-HGUT-04161:s__Collinsella unknown_418	0.041657672	0.555944765	0.779321052	d__Bacteria	p__Actinobacteriota	c__Coriobacteriia	o__Coriobacteriales	f__Coriobacteriaceae	g__Collinsella	s__Collinsella unknown_418
MGYG-HGUT-04163:s__Ruminiclostridium_C unknown_4	0.176973217	0.376138352	0.891825559	d__Bacteria	p__Firmicutes_A	c__Clostridia	o__Oscillospirales	f__Oscillospiraceae	g__Ruminiclostridium_C	s__Ruminiclostridium_C unknown_4
MGYG-HGUT-04165:s__ER4 sp003522105	0.203378107	0.341386727	0.891825559	d__Bacteria	p__Firmicutes_A	c__Clostridia	o__Oscillospirales	f__Oscillospiraceae	g__ER4	s__ER4 sp003522105
MGYG-HGUT-04169:s__Lachnospiraceae unknown unknown_40	0.622072023	-0.1378377 22	0.97567175 7	d__Bacteria	p__Firmicutes_A	c__Clostridia	o__Lachnospirales	f__Lachnospiraceae	g__Lachnospiraceae unknown	s__Lachnospiraceae unknown unknown_40
MGYG-HGUT-04171:s__Anaerovoracaceae unknown unknown_9	0.649805069	-0.12457	0.97567175 7	d__Bacteria	p__Firmicutes_A	c__Clostridia	o__Peptostreptococcales	f__Anaerovoracaceae	g__Anaerovoracaceae unknown	s__Anaerovoracaceae unknown unknown_9

		3018								
MGYG-HGUT-04176:s__FJAT-27916 sp001183965	0.713540328	0.102802454	0.979458475	d__Bacteria	p__Firmicutes	c__Bacilli	o__Bacillales	f__Bacillaceae_A	g__FJAT-27916	s__FJAT-27916 sp001183965
MGYG-HGUT-04180:s__Parabacteroides unknown_16	0.850444628	-0.05254299	0.992878088	d__Bacteria	p__Bacteroidota	c__Bacteroidia	o__Bacteroidales	f__Tannerellaceae	g__Parabacteroides	s__Parabacteroides unknown_16
MGYG-HGUT-04181:s__Collinsella sp002232035_35	0.242858861	0.314898194	0.902181367	d__Bacteria	p__Actinobacteriota	c__Coriobacteriia	o__Coriobacteriales	f__Coriobacteriaceae	g__Collinsella	s__Collinsella sp002232035_35
MGYG-HGUT-04182:s__Bacteroides_A unknown_14	0.312657812	-0.282643078	0.929025913	d__Bacteria	p__Bacteroidota	c__Bacteroidia	o__Bacteroidales	f__Bacteroidaceae	g__Bacteroides_A	s__Bacteroides_A unknown_14
MGYG-HGUT-04184:s__QANG01 unknown	0.904463246	0.031820215	0.994479474	d__Bacteria	p__Firmicutes_A	c__Clostridia	o__Oscillospirales	f__Ruminococcaceae	g__QANG01	s__QANG01 unknown
MGYG-HGUT-04185:s__Bacteroides unknown_17	0.621732575	-0.137762827	0.975671757	d__Bacteria	p__Bacteroidota	c__Bacteroidia	o__Bacteroidales	f__Bacteroidaceae	g__Bacteroides	s__Bacteroides unknown_17
MGYG-HGUT-04188:s__Bacteroides unknown_18	0.521853621	0.179575757	0.968532073	d__Bacteria	p__Bacteroidota	c__Bacteroidia	o__Bacteroidales	f__Bacteroidaceae	g__Bacteroides	s__Bacteroides unknown_18
MGYG-HGUT-04194:s__UBA737 sp002431945	0.282554657	0.295087401	0.929025913	d__Bacteria	p__Firmicutes_A	c__Clostridia	o__Oscillospirales	f__Acutalibacteraceae	g__UBA737	s__UBA737 sp002431945
MGYG-HGUT-04196:s__CAG-83 sp001916855	0.40785	0.21732	0.93970	d__Bact	p__Firmicutes_A	c__Clostridia	o__Oscillospirales	f__Oscillospiraceae	g__CAG-83	s__CAG-83 sp001916855

	0928	1297	925	eria						
MGYG-HGUT-04200:s__F23-B02 sp000431075	0.645565281	-0.128891076	0.975671757	d__Bacteria	p__Firmicutes_A	c__Clostridia	o__Oscillospirales	f__Oscillospiraceae	g__F23-B02	s__F23-B02 sp000431075
MGYG-HGUT-04203:s__Acetatifactor unknown_8	0.513621137	0.182273025	0.968532073	d__Bacteria	p__Firmicutes_A	c__Clostridia	o__Lachnospirales	f__Lachnospiraceae	g__Acetatifactor	s__Acetatifactor unknown_8
MGYG-HGUT-04206:s__ER4 unknown_5	0.215271253	0.339721006	0.891825559	d__Bacteria	p__Firmicutes_A	c__Clostridia	o__Oscillospirales	f__Oscillospiraceae	g__ER4	s__ER4 unknown_5
MGYG-HGUT-04207:s__UBA6398 sp003150315	0.330381279	0.270251524	0.930624692	d__Bacteria	p__Bacteroidota	c__Bacteroidia	o__Bacteroidales	f__Bacteroidaceae	g__UBA6398	s__UBA6398 sp003150315
MGYG-HGUT-04209:s__Collinsella unknown_419	0.913784568	-0.028941475	0.994479474	d__Bacteria	p__Actinobacteriota	c__Coriobacteriia	o__Coriobacteriales	f__Coriobacteriaceae	g__Collinsella	s__Collinsella unknown_419
MGYG-HGUT-04215:s__UBA1234 unknown unknown_3	0.643512758	-0.129255801	0.975671757	d__Bacteria	p__Firmicutes_A	c__Clostridia	o__TANB77	f__UBA1234	g__UBA1234 unknown	s__UBA1234 unknown unknown_3
MGYG-HGUT-04222:s__CAG-269 unknown_26	0.216489193	-0.34471333	0.891825559	d__Bacteria	p__Firmicutes_A	c__Clostridia	o__TANB77	f__CAG-508	g__CAG-269	s__CAG-269 unknown_26
MGYG-HGUT-04231:s__CAG-382 unknown unknown_9	0.521879006	-0.174224819	0.968532073	d__Bacteria	p__Firmicutes_A	c__Clostridia	o__Oscillospirales	f__CAG-382	g__CAG-382 unknown	s__CAG-382 unknown unknown_9

MGYG-HGUT-04232:s__Terrisporobacter unknown_2	0.175763672	-0.376324464	0.891825559	d__Bacteria	p__Firmicutes_A	c__Clostridia	o__Peptostreptococcales	f__Peptostreptococcaceae	g__Terrisporobacter	s__Terrisporobacter unknown_2
MGYG-HGUT-04233:s__Fusicatenibacter unknown_3	0.494140089	0.187920233	0.968532073	d__Bacteria	p__Firmicutes_A	c__Clostridia	o__Lachnospirales	f__Lachnospiraceae	g__Fusicatenibacter	s__Fusicatenibacter unknown_3
MGYG-HGUT-04234:s__Massiliomicrobiota sp002160815	0.22159432	-0.339646612	0.891825559	d__Bacteria	p__Firmicutes	c__Bacilli	o__Erysipelotrichales	f__Erysipelatoclostridiaceae	g__Massiliomicrobiota	s__Massiliomicrobiota sp002160815
MGYG-HGUT-04235:s__Christensenellaceae unknown unknown_5	0.216601145	0.336416461	0.891825559	d__Bacteria	p__Firmicutes_A	c__Clostridia	o__Christensenellales	f__Christensenellaceae	g__Christensenellaceae unknown	s__Christensenellaceae unknown unknown_5
MGYG-HGUT-04236:s__Acutalibacteraceae unknown unknown_71	0.686480426	0.113148327	0.975768579	d__Bacteria	p__Firmicutes_A	c__Clostridia	o__Oscillospirales	f__Acutalibacteraceae	g__Acutalibacteraceae unknown	s__Acutalibacteraceae unknown unknown_71
MGYG-HGUT-04237:s__CAG-492 sp000434335	0.636600739	-0.130176766	0.975671757	d__Bacteria	p__Firmicutes_A	c__Clostridia	o__TANB77	f__CAG-508	g__CAG-492	s__CAG-492 sp000434335
MGYG-HGUT-04238:s__Senegalimassilia unknown_5	0.782060653	-0.0776363605	0.980737328	d__Bacteria	p__Actinobacteriota	c__Coriobacteriia	o__Coriobacteriales	f__Eggerthellaceae	g__Senegalimassilia	s__Senegalimassilia unknown_5
MGYG-HGUT-04245:s__CAG-138 unknown unknown_6	0.80327374	0.066457226	0.987142302	d__Bacteria	p__Firmicutes_A	c__Clostridia	o__Christensenellales	f__CAG-138	g__CAG-138 unknown	s__CAG-138 unknown unknown_6
MGYG-HGUT-04248:s__CAG-170 sp000436735	0.13463	0.39352	0.89182	d__Bact	p__Firmicutes_A	c__Clostridia	o__Oscillospirales	f__Oscillospiraceae	g__CAG-170	s__CAG-170 sp000436735

	6252	2138	5559	eria						
MGYG-HGUT-04249:s__CAG-822 unknown unknown_11	0.852707791	0.051686123	0.992878088	d__Bacteria	p__Firmicutes	c__Bacilli	o__RF39	f__CAG-822	g__CAG-822 unknown	s__CAG-822 unknown unknown_11
MGYG-HGUT-04250:s__Acetatifactor sp003447295	0.069469098	0.480758623	0.877826247	d__Bacteria	p__Firmicutes_A	c__Clostridia	o__Lachnospirales	f__Lachnospiraceae	g__Acetatifactor	s__Acetatifactor sp003447295
MGYG-HGUT-04256:s__CAG-177 sp003538135	0.876301417	0.042805635	0.994479474	d__Bacteria	p__Firmicutes_A	c__Clostridia	o__Oscillospirales	f__Acutalibacteraceae	g__CAG-177	s__CAG-177 sp003538135
MGYG-HGUT-04262:s__CAG-110 unknown_34	0.482024886	0.191929434	0.968532073	d__Bacteria	p__Firmicutes_A	c__Clostridia	o__Oscillospirales	f__Oscillospiraceae	g__CAG-110	s__CAG-110 unknown_34
MGYG-HGUT-04265:s__UBA1691 unknown_5	0.103565786	-0.427799742	0.883307256	d__Bacteria	p__Firmicutes_A	c__Clostridia	o__Oscillospirales	f__Acutalibacteraceae	g__UBA1691	s__UBA1691 unknown_5
MGYG-HGUT-04266:s__Streptococcus unknown_20	0.204677914	0.337599972	0.891825559	d__Bacteria	p__Firmicutes	c__Bacilli	o__Lactobacillales	f__Streptococcaceae	g__Streptococcus	s__Streptococcus unknown_20
MGYG-HGUT-04268:s__Pauljensenia bouchesdurhonensis	0.703066548	-0.10652022	0.979458475	d__Bacteria	p__Actinobacteriota	c__Actinobacteria	o__Actinomycetales	f__Actinomycetaceae	g__Pauljensenia	s__Pauljensenia bouchesdurhonensis
MGYG-HGUT-04269:s__Blautia_A unknown_21	0.994492878	0.00191749	0.998803346	d__Bacteria	p__Firmicutes_A	c__Clostridia	o__Lachnospirales	f__Lachnospiraceae	g__Blautia_A	s__Blautia_A unknown_21
MGYG-HGUT-04271:s__Roseburia unknown_8	0.31747	0.26010	0.92902	d__Bact	p__Firmicutes_A	c__Clostridia	o__Lachnospirales	f__Lachnospiraceae	g__Roseburia	s__Roseburia unknown_8

	2233	4744	5913	eria						
MGYG-HGUT-04275:s__Ruminococcus_C sp000437175	0.571612288	0.157856714	0.974686221	d__Bacteria	p__Firmicutes_A	c__Clostridia	o__Oscillospirales	f__Ruminococcaceae	g__Ruminococcus_C	s__Ruminococcus_C sp000437175
MGYG-HGUT-04276:s__ER4 unknown_6	0.741429098	0.087171734	0.979458475	d__Bacteria	p__Firmicutes_A	c__Clostridia	o__Oscillospirales	f__Oscillospiraceae	g__ER4	s__ER4 unknown_6
MGYG-HGUT-04280:s__Collinsella unknown_424	0.038049209	0.55968274	0.768225834	d__Bacteria	p__Actinobacteriota	c__Coriobacteriia	o__Coriobacteriales	f__Coriobacteriaceae	g__Collinsella	s__Collinsella unknown_424
MGYG-HGUT-04282:s__Ruminococcus_D sp000434695	0.497031472	-0.172273998	0.968532073	d__Bacteria	p__Firmicutes_A	c__Clostridia	o__Oscillospirales	f__Ruminococcaceae	g__Ruminococcus_D	s__Ruminococcus_D sp000434695
MGYG-HGUT-04284:s__Collinsella unknown_425	0.108722029	0.432099855	0.883307256	d__Bacteria	p__Actinobacteriota	c__Coriobacteriia	o__Coriobacteriales	f__Coriobacteriaceae	g__Collinsella	s__Collinsella unknown_425
MGYG-HGUT-04286:s__Collinsella sp002232035_37	0.220679881	0.340329233	0.891825559	d__Bacteria	p__Actinobacteriota	c__Coriobacteriia	o__Coriobacteriales	f__Coriobacteriaceae	g__Collinsella	s__Collinsella sp002232035_37
MGYG-HGUT-04290:s__Alistipes unknown_26	0.074108816	0.496508911	0.877826247	d__Bacteria	p__Bacteroidota	c__Bacteroidia	o__Bacteroidales	f__Rikenellaceae	g__Alistipes	s__Alistipes unknown_26
MGYG-HGUT-04294:s__Collinsella unknown_426	0.152911653	0.39578852	0.891825559	d__Bacteria	p__Actinobacteriota	c__Coriobacteriia	o__Coriobacteriales	f__Coriobacteriaceae	g__Collinsella	s__Collinsella unknown_426
MGYG-HGUT-04296:s__Ruminococcus_A sp000437095	0.585069434	0.151595038	0.975671757	d__Bacteria	p__Firmicutes_A	c__Clostridia	o__Lachnospirales	f__Lachnospiraceae	g__Ruminococcus_A	s__Ruminococcus_A sp000437095

MGYG-HGUT-04297:s__Collinsella unknown_427	0.010869625	0.689726644	0.768225834	d__Bacteria	p__Actinobacteriota	c__Coriobacteriia	o__Coriobacteriales	f__Coriobacteriaceae	g__Collinsella	s__Collinsella unknown_427
MGYG-HGUT-04305:s__Collinsella unknown_429	0.018256145	0.652030902	0.768225834	d__Bacteria	p__Actinobacteriota	c__Coriobacteriia	o__Coriobacteriales	f__Coriobacteriaceae	g__Collinsella	s__Collinsella unknown_429
MGYG-HGUT-04307:s__Collinsella aerofaciens_F_34	0.122923368	0.416629878	0.885742175	d__Bacteria	p__Actinobacteriota	c__Coriobacteriia	o__Coriobacteriales	f__Coriobacteriaceae	g__Collinsella	s__Collinsella aerofaciens_F_34
MGYG-HGUT-04308:s__CAG-145 sp000435615	0.465117365	0.200662807	0.968037613	d__Bacteria	p__Firmicutes_A	c__Clostridia	o__Peptostreptococcales	f__Anaerovoracaceae	g__CAG-145	s__CAG-145 sp000435615
MGYG-HGUT-04312:s__Methanomassiliicoccus_A unknown_3	0.407057118	0.230678638	0.93970925	d__Archaea	p__Thermoplasmatota	c__Thermoplasmata	o__Methanomassiliicoccales	f__Methanomassiliicoccaceae	g__Methanomassiliicoccus_A	s__Methanomassiliicoccus_A unknown_3
MGYG-HGUT-04316:s__Granulicatella adiacens	0.661660505	0.121154891	0.975671757	d__Bacteria	p__Firmicutes	c__Bacilli	o__Lactobacillales	f__Aerococcaceae	g__Granulicatella	s__Granulicatella adiacens
MGYG-HGUT-04317:s__Lachnospira sp900316325	0.192023753	0.356516411	0.891825559	d__Bacteria	p__Firmicutes_A	c__Clostridia	o__Lachnospirales	f__Lachnospiraceae	g__Lachnospira	s__Lachnospira sp900316325
MGYG-HGUT-04320:s__Collinsella unknown_431	0.187905235	0.368146142	0.891825559	d__Bacteria	p__Actinobacteriota	c__Coriobacteriia	o__Coriobacteriales	f__Coriobacteriaceae	g__Collinsella	s__Collinsella unknown_431
MGYG-HGUT-04321:s__Streptococcus constellatus	0.878301315	-0.0424700 41	0.994479474	d__Bacteria	p__Firmicutes	c__Bacilli	o__Lactobacillales	f__Streptococcaceae	g__Streptococcus	s__Streptococcus constellatus

MGYG-HGUT-04323:s__CAG-180 sp000432435	0.447319566	0.209228804	0.961292208	d__Bacteria	p__Firmicutes_A	c__Clostridia	o__Oscillospirales	f__Acutalibacteraceae	g__CAG-180	s__CAG-180 sp000432435
MGYG-HGUT-04325:s__Collinsella unknown_433	0.059667094	0.501946832	0.857211132	d__Bacteria	p__Actinobacteriota	c__Coriobacteriia	o__Coriobacteriales	f__Coriobacteriaceae	g__Collinsella	s__Collinsella unknown_433
MGYG-HGUT-04326:s__Collinsella unknown_434	0.39627594	0.231299036	0.939709925	d__Bacteria	p__Actinobacteriota	c__Coriobacteriia	o__Coriobacteriales	f__Coriobacteriaceae	g__Collinsella	s__Collinsella unknown_434
MGYG-HGUT-04328:s__Collinsella aerofaciens_F_35	0.176274211	0.360519087	0.891825559	d__Bacteria	p__Actinobacteriota	c__Coriobacteriia	o__Coriobacteriales	f__Coriobacteriaceae	g__Collinsella	s__Collinsella aerofaciens_F_35
MGYG-HGUT-04329:s__Prevotellamassilia unknown_14	0.876744453	-0.042777711	0.994479474	d__Bacteria	p__Bacteroidota	c__Bacteroidia	o__Bacteroidales	f__Bacteroidaceae	g__Prevotellamassilia	s__Prevotellamassilia unknown_14
MGYG-HGUT-04330:s__Collinsella unknown_435	0.617606777	-0.138272384	0.975671757	d__Bacteria	p__Actinobacteriota	c__Coriobacteriia	o__Coriobacteriales	f__Coriobacteriaceae	g__Collinsella	s__Collinsella unknown_435
MGYG-HGUT-04331:s__Collinsella unknown_436	0.36390184	0.24624017	0.939709925	d__Bacteria	p__Actinobacteriota	c__Coriobacteriia	o__Coriobacteriales	f__Coriobacteriaceae	g__Collinsella	s__Collinsella unknown_436
MGYG-HGUT-04332:s__Collinsella unknown_437	0.706327888	-0.104101305	0.979458475	d__Bacteria	p__Actinobacteriota	c__Coriobacteriia	o__Coriobacteriales	f__Coriobacteriaceae	g__Collinsella	s__Collinsella unknown_437
MGYG-HGUT-04334:s__Ruminococcus_E sp002493635	0.026891987	0.608263752	0.768225834	d__Bacteria	p__Firmicutes_A	c__Clostridia	o__Oscillospirales	f__Acutalibacteraceae	g__Ruminococcus_E	s__Ruminococcus_E sp002493635

MGYG-HGUT-04335:s__Collinsella unknown_438	0.38037971 1	-0.240645342	0.93970925	d__Bacteria	p__Actinobacteriota	c__Coriobacteriia	o__Coriobacteriales	f__Coriobacteriaceae	g__Collinsella	s__Collinsella unknown_438
MGYG-HGUT-04336:s__Faecalibacterium unknown_16	0.2418121 07	0.3276632 97	0.9021813 67	d__Bacteria	p__Firmicutes_A	c__Clostridia	o__Oscillospirales	f__Ruminococcaceae	g__Faecalibacterium	s__Faecalibacterium unknown_16
MGYG-HGUT-04340:s__Faecalibacterium unknown_17	0.0456336 61	0.5473218 75	0.7996670 98	d__Bacteria	p__Firmicutes_A	c__Clostridia	o__Oscillospirales	f__Ruminococcaceae	g__Faecalibacterium	s__Faecalibacterium unknown_17
MGYG-HGUT-04341:s__CAG-110 sp000435995	0.2957438 82	-0.2871723 47	0.9290259 13	d__Bacteria	p__Firmicutes_A	c__Clostridia	o__Oscillospirales	f__Oscillospiraceae	g__CAG-110	s__CAG-110 sp000435995
MGYG-HGUT-04344:s__Collinsella unknown_439	0.5955471 95	0.1451113 34	0.9756717 57	d__Bacteria	p__Actinobacteriota	c__Coriobacteriia	o__Coriobacteriales	f__Coriobacteriaceae	g__Collinsella	s__Collinsella unknown_439
MGYG-HGUT-04345:s__Streptococcus thermophilus	0.5481058 18	0.1627225 87	0.9685320 73	d__Bacteria	p__Firmicutes	c__Bacilli	o__Lactobacillales	f__Streptococcaceae	g__Streptococcus	s__Streptococcus thermophilus
MGYG-HGUT-04346:s__Collinsella unknown_440	0.4396391 1	0.2165676 75	0.9557604 5	d__Bacteria	p__Actinobacteriota	c__Coriobacteriia	o__Coriobacteriales	f__Coriobacteriaceae	g__Collinsella	s__Collinsella unknown_440
MGYG-HGUT-04349:s__Collinsella unknown_441	0.5852569 17	0.1510300 3	0.9756717 57	d__Bacteria	p__Actinobacteriota	c__Coriobacteriia	o__Coriobacteriales	f__Coriobacteriaceae	g__Collinsella	s__Collinsella unknown_441
MGYG-HGUT-04350:s__CAG-245 unknown_4	0.3241023 52	-0.2755366 54	0.9306246 92	d__Bacteria	p__Firmicutes_A	c__Clostridia	o__TANB77	f__CAG-508	g__CAG-245	s__CAG-245 unknown_4

MGYG-HGUT-04358:s__Collinsella unknown_442	0.567452209	0.154641241	0.971339903	d__Bacteria	p__Actinobacteriota	c__Coriobacteriia	o__Coriobacteriales	f__Coriobacteriaceae	g__Collinsella	s__Collinsella unknown_442
MGYG-HGUT-04359:s__Anaerostipes unknown_4	0.773720211	-0.0798946 98	0.979520484	d__Bacteria	p__Firmicutes_A	c__Clostridia	o__Lachnospirales	f__Lachnospiraceae	g__Anaerostipes	s__Anaerostipes unknown_4
MGYG-HGUT-04363:s__Collinsella unknown_443	0.211641161	0.344744142	0.891825559	d__Bacteria	p__Actinobacteriota	c__Coriobacteriia	o__Coriobacteriales	f__Coriobacteriaceae	g__Collinsella	s__Collinsella unknown_443
MGYG-HGUT-04364:s__Collinsella unknown_444	0.761601804	-0.082234335	0.979458475	d__Bacteria	p__Actinobacteriota	c__Coriobacteriia	o__Coriobacteriales	f__Coriobacteriaceae	g__Collinsella	s__Collinsella unknown_444
MGYG-HGUT-04366:s__Collinsella unknown_445	0.630708675	0.132892535	0.975671757	d__Bacteria	p__Actinobacteriota	c__Coriobacteriia	o__Coriobacteriales	f__Coriobacteriaceae	g__Collinsella	s__Collinsella unknown_445
MGYG-HGUT-04367:s__Collinsella unknown_446	0.114606065	0.428641437	0.883307256	d__Bacteria	p__Actinobacteriota	c__Coriobacteriia	o__Coriobacteriales	f__Coriobacteriaceae	g__Collinsella	s__Collinsella unknown_446
MGYG-HGUT-04368:s__Collinsella aerofaciens_A_2	0.294604497	0.263545306	0.929025913	d__Bacteria	p__Actinobacteriota	c__Coriobacteriia	o__Coriobacteriales	f__Coriobacteriaceae	g__Collinsella	s__Collinsella aerofaciens_A_2
MGYG-HGUT-04369:s__CAG-882 sp000435595	0.492987066	0.188425806	0.968532073	d__Bacteria	p__Firmicutes_A	c__Clostridia	o__Lachnospirales	f__Lachnospiraceae	g__CAG-882	s__CAG-882 sp000435595
MGYG-HGUT-04371:s__Prevotella sp002251385	0.747635454	-0.087447905	0.979458475	d__Bacteria	p__Bacteroidota	c__Bacteroidia	o__Bacteroidales	f__Bacteroidaceae	g__Prevotella	s__Prevotella sp002251385

MGYG-HGUT-04373:s__Collinsella unknown_447	0.620127752	-0.137699103	0.975671757	d__Bacteria	p__Actinobacteriota	c__Coriobacteriia	o__Coriobacteriales	f__Coriobacteriaceae	g__Collinsella	s__Collinsella unknown_447
MGYG-HGUT-04375:s__Holdemanella unknown_4	0.965110448	0.012065654	0.994479474	d__Bacteria	p__Firmicutes	c__Bacilli	o__Erysipelotrichales	f__Erysipelotrichaceae	g__Holdemanella	s__Holdemanella unknown_4
MGYG-HGUT-04376:s__CAG-83 unknown_17	0.088042281	0.45762	0.882716064	d__Bacteria	p__Firmicutes_A	c__Clostridia	o__Oscillospirales	f__Oscillospiraceae	g__CAG-83	s__CAG-83 unknown_17
MGYG-HGUT-04378:s__Collinsella unknown_449	0.629564932	0.134690009	0.975671757	d__Bacteria	p__Actinobacteriota	c__Coriobacteriia	o__Coriobacteriales	f__Coriobacteriaceae	g__Collinsella	s__Collinsella unknown_449
MGYG-HGUT-04380:s__CAG-590 unknown_5	0.461479806	0.192319399	0.965574692	d__Bacteria	p__Firmicutes_A	c__Clostridia	o__Lachnospirales	f__Lachnospiraceae	g__CAG-590	s__CAG-590 unknown_5
MGYG-HGUT-04381:s__Lachnospira unknown_4	0.768020382	0.0793807	0.979520484	d__Bacteria	p__Firmicutes_A	c__Clostridia	o__Lachnospirales	f__Lachnospiraceae	g__Lachnospira	s__Lachnospira unknown_4
MGYG-HGUT-04382:s__UBA2821 unknown	0.140238821	0.405999982	0.891825559	d__Bacteria	p__Firmicutes_A	c__Clostridia	o__Lachnospirales	f__Lachnospiraceae	g__UBA2821	s__UBA2821 unknown
MGYG-HGUT-04384:s__Ruminococcus unknown_8	0.984637992	0.005275723	0.998803346	d__Bacteria	p__Firmicutes_A	c__Clostridia	o__Oscillospirales	f__Ruminococcaceae	g__Ruminococcus	s__Ruminococcus unknown_8
MGYG-HGUT-04385:s__Lachnospira unknown_5	0.606058228	0.143738419	0.975671757	d__Bacteria	p__Firmicutes_A	c__Clostridia	o__Lachnospirales	f__Lachnospiraceae	g__Lachnospira	s__Lachnospira unknown_5

MGYG-HGUT-04386:s__Collinsella unknown_451	0.460212407	0.197382567	0.965574692	d__Bacteria	p__Actinobacteriota	c__Coriobacteriia	o__Coriobacteriales	f__Coriobacteriaceae	g__Collinsella	s__Collinsella unknown_451
MGYG-HGUT-04387:s__Collinsella unknown_452	0.145501545	0.405992047	0.891825559	d__Bacteria	p__Actinobacteriota	c__Coriobacteriia	o__Coriobacteriales	f__Coriobacteriaceae	g__Collinsella	s__Collinsella unknown_452
MGYG-HGUT-04388:s__F23-B02 sp001916715	0.084733971	0.473720618	0.882716064	d__Bacteria	p__Firmicutes_A	c__Clostridia	o__Oscillospirales	f__Oscillospiraceae	g__F23-B02	s__F23-B02 sp001916715
MGYG-HGUT-04390:s__Prevotella sp002265625	0.742934457	-0.090171946	0.979458475	d__Bacteria	p__Bacteroidota	c__Bacteroidia	o__Bacteroidales	f__Bacteroidaceae	g__Prevotella	s__Prevotella sp002265625
MGYG-HGUT-04392:s__Eubacterium_R unknown_23	0.515135947	0.179731939	0.968532073	d__Bacteria	p__Firmicutes_A	c__Clostridia	o__Oscillospirales	f__Acutalibacteraceae	g__Eubacterium_R	s__Eubacterium_R unknown_23
MGYG-HGUT-04396:s__Collinsella unknown_453	0.758816313	0.083437832	0.979458475	d__Bacteria	p__Actinobacteriota	c__Coriobacteriia	o__Coriobacteriales	f__Coriobacteriaceae	g__Collinsella	s__Collinsella unknown_453
MGYG-HGUT-04398:s__Collinsella unknown_454	0.313309118	0.277941221	0.929025913	d__Bacteria	p__Actinobacteriota	c__Coriobacteriia	o__Coriobacteriales	f__Coriobacteriaceae	g__Collinsella	s__Collinsella unknown_454
MGYG-HGUT-04399:s__Collinsella unknown_455	0.083275278	0.476297081	0.881142843	d__Bacteria	p__Actinobacteriota	c__Coriobacteriia	o__Coriobacteriales	f__Coriobacteriaceae	g__Collinsella	s__Collinsella unknown_455
MGYG-HGUT-04402:s__RC9 sp000432515	0.853169489	0.05079914	0.992878088	d__Bacteria	p__Bacteroidota	c__Bacteroidia	o__Bacteroidales	f__UBA932	g__RC9	s__RC9 sp000432515

MGYG-HGUT-04403:s__Agathobacter unknown_6	0.325289994	0.270546629	0.930624692	d__Bacteria	p__Firmicutes_A	c__Clostridia	o__Lachnospirales	f__Lachnospiraceae	g__Agathobacter	s__Agathobacter unknown_6
MGYG-HGUT-04407:s__Collinsella unknown_456	0.417528957	0.222176534	0.939709925	d__Bacteria	p__Actinobacteriota	c__Coriobacteriia	o__Coriobacteriales	f__Coriobacteriaceae	g__Collinsella	s__Collinsella unknown_456
MGYG-HGUT-04408:s__Collinsella unknown_457	0.519124572	0.173159611	0.968532073	d__Bacteria	p__Actinobacteriota	c__Coriobacteriia	o__Coriobacteriales	f__Coriobacteriaceae	g__Collinsella	s__Collinsella unknown_457
MGYG-HGUT-04412:s__CAG-83 sp900313295	0.598582137	0.141232743	0.975671757	d__Bacteria	p__Firmicutes_A	c__Clostridia	o__Oscillospirales	f__Oscillospiraceae	g__CAG-83	s__CAG-83 sp900313295
MGYG-HGUT-04413:s__CAG-488 sp003486665	0.667253755	-0.119781966	0.975671757	d__Bacteria	p__Firmicutes_A	c__Clostridia	o__Oscillospirales	f__Acutalibacteraceae	g__CAG-488	s__CAG-488 sp003486665
MGYG-HGUT-04414:s__Agathobacter unknown_7	0.043198931	0.55899618	0.779321052	d__Bacteria	p__Firmicutes_A	c__Clostridia	o__Lachnospirales	f__Lachnospiraceae	g__Agathobacter	s__Agathobacter unknown_7
MGYG-HGUT-04415:s__Collinsella unknown_458	0.038921155	0.553927154	0.768225834	d__Bacteria	p__Actinobacteriota	c__Coriobacteriia	o__Coriobacteriales	f__Coriobacteriaceae	g__Collinsella	s__Collinsella unknown_458
MGYG-HGUT-04421:s__Collinsella unknown_459	0.073498572	0.480199429	0.877826247	d__Bacteria	p__Actinobacteriota	c__Coriobacteriia	o__Coriobacteriales	f__Coriobacteriaceae	g__Collinsella	s__Collinsella unknown_459
MGYG-HGUT-04422:s__Collinsella unknown_460	0.366774509	0.251384665	0.939709925	d__Bacteria	p__Actinobacteriota	c__Coriobacteriia	o__Coriobacteriales	f__Coriobacteriaceae	g__Collinsella	s__Collinsella unknown_460

MGYG-HGUT-04431:s__COE1 sp001916965	0.685808349	0.113369103	0.975768579	d__Bacteria	p__Firmicutes_A	c__Clostridia	o__Lachnospirales	f__Lachnospiraceae	g__COE1	s__COE1 sp001916965
MGYG-HGUT-04433:s__Bilophila unknown_5	0.367877775	0.252323162	0.939709925	d__Bacteria	p__Desulfobacterota_A	c__Desulfovibrionia	o__Desulfovibrionales	f__Desulfovibrionaceae	g__Bilophila	s__Bilophila unknown_5
MGYG-HGUT-04438:s__UBA4644 unknown_3	0.219065497	0.326653395	0.891825559	d__Bacteria	p__Firmicutes_A	c__Clostridia	o__Oscillospirales	f__Butyricicoccaceae	g__UBA4644	s__UBA4644 unknown_3
MGYG-HGUT-04440:s__CAG-449 unknown_2	0.011561294	0.695692334	0.768225834	d__Bacteria	p__Firmicutes	c__Bacilli	o__RFN20	f__CAG-449	g__CAG-449	s__CAG-449 unknown_2
MGYG-HGUT-04449:s__CAG-245 unknown_5	0.688085252	0.110090944	0.975768579	d__Bacteria	p__Firmicutes_A	c__Clostridia	o__TANB77	f__CAG-508	g__CAG-245	s__CAG-245 unknown_5
MGYG-HGUT-04450:s__Collinsella unknown_463	0.299113466	-0.278232561	0.929025913	d__Bacteria	p__Actinobacteriota	c__Coriobacteriia	o__Coriobacteriales	f__Coriobacteriaceae	g__Collinsella	s__Collinsella unknown_463
MGYG-HGUT-04451:s__CAG-313 sp003539625	0.942043684	-0.020355384	0.994479474	d__Bacteria	p__Firmicutes	c__Bacilli	o__ML615J-28	f__CAG-313	g__CAG-313	s__CAG-313 sp003539625
MGYG-HGUT-04456:s__Prevotella unknown_56	0.580643247	0.151482162	0.975671757	d__Bacteria	p__Bacteroidota	c__Bacteroidia	o__Bacteroidales	f__Bacteroidaceae	g__Prevotella	s__Prevotella unknown_56
MGYG-HGUT-04463:s__CAG-83 unknown_18	0.583266066	0.14533378	0.975671757	d__Bacteria	p__Firmicutes_A	c__Clostridia	o__Oscillospirales	f__Oscillospiraceae	g__CAG-83	s__CAG-83 unknown_18

MGYG-HGUT-04466:s__Collinsella unknown_464	0.51401010164	0.17629973 8	0.96853207 3	d__Bacteria	p__Actinobacteriota	c__Coriobacteriia	o__Coriobacteriales	f__Coriobacteriaceae	g__Collinsella	s__Collinsella unknown_464
MGYG-HGUT-04467:s__Collinsella unknown_465	0.67334748 3	-0.11848403 2	0.97567175 7	d__Bacteria	p__Actinobacteriota	c__Coriobacteriia	o__Coriobacteriales	f__Coriobacteriaceae	g__Collinsella	s__Collinsella unknown_465
MGYG-HGUT-04471:s__UBA1394 unknown_3	0.37515205 5	-0.24268813 5	0.93970925	d__Bacteria	p__Firmicutes_A	c__Clostridia	o__Oscillospirales	f__Ruminococcaceae	g__UBA1394	s__UBA1394 unknown_3
MGYG-HGUT-04472:s__QALR01 unknown_3	0.16971746 9	0.38323812 6	0.89182555 9	d__Bacteria	p__Firmicutes_A	c__Clostridia	o__Oscillospirales	f__CAG-272	g__QALR01	s__QALR01 unknown_3
MGYG-HGUT-04475:s__CAG-83 unknown_19	0.61133692 9	0.13736561 5	0.97567175 7	d__Bacteria	p__Firmicutes_A	c__Clostridia	o__Oscillospirales	f__Oscillospiraceae	g__CAG-83	s__CAG-83 unknown_19
MGYG-HGUT-04477:s__CAG-273 unknown_5	0.73198942	0.09499598 4	0.97945847 5	d__Bacteria	p__Firmicutes_A	c__Clostridia	o__TANB77	f__CAG-508	g__CAG-273	s__CAG-273 unknown_5
MGYG-HGUT-04479:s__Bacteroides_A mediterraneensis	0.68247884 5	0.11466991 4	0.97567175 7	d__Bacteria	p__Bacteroidota	c__Bacteroidia	o__Bacteroidales	f__Bacteroidaceae	g__Bacteroides_A	s__Bacteroides_A mediterraneensis
MGYG-HGUT-04480:s__Collinsella unknown_466	0.70611057 7	0.10591838 2	0.97945847 5	d__Bacteria	p__Actinobacteriota	c__Coriobacteriia	o__Coriobacteriales	f__Coriobacteriaceae	g__Collinsella	s__Collinsella unknown_466
MGYG-HGUT-04481:s__UBA1191 unknown_8	0.49272559 1	0.18920562 3	0.96853207 3	d__Bacteria	p__Firmicutes_A	c__Clostridia	o__Peptostreptococcales	f__Anaerovoraceae	g__UBA1191	s__UBA1191 unknown_8

MGYG-HGUT-04482:s__ER4 unknown_7	0.139071806	0.401666174	0.891825559	d__Bacteria	p__Firmicutes_A	c__Clostridia	o__Oscillospirales	f__Oscillospiraceae	g__ER4	s__ER4 unknown_7
MGYG-HGUT-04486:s__F23-B02 sp003533405	0.579599862	0.152725485	0.975671757	d__Bacteria	p__Firmicutes_A	c__Clostridia	o__Oscillospirales	f__Oscillospiraceae	g__F23-B02	s__F23-B02 sp003533405
MGYG-HGUT-04487:s__CAG-83 unknown_20	0.176829192	0.366906388	0.891825559	d__Bacteria	p__Firmicutes_A	c__Clostridia	o__Oscillospirales	f__Oscillospiraceae	g__CAG-83	s__CAG-83 unknown_20
MGYG-HGUT-04489:s__ER4 unknown_8	0.232123343	0.320649501	0.902181367	d__Bacteria	p__Firmicutes_A	c__Clostridia	o__Oscillospirales	f__Oscillospiraceae	g__ER4	s__ER4 unknown_8
MGYG-HGUT-04491:s__Prevotella unknown_58	0.674662709	0.115069846	0.975671757	d__Bacteria	p__Bacteroidota	c__Bacteroidia	o__Bacteroidales	f__Bacteroidaceae	g__Prevotella	s__Prevotella unknown_58
MGYG-HGUT-04496:s__Acutalibacteraceae unknown unknown_76	0.209265132	0.349371351	0.891825559	d__Bacteria	p__Firmicutes_A	c__Clostridia	o__Oscillospirales	f__Acutalibacteraceae	g__Acutalibacteraceae unknown	s__Acutalibacteraceae unknown unknown_76
MGYG-HGUT-04497:s__Collinsella aerofaciens_F_37	0.738095112	-0.092795773	0.979458475	d__Bacteria	p__Actinobacteriota	c__Coriobacteriia	o__Coriobacteriales	f__Coriobacteriaceae	g__Collinsella	s__Collinsella aerofaciens_F_37
MGYG-HGUT-04500:s__Oscillibacter unknown_21	0.204195666	0.346102241	0.891825559	d__Bacteria	p__Firmicutes_A	c__Clostridia	o__Oscillospirales	f__Oscillospiraceae	g__Oscillibacter	s__Oscillibacter unknown_21
MGYG-HGUT-04505:s__Collinsella unknown_468	0.735092899	0.091358385	0.979458475	d__Bacteria	p__Actinobacteriota	c__Coriobacteriia	o__Coriobacteriales	f__Coriobacteriaceae	g__Collinsella	s__Collinsella unknown_468

MGYG-HGUT-04506:s__Collinsella unknown_469	0.806277012	0.066471479	0.987142302	d__Bacteria	p__Actinobacteriota	c__Coriobacteriia	o__Coriobacteriales	f__Coriobacteriaceae	g__Collinsella	s__Collinsella unknown_469
MGYG-HGUT-04514:s__Collinsella unknown_470	0.448706596	-0.206133462	0.961292208	d__Bacteria	p__Actinobacteriota	c__Coriobacteriia	o__Coriobacteriales	f__Coriobacteriaceae	g__Collinsella	s__Collinsella unknown_470
MGYG-HGUT-04520:s__Ruminococcus_E bromii_A	0.258062899	0.313480617	0.907792127	d__Bacteria	p__Firmicutes_A	c__Clostridia	o__Oscillospirales	f__Acutalibacteraceae	g__Ruminococcus_E	s__Ruminococcus_E bromii_A
MGYG-HGUT-04522:s__Collinsella unknown_471	0.157130606	0.385094901	0.891825559	d__Bacteria	p__Actinobacteriota	c__Coriobacteriia	o__Coriobacteriales	f__Coriobacteriaceae	g__Collinsella	s__Collinsella unknown_471
MGYG-HGUT-04524:s__Collinsella unknown_472	0.109879119	0.43149607	0.883307256	d__Bacteria	p__Actinobacteriota	c__Coriobacteriia	o__Coriobacteriales	f__Coriobacteriaceae	g__Collinsella	s__Collinsella unknown_472
MGYG-HGUT-04525:s__CAG-83 unknown_21	0.558080278	0.150960544	0.968532073	d__Bacteria	p__Firmicutes_A	c__Clostridia	o__Oscillospirales	f__Oscillospiraceae	g__CAG-83	s__CAG-83 unknown_21
MGYG-HGUT-04526:s__CAG-103 sp000432375	0.779784668	-0.078459311	0.979520484	d__Bacteria	p__Firmicutes_A	c__Clostridia	o__Oscillospirales	f__Oscillospiraceae	g__CAG-103	s__CAG-103 sp000432375
MGYG-HGUT-04534:s__CAG-245 sp000434195	0.475701906	-0.19861331	0.968532073	d__Bacteria	p__Firmicutes_A	c__Clostridia	o__TANB77	f__CAG-508	g__CAG-245	s__CAG-245 sp000434195
MGYG-HGUT-04542:s__Collinsella unknown_473	0.213697128	0.338570486	0.891825559	d__Bacteria	p__Actinobacteriota	c__Coriobacteriia	o__Coriobacteriales	f__Coriobacteriaceae	g__Collinsella	s__Collinsella unknown_473

MGYG-HGUT-04544:s__Alistipes sp900290115	0.3555971107	0.256957084	0.939709 25	d__Bacteria	p__Bacteroidota	c__Bacteroidia	o__Bacteroidales	f__Rikenellaceae	g__Alistipes	s__Alistipes sp900290115
MGYG-HGUT-04548:s__Lachnospiraceae unknown unknown_42	0.4144156 41	0.2257007 74	0.939709 25	d__Bacteria	p__Firmicutes_A	c__Clostridia	o__Lachnospirales	f__Lachnospiraceae	g__Lachnospiraceae unknown	s__Lachnospiraceae unknown unknown_42
MGYG-HGUT-04552:s__UBA7096 unknown_8	0.8004007 31	-0.0676719 71	0.9871056 64	d__Bacteria	p__Firmicutes_A	c__Clostridia	o__Lachnospirales	f__Lachnospiraceae	g__UBA7096	s__UBA7096 unknown_8
MGYG-HGUT-04558:s__Faecalicatena unknown_16	0.0801062 71	-0.4779527 76	0.8778262 47	d__Bacteria	p__Firmicutes_A	c__Clostridia	o__Lachnospirales	f__Lachnospiraceae	g__Faecalicatena	s__Faecalicatena unknown_16
MGYG-HGUT-04560:s__UBA737 unknown_18	0.6866216 61	-0.1127235 92	0.9757685 79	d__Bacteria	p__Firmicutes_A	c__Clostridia	o__Oscillospirales	f__Acutalibacteraceae	g__UBA737	s__UBA737 unknown_18
MGYG-HGUT-04562:s__Gemella morbillorum	0.9093315 08	-0.0313910 26	0.9947947 74	d__Bacteria	p__Firmicutes	c__Bacilli	o__Staphylococcales	f__Gemellaceae	g__Gemella	s__Gemella morbillorum
MGYG-HGUT-04571:s__UBA6382 unknown	0.7195044 54	-0.0945015 19	0.9794584 75	d__Bacteria	p__Bacteroidota	c__Bacteroidia	o__Bacteroidales	f__Bacteroidaceae	g__UBA6382	s__UBA6382 unknown
MGYG-HGUT-04572:s__Dorea unknown_6	0.5218101 82	-0.1775289 79	0.9685320 73	d__Bacteria	p__Firmicutes_A	c__Clostridia	o__Lachnospirales	f__Lachnospiraceae	g__Dorea	s__Dorea unknown_6

MGYG-HGUT-04579:s__Coprococcus_B unknown_2	0.277552363	0.284546094	0.929025913	d__Bacteria	p__Firmicutes_A	c__Clostridia	o__Lachnospirales	f__Lachnospiraceae	g__Coprococcus_B	s__Coprococcus_B unknown_2
MGYG-HGUT-04580:s__Oscillospiraceae unknown unknown_23	0.618243102	0.133239619	0.975671757	d__Bacteria	p__Firmicutes_A	c__Clostridia	o__Oscillospirales	f__Oscillospiraceae	g__Oscillospiraceae unknown	s__Oscillospiraceae unknown unknown_23
MGYG-HGUT-04581:s__Coprococcus_A unknown	0.220516821	0.339070328	0.891825559	d__Bacteria	p__Firmicutes_A	c__Clostridia	o__Lachnospirales	f__Lachnospiraceae	g__Coprococcus_A	s__Coprococcus_A unknown
MGYG-HGUT-04582:s__CAG-81 unknown_2	0.554652025	-0.164456283	0.968532073	d__Bacteria	p__Firmicutes_A	c__Clostridia	o__Lachnospirales	f__Lachnospiraceae	g__CAG-81	s__CAG-81 unknown_2
MGYG-HGUT-04586:s__Ruminococcus_C unknown_3	0.306720114	0.284362253	0.929025913	d__Bacteria	p__Firmicutes_A	c__Clostridia	o__Oscillospirales	f__Ruminococcaceae	g__Ruminococcus_C	s__Ruminococcus_C unknown_3
MGYG-HGUT-04593:s__CAG-194 sp000432915	0.668079833	0.115766163	0.975671757	d__Bacteria	p__Firmicutes_A	c__Clostridia	o__Lachnospirales	f__Lachnospiraceae	g__CAG-194	s__CAG-194 sp000432915
MGYG-HGUT-04597:s__Collinsella unknown_478	0.039120622	0.560787919	0.768225834	d__Bacteria	p__Actinobacteriota	c__Coriobacteriia	o__Coriobacteriales	f__Coriobacteriaceae	g__Collinsella	s__Collinsella unknown_478
MGYG-HGUT-04599:s__Bacteroides_A unknown_15	0.623019164	0.137123447	0.975671757	d__Bacteria	p__Bacteroidota	c__Bacteroidia	o__Bacteroidales	f__Bacteroidaceae	g__Bacteroides_A	s__Bacteroides_A unknown_15
MGYG-HGUT-04600:s__CAG-307 sp001916215	0.632957581	0.134123508	0.975671757	d__Bacteria	p__Firmicutes	c__Bacilli	o__Acholeplasmatales	f__CAG-307	g__CAG-307	s__CAG-307 sp001916215

MGYG-HGUT-04602:s__Collinsella aerofaciens_F_39	0.017664817	0.636291924	0.768225834	d__Bacteria	p__Actinobacteriota	c__Coriobacteriia	o__Coriobacteriales	f__Coriobacteriaceae	g__Collinsella	s__Collinsella aerofaciens_F_39
MGYG-HGUT-04603:s__Streptococcus oralis_V	0.470595044	0.199707866	0.968194079	d__Bacteria	p__Firmicutes	c__Bacilli	o__Lactobacillales	f__Streptococcaceae	g__Streptococcus	s__Streptococcus oralis_V
MGYG-HGUT-04607:s__Collinsella unknown_479	0.033162614	0.589964622	0.768225834	d__Bacteria	p__Actinobacteriota	c__Coriobacteriia	o__Coriobacteriales	f__Coriobacteriaceae	g__Collinsella	s__Collinsella unknown_479
MGYG-HGUT-04608:s__Collinsella unknown_480	0.689863053	0.111312806	0.975768579	d__Bacteria	p__Actinobacteriota	c__Coriobacteriia	o__Coriobacteriales	f__Coriobacteriaceae	g__Collinsella	s__Collinsella unknown_480
MGYG-HGUT-04609:s__Faecalicatena unknown_17	0.299337447	-0.289948772	0.929025913	d__Bacteria	p__Firmicutes_A	c__Clostridia	o__Lachnospirales	f__Lachnospiraceae	g__Faecalicatena	s__Faecalicatena unknown_17
MGYG-HGUT-04610:s__Faecalicoccus unknown_2	0.080003034	0.487214569	0.877826247	d__Bacteria	p__Firmicutes	c__Bacilli	o__Erysipelotrichales	f__Erysipelotrichaceae	g__Faecalicoccus	s__Faecalicoccus unknown_2
MGYG-HGUT-04618:s__Anaerotruncus sp900199635	0.238831947	-0.323063272	0.902181367	d__Bacteria	p__Firmicutes_A	c__Clostridia	o__Oscillospirales	f__Ruminococcaceae	g__Anaerotruncus	s__Anaerotruncus sp900199635
MGYG-HGUT-04623:s__Blautia_A unknown_22	0.178744748	0.374324588	0.891825559	d__Bacteria	p__Firmicutes_A	c__Clostridia	o__Lachnospirales	f__Lachnospiraceae	g__Blautia_A	s__Blautia_A unknown_22
MGYG-HGUT-04625:s__Collinsella unknown_482	0.791578412	0.073304841	0.9829663	d__Bacteria	p__Actinobacteriota	c__Coriobacteriia	o__Coriobacteriales	f__Coriobacteriaceae	g__Collinsella	s__Collinsella unknown_482

MGYG-HGUT-04628:s__Lawsonibacter unknown_11	0.629691675	-0.134980025	0.975671757	d__Bacteria	p__Firmicutes_A	c__Clostridia	o__Oscillospirales	f__Oscillospiraceae	g__Lawsonibacter	s__Lawsonibacter unknown_11
MGYG-HGUT-04631:s__Mogibacterium sp900315625	0.975144396	0.008745419	0.996587801	d__Bacteria	p__Firmicutes_A	c__Clostridia	o__Peptostreptococcales	f__Anaerovoraceae	g__Mogibacterium	s__Mogibacterium sp900315625
MGYG-HGUT-04633:s__Erysipelatoclostridium sp002160495	0.661523055	0.122930436	0.975671757	d__Bacteria	p__Firmicutes	c__Bacilli	o__Erysipelotrichales	f__Erysipelatoclostridiaceae	g__Erysipelatoclostridium	s__Erysipelatoclostridium sp002160495
MGYG-HGUT-04643:s__Eubacterium_R sp003526845	0.141058313	0.398044387	0.891825559	d__Bacteria	p__Firmicutes_A	c__Clostridia	o__Oscillospirales	f__Acutalibacteraceae	g__Eubacterium_R	s__Eubacterium_R sp003526845
MGYG-HGUT-04644:s__Negativibacillus unknown_4	0.937306133	0.021418582	0.994479474	d__Bacteria	p__Firmicutes_A	c__Clostridia	o__Oscillospirales	f__Ruminococcaceae	g__Negativibacillus	s__Negativibacillus unknown_4

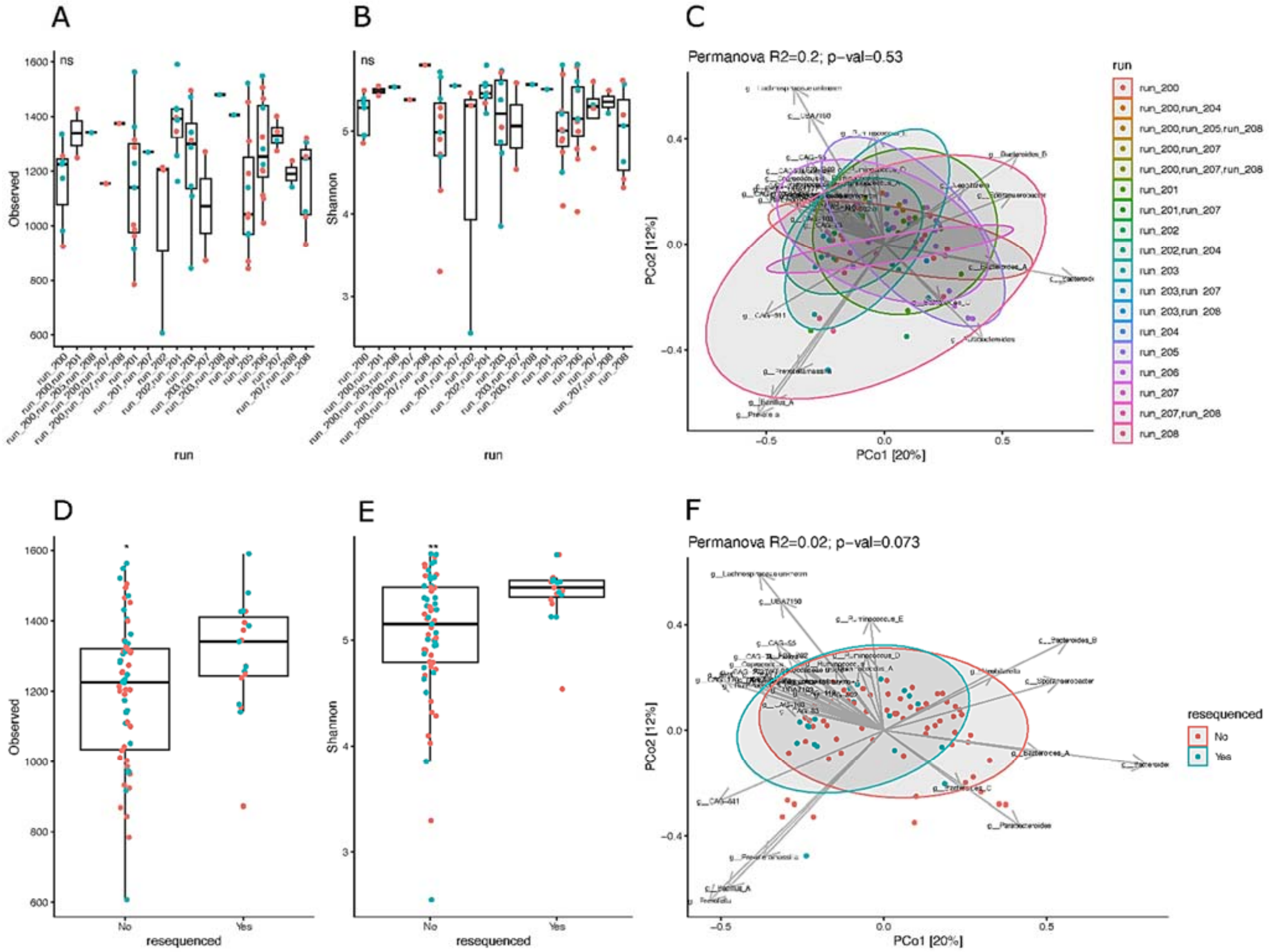

Supplemental Figure 1: Assessment of batch effects associated with the Nanopore sequencing process. Boxplot of species richness (A) and Shannon diversity (B) across samples sequenced in different runs (ns=p-value>0.05 Kruskal-Wallis test). (C) PCoA ordination based on Bray-Curtis beta-diversity matrix computed from genus-level abundance data. Samples are colored based on the sequencing run. PERMANOVA results of the impact of sequencing run on microbiome composition is shown at the top of the PCoA. Abundance vectors of 18 bacterial genera with the most significant associations with the k=3 enterotypes (FDR<0.05; Kruskal-Wallis test; Supplemental Figure 2) are fitted on the ordination plot with envfit function of the vegan R package. (D) Boxplot of species richness across samples that have been sequenced >1 time (resequenced=Yes) vs. samples sequenced only once (resequenced=No). *=p-value<0.05, Wilcoxon rank-sum test. (E) Same as D for Shannon's diversity. **=p-value<0.01 Wilcoxon rank-sum test. (F) Same as C coloring samples by the resequencing status.

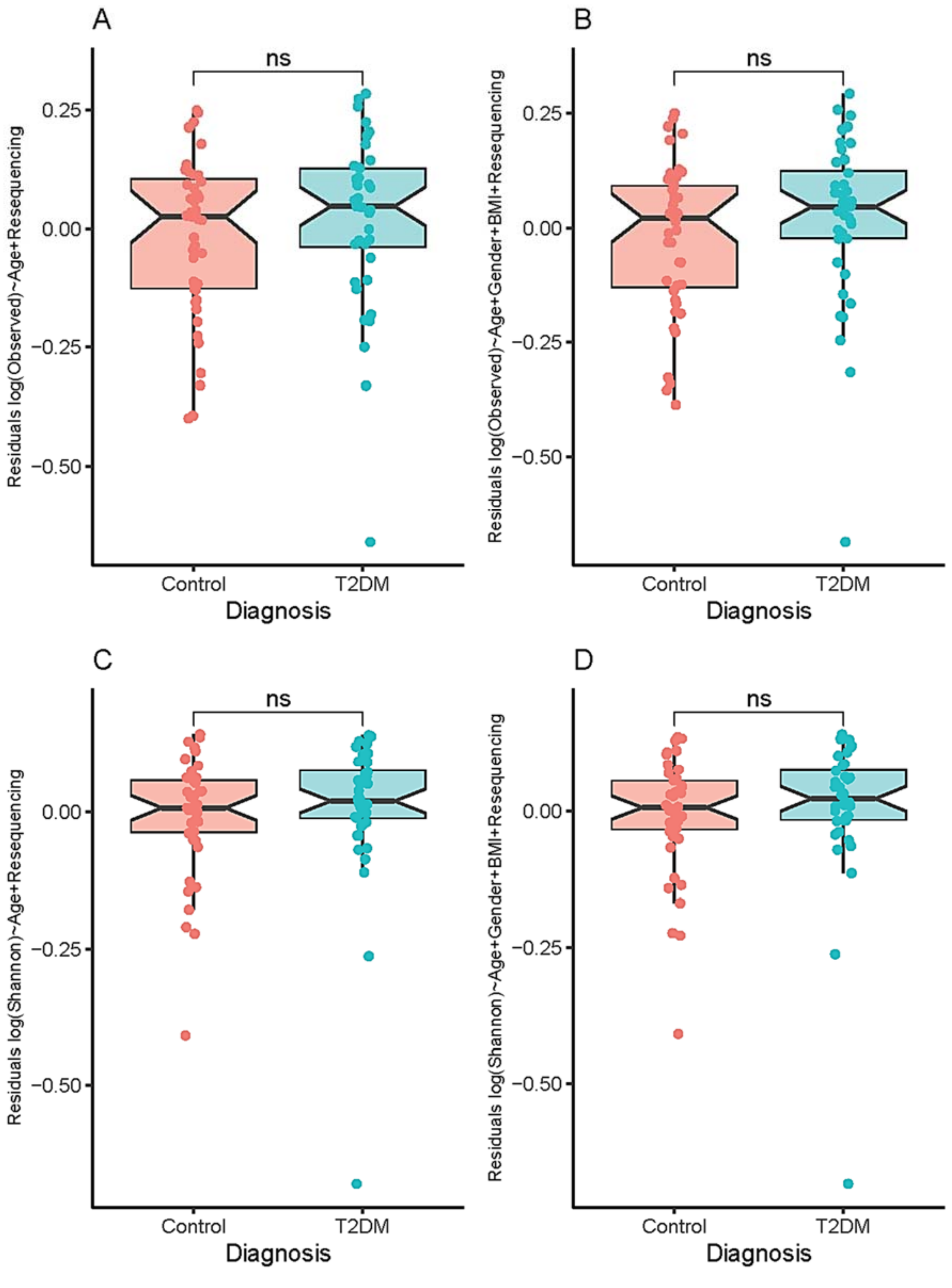

Supplemental Figure 2: Cofounder-adjusted diversity distributions across the study cohort.
(A) and (B) panels represent the distributions of the residuals of linear regression models of species richness by Age+Resequencing status and Age+gender+BMI+Resequencing status respectively between T2DM and Control groups. (C) and (D) panels correspond to the same analyses for species evenness (Shannon index). ns=p-value<0.05; Wilcoxon rank-sum test.

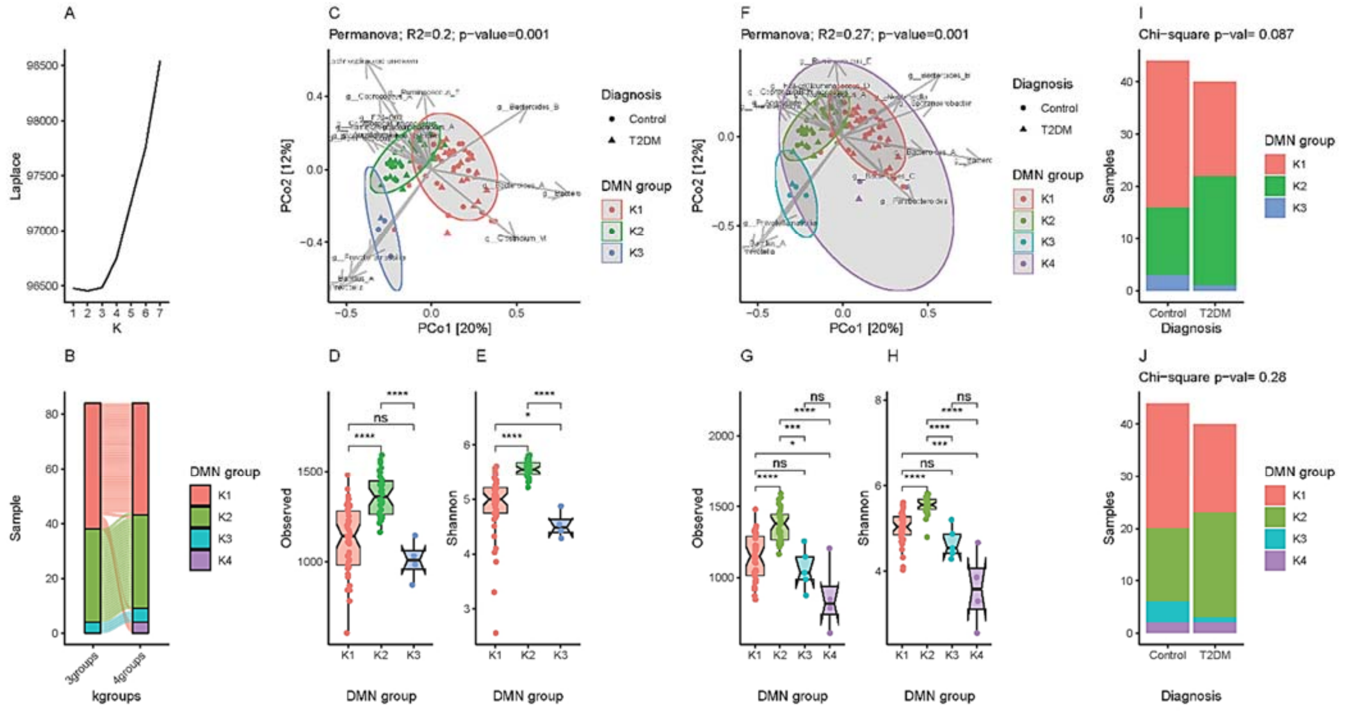

Supplemental Figure 3: Enterotype composition of the study cohort. (A) Line plot of the minimum Laplace metric (Information criterium) used to define the optimal number of clusters (enterotypes) in the study cohort (N=84 individuals) with Dirichlet-Multinomial-Mixture (DMM)-based community-typing. (B) Alluvial plot representing the change in enterotype assignment between K=3 and K=4 in the DMM approach. (C) PCoA (genus-level Bray-Curtis beta-diversity) visualizing the stratification at K=3 enterotypes (colored data points) resulting from community-typing using the DMM approach (on genus-level fecal microbiome profiles, with a shape representing the clinical group. Abundance vectors of 18 bacterial genera with significant association with the k=3 enterotypes (FDR<0.05; Kruskal-Wallis test) are fitted on the ordination plot with envfit function of vegan R package. (D) Species richness and E) evenness distributions across k=4 enterotypes (ns=non-significant; **=p-value<0.01; ****=p- value<0.0001. Wilcoxon rank-sum test). (F) Same as (B) coloring points by the stratification at K=4 groups. (G) and (H) same as (D) and (E) respectively for the stratification of the cohort into k=4 groups (I) Enterotype composition of the control (n=44) and T2DM (n=40) groups based on the 3 groups decomposition of the study cohort with the DMM approach. (J) same as (I) for the stratification of the cohort into k=4 groups.

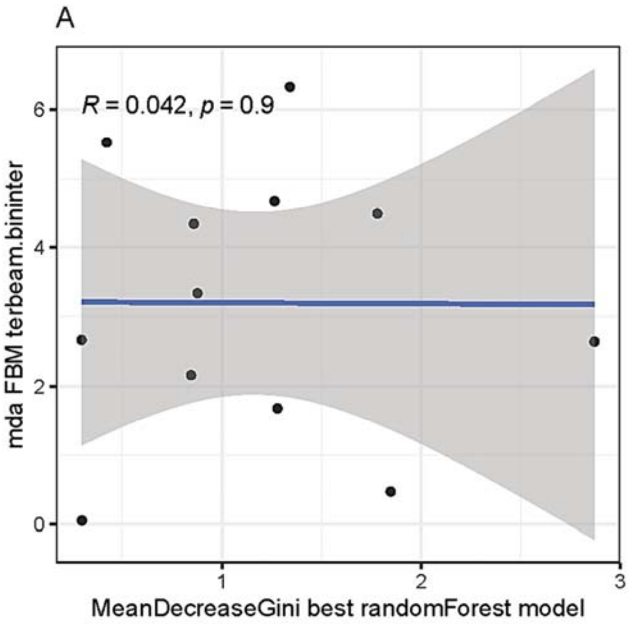

Supplementary Figure 4: Comparison of feature importance values of features in Predomics models vs. best Random Forest models. (A) Scatterplot of the mean decrease accuracy (mda) of 12 species features in the Family of Best Models (FBM) retrieved by Predomics on species abundance data (Figure 4E) and the corresponding feature importance in the best Random Forest model (Mean Decrease Gini) for the same classification task (control vs. T2D groups). (B) Same as (A) with the 14 species features in the FBM retrieved by Predomics on species presence/absence data (Figure 5E).
